# Public Perceptions of Aquaculture: Evaluating Spatiotemporal Patterns of Sentiment around the World

**DOI:** 10.1371/journal.pone.0169281

**Published:** 2017-01-03

**Authors:** Halley E. Froehlich, Rebecca R. Gentry, Michael B. Rust, Dietmar Grimm, Benjamin S. Halpern

**Affiliations:** 1 National Center for Ecological Analysis and Synthesis, University of California, Santa Barbara, California, United States of America; 2 Bren School of Environmental Science and Management, University of California, Santa Barbara, Santa Barbara, California, United States of America; 3 Office of Aquaculture, National Oceanic and Atmospheric Administration, Silver Spring, Maryland, United States of America; 4 The Nature Conservancy, 4–2 Qijiayuan Diplomatic Compound, Chaoyang District, Beijing, China; 5 Imperial College London, Silwood Park Campus, Ascot, United Kingdom; University of Regina, CANADA

## Abstract

Aquaculture is developing rapidly at a global scale and sustainable practices are an essential part of meeting the protein requirements of the ballooning human population. Locating aquaculture offshore is one strategy that may help address some issues related to nearshore development. However, offshore production is nascent and distinctions between the types of aquatic farming may not be fully understood by the public–important for collaboration, research, and development. Here we evaluate and report, to our knowledge, the first multinational quantification of the relative sentiments and opinions of the public around distinct forms of aquaculture. Using thousands of newspaper headlines (N_total_ = 1,596) from developed (no. countries = 26) and developing (42) nations, ranging over periods of 1984 to 2015, we found an expanding positive trend of general ‘aquaculture’ coverage, while ‘marine’ and ‘offshore’ appeared more negative. Overall, developing regions published proportionally more positive than negative headlines than developed countries. As case studies, government collected public comments (N_total_ = 1,585) from the United States of America (USA) and New Zealand mirrored the media sentiments; offshore perception being particularly negative in the USA. We also found public sentiment may be influenced by local environmental disasters not directly related to aquaculture (e.g., oil spills). Both countries voiced concern over environmental impacts, but the concerns tended to be more generalized, rather than targeted issues. Two factors that could be inhibiting informed discussion and decisions about offshore aquaculture are lack of applicable knowledge and actual local development issues. Better communication and investigation of the real versus perceived impacts of aquaculture could aid in clarifying the debate about aquaculture, and help support future sustainable growth.

## Introduction

Aquaculture is expanding quickly worldwide, and marine-based production may have potential in helping meet the needs and demands of the ever-expanding appetite of the human population [1–3]. Indeed, capture fisheries appear to have stagnated or declined [4], while aquaculture continues to be one of the most rapidly growing industries on the planet [3]. Although aquaculture as a whole is expanding, marine aquaculture accounts for a smaller percentage of current production (about one third) compared to more inland fish-farming [3]. Given inherent constraints to growth of land-based and nearshore coastal aquaculture (e.g., use of land, freshwater, energy, space use conflicts, etc.), opportunities for sustainable growth in aquaculture may be from open-ocean farming–typically referred to as offshore aquaculture. Numerous countries are practicing or establishing offshore farming, including the United States of America, Canada, New Zealand, Australia, and several other countries in the EU [5,6]. Developing nations, such as Morocco and Mexico, have also been documented perusing offshore aquaculture [5,6]. Yet, to date offshore production is small compared to more common forms of aquaculture.

Part of the growing interest in offshore aquaculture is the potential for improved sustainability. By moving farther offshore into the less protected ocean environment, open-ocean farming has the potential to reduce some of the many negative impacts associated with more nearshore practices and even create positive impacts through greater resource efficiency use [6,7]. Indeed, some poorly managed nearshore aquaculture sites and species can cause water-quality deterioration, transfer disease to wild populations, and increase invasive species risks, among other impacts [8–10]. Farmed salmon and shrimp species have particularly bad reputations [11–13], but sustainable management practices are improving the environmental record of these industries. Although empirical evidence is limited for offshore farming, the faster currents, deeper waters, and greater distances from important coastal ecosystems appear to support ecologically sustainable production at higher levels, especially if sited well [5,6]. However, the distinction between offshore and other types of aquaculture may not be apparent to the public.

If open-ocean aquaculture is to be responsibly developed, there is a need to fully comprehend what is inhibiting or enabling growth, and public perception is part of that understanding. Perceptions can influence the acceptance, investigation, and implementation of aquaculture [14,15]. Although social science is a rather new approach for the aquaculture field [16], several studies have reported negative connotations in country level news media or local surveys [16–22]. Public concerns appear to range from environmental to health related, but it is difficult to infer whether or how these general concerns and trends of sentiment compare to the relatively new area of offshore aquaculture. Yet, being able to identify key distinctions between aquaculture types and feelings of the public could help target science, management, and communication efforts for various stakeholders [14,23].

Media acts as an intermediary for scientific information reaching the public, particularly in the food sector [24,25]. In fact, studies have found that the majority of consumers receive information about the food industry through popular press and television [26,27]. Although the magnitude of influence the media has on public perception is convoluted, mass media does appear to affect and/or reflect a level of people’s opinions [16,24]. As a result, use of media sources, such as news articles, as a proxy for public sentiment has been applied in many contexts, including aquaculture [16,17,24,28]. However, it can be difficult to quantify the overall perception of news, particularly at larger temporal and/or spatial scales, due to the many topics and framings of an argument that can occur in a single article. As a result, studies that have used media to gauge sentiment and opinions around aquaculture have done so for only a few select developed countries and time periods [16,17,28].

In a society of online interaction and immediacy, a larger proportion of the public appear to depend on headlines for quick information [29,30]. News headlines offer rapid context, tone, and initial, and perhaps most pressing, interpretation of content in an article [24,31,32]. Importantly, headlines appear to influence the reader’s perception of an article or topic that they do eventually read [33–35]. As a result, headlines potentially provide a basic, but rapid, metric to gauge sentiment of a topic, such as aquaculture, across larger time scales and geographic regions that cannot easily be achieved using the full articles. Although identifying the precise drivers and opinions require more detailed assessment–ideally including more explicitly public-based data sources (e.g., survey, public comments)–headlines can offer a generalized sentiment measure for comparison.

Our research assesses the public sentiment around aquaculture by investigating **(1)** how it differs over large spatial and temporal scales, **(2)** how it compares to sentiment of marine and offshore practices, and **(3)** what the most common public opinions are, negative and positive, relative to actual marine and offshore policy and proposals for expansion. First, we use newspaper headlines to explore the general baseline trends of sentiment between aquaculture, marine aquaculture, and offshore aquaculture internationally (developed versus developing) and over time. Second, knowing media coverage doesn’t necessarily translate to or emerge from public perception, we conduct an in-depth analysis of government solicited public comments from two countries to compare overall sentiment to what is represented in the media and discern the most voiced public concerns and supporting opinions. The two countries (the United States of America and New Zealand) chosen as case studies are involved in aquaculture in different levels of capacity, were well captured by our headline analyses, and have accessible public comments submitted to their respective governments. Moreover, we begin to disentangle the differences in public opinion with regard to general (i.e., national) policy versus local development. Lastly, we explore the primary stakeholder groups driving the perceived patterns. Ultimately, the media assessment allows for more general, large-scale exploration of public trends, while the government comments provide a finer-scale evaluation and comparison of actual public opinion.

## Materials & Methods

### Headlines

Headlines pertaining to ‘aquaculture’, ‘marine aquaculture’, or ‘offshore aquaculture’ were extracted using the common text-data platform of LexisNexis^®^, which provides access to an array of archived media sources, including newspapers, blogs, and magazines [36]. LexisNexis^®^ identifies articles that reference the selected search term(s) anywhere in the title or text using Boolean logic; thus, not all headlines explicitly reference a search term. We focused our evaluation on newspaper headlines (which include online newspaper articles; N_total_ = 1,596) under the assumption that the information source is more mainstream and reliable in the public arena [37]. Although we did not include other forms of media, according to the LexisNexis^®^ metadata of the aquaculture search terms the vast majority (80%) of all references resided in newspapers. In addition to collecting the specific headline text, we also compiled published year, geographic origin, and publication source in order to differentiate distinct temporal and spatial trends across the globe (S1 Table).

Since only English headlines were evaluated and thus bias the results towards English speaking countries, we corrected for some of the bias by separately evaluating trends based on *developing* (n_articles_ = 1,165; n_countries_ = 26) versus *developed* (n_articles_ = 430; n_countries_ = 42) nations. While sample sizes are still greater for English speaking countries, we are capturing and better reflecting the trends of developing nations, where English may not be a primary language. Overall, headlines from 68 countries in 6 continents, spanning as far back as 1984 were included. Other terms, such as ‘fish farming,’ did not significantly increase sample size and tended to be captured by the aquaculture search terms.

Two co-authors (H.E. Froehlich and R.R. Gentry) processed headlines independently based on a metric of sentiment. Specifically, positive, negative, or neutral sounding headlines were assigned a 1, -1, or 0, respectively. Any discrepancies in a headline resulted in reevaluation and final sentiment determination. Over 75% of all headlines were categorized with the same sentiment, only 1.2% were assigned complete opposite polarity (-1 vs. 1), and the remaining 23.5% of headlines deviated positively or negatively from a neutral classification. For the few absolute contradictory sentiments, each of those headlines was re-read and the researchers reached a consensus. Conversely, if a positive or negative value was given against a neutral categorization (14.1% and 9.4%, respectively), the non-neutral sentiment was assigned to capture any and all polarizing sentiment, regardless the strength of feeling. The overall frequency of sentiment was then compared across time and geographic region. We tried to identify ‘region’ down to the country level based on the geographic and source information provided by LexisNexis^®^. This process was performed separately for ‘aquaculture’, ‘marine aquaculture’, and ‘offshore aquaculture’ (topic) headlines.

Linear modelling (LM) was used to determine statistical significance of the total number of news headlines (positive, negative, and neutral) and sentiment difference (Δ_sentiment_ = no. positive headlines–no. negative headlines) over time. Analyzing the absolute temporal trends of all articles provided the general patterns of press coverage. Evaluating the difference between the number of positive and negative (Δ_sentiment_) headlines gave a single response variable of measurement and magnitude of sentiment over time. We separately modeled both response variables for each aquaculture topic (‘aquaculture’, ‘marine aquaculture’, ‘offshore aquaculture’) given *nation type* (developed, developing), *year*, and an interaction term (nation:year). The same method(s) could not be performed at the country (instead of nation) level due to the larger discrepancies in sample sizes. However, overall geographic frequency and proportional patterns were still numerically compared and referenced. It should also be noted that although the rate estimates and proportion of headlines might contain some biases due to the limitations of the LexisNexis^®^ platform, the overall relative differences in sentiments are still informative.

### Government public comments

In order to compare news headlines to actual public sentiment and evaluate public perception of aquaculture topics focused on specific actions, we compiled, processed, and analyzed the content of thousands of public comments (N_total_ = 1,585) submitted to government agencies concerning aquaculture policy and development in marine waters. While news article content is informative, it does not necessarily reflect public concern and can contain a multitude of confounding topics and argument framings in a single piece that makes it extremely difficult to quantify sentiment at large spatiotemporal scales. Alternatively, public comments offer more direct language in support or opposition of a single topic in order to sway regulators in one direction or the other; this makes them ideal for analyzing sentiment polarity. Although reflecting only the most vocal interest groups, such comments reflect a form of real public (versus media) concern that can and does have an impact on policy and development of aquaculture.

Depending on the country, a period of public feedback is a required part of the regulatory process. We were able to access public submissions from two countries: The United States of America (USA) and New Zealand (NZ). Both developed nations, these two countries differ in size and emphasis on aquaculture and thus provide a glimpse into the similarities and differences around aquaculture sentiment, specifically in the marine environment. While submissions from only these countries were accessible and able to be formatted for analysis, they do represent central players in the offshore aquaculture industry. Equivalent documents from a developing country were not available, either due to governmental restrictions or no such comments appeared to exist.

For each developed country we were able to obtain two different types of public comments: one pertaining to general (i.e., national) policy change and one to more specific development proposals that have occurred within the last decade. For the USA, we analyzed comments pertaining to the 2011 National Marine Aquaculture Policy and the 2008 Regulatory Plan for Offshore Marine Aquaculture in the Gulf of Mexico (GOM); both organized by the National Oceanographic and Atmospheric Administration (NOAA). The policy comments related to the general framework to regulate development of all types of marine-based farming in the US, while the offshore plan specifically addressed establishing open-ocean marine finfish farming in the GOM. For NZ, we used comments from the 2011 NZ Parliament on an Aquaculture Legislation Bill (No. 3) relating to general permitting of marine aquaculture, and 2009 Marlborough District Council comments about expanding existing marine salmon farming in territorial waters of Marlborough Sounds [38]. Thus, comments from both countries separately touch upon broader policy issues and specific local development concerns. The associated dates correspond to the initial feedback period(s) of the policy or plan, not necessarily the actual year of implementation. The public comments for the GOM (2008–2015) span several years. More recent documents for all sources were not available, which is representative of the pace of new or updated aquaculture frameworks moderated at the local and/or federal level. All original USA (http://www.regulations.gov/) and NZ (http://www.marlborough.govt.nz/; http://www.parliament.nz/) comments are publicly available through their respective governmental websites.

All comment documents for each separate submission and associated topic were converted and processed for discrete sentiment analyses in order to determine the overall tone of the comments [39]. Starting with an ‘opinion lexicon’–a compilation of ca. 6,800 English words identified as positive or negative [40]–we expanded the list to include words and associations specific to aquaculture (n = 30; S2 Table). All PDF documents were read into R *v*3.2.0, converted to text files, and processed using the *tm* package [41]. Frequency of words and their associated sentiments were determined; generalized sentiment was calculated based on the summation of positive versus negative words and frequency of use (S3 Table). In order to compare word usage across source, topic (policy vs. development), and country (US vs. NZ) we standardized term frequency relative to the number of total submissions of the respective government source. Neutral words were not considered in the ultimate determination of sentiment polarity, but were tabulated for relative comparison of sentiment magnitude [42]. Words that appeared less than 10 times were excluded, except for the NZ plan documents (word frequency limit = 2) due to the comparatively small sample size (n = 36). In fact, due to small sample size for topic and country (n = 2), statistical differences (e.g., ANOVA) in sentiment were not informative. However, we found utility in descriptive comparisons nonetheless.

We explored specific perceived concerns and benefits around each aquaculture topic by identifying and comparing overall use of the top five most frequently used, least ambiguous negative and positive words in all four document sources. Comparing the most used positive and negative terms could result in a total of 20, and minimum of 5, ‘top’ words depending on the level of word usage similarity. In order to avoid ambiguous terminology, particularly regarding negative words, we tried to identify terms with clear context. For instance, ‘risk’ is a negative word, but does not offer detailed information of the specific risk factor and thus was not selected as a ‘top’ association; such language is however accounted for in the overall sentiment analysis previously described. In order to confer specific word sentiment associations (i.e., negative and positive) we randomly subsampled (n = 50) all documents and manually inspected (using the PDF search tool) the context of use.

We further investigated the primary *concerns* by calculating their strongest verbal correlations (r > 0.80) with other terms using the *tm* package [41]. We focused more closely on negative connotations to identify and clarify issues that need to be addressed at the management, scientific, and/or communication level. The correlation analysis also provided insight into context of the top negative words and aided in identifying perhaps less frequently used, but closely associated, terminologies. The context of the word was particularly important to support the initial sentiment classification (e.g., negative). Identical submissions (i.e., letter writing campaigns) from the same or different respondents were treated as single, multipage entries to reduce correlative biases.

Lastly, to provide an understanding of who was driving particular sentiment and opinions in the governmental comments we compared the proportions of submissions given eight demographic groupings. Groups were decided *a priori* based on known common aquaculture interest groups; they included *citizen*, *environmental*, *food*, *government*, *aquaculture*, *academic*, *fisheries*, and *other*. If an individual or group identified their association they were categorized into at least one of the groupings. Some people did not divulge their affiliation (38% of total submissions) and were thus excluded from this evaluation. The subset of identifiable affiliations was analyzed using a Chi-squared test to determine statistical difference between the proportions of grouped respondents within a topic. All analyses were performed in R *v*3.2.0 [43]. Analysis of the publically available third-party source comments were analyzed and reported anonymously and thus pose no threat to personal privacy or damage to the reputation of any individuals whose data were used.

## Results

### Headline trends

**Fig 1:**
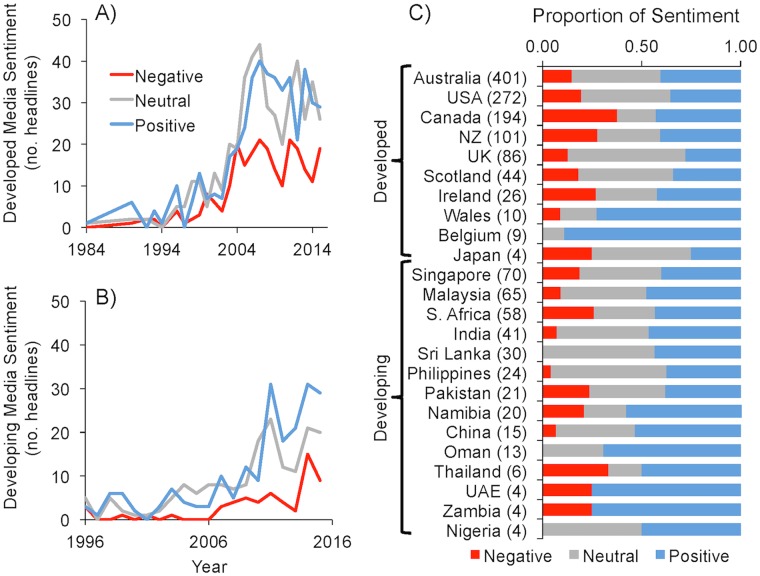
Newspaper ‘aquaculture’ media sentiment. Sentiment over time based on the frequency of newspaper headlines with negative (*red*), positive (*blue*), and neutral (*gray*) titles for **(A)** developed (n = 1,165) and **(B)** developing (n = 430) nations. Also depicted are the **(C)** proportional contributions of sentiment headlines relative to each developed and developing country, with the number of headlines from each country shown in parentheses. Only countries with more than 4 headlines are shown; 44 countries that contributed only 4.5% of the total headlines are not depicted, but can be found in the S1 Table. Headlines were compiled using the LexisNexis^®^ platform.

Internationally, newspaper ‘aquaculture’ headlines have increased and are overall more positive than negative. Of the developed nations, the total ‘aquaculture’ headlines (n = 1,165) spanning 1984 to 2015 and across 26 countries, have significantly (*p* < 0.001, F-stat = 49.5, R^2^_adj_ = 0.78) increased at a rate of 3.7 (SE ± 0.38) articles per year (Fig 1A). Although ‘aquaculture’ headlines did not appear in any of the 42 developing countries (n = 430) until 1996, the rate of increase thereafter averaged 2.8 (SE ± 1.1) articles per year (Fig 1B). All three sentiments showed an increase over time in both developed and developing nations, but positive (and neutral) headlines significantly (*p* < 0.001, F-stat = 14.1, R^2^_adj_ = 0.47) outpaced negative headlines (developed Δ_sentiment_ ± SE = 0.71 ± 0.15 headlines yr^-1^; developing = 0.95 ± 0.41 headlines yr^-1^).

Geographically nearly every country appeared to have published more positive (developed = 459; developing = 203) than negative (244; 58) ‘aquaculture’ headlines, with developing nations having proportionally more positive headlines than negative compared to the developed countries (Fig 1C). We found Australia, the USA, Canada, New Zealand, and United Kingdom made up the majority (83%) of developed nation headlines, while 10 countries contributed to over 80% of the developing nation headline trends (Fig 1C). The seemingly sweeping positive sentiment was somewhat surprising, so post-hoc we evaluated a subset of the data focused on headlines with ‘salmon’ included in the title–a topic typically plagued with negative associations [44]. While only 56 of the total 1,596 headlines referenced salmon directly, the sentiment was indeed negative (52% of headlines), with Canada driving that pattern by contributing 69% of the adverse salmon-based titles. Chile was the only developing nation with one negative salmon headline. These results highlight the diversity of media aquaculture reporting; specifically, salmon coverage from Canada does not represent the entirety of aquaculture media and does not appear to bias media at this level.

**Fig 2:**
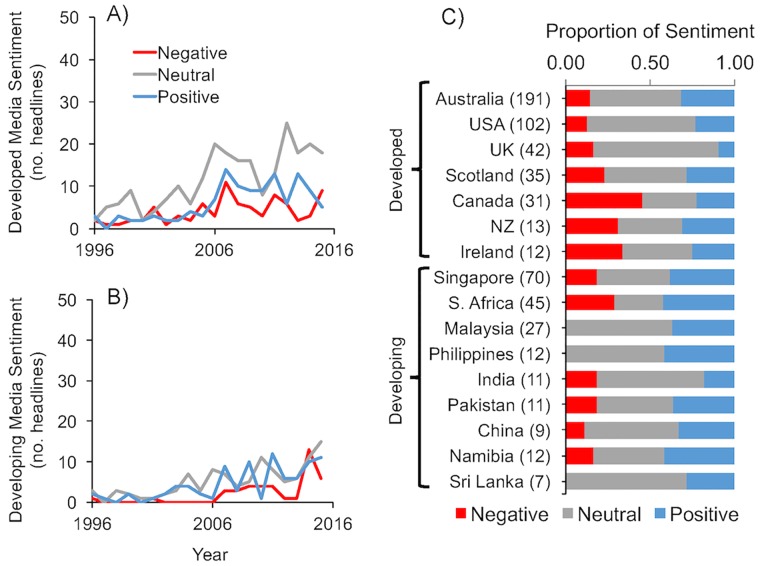
Newspaper ‘marine aquaculture’ media sentiment. Sentiment over time based on the frequency of newspaper headlines with negative (*red*), positive (*blue*), and neutral (*gray*) titles for **(A)** developed (n = 435) and **(B)** developing (n = 232) nations. Also depicted are the **(C)** proportional contributions of sentiment headlines relative to each developed and developing country, with the number of headlines from each country shown in parentheses. Only countries with more than 4 headlines are shown; 29 countries that contributed only 5.8% of the total headlines are not depicted, but can be found in the S1 Table. Headlines were compiled using the LexisNexis^®^ platform.

Although global ‘aquaculture’ media appears more positive, the polarity gets less definitive for ‘marine aquaculture’ (Fig 2). Similar to ‘aquaculture’, mariculture references for developed (n_articles_ = 435; Fig 2A) and developing (n_articles_ = 232; Fig 2B) nations have significantly (*p* < 0.001, F-stat = 38.9, R^2^_adj_ = 0.75) increased in newspapers over the period of 1996 to 2015 –although, less rapidly (developed ± SE = 1.7 ± 0.23 articles yr^-1^; developing = 1.4 ± 0.56 article yr^-1^). In addition, the magnitude of difference in the number of positive and negative titles was reduced for both developed (Δ_sentiment_ ± SE = 0.26 ± 0.12 headlines yr^-1^) and developing nations (0.14 ± 0.29 headlines yr^-1^) and the difference was no longer statistically significant. There were still slightly more positive than negative headlines, but more neutral headlines overall (Fig 2).

Developing nations still published fewer, but proportionally more positive than negative ‘marine aquaculture’ headlines compared to developed countries and top reporting countries differed slightly from general ‘aquaculture’ media coverage (Fig 2C). Australia and the USA accounted for almost half of all the headlines (293 out of 668). Singapore and South Africa more frequently published about marine-based aquaculture than New Zealand and the United Kingdom (Fig 2C).

**Fig 3:**
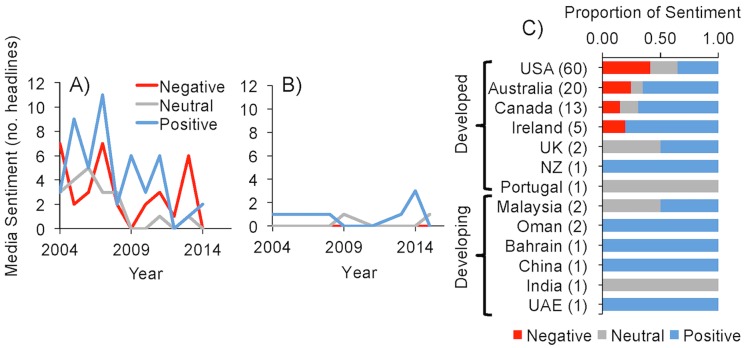
Newspaper ‘offshore aquaculture’ media sentiment. Sentiment over time based on the frequency of newspaper headlines with negative (*red*), positive (*blue*), and neutral (*gray*) titles for **(A)** developed (n = 102) and **(B)** developing (n = 8) nations. Also depicted are the **(C)** proportional contributions of sentiment headlines relative to each developed and developing country, with the number of headlines from each country shown in parentheses. All countries used in the analysis are depicted. Headlines were compiled using the LexisNexis^®^ platform.

Reference to ‘offshore aquaculture’ showed opposite headline trends to ‘aquaculture’ and ‘marine aquaculture’ and tended to be overall more negative. Headlines in the developed nations (n = 102) significantly (*p* < 0.001, F-stat = 12.66, R^2^_adj_ = 0.67) declined by an average of 1.3 (SE ± 0.33) each year (Fig 3A), while headlines from developing countries (n = 8) were close to zero (0.07 ± 0.082 headlines yr^-1^; Fig 3B). Although not statistically significant, developed nation sentiment was on average more negative (Δ_sentiment_ ± SE = - 0.25 ± 0.30 headlines yr^-1^) and the few developing headlines averaged positive to neutral over the years (0.04 ± 0.75 headlines yr^-1^). Offshore temporal patterns actually resemble the earlier, more divided time periods of ‘aquaculture’ and ‘marine aquaculture’ (Figs 1A and 1B and 2A and 2B).

At the country level, ‘offshore aquaculture’ headlines had the largest differences in number and overall sentiment. Notably, the USA accounted for over half of all offshore headlines and published more negative than positive titles (Fig 3C). Likely a partial artifact of sample size, not one developing nation published a negative ‘offshore aquaculture’ headline; the majority of which came from countries in the Middle East (Fig 3C).

### Public comment trends & opinions

Sentiment about specific marine aquaculture development plans tended to be more negative and general marine policy more positive, with similar sentiments reflected in the news headlines. Feeling towards the offshore development plan in the USA Gulf of Mexico was the most negative, with a greater than 10-fold discrepancy between the use of negative to positive terms (Table 1; freq. ratio = 11:1 negative to positive words per submission). The NZ marine expansion plan was the least definitive, with the smallest difference between the number of positive versus negative words, but less frequent use of negative terms (Table 1). Both the USA and NZ marine policy-based comments were more positive in the number and frequency of words used (Table 1). In fact, the USA policy and NZ documents had similar frequency ratios of negative to positive mentions (freq. ratio range = 0.5–0.6:1 negative to positive words per submission). Overall, sentiment from public comments appeared to generally parallel the topical and country-level sentiments of newspaper headlines–particularly for the USA and offshore aquaculture. More in-depth analyses are needed to fully disentangle the nested sentiments of aquaculture types and topics, but this more generalized approach emphasizes the importance of considering such confounding variables and shows promise in gauging public perception of aquaculture from the media.

**Table 1:** Total number and frequency (shown in parentheses) of identifiable negative and positive words from public comments.

Source	No. Negative Words and Freq.	No. Positive Words and Freq.	No. Neutral Words and Freq.
*USA Policy 2011* (N = 179)	52 (2,228)	**75 (3,462)**	1,043 (39,101)
*USA GOM Offshore Plan* (N = 1226)	**92 (28,107)**	59 (2,479)	997 (78,189)
*NZ Policy No*. *3 Policy 2011* (N = 144)	78 (3,915)	**116 (7,597)**	1,088 (104,612)
*NZ Mgt*. *Plan* (N = 36)	81 (393)	**91 (648)**	1,089 (10,116)

Words come from the United States of America (USA) and New Zealand (NZ) submitted public comments concerning the four marine and offshore aquaculture source topics; sample sizes (N) correspond to number of submissions. Polarity of sentiment was determined based on the perverseness (number and frequency) of negative and positive words. Neutral words are provided for relative reference of sentiment magnitude. The dominant sentiment(s) are **bolded**.

**Fig 4:**
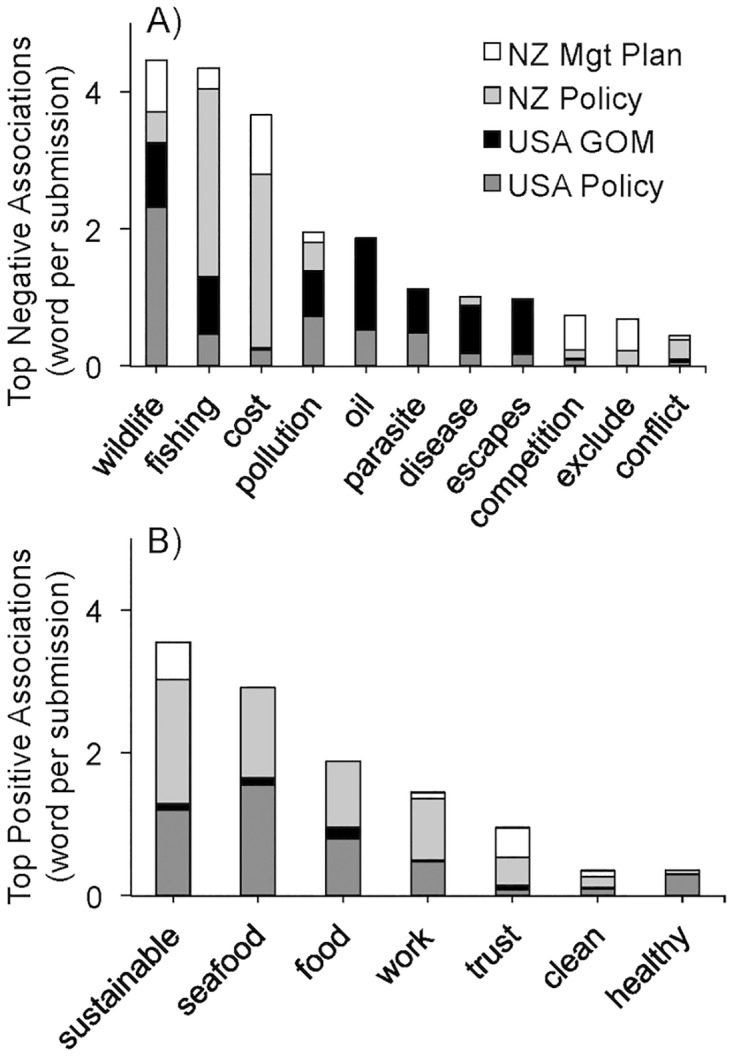
The most frequently (word per submission) used negative (A) and positive (B) word associations from government elicited public comments. Depicted frequencies from 2011 USA Marine Aquaculture Policy (*dark gray*), 2008 USA Offshore Aquaculture Gulf of Mexico (GOM) regulatory plan (*black*), 2011 NZ Policy Legislation Bill (No. 3) for marine farming (*light gray*), and the 2009 NZ expansion plan for salmon farming (*white*) in Marlborough marine waters.

Across the two countries, negative sentiment was more diverse than positive opinions, with USA documents tending to focus on negative environmental concerns, NZ comments on monetary costs, and both identifying impacts on fishing as a major issue. A total of 11 words were determined as the most frequently used negative terms, while only 7 positive words differed across the four aquaculture sources. The USA negative comments tended to be linked to the environment, with ‘wildlife’ (which captured the terms ‘wild’ or 'wildlife’) and ‘oil’ as most commonly referenced negative associations (Fig 4A). Alternatively, NZ negative terms tended to revolve around monetary–with some environmental–concerns, either regarding ‘cost’ or ‘competition/conflict’ (Fig 4A). Other than ‘wildlife’ and ‘cost’, only ‘fishing’ and ‘pollution’ were mentioned at relatively higher frequency in all four document sources. On the positive side, ‘sustainable’ and ‘seafood/food’ were the most commonly used terms, but only ‘sustainable’, ‘work’, ‘trust’ and ‘clean’ were referenced in all four cases (Fig 4B). Manual evaluation of ‘wildlife’, ‘fishing,’ and ‘sustainable’ supported the sentiment classifications. The correlation analysis also provides additional support to the sentiment assignments.

Negative word correlation analysis revealed general groupings of concerns that are not mutually exclusive, as well as potential influence of local environmental disasters (e.g., oil spills) unrelated to aquaculture. In the GOM submissions ‘oil’ correlated nearly 100% with ‘escapes’, ‘parasite’, disease’, ‘pollution’, ‘fishing’, and ‘spill’ (i.e., 2010 Deep Horizon oil spill). The same perceived issues (excluding ‘oil’) in the USA policy documents were also highly correlated (r = 0.99), but more prominently linked to terms associated with salmon (i.e., *mykiss*, salmonid, chum, pink). For the NZ documents, the primary correlative (r = 0.93–0.99) environmental concerns for ‘wildlife’ were more general, relating to potential impacts on ‘sanctuaries’ and ‘biodiversity.’ More specific ‘costs’ correlated with (r = 0.87–0.95) ‘fishing’, ‘ecotourism’, and ‘individuals’ in expansion plan documents and ‘assessment’ and ‘owners’ in policy related submissions. Note, the frequency of use of the top five terms was not equivalent across source topics (Fig 4) and thus only the major (r > 0.80) correlations are discussed.

### Public comment demographics

A significantly higher proportion of citizens and environmentalists contributed to specific marine aquaculture expansion-based public comments, which tended to be more negative in sentiment (Table 2). For the GOM, a total of 75% of the comments (*X*^*2*^ = 122.07, *p* < 0.001) were submitted by people identifying themselves as a citizen or affiliated with an environmental group (Table 2). Similarly, the NZ salmon expansion plan was almost entirely (80%) commented on by citizens and environmental groups (*X*^*2*^ = 15.75, *p* < 0.001). Alternatively, both policy-based topics had a more evenly distributed number of demographic groups that submitted comments; importantly, people associated with the aquaculture industry. Notably, academics contributed more in the US than in NZ, and fishers consistently showed little input across countries and topics (Table 2).

**Table 2:** Public comment groups and associated percentages of the identifiable respondent contributions to each marine aquaculture topic.

Topic	N	*Citizen*	*Environmental*	*Food*	*Government*	*Aquaculture*	*Academic*	*Fisheries*	*Other*
**USA Policy 2011**	164 (179)	15%	16%	5%	13%	16%	15%	5%	16%
**USA GOM Offshore Plan 2008**	290 (1226)	46%	29%	6%	8%	3%	2%	1%	9%
**NZ No. 3 Policy 2011**	123 (144)	29%	9%	2%	21%	22%	0%	4%	13%
**NZ Mgt. Plan 2009**	32 (36)	40%	40%	0%	3%	9%	0%	3%	6%

Total number (N) of comments is depicted in parentheses, while the number of categorized individuals is shown outside the parentheses. *Gray* shading represents significantly (*X*^*2*^; *p* < 0.05) different (i.e., larger) proportions compared to the *white* cells within each topic.

## Discussion

We explored the cross-national sentiment patterns and specific underlying perceptions of aquaculture using media and public comments. Using newspaper headlines we found a tendency towards growing positive sentiment of aquaculture across time and geographic regions, but lack of knowledge and unfamiliarity with differing types of aquaculture may be influencing public trends. Notably, developed national media was proportionally more negative than developing countries. Exploring the particularly negative patterns of offshore aquaculture–predominantly in the USA–revealed a potential combination of drivers, including previous environmental disturbances (e.g., oil spills) not directly related to aquaculture. In addition, the common negative rhetoric of the very general or repeated terms being used in the public comments highlights awareness, but non-targeted concerns of marine farming. Lastly, we found the majority of negative sentiment appeared to be driven by concerned citizens and environmental groups, with very few fishers voicing their opinions.

Newspaper headlines appear to be increasing and becoming more positive towards the general concept of aquaculture, suggesting some level of acceptance or positive feeling of aquatic farming around the world. With news media identified as the primary source for information from the food sector [24–27], perhaps our results represent a shift in perception and understanding of aquaculture. Indeed, the few past survey and interview perception studies have a common theme of people showing no strong aversion to farmed seafood [15,25,45–49]. Yet, most of these studies also point to a general lack of knowledge around aquaculture. In several cases, when respondents had relatively little knowledge of aquaculture and were then provided some level of neutral information, they showed preferential favoritism of wild over farmed fish [17,45,50]. Such changes in attitudes suggest negative news may have a disproportionate effect on public perception. Indeed, risk perception and how the news frames risk appears to play a major role in avoidance of certain foods and associated technology (e.g., GMOs) [24,51]. In order to improve understanding of sustainable aquaculture–especially countries with more negative press, such as the developed nations of Canada, Australia, and the USA–new and innovative forms of information streams need to be established. For example, government and industry partnering with ocean literacy groups, as the EU Commission has just done to help develop and promote Blue Growth.

Developed nation headlines were overall more negative than developing countries–particularly around offshore practices–suggesting two slightly different viewpoints on aquaculture. Other than knowledge, other studies report an array of drivers influencing perception of developed countries, including demographics [15,46,52], trust of the industry and government [20], health and food safety [15,49], environment [53], and local context [52,54]. From the public comments we evaluated, we found development plans (especially offshore), not policy, garnered the most negative criticism from citizens and environmentalists. This suggest a level of ‘not in my backyard’ (NIMBY) perspective, where local proximity to marine aquaculture development appears to increase negative impressions [55,56]. Although NIMBYism should not diminish the importance of addressing such perceived or real concerns [23,57–59], it does create economic and seafood transparency tradeoffs with most of developed nations’ seafood not being sourced from their respective countries [3]. Although we were unable to obtain public comments from a developing country, the very few perception studies from developing regions (Chile, Ghana, Kenya, Philippines, and Tanzania) show a general acknowledgement and positive association with the economic importance of aquaculture, while taste and health concerns contributed to some negative connotations [60–64]. It is also important to note that while offshore aquaculture tends to be associated in a more developed nation context, several developing countries–particularly from the Middle East–had enough interest to publish positive news articles in English.

The magnitude of negative perception of aquaculture could be dependent on recent and local environmental catastrophes. The GOM Deep Horizon oil spill in 2010 left a lasting impression on the local coastal communities in the USA, which is overtly apparent in the offshore aquaculture public comments. The issues of uncertainty around the magnitude of impact the oil spill had on the Gulf appeared to manifest as negative sentiment around offshore development in general–which is apparent in the highly correlated, yet unrelated terms of ‘oil’, ‘parasite’, ‘escape’, etc. Indeed, Murray and D’Anna (2015) found in B.C., Canada that environmental issues and personal experience tend to be interpreted together, while economic implications of aquaculture are comprehended somewhat independently. Certainly, the NZ comments tend to revolve around financial consequences–good and bad–and more general concerns of marine farming affecting protected habitat and species. The absence of a recent environmental crisis in that region may explain some of the positive and negative discrepancies in NZ and the USA sentiments, respectively. As the spill event fades from the minds of the GOM residents and the media spotlight, perhaps negative perceptions of offshore development will also diminish [65].

Assessing the comments in more detail revealed the most pervasive concern was impacts on ‘wildlife,’ but the specific issues followed a generalized rhetoric. Both countries frequently cited wildlife impacts as worrisome, but the basis and specificity for concern varied. Specifically, NZ comments showed general concern over protected or sensitive areas or species, while the US submissions grouped many specific negative issues all together. Expressly, escapes, parasites, disease, and/or pollution *can* occur under poorly managed aquaculture facilities [10,22,66], but not necessarily all together, nor are they inevitable if management is good. In fact, the nearly 100% correlation between the ‘usual suspects’ of aquaculture problems in the public arena reveals the level of uncertainty around the actual versus perceived threats to the local ecosystems–particularly in an offshore perspective. Moreover, a portion of the generalized negative referencing comes from ‘letter writing campaigns’, where the exact same grievance(s) are submitted separately by hundreds of people. Interestingly, offshore applications may actually mitigate some of these perceived correlated impacts, such as pollution and disease [6], and yet despite this potential of increased sustainability we found a more negative sentiment for offshore aquaculture. This suggests that the public and media may be reflecting a lack of knowledge about offshore aquaculture, along with scientific uncertainty around marine aquaculture expansion in general. Actual measures of risk and impact are scarce for the burgeoning offshore aquaculture sector [22]. Better development and communication of the distinctions between the types of aquaculture may be an achievable first step to help clarify and identify the real public issues.

There also appears to be a discrepancy between actual fishers voicing their opinions and concern over impacts on fishing. A more qualitative evaluation of published scientific and gray literature on aquaculture stakeholder impacts identified fishers as a key concerned group in the EU [23]; yet, their presence is minimal in the government collected comments we assessed. It is unclear whether low percentages of fishers commenting on any and all of the aquaculture issues are the result of an inherently small population, simple disinterest, and/or perhaps a perception that their opinions will do little to change the outcome. However, even with only a small percentage of individuals self-identified as a fisher, concern for ‘fishing’ was one of the most frequently cited in the documents. Such discrepancies in public input need to be explored in order to fully understand and address the real versus perceived concerns of the interrelated markets of aquaculture and wild caught fisheries [67,68].

Our sentiment analyses of newspaper headlines and government public comments have several important limitations. Regarding the headlines, we only assessed English news titles. While we accounted for some of the bias by analyzing trends based on developed versus developing nations, the headlines are still skewed towards more English speaking regions and do not proportionally represent some of the largest aquaculture producing countries, such as China. Due to the scale and comparative offshore focus of our study, we did not evaluate the specific content of the news articles. However, selection of a subset of countries and running a more complex and rigorous analysis on how news sources frame the content of the articles could be beneficial in further identifying primary public concerns [16]; such an approach may be particularly applicable for developing nations. Indeed, there is a dearth of information regarding the perceptions of aquaculture in developing countries [63]. We were unable to access and/or find government collected public comments for a developing nation, and thus more publically voiced and explicit opinions relating to the various forms of aquaculture could not be gleaned. The opinions of the public comments also present some biases in that they reflect only the most vocal interest groups and may not capture the more variable perspectives of a country. Such aspects of perception could be explored in more detail, but the intent of this study was to reveal more general and larger scale sentiment patterns using new data sources and innovate approaches.

## Conclusions

This research provides a new comparative perspective and novel methodology to explore public perception of aquaculture in its many forms around the world. Different types (nearshore vs. offshore) and contexts (policy vs. development) of aquaculture have important implications for the understanding and management of public opinion. Overall, sentiment appears positive around general aquaculture practices and policy, yet the negative sentiment and indiscriminate concerns of groups towards offshore aquaculture development speaks to the misunderstanding of differing types of risks associated with the various forms of aquaculture. Whether or not individuals, communities, and governments are supportive of aquaculture, accurate representation and communication of real risks versus misconceptions is critical to constructive and informed dialogue. As the aquaculture industry continues to expand and prepares to use more of the ocean for production [3,7,8], such clarity is all the more important to ensure the growth of the sector happens in the most sustainable and beneficial way [69].

## Supporting Information

S1 Table Complete list of newspaper headlines. Included in the table are the associated types of aquaculture (‘aquaculture’, ‘marine aquaculture’, ‘offshore aquaculture’), region of origin, year, and the sentiment values used in the linear analyses. *Positive* sounding headlines were given a value of 1, *negative* headlines a -1, and *neutral* headlines a 0.
(DOCX)

S2 Table List of added negative and positive terms specific to aquaculture. (DOCX)

S3 Table Complete list of words from government public comments. Included in the table are the words extracted from all government documents, the total frequency of use, and the ‘opinion lexicon’ assigned sentiment associations (positive, negative, and neutral). Documents include the 2011 USA Marine Aquaculture Policy, 2008 USA Offshore Aquaculture Gulf of Mexico (GOM) regulatory plan, 2011 NZ Policy Legislation Bill (No. 3) for marine farming, and the 2009 NZ expansion plan for salmon farming in Marlborough marine waters.
(DOCX)
