# Supplementary material for: Public Perceptions of Aquaculture: Evaluating Spatiotemporal Patterns of Sentiment around the World
Source: PLoS One. 2017 Jan 3;12(1):e0169281. doi: 10.1371/journal.pone.0169281 (PMC5207524; doi:10.1371/journal.pone.0169281)
Supplement: S1 Table — Included in the table are the associated types of aquaculture (‘aquaculture’, ‘marine aquaculture’, ‘offshore aquaculture’), region of origin, year, and the sentiment values used in the linear analyses. Positive sounding headlines were given a value of 1, negative headlines a -1, and neutral headlines a 0. (DOCX) [file pone.0169281.s001.docx]

**S1 Table Complete list of newspaper headlines.** Included in the table are the associated types of aquaculture (‘aquaculture’, ‘marine aquaculture’, ‘offshore aquaculture’), region of origin, year, and finally the sentiment values used in the linear analyses. *Positive* sounding headlines were given a value of 1, *negative* headlines a -1, and *neutral* headlines a 0.

| **Headline** | **Type** | **Region** | **Year** | **Sentiment** |
| --- | --- | --- | --- | --- |
| `Tap aquaculture industry potential' | Aquaculture | Malaysia | 2015 | 1 |
| El Nino aquaculture aid to focus on worst-hit fishing communities | Aquaculture | Philippines | 2015 | 1 |
| SIPA 2015: Three development agreements inked | Aquaculture | Algeria | 2015 | 0 |
| Industry warned not to ignore climate change | Aquaculture | New Zealand | 2015 | -1 |
| Fisheries to Rethink Freshwater Aquaculture | Aquaculture | Namibia | 2015 | 1 |
| N$12 Million for Aquaculture | Aquaculture | Namibia | 2015 | 1 |
| Kentucky: KDA aquaculture specialist named to national task force | Aquaculture | USA | 2015 | 1 |
| Fisheries Launches Plan to Bridge Inequality | Aquaculture | Namibia | 2015 | 1 |
| DNV GL Announces Acquisition of Noomas Certification | Aquaculture | India | 2015 | 0 |
| Project could give boost to marine aquaculture | Aquaculture | USA | 2015 | 1 |
| Aqua Culture Master Plan Unveiled | Aquaculture | Namibia | 2015 | 1 |
| AQUAEXCEL2020: AQUAculture infrastructures for EXCELlence in European fish research towards 2020 | Aquaculture | France | 2015 | 1 |
| OCEANFISH: Open Ocean Fish farms | Aquaculture | Israel | 2015 | 0 |
| Aquaculture the Way to Go for Namibia - Esau | Aquaculture | Namibia | 2015 | 1 |
| WA aquaculture earns strategic support status | Aquaculture | Australia | 2015 | 1 |
| Report on aquaculture calls for transparency | Aquaculture | Canada | 2015 | -1 |
| Aquaculture report falls short: critics; But industry praises Senate report, sees need for overhaul | Aquaculture | Canada | 2015 | -1 |
| Petuna nets world status | Aquaculture | Australia | 2015 | 0 |
| Aquaculture firm lands a double prize catch | Aquaculture | Australia | 2015 | 1 |
| Aquaculture industry needs strict regulation | Aquaculture | Australia | 2015 | -1 |
| Fishtimator: Continual Acoustic Based Multifunctional Cage Mounted Fish estimator Deigned To Reduce Feed Waste, Fish Mortality, and Predator and Fish Escape Control | Aquaculture | Belgium | 2015 | 1 |
| Aquaculture's Explosive Growth Means Farmers Are About to Run Out of Fish Food | Aquaculture | Nepal | 2015 | -1 |
| Aquaculture Industry Still in Its Infancy | Aquaculture | Namibia | 2015 | 0 |
| Increasing production from inland aquaculture in Papua New Guinea New Guinea for food and income security | Aquaculture | Australia | 2015 | 1 |
| Shellfish Aquaculture Technology Research Center | Aquaculture | Japan | 2015 | 0 |
| Project for Strengthening Research and Development on Fisheries and Aquaculture | Aquaculture | Lao | 2015 | 1 |
| EASY: ECO-INNOVATE-AQUACULTURE-SYSTEM | Aquaculture | Belgium | 2015 | 1 |
| SPACETASTE: Towards off flavour free finfish aquaculture | Aquaculture | Switzerland | 2015 | -1 |
| Aquaculture firm opens plant | Aquaculture | Australia | 2015 | 1 |
| From farm to palate | Aquaculture | China | 2015 | 1 |
| Sea change looming; Aquaculture | Aquaculture | Australia | 2015 | -1 |
| ECOFISH: Researches on the potential conversion of conventional fish farms into organic by establishing a model and good practice guide | Aquaculture | Belgium | 2015 | 1 |
| Solwezi in Aquaculture Intervention Move | Aquaculture | Zambia | 2015 | -1 |
| Paving the way for aquaculture; Fish farming: New area for growth | Aquaculture | Australia | 2015 | 1 |
| Aquaculture saviour for Bluff and island Ministry of Primary Industries report | Aquaculture | New Zealand | 2015 | 1 |
| Namibia, Congo Discuss Aquaculture Improvement | Aquaculture | Namibia | 2015 | 1 |
| Aquaculture Can Tackle Poverty and Unemployment | Aquaculture | Namibia | 2015 | 1 |
| Strategic drive to boost sustainable aquaculture | Aquaculture | Ireland | 2015 | 1 |
| Aquaculture firm appealing protection order | Aquaculture | Canada | 2015 | -1 |
| Fison Seeks Establishment of Fisheries Commission | Aquaculture | Nigeria | 2015 | 0 |
| Global Aquaculture Alliance and Preferred Freezer Services partnership empowers UMass Dartmouth students to build global aquaculture facilities database | Aquaculture | USA | 2015 | 1 |
| Huge Potential Seen for Aquaculture in Krishna | Aquaculture | India | 2015 | 1 |
| Zone concern for fishers | Aquaculture | USA | 2015 | -1 |
| Experts to look at why EU aquaculture lacks growth | Aquaculture | Scotland | 2015 | 1 |
| Louisiana: Aquaculture offers economic opportunities worldwide | Aquaculture | USA | 2015 | 1 |
| Super prawns breakthrough | Aquaculture | Australia | 2015 | 1 |
| Aquaculture projects to generate flow-on effect | Aquaculture | Australia | 2015 | 1 |
| M.A.P. INSIGHTS; OUR GOLD COAST | Aquaculture | Indonesia | 2015 | 1 |
| Research and Markets: Aquaculture Market in China 2015-2019 | Aquaculture | India | 2015 | 0 |
| Triton Group pledges $54m investment in Nigeria's aquaculture | Aquaculture | Nigeria | 2015 | 1 |
| New zones proposed for Franklin Harbour | Aquaculture | Australia | 2015 | 0 |
| Aquaculture a Headache - Esau | Aquaculture | Namibia | 2015 | -1 |
| Virginia's aquaculture oyster, clam harvests set records | Aquaculture | USA | 2015 | 1 |
| Contributions of aquaculture highlighted | Aquaculture | Pakistan | 2015 | 1 |
| Diversification of smallholder coastal aquaculture in Indonesia | Aquaculture | Indonesia | 2015 | 1 |
| Cooke Aquaculture division nets 'four star' certification | Aquaculture | Canada | 2015 | 1 |
| Ted Maney is panelist for webinar on permitting offshore aquaculture | Aquaculture | USA | 2015 | 1 |
| Upset residents want more say in aquaculture vetting | Aquaculture | Australia | 2015 | -1 |
| Six nations join FAO for sustainable aquaculture | Aquaculture | Thailand | 2015 | 1 |
| North-West Earmarked for Aquaculture Value Chain | Aquaculture | Zambia | 2015 | 1 |
| Use science to guide competing fisheries | Aquaculture | Canada | 2015 | 0 |
| Aquaculture focus | Aquaculture | Philippines | 2015 | 0 |
| New Facilities to Boost Aquaculture Production | Aquaculture | Namibia | 2015 | 1 |
| New Aquaculture Licensing in British Columbia to Improve Business Planning and Encourage Investments in Sustainable Practices | Aquaculture | Canada | 2015 | 1 |
| CityU course seeks to modernise aquaculture | Aquaculture | Scotland | 2015 | 1 |
| Aquaculture outreach at Iowa State evolves with industry | Aquaculture | USA | 2015 | 1 |
| NAQUA presented with 'Best Aquaculture Practices' certificate | Aquaculture | USA | 2015 | 1 |
| Mixed reception for aquaculture leases | Aquaculture | Wales | 2015 | -1 |
| Fostering Economic Growth by Supporting Aquaculture | Aquaculture | Canada | 2015 | 1 |
| USPTO grants trade mark INTELLILUM" to Gregory A.M. Brown (INDIVIDUAL) (NEVADA)" | Aquaculture | USA | 2015 | 0 |
| Army Corps opens comment period for aquaculture; Shellfish farming needs corps approval before leases begin | Aquaculture | USA | 2015 | 0 |
| CARGILL FEEDING ON FISH FARMING | Aquaculture | USA | 2015 | 0 |
| DEAKIN students and disadvantaged communities will | Aquaculture | Canada | 2015 | 0 |
| PRESS RELEASE - CANADIAN COUNCIL OF FISHERIES AND AQUACULTURE MINISTERS CONTINUE TO WORK TOGETHER TO PROTECT CANADA'S FISHERIES | Aquaculture | Canada | 2015 | -1 |
| SQU conducts special fish feast to boost aquaculture | Aquaculture | Oman | 2015 | 1 |
| Marine Aquaculture Development in the Sultanate of Oman/ Symposium | Aquaculture | Oman | 2014 | 1 |
| Fine imposed on Cooke Aquaculture subsidiary | Aquaculture | Canada | 2014 | -1 |
| Rhodes boffins are fully behind Africa aquaculture plan | Aquaculture | South Africa | 2014 | 1 |
| Rhodes fish experts link up with African varsities | Aquaculture | South Africa | 2014 | 0 |
| Social licence vital for aquaculture | Aquaculture | New Zealand | 2014 | 1 |
| Appointments to the Climate and Aquaculture Task Forces by the Marine Fisheries Advisory Committee | Aquaculture | USA | 2014 | 1 |
| Queensland to reap benefits of aquaculture boom | Aquaculture | Canada | 2014 | 1 |
| NMIT to launch aquaculture degree | Aquaculture | New Zealand | 2014 | 1 |
| Aquaculture courses a first | Aquaculture | New Zealand | 2014 | 0 |
| AUSTRALIA'S aquaculture sector needs real capital"" | Aquaculture | Australia | 2014 | 0 |
| Portland biotech firm alleges patent infringements by Cooke Aquaculture | Aquaculture | USA | 2014 | -1 |
| Maclean hears fish farming feeds more than beef does | Aquaculture | Australia | 2014 | 1 |
| Fledgling fisheries lure China; Brad Thompson; WA aquaculture interest | Aquaculture | Australia | 2014 | 1 |
| Fisheries of the Caribbean, Gulf, and South Atlantic; Aquaculture | Aquaculture | USA | 2014 | 0 |
| Firm views held on sea farm effect | Aquaculture | New Zealand | 2014 | -1 |
| Ailing Fish Farmer Shows Gaps in Greek Bankruptcy Regime | Aquaculture | Greece | 2014 | 0 |
| Future of aquaculture 'oversold' | Aquaculture | New Zealand | 2014 | -1 |
| PROMOTING FISHING OF MARINE AND INLAND FISH | Aquaculture | India | 2014 | 0 |
| Demand for inland water fish on the rise - Rajitha | Aquaculture | Sri Lanka | 2014 | 0 |
| $20 million federal grant to fund Maine aquaculture research network | Aquaculture | USA | 2014 | 1 |
| Rapid aquaculture expansion alarms residents on Bagaduce River | Aquaculture | USA | 2014 | -1 |
| Aquaculture farms to revive plunging fish stocks | Aquaculture | United Arab Emirates | 2014 | 1 |
| Delaware: DNREC announces final regulation for shellfish aquaculture in the Inland Bays | Aquaculture | USA | 2014 | 0 |
| Catalyzing women participation in aquaculture | Aquaculture | India | 2014 | 1 |
| Aquaculture strategy launched | Aquaculture | Malta | 2014 | 1 |
| 'Mahinda Rajapaksa Global Aquaculture Fund' endorsed | Aquaculture | Sri Lanka | 2014 | 1 |
| WAUCHOPE company Aquasonic Pty Ltd, the largest aquaculture | Aquaculture | Australia | 2014 | 0 |
| NEW POLICY TO BE BROUGHT FOR FAST GROWTH OF FISHERIES SECTOR AND FISHERMEN WELFARE, AGRICULTURE MINISTER SINGH INAUGURATES 33RD SESSION OF ASIA PACIFIC FISHERIES COMMISSION | Aquaculture | India | 2014 | 0 |
| New aquaculture centre aims to boost industry | Aquaculture | Scotland | 2014 | 1 |
| Comox event will also aid North Island College | Aquaculture | Canada | 2014 | 0 |
| Back fish farming, urges sector expert | Aquaculture | Australia | 2014 | 1 |
| Farmed fish must double | Aquaculture | Australia | 2014 | 1 |
| EP leads aquaculture boom | Aquaculture | Australia | 2014 | 1 |
| Computer planning for aquaculture growth | Aquaculture | Australia | 2014 | 1 |
| Seafood farming valued at $1.3bn | Aquaculture | Australia | 2014 | 1 |
| RGCA'S LAB IS INDIA'S 1ST NABL ACCREDITED AQUACULTURE PATHOLOGY LAB | Aquaculture | India | 2014 | 0 |
| MPEDA aquaculture path lab gets NABL accreditation | Aquaculture | India | 2014 | 1 |
| Henties Bay to Harnass Mariculture and Aquaculture | Aquaculture | Namibia | 2014 | 1 |
| Senura Aqua invests US$ 2m for stage II | Aquaculture | Sri Lanka | 2014 | 0 |
| Aquaculture online is meeting critical needs | Aquaculture | Wales | 2014 | 1 |
| A new act can't fix aquaculture | Aquaculture | Canada | 2014 | -1 |
| LIFE-AQUASEF - ECO-EFFICIENT TECHNOLOGIES DEVELOPMENT FOR ENVIRONMENTAL IMPROVEMENT OF AQUACULTURE, AQUASEF | Aquaculture | Spain | 2014 | 1 |
| Aquarium one of the world's best | Aquaculture | Australia | 2014 | 0 |
| Canada needs to lead in aquaculture | Aquaculture | Canada | 2014 | 1 |
| Aquaculture study may miss small fry | Aquaculture | Australia | 2014 | -1 |
| Research and Markets: Aquaculture Market Report Global Forecast & Opportunities 2013 - 2019 | Aquaculture | United Kingdom | 2014 | 0 |
| World showcase for our aquaculture | Aquaculture | Australia | 2014 | 1 |
| Agri sector on board to grant MFN status to India: Bosan | Aquaculture | Pakistan | 2014 | 0 |
| Improving community-based aquaculture in Fiji, Kiribati, Samoa and Vanuatu | Aquaculture | Samoa | 2014 | 1 |
| Lack of latest tech hurting agriculture productivity: Bosan | Aquaculture | Pakistan | 2014 | 0 |
| Industrial wastage threat to aquaculture | Aquaculture | Pakistan | 2014 | -1 |
| Fish population being threatened due to industrial effluents: Punjab governor | Aquaculture | Pakistan | 2014 | -1 |
| Aquaculture may help ward off food insecurity | Aquaculture | Pakistan | 2014 | 1 |
| 'State Yet to Exploit Aquaculture Prospect' | Aquaculture | India | 2014 | 1 |
| Senators visit Tofino's aquaculture facility | Aquaculture | Canada | 2014 | 0 |
| Aquaculture vital for meeting growing demand for fish | Aquaculture | India | 2014 | 1 |
| Aquaculture park goes to public consultation | Aquaculture | Australia | 2014 | 0 |
| CCC workshop on aquaculture development | Aquaculture | Sri Lanka | 2014 | 0 |
| Aquaculture workshop | Aquaculture | Australia | 2014 | 0 |
| $2000 up for grabs for young aquaculture leaders | Aquaculture | Australia | 2014 | 0 |
| Research and Markets: India Commercial Aquafeed Market Outlook 2018 Provides Market Forecast of 2014 & 2018 for Aquaculture Production by the Type of Finfish | Aquaculture | India | 2014 | 0 |
| Acquaculture Fish Harvest Low | Aquaculture | Namibia | 2014 | -1 |
| Delaware: REMINDER: DNREC's Division of Fish and Wildlife to hold second public workshop Feb. 26 on Inland Bays shellfish aquaculture regulations | Aquaculture | USA | 2014 | 0 |
| Aquaculture interests run deep and wide; Start-up costs are major concern; meeting set Wednesday, Feb. 26 | Aquaculture | USA | 2014 | 1 |
| BOI approves aquaculture project in Chilaw | Aquaculture | Sri Lanka | 2014 | 1 |
| Fisheries minister offers up $54M; Cash will be used to continue research and improve regulatory management in the industry | Aquaculture | Canada | 2014 | 1 |
| True North Salmon aims for fourth star | Aquaculture | Canada | 2014 | 1 |
| Community-based Climate Resilient Fisheries and Aquaculture Development in Bangladesh | Aquaculture | Bangladesh | 2014 | 1 |
| Coveney admits frustrations over aquaculture | Aquaculture | Ireland | 2014 | -1 |
| Federal government supports B.C. salmon; Aquaculture moratorium continues; investments made in fishery: minister | Aquaculture | Canada | 2014 | 1 |
| PHL to export milkfish, other aquaculture products to UAE | Aquaculture | Philippines | 2014 | 1 |
| Farms to Produce 60 Percent of Global Food Fish By 2030 | Aquaculture | Lagos | 2014 | 1 |
| Investors eye Gulf aquaculture boom | Aquaculture | United Arab Emirates | 2014 | 1 |
| Oman, Saudi Arabia ride new wave of investment in aquaculture industry | Aquaculture | Oman | 2014 | 1 |
| WAAPP - Nigeria Hosts Aquaculture Experts | Aquaculture | Nigeria | 2014 | 0 |
| Asia to meet global seafood demand | Aquaculture | Pakistan | 2014 | 1 |
| DNREC sets workshop on Inland Bays shellfish aquaculture regs for Jan. 30 | Aquaculture | USA | 2014 | 0 |
| Washington DNREC's Division of Fish and Wildlife to hold public workshop January 30 on Inland Bays shellfish aquaculture regulations | Aquaculture | USA | 2014 | 0 |
| Research and Markets: Global Aquaculture Market Report 2013 - 2019: Carp, Molluscs, Crustaceans, Salmon, Trout and Other Fishes | Aquaculture | India | 2014 | 0 |
| Republic Poly starts research centre for aquaculture; Facility will study ways to increase food fish production, boost manpower training | Aquaculture | Sigapore | 2014 | 1 |
| Aquaculture regs to be issued in July; Delaware is last Atlantic Coast state to establish fish farming rules | Aquaculture | USA | 2014 | 0 |
| Don't believe aquaculture hype | Aquaculture | Canada | 2014 | 0 |
| Canada needs a national aquaculture strategy | Aquaculture | Canada | 2013 | -1 |
| Workshop on aquaculture development | Aquaculture | Sri Lanka | 2013 | 1 |
| Aaa organises workshop on aquaculture development | Aquaculture | Sri Lanka | 2013 | 1 |
| Professor to study green aquaculture; Oceanographer Jon Grant has been awarded $1.6-million to investigate the environmental effects of a booming Maritime business | Aquaculture | Canada | 2013 | 1 |
| Netting $800K for sustainable fish farm research | Aquaculture | Canada | 2013 | 1 |
| Hawaii: Commercial aquaculture could increase | Aquaculture | USA | 2013 | 1 |
| Cooke Aquaculture invests in research | Aquaculture | Canada | 2013 | 1 |
| Local fisheries on world stage | Aquaculture | Australia | 2013 | 0 |
| Alltech Announces New Technologies At APA13 To Support Aquaculture Producers In Asia-Pacific For Immediate Release | Aquaculture | China | 2013 | 1 |
| Aquaculture Association Decries Importation of Fish | Aquaculture | Ghana | 2013 | 0 |
| SA's big aquaculture catch | Aquaculture | Australia | 2013 | 1 |
| Washington: Aquaculture market is expected to reach $195 billion by 2019 | Aquaculture | USA | 2013 | 1 |
| Tuna leads aquaculture growth | Aquaculture | Australia | 2013 | 1 |
| Legal war guts aquaculture plan | Aquaculture | New Zealand | 2013 | -1 |
| Panel focuses on sustainable aquaculture | Aquaculture | USA | 2013 | 1 |
| Early taste of aquaculture | Aquaculture | New Zealand | 2013 | 1 |
| Coordinated Aquaculture Needed to Meet Global Demand, Says FAO | Aquaculture | Lagos | 2013 | 1 |
| Fish farmer hits cutbacks | Aquaculture | Australia | 2013 | 0 |
| Embattled seafood farmers will lobby for reform | Aquaculture | Australia | 2013 | 0 |
| Governor re-appoints professor, alumnus to state advisory board | Aquaculture | USA | 2013 | 0 |
| Global fish output eclipses red meat; Open-ocean farm growth predicted | Aquaculture | South Africa | 2013 | 1 |
| Fish farming output eclipses beef | Aquaculture | South Africa | 2013 | 1 |
| Scholarships to boost fish farming | Aquaculture | New Zealand | 2013 | 1 |
| Oyster harvesting to return to Inland Bays; Bill allows leasing of waters for shellfish | Aquaculture | USA | 2013 | 1 |
| Delaware: Governor Signs Law Bringing Economic and Environmental Benefits of Aquaculture to Delaware | Aquaculture | USA | 2013 | 1 |
| Aquaculture can reduce food insecurity in Pakistan | Aquaculture | Pakistan | 2013 | 1 |
| BMMP delegation for Norway Aqua-nor int'l exhibition | Aquaculture | Sri Lanka | 2013 | 0 |
| Aquaculture centre gives teens new angles to explore | Aquaculture | Australia | 2013 | 1 |
| Economic Indicators; Seaweeds succumb to 'ice ice' disease | Aquaculture | Philippines | 2013 | -1 |
| Wakatu, Cawthron sign pact | Aquaculture | New Zealand | 2013 | 0 |
| Wary of government? Yes, but we need some level of trust | Aquaculture | USA | 2013 | 1 |
| AQUACULTURE : SECTOR COMMITS TO SUSTAINABLE DEVELOPMENT | Aquaculture | United Kingdom | 2013 | 1 |
| Fish farm rates target | Aquaculture | Australia | 2013 | 1 |
| JEFI inks three MoUs on aquaculture projects | Aquaculture | Malaysia | 2013 | 0 |
| Fish farms' chips roll in $7m for Macquarie Harbour | Aquaculture | Australia | 2013 | 1 |
| Ministry of Agriculture: MOA, FAO Jointly Hold a Training Course on Aqua-Feed and Aqua-Seed | Aquaculture | China | 2013 | 0 |
| Sri Lanka in aquaculture drive with Viet Namnam | Aquaculture | Viet Nam | 2013 | 1 |
| FISHERIES : OPEN METHOD OF COORDINATION TO BOOST AQUACULTURE | Aquaculture | United Kingdom | 2013 | 1 |
| Aquaculture - Good Investment Opportunity for the Nation | Aquaculture | Nigeria | 2013 | 1 |
| Sharing aquaculture ideas | Aquaculture | New Zealand | 2013 | 1 |
| New zone to benefit aquaculture | Aquaculture | Australia | 2013 | 1 |
| Youths Should Try Aquaculture [opinion] | Aquaculture | Zambia | 2013 | 1 |
| Food security fund to transform sector. Drive to raise Dh550m for aquaculture | Aquaculture | Oman | 2013 | 1 |
| Cooke Aquaculture purchases stake in Chilean salmon farm; Markets Cooke Aquaculture purchased a 54 per cent stake in the South American company | Aquaculture | Canada | 2013 | 1 |
| Aquaculture project 'important first step, but more is needed' | Aquaculture | South Africa | 2013 | 1 |
| SA moves to nurture aquaculture sector | Aquaculture | South Africa | 2013 | 1 |
| Registration of freshwater aqua farms made compulsory | Aquaculture | India | 2013 | 0 |
| Cast your net to earn a living; - Tribal students undergo training at institute of aquaculture | Aquaculture | India | 2013 | 1 |
| Aquaculture and wild fisheries must aim for sustainable co-existence | Aquaculture | Scotland | 2013 | 1 |
| Scots Salmon farmers hit out at aquaculture guide | Aquaculture | Scotland | 2013 | -1 |
| Markey eyes fishing, opposes NOAA aquaculture | Aquaculture | USA | 2013 | -1 |
| OriginOil Partners with Aquaculture Producer to Study Impact of Its Technology in Transforming $100 Billion Global Market | Aquaculture | India | 2013 | 1 |
| Open day for aquaculture | Aquaculture | New Zealand | 2013 | 1 |
| 'Thought-provoking' study sees big potential in farmed trout | Aquaculture | New Zealand | 2013 | 1 |
| BRIEF: Aquaculture conference planned | Aquaculture | USA | 2013 | 0 |
| Quarantined salmon headed to New Brunswick for processing | Aquaculture | Canada | 2013 | -1 |
| Hungary : Improvement of research and innovation potential of Research Institute for Fisheries, Aquaculture and Irrigation (HAKI) to promote technological development in Central and Eastern European aquaculture | Aquaculture | United Kingdom | 2013 | 0 |
| Aquaculture, culinary arts students feed each other's projects at Cape May Tech | Aquaculture | USA | 2013 | 0 |
| Anti-aquaculture body 'aiding fish farm growth' | Aquaculture | United Kingdom | 2013 | -1 |
| Fewer workers in aquaculture | Aquaculture | Australia | 2013 | -1 |
| ENVIRONMENT : RECONCILING AQUACULTURE AND NATURE PROTECTION | Aquaculture | United Kingdom | 2012 | 1 |
| Make sure of demand, aquaculture project warned | Aquaculture | South Africa | 2012 | -1 |
| Video highlights benefits of soy use in fish feed; Consumers demand more fish; industry tries to increase supply | Aquaculture | USA | 2012 | 1 |
| Scientists question open-net farming | Aquaculture | Canada | 2012 | -1 |
| Waterworld | Aquaculture | India | 2012 | 0 |
| Salmon farming's foul record | Aquaculture | Canada | 2012 | -1 |
| Farmed fish feed high on food chain | Aquaculture | Canada | 2012 | -1 |
| Whyalla City Council will aim to protect and enhance | Aquaculture | Australia | 2012 | 0 |
| Carter backs $1b aquaculture target | Aquaculture | New Zealand | 2012 | 1 |
| Aquaculture research plans on view | Aquaculture | Wales | 2012 | 0 |
| Hughes-Gibb appoints aquaculture insurance broking, risk management division head | Aquaculture | United Kingdom | 2012 | 0 |
| 'Farmers lack drive to earn money' | Aquaculture | India | 2012 | -1 |
| Nepal sees potential in aquaponics | Aquaculture | Nepal | 2012 | 1 |
| Global Initiative Advocates Aquaculture to Fight Hunger | Aquaculture | Lagoa | 2012 | 1 |
| Aquaculture workshop slated Oct. 4 at UAPB | Aquaculture | USA | 2012 | 0 |
| Council wants shark risk looked at | Aquaculture | Australia | 2012 | -1 |
| Governor signs Chesbro aquaculture bill | Aquaculture | USA | 2012 | 0 |
| Catch 22 | Aquaculture | Australia | 2012 | -1 |
| Meet to discuss aquaculture trends and future | Aquaculture | India | 2012 | 0 |
| 20-year plan to boost aquaculture | Aquaculture | Oman | 2012 | 1 |
| Seminar on Investment Opportunities in Aquaculture Field | Aquaculture | Oman | 2012 | 1 |
| NEPAD Experts Advice Govt on Fisheries | Aquaculture | Sierra Leone | 2012 | 0 |
| Pact to boost fish production | Aquaculture | Malaysia | 2012 | 1 |
| Chesbro aquaculture bill headed to state governor | Aquaculture | USA | 2012 | 0 |
| Industry growing on back of tuna | Aquaculture | Australia | 2012 | -1 |
| Cooke Aquaculture CEO goes undercover; Undercover Boss | Aquaculture | Canada | 2012 | -1 |
| Gujarat can feed fish food to entire country | Aquaculture | India | 2012 | 1 |
| Rich Products' Texas manufacturing Plant Receives 'Best Aquaculture Practices' Certification | Aquaculture | India | 2012 | 1 |
| Leases to impact wildlife | Aquaculture | Australia | 2012 | -1 |
| Target for aquaculture industry | Aquaculture | Malaysia | 2012 | 0 |
| Fish farmers into aquaculture | Aquaculture | Australia | 2012 | 0 |
| National aquaculture group not supporting certification process | Aquaculture | Canada | 2012 | -1 |
| Farmed fish output to increase 33pc; Farmed fish | Aquaculture | United Kingdom | 2012 | 0 |
| Next time, make sure it's Aussie barramundi; Rob Broadfield Food Editor | Aquaculture | Australia | 2012 | 0 |
| Kiwi student following tradition in Australia | Aquaculture | New Zealand | 2012 | 0 |
| Barra on course | Aquaculture | Australia | 2012 | 0 |
| Play a role in aquaculture future | Aquaculture | Australia | 2012 | 1 |
| State-owned company for aquaculture on cards | Aquaculture | Oman | 2012 | 0 |
| Plan set for Fisheries Bill | Aquaculture | Scotland | 2012 | 0 |
| Aquaculture the key | Aquaculture | Australia | 2012 | 1 |
| Agriculture and Fisheries Minister Meets Korean Officials and Businessmen | Aquaculture | Oman | 2012 | 0 |
| Oyster industry given funding boost | Aquaculture | New Zealand | 2012 | 1 |
| Viet Namnam helps fish farming | Aquaculture | Australia | 2012 | 1 |
| Aquaculture salmon healthy, disease-free | Aquaculture | Canada | 2012 | 1 |
| Aquaculture potential of BTC area underlined | Aquaculture | India | 2012 | 1 |
| Training on aquaculture, sustainable farming | Aquaculture | India | 2012 | 1 |
| Sea cages for trial fish farm | Aquaculture | Australia | 2012 | 1 |
| Salmon farm considered at former Navy site in Corea | Aquaculture | USA | 2012 | 1 |
| Threat to mangrove swamps | Aquaculture | Malaysia | 2012 | -1 |
| Industry to ?adopt? beaches | Aquaculture | Australia | 2012 | 0 |
| 'Freshwater aquaculture vital for food security' | Aquaculture | India | 2012 | 1 |
| Tutor lifts NMIT's expertise | Aquaculture | New Zealand | 2012 | 0 |
| Expertise for state's aquaculture | Aquaculture | Australia | 2012 | 0 |
| Aquaculture sector faces 'pressures': report; ? APEC outlook comes just as N.B.-based company deals with fish farm virus | Aquaculture | Canada | 2012 | -1 |
| More debris washed ashore | Aquaculture | Australia | 2012 | -1 |
| AfDB Supports Largest Tilapia Fish Farm | Aquaculture | Harare | 2012 | 1 |
| China : Demonstration Project on Promotion of the Ecological Aquaculture Mode to Reduce Land-based Pollution (CPR/SGP/OP5/CORE/IW/11/01) Project | Aquaculture | Taiwan | 2012 | 1 |
| China : Demonstration of Eco-restoration and Eco-aquaculture in Mangrove Forest in the Beibu Gulf of Guangxi (CPR/SGP/OP5/CORE/IW/11/03) Project | Aquaculture | China | 2012 | 1 |
| U of G aquaculture researchers receive $1 million from Ottawa | Aquaculture | Canada | 2012 | 1 |
| Aquaculture oysters not free | Aquaculture | Australia | 2012 | -1 |
| BRIEF: Registration underway for 2012 aquaculture development event | Aquaculture | USA | 2012 | 0 |
| Clean Seas Tuna Limited has announced the | Aquaculture | Australia | 2012 | 0 |
| 'Disability Cannot Be an Obstacle to Productivity', Says an Aquaculture Operator | Aquaculture | Gambia | 2012 | 0 |
| N.S. group concerned that Kelly Cove Salmon wants to set up shop there | Aquaculture | Canada | 2012 | -1 |
| Belgium: Supporting governance and multi-stakeholder participation in aquaculture research and innovation (AQUAINNOVA) | Aquaculture | Belgium | 2012 | 1 |
| N.J. report sees great potential in aquaculture | Aquaculture | USA | 2012 | 1 |
| Italy: Aquaculture for Food Security, Poverty Alleviation and Nutrition (AFSPAN) | Aquaculture | Italy | 2012 | 1 |
| KOICA helps aquaculture development | Aquaculture | Sri Lanka | 2011 | 1 |
| Belgium: Supporting governance and multi-stakeholder participation in aquaculture research and innovation (AQUAINNOVA) | Aquaculture | Belgium | 2012 | 1 |
| Spain: Maximizing marine omega-3 retention in farmed fish: sustainable production of healthy food. (OMEGA3MAX) | Aquaculture | Spain | 2011 | 1 |
| Denmark: Multi-sensor automated water quality monitoring and control system for continuous use in recirculation aquaculture systems (AQUALITY) | Aquaculture | Denmark | 2011 | 0 |
| Incentives to attract aquaculture farmers in Oman | Aquaculture | Oman | 2011 | 1 |
| International Conference on Aquaculture Concludes | Aquaculture | Oman | 2011 | 0 |
| Aquaculture meet to boost tech input, investments in Oman | Aquaculture | Oman | 2011 | 1 |
| International Conference on Aquaculture Kicks off | Aquaculture | Oman | 2011 | 0 |
| Centre opened | Aquaculture | Australia | 2011 | 0 |
| Denmark: Multi-sensor automated water quality monitoring and control system for continuous use in recirculation aquaculture systems | Aquaculture | United Kingdom | 2011 | 0 |
| Research unit set to boost aquaculture | Aquaculture | Australia | 2011 | 1 |
| Egypt: Fisheries and aquaculture-Oriented Research Capacity | Aquaculture | Egypt | 2011 | 0 |
| Aqua zone group reps questioned | Aquaculture | Australia | 2011 | -1 |
| Aquaculture to help meet the global fish demand? | Aquaculture | India | 2011 | 1 |
| Malaysian Aquaculture Project Impresses Deputy Minister Nguvauva | Aquaculture | Malaysia | 2011 | 1 |
| Aquaculture boss quits | Aquaculture | New Zealand | 2011 | -1 |
| Aquaculture opponents lambasted by minister | Aquaculture | New Zealand | 2011 | 0 |
| A pearler of a plan released | Aquaculture | Australia | 2011 | 0 |
| Tumby won?t be at aqua zone meeting | Aquaculture | Australia | 2011 | 0 |
| Final submissions see clash of interests | Aquaculture | Canada | 2011 | -1 |
| New site worries landowners | Aquaculture | Australia | 2011 | -1 |
| In March 2011 PIRSA Fisheries and Aquaculture | Aquaculture | Australia | 2011 | 0 |
| Council questions finfish zone impact | Aquaculture | Australia | 2011 | -1 |
| Aquaculture zone proposal | Aquaculture | Australia | 2011 | 0 |
| Sustainable aquaculture? | Aquaculture | Wales | 2011 | 0 |
| Government Promotes Aquaculture | Aquaculture | Mozambique | 2011 | 1 |
| Is Aquaculture the Missing Link? [analysis] | Aquaculture | Harare | 2011 | 1 |
| Key witnesses draw crowd to spirited panel talks on salmon | Aquaculture | Canada | 2011 | 0 |
| Aquaculture gets lift from law change | Aquaculture | New Zealand | 2011 | 1 |
| Aquaculture law heralds expansion | Aquaculture | New Zealand | 2011 | 1 |
| Marine 'roadblock' removed | Aquaculture | New Zealand | 2011 | 1 |
| Aquaculture bill likely to pass today | Aquaculture | New Zealand | 2011 | 1 |
| AQUA 2000 : International conference on responsible aquaculture in the new millennium | Aquaculture | Belgium | 2011 | 1 |
| Advanced workshop: sea urchin aquaculture | Aquaculture | Japan | 2011 | 0 |
| Guatemala: Aquaculture and Farm Product Processing Technology Improvement Project Aquaculture | Aquaculture | Taiwan | 2011 | 1 |
| Aquaculture revives fishery; Newfoundland hails an industry that is putting social services recipients back into the workforce | Aquaculture | Canada | 2011 | 1 |
| Rice-fish culture | Aquaculture | Sri Lanka | 2011 | 0 |
| Marine stocks | Aquaculture | Sri Lanka | 2011 | 0 |
| Aquaculture to propel development | Aquaculture | Sri Lanka | 2011 | 1 |
| Protect region's aquaculture | Aquaculture | Sri Lanka | 2011 | 1 |
| NAQDA | Aquaculture | Sri Lanka | 2011 | 0 |
| Major species groups used in aquaculture | Aquaculture | Sri Lanka | 2011 | 0 |
| President to inaugurate 5th FAO confab | Aquaculture | Sri Lanka | 2011 | 0 |
| Development activities | Aquaculture | Sri Lanka | 2011 | 0 |
| Aquaculture production | Aquaculture | Sri Lanka | 2011 | 0 |
| Importance | Aquaculture | Sri Lanka | 2011 | 0 |
| Over 12,000 reservoirs to become fresh water fish farming tanks | Aquaculture | Sri Lanka | 2011 | 1 |
| Microbes as positive actors for more sustainable aquaculture | Aquaculture | Belgium | 2011 | 1 |
| ASEM Aquaculture Platform | Aquaculture | Belgium | 2011 | 0 |
| AQUAculture infrastructures for EXCELLence in European Fish research | Aquaculture | United Kingdom | 2011 | 1 |
| Papua New Guinea New Guinea: Increasing production from inland aquaculture in Papua New Guinea New Guinea for food and income security | Aquaculture | Papua New Guinea | 2011 | 1 |
| Imperial man still has hopes for aquaculture | Aquaculture | USA | 2011 | 1 |
| N.S. group tries to halt aquaculture expansion; Aquaculture Coalition says lawsuit a bid to force Nova Scotia government to tighten up controls on salmon farming | Aquaculture | Canada | 2011 | -1 |
| Codes of conduct crafted for shrimp industry | Aquaculture | Bangladesh | 2011 | 0 |
| Business buzz | Aquaculture | Brazil | 2011 | 0 |
| Led by China, fish farms soaring: study | Aquaculture | China | 2011 | 1 |
| Coalition protests Bay of Fundy aquaculture venture | Aquaculture | Canada | 2011 | -1 |
| Commerce eyes push to beef up aquaculture | Aquaculture | USA | 2011 | 1 |
| Embrace Aquaculture, French Envoy Advises | Aquaculture | Kenya | 2011 | 1 |
| Leaders in fish farming crowned | Aquaculture | Scotland | 2011 | 1 |
| SEA FOOD EXPORTS TO SCALE NEW HEIGHTS WITH REMARKABLE BREAKTHROUGH FOR ENSURING SUPPLY OF HIGH QUALITY ALL MALE SCAMPI SEEDS | Aquaculture | India | 2011 | 1 |
| Belgium: Microbes as positive actors for more sustainable aquaculture | Aquaculture | Belgium | 2011 | 1 |
| Aquaculture spurred marine production growth - BFAR | Aquaculture | Philippines | 2011 | 1 |
| Aquaculture projects revived | Aquaculture | Unknown | 2011 | 1 |
| Aquaculture in demand | Aquaculture | Wales | 2011 | 1 |
| India has bright future in aquaculture: Expert | Aquaculture | India | 2011 | 1 |
| Aquaculture contributes to half of marine output | Aquaculture | Philippines | 2011 | 1 |
| Pakistan: 'Need to initiate global campaign against commercial aquaculture' | Aquaculture | Pakistan | 2011 | -1 |
| Aquaculture Disappoints Esau | Aquaculture | Namibia | 2011 | -1 |
| Cell Aquaculture upgrades Thailand production facility | Aquaculture | Australia | 2011 | 0 |
| Fish farm sues activist group over 'Salmon Farming Kills' campaign | Aquaculture | Canada | 2011 | -1 |
| An act for fi sh farms; Aquaculture industry needs updated legal oversight | Aquaculture | Canada | 2011 | -1 |
| Aquaculture program offered in state webinar | Aquaculture | USA | 2011 | 0 |
| Facts from aquaculture industry come under fire | Aquaculture | Canada | 2011 | -1 |
| Conference highlight's state's aquaculture industry | Aquaculture | USA | 2011 | 0 |
| U.S. Proposes Aquaculture Guidelines | Aquaculture | USA | 2011 | 0 |
| N.C. aquaculture conference this week | Aquaculture | USA | 2011 | 0 |
| Finding the pot of gold in a fish pond | Aquaculture | South Africa | 2011 | 1 |
| Opening of aquaculture course hailed | Aquaculture | New Zealand | 2011 | 1 |
| Economic potential in aquaculture | Aquaculture | Wales | 2011 | 1 |
| Global fish farm production soars; But aquaculture's growth faces limits, even as it tops traditional fisheries | Aquaculture | USA | 2011 | 1 |
| Feb. hearings on proposed oyster aquaculture plan | Aquaculture | USA | 2011 | 0 |
| Fisheries Department Reiterates Need for More Aquaculture Practice | Aquaculture | Gambia | 2011 | 1 |
| Nothing fishy about it | Aquaculture | India | 2011 | 1 |
| Aquaculture changes will create Nanaimo jobs | Aquaculture | Canada | 2011 | 1 |
| New This Week | Aquaculture | Chile | 2011 | 0 |
| Aquaculture forum planned for Jan. 15 in Lewisburg | Aquaculture | USA | 2011 | 0 |
| Island gains 43 jobs in aquaculture; Most employees will be at work within next three weeks, DFO says | Aquaculture | Canada | 2011 | 1 |
| Aquaculture forum planned in January in Lewisburg | Aquaculture | USA | 2010 | 0 |
| BRIEF: Idaho aquaculture producers eligible for grant funding | Aquaculture | USA | 2010 | 0 |
| EU aquaculture plan described as positive | Aquaculture | United Kingdom | 2010 | 1 |
| New rules open way for fin fish farming | Aquaculture | New Zealand | 2010 | 1 |
| Law gives marine farms fast track | Aquaculture | New Zealand | 2010 | 1 |
| Aquaponics catching interest | Aquaculture | Wales | 2010 | 1 |
| New coalition wants major changes to aquaculture methods | Aquaculture | Canada | 2010 | -1 |
| Aqua the key word to growth | Aquaculture | Australia | 2010 | 1 |
| Cell Aquaculture begins Thailand production | Aquaculture | Thailand | 2010 | 0 |
| More rohu for the Bengali plate | Aquaculture | India | 2010 | 0 |
| NFA boosts aquaculture | Aquaculture | Papua New Guinea | 2010 | 1 |
| Course offers pathway to aquaculture | Aquaculture | New Zealand | 2010 | 1 |
| FAO proposes new guidelines for aquaculture certification | Aquaculture | Philippines | 2010 | 0 |
| Sassi aquaculture guide a big mistake | Aquaculture | South Africa | 2010 | -1 |
| Fish is Among Continent's Most Valuable Natural Resources | Aquaculture | Banjul | 2010 | 1 |
| Worthy Sassi list leads consumers into sea of confusion | Aquaculture | South Africa | 2010 | -1 |
| Government of Canada Invests in Nova Scotia's Aquaculture Industry | Aquaculture | Canada | 2010 | 1 |
| Boost for inland aquaculture | Aquaculture | Sri Lanka | 2010 | 1 |
| Harper Government Supports Six Innovative B.c. Aquaculture Projects | Aquaculture | Canada | 2010 | 1 |
| 'Huge leaps being taken' | Aquaculture | United Kingdom | 2010 | 1 |
| Cockles show promise as aquaculture product; Lowly bivalve robust species, quick to grow, say scientists | Aquaculture | Canada | 2010 | 1 |
| Foreshore and seabed win for aquaculture | Aquaculture | New Zealand | 2010 | 1 |
| FISH FARMS HOPE TO CATCH EYES | Aquaculture | USA | 2010 | 1 |
| CIFA holds workshop, training on community aquaculture | Aquaculture | India | 2010 | 0 |
| USA: MEPs call for strong European aquaculture sector | Aquaculture | United Kingdom | 2010 | 1 |
| LETTERS TO THE EDITOR | Aquaculture | Australia | 2010 | 0 |
| Aquaculture production increases; Environmental JOurnal | Aquaculture | USA | 2010 | 1 |
| The world's our oyster . . . | Aquaculture | Australia | 2010 | 1 |
| Aquaculture production evolves as a new avenue | Aquaculture | Australia | 2010 | 1 |
| Fish farms are more of a scientific issue | Aquaculture | Canada | 2010 | -1 |
| Is salmon farming sustainable? | Aquaculture | Canada | 2010 | -1 |
| Viet Nam, Egypt sign fishery, aquaculture agreement | Aquaculture | Viet Nam | 2010 | 1 |
| Report emphasizes economic importance of B.C. fish farms | Aquaculture | Canada | 2010 | 1 |
| Huon fish farm lands top awards | Aquaculture | Australia | 2010 | 1 |
| Canada's aquaculture industry netting $2.1 billion annually: report | Aquaculture | Canada | 2010 | 1 |
| Pakistan: More research needed in conservation & management of biodiversity | Aquaculture | Pakistan | 2010 | 0 |
| Fishing Policy Must Be Consistent | Aquaculture | USA | 2010 | -1 |
| Brunei: Bimp-Eaga holds high value aquaculture conference | Aquaculture | Philippines | 2010 | 0 |
| NOAA launches aquaculture plan, economic incentives | Aquaculture | USA | 2010 | 0 |
| Boosting aquaculture in regional growth area | Aquaculture | Malaysia | 2010 | 1 |
| BMIP-EAGA to open investment conference on high-value aquaculture | Aquaculture | Philippines | 2010 | 0 |
| Sri Lanka: US govt study identifies aquaculture opportunities in Eastern Sri Lanka | Aquaculture | Sri Lanka | 2010 | 1 |
| Highlands keen on fish farming | Aquaculture | Australia | 2010 | 1 |
| Fish farms could heal themselves; UVic is part of a group studying new aquaculture techniques | Aquaculture | Canada | 2010 | 1 |
| Hooked to success | Aquaculture | India | 2010 | 1 |
| Provincial journal | Aquaculture | Canada | 2010 | 0 |
| FISHERIES : MEPS AND SECTOR PUSH FOR MORE COMPETITIVE AQUACULTURE | Aquaculture | United Kingdom | 2010 | 0 |
| Special Report: A new paradigm in aquaculture | Aquaculture | Malaysia | 2010 | 0 |
| Bureau nets fresh catch | Aquaculture | Australia | 2010 | 0 |
| Cell Aquaculture appoints finance director, holds 1.6m shares | Aquaculture | Australia | 2010 | 0 |
| Cell Aquaculture issue 25.2m shares | Aquaculture | Australia | 2010 | 0 |
| Aquaculture viable if planned responsibly; U.S. bill aims to set standards to ensure environment is respected | Aquaculture | USA | 2010 | 1 |
| Research led by UNBSJ prof gets $5M in funding | Aquaculture | Canada | 2010 | 1 |
| European Fund invests $2.4m in Cell Aquaculture | Aquaculture | Malaysia | 2010 | 1 |
| Aquaculture plans a worry for environmental groups | Aquaculture | New Zealand | 2010 | -1 |
| B.C. slaps moratorium on finfish aquaculture licences | Aquaculture | Canada | 2010 | -1 |
| Govt shells out to aquaculture | Aquaculture | New Zealand | 2010 | 0 |
| Aquaculture changes will come at cost - experts | Aquaculture | New Zealand | 2009 | -1 |
| Aquatic growth | Aquaculture | India | 2009 | 0 |
| Employment and income generator for East | Aquaculture | Sri Lanka | 2009 | 1 |
| Trout farm ban costs New Zealand millions in lost revenue | Aquaculture | New Zealand | 2009 | 0 |
| No funding for agency | Aquaculture | New Zealand | 2009 | 0 |
| Cautious support | Aquaculture | New Zealand | 2009 | 0 |
| Job created to push bid for aquaculture | Aquaculture | New Zealand | 2009 | 1 |
| Marine-farm law changes eyed | Aquaculture | New Zealand | 2009 | -1 |
| Kick-start for aquaculture | Aquaculture | New Zealand | 2009 | 1 |
| Provincial journal | Aquaculture | Canada | 2010 | 0 |
| Local fish farms vie for awards | Aquaculture | Australia | 2009 | 0 |
| Trajectory of aquaculture extraordinary; Industry But Atlantic Canadians must invest heavier in science and technology, expert says | Aquaculture | Canada | 2009 | 1 |
| Farm-Fresh Fish -- With a Catch; Aquaculture Boom Raises Concerns | Aquaculture | USA | 2009 | -1 |
| Aquaculture coordinator featured speaker at Salmon Forum | Aquaculture | USA | 2009 | 0 |
| Rules Guiding Fish Farming In the Gulf Are Readied | Aquaculture | USA | 2009 | 0 |
| Scotland agrees aquaculture deal with Norway | Aquaculture | Scotland | 2009 | 1 |
| Keeping firm grip on aquaculture Controlled approach despite Asian farmers stealing march | Aquaculture | Australia | 2009 | -1 |
| Fishermen reeling | Aquaculture | Australia | 2009 | -1 |
| Anglers reeling | Aquaculture | Australia | 2009 | -1 |
| Guyana receives international assistance in aquaculture development | Aquaculture | Caribbean | 2009 | 1 |
| FISHERIES COUNCIL : MINISTERS WILLING TO GIVE BOOST TO AQUACULTURE | Aquaculture | United Kingdom | 2009 | 1 |
| Bid to resurrect aquaculture body | Aquaculture | United Kingdom | 2009 | 1 |
| Tide turning as aquaculture produces good quality fish; Canada accounts for only 0.2% of farmed seafood | Aquaculture | Canada | 2009 | 1 |
| WWF aquaculture green plan for shrimp and scallops sparks outrage | Aquaculture | United Kingdom | 2009 | -1 |
| Heatley plans sea change in aquaculture | Aquaculture | New Zealand | 2009 | 0 |
| Canada's aquaculture could be worth $2.8B - with help; Growth Many areas of the country are held back by inadequate public infrastructure, industry executive says | Aquaculture | Canada | 2009 | 1 |
| FISHERIES : COMMISSION ACTS TO BOLSTER EUROPEAN AQUACULTURE | Aquaculture | United Kingdom | 2009 | 1 |
| Western Cape tables strategy for fish farms | Aquaculture | South Africa | 2009 | -1 |
| Aquaculture review could scrap AMAs | Aquaculture | New Zealand | 2009 | 0 |
| Officials tout potential of aquaculture | Aquaculture | Canada | 2009 | 1 |
| Shucking the expanding market; Aquaculture Streamlining regulation, marketing region's products and resource sharing all(part of emerging policy | Aquaculture | Canada | 2009 | 1 |
| Shaping the - relatively young - aquaculture industry; Aquaculture Leaders meeting with ministers for a two-day summit that wraps up today | Aquaculture | Canada | 2009 | 1 |
| Changes on the cards for fish farming | Aquaculture | South Africa | 2009 | 0 |
| Salmon swimming against the recession; Aquaculture Sector gathering to outline priorities that will enable it to grow to its full potential | Aquaculture | Canada | 2009 | 1 |
| AQUACULTURE : COMMISSION ACTS TO BOLSTER EUROPEAN AQUACULTURE | Aquaculture | United Kingdom | 2009 | 1 |
| Aquaculture could yield more Irish jobs | Aquaculture | Ireland | 2009 | 1 |
| Letters to the Editor | Aquaculture | Australia | 2010 | 0 |
| Cell Aquaculture harvest first commercial barramundi | Aquaculture | Australia | 2009 | 1 |
| Can aquaculture save the oyster industry? | Aquaculture | USA | 2009 | 0 |
| Cooke Aquaculture setting up farmed cod demonstration project | Aquaculture | Canada | 2009 | 0 |
| A letter to the Minister of Fisheries... | Aquaculture | Canada | 2009 | 0 |
| Cell Aquaculture has $680k loss | Aquaculture | Australia | 2009 | -1 |
| Aquaculture mulls improving its image; Promotion Fish farmer wants to know what province is going to do back up industry | Aquaculture | Canada | 2009 | -1 |
| A focused strategy for success; Award Cooke Aquaculture Inc. named one of Canada's 50 best managed companies | Aquaculture | Canada | 2009 | 1 |
| Aquaculture hearings scheduled; Sessions lead up to industry summit in April | Aquaculture | Canada | 2009 | 0 |
| Family-owned firm takes pride in values | Aquaculture | Canada | 2009 | 1 |
| Federal government shows faith in aquaculture | Aquaculture | Canada | 2009 | 1 |
| Feds spread aquaculture net wider, beyond salmon | Aquaculture | Canada | 2009 | 1 |
| Looser legal net urged for aquaculture; New Fisheries chief calls for less red tape to help WA catch up with world initiatives on big fish-farming projects | Aquaculture | Australia | 2009 | 1 |
| Mangrove forests still under threat | Aquaculture | Malaysia | 2008 | -1 |
| Greater space for marine farming | Aquaculture | New Zealand | 2008 | 1 |
| Australis Aquaculture wins new $US1m loan | Aquaculture | USA | 2008 | 1 |
| Minister gets first-hand look at aquaculture industry | Aquaculture | Canada | 2008 | 0 |
| Aquaculture holds gleaming prospects | Aquaculture | Malaysia | 2008 | 1 |
| Clammers debate aquaculture rights | Aquaculture | USA | 2008 | -1 |
| Small fishers hard put to meet demand | Aquaculture | Philippines | 2008 | 0 |
| Payout to iwi set to lift region | Aquaculture | New Zealand | 2008 | 0 |
| Aquaculture plan is OK | Aquaculture | USA | 2008 | 0 |
| Fish-farm cages shifted from controversial site; Aquaculture High tides kept fish from eating and growing | Aquaculture | Canada | 2008 | -1 |
| FISHERIES : PARLIAMENT CALLS FOR FINANCIAL SUPPORT FOR AQUACULTURE | Aquaculture | United Kingdom | 2008 | 1 |
| Feed them to the fishes | Aquaculture | Austria | 2008 | 0 |
| Iwi unveils plans for seafood centre; AQUACULTURE DEVELOPMENT | Aquaculture | New Zealand | 2008 | 1 |
| Seafood centre planned; Iwi to foster research, aquaculture | Aquaculture | New Zealand | 2008 | 1 |
| Government trying to destroy aquaculture sector, IFA claims | Aquaculture | Ireland | 2008 | -1 |
| Aquaculture soon to replace commercial fishing operations | Aquaculture | Australia | 2008 | 0 |
| State may require waste discharge options; Water board, in effort to protect aquaculture, seeks input on disposal policies | Aquaculture | USA | 2008 | 1 |
| Aquaculture is the future `Australia imports about 70 per cent of the seafood we consume' | Aquaculture | Australia | 2008 | 1 |
| Aquaculture plant set for Machiasport | Aquaculture | USA | 2008 | 0 |
| Funds boost regional aquaculture industry | Aquaculture | New Zealand | 2008 | 1 |
| IFA claims licence delays are harming aquaculture | Aquaculture | Ireland | 2008 | 1 |
| Taking Stock | Aquaculture | Australia | 2008 | 0 |
| Australis Aquaculture begins stocking Viet Namnam operations | Aquaculture | Australia | 2008 | 1 |
| REGULATION ON AQUACULTURE STATISTICS | Aquaculture | United Kingdom | 2008 | 0 |
| 'Fifth-largest company in the world in aquaculture' receives government loan | Aquaculture | Canada | 2008 | 1 |
| Casting the fish net wide; Fishing Industry Cooke Aquaculture Inc. expanding operations with funding deal | Aquaculture | Canada | 2008 | 1 |
| Booming industry flexes its mussels | Aquaculture | Australia | 2008 | 1 |
| Australis Aquaculture to set up in Viet Namnam | Aquaculture | Australia | 2008 | 1 |
| Aquaculture The $70 billion industry is now the world's fastest-growing source of food production | Aquaculture | USA | 2008 | 1 |
| 'Great expectations'; Aquaculture facility in Franklin celebrates mission to raise quality salmon | Aquaculture | USA | 2008 | 1 |
| Vying for awards in aquaculture sector | Aquaculture | Scotland | 2008 | 0 |
| Demand for farmed fish growing; Deck head | Aquaculture | Canada | 2008 | 1 |
| Aquaculture production strategy smells a bit fishy to some | Aquaculture | Australia | 2008 | -1 |
| WWF to protect shellfish industry | Aquaculture | New Zealand | 2008 | 1 |
| Cell Aquaculture restructures board | Aquaculture | Australia | 2008 | 0 |
| Aquaculture a worthwhile angle | Aquaculture | Australia | 2008 | 1 |
| Microgreens prize-winning idea for N.B. entrepreneurs; Awards Innovative aquaculture startup in St. Andrews wins Innovation Foundation honour | Aquaculture | Canada | 2008 | 1 |
| Fishermen fear impact of Maces Bay fish farm | Aquaculture | Canada | 2008 | -1 |
| Awards scheme will now cover entire aquaculture sector | Aquaculture | Scotland | 2008 | 0 |
| Farm frenzy; Aquaculture will soon surpass wild-caught fish as the leading source for seafood | Aquaculture | USA | 2008 | 1 |
| 'Simplistic' salmon report slammed by industry; 'Pure and utter nonsense,' Wash. aquaculture consultant claims | Aquaculture | Canada | 2008 | -1 |
| Salmon report spurs debate; Report says sea lice from farms is hurting fish stocks, but critics say study is too simple | Aquaculture | Canada | 2008 | -1 |
| Fish farms harm wild salmon: report | Aquaculture | Canada | 2008 | -1 |
| RURAL SERIES WEEK 6 OF 6 SEAFOOD $1bn to flow from ocean's bounty | Aquaculture | Australia | 2008 | 0 |
| Aquaculture group plans expansion | Aquaculture | Australia | 2008 | 1 |
| Three agencies to combat pollution from aquaculture | Aquaculture | Philippines | 2008 | 0 |
| Aquaculture association for Wales launches | Aquaculture | Wales | 2008 | 1 |
| Cell Aquaculture has $1m cash | Aquaculture | Australia | 2008 | 1 |
| What's free about free-market aquaculture? | Aquaculture | USA | 2008 | 0 |
| Chance to reel in a fish farm with lease near Port Lincoln | Aquaculture | USA | 2008 | -1 |
| Aquaculture sites crowding them out, herring fishermen contend | Aquaculture | Canada | 2008 | -1 |
| Lotus key to a new blooming industry | Aquaculture | Australia | 2008 | 1 |
| Council draws a line in the sand on aquaculture | Aquaculture | USA | 2008 | -1 |
| Fish list reels in bulls | Aquaculture | Australia | 2008 | 0 |
| Future holds promise for high-end wild fish products | Aquaculture | USA | 2008 | 1 |
| Lotus industry mooted Project blossoming | Aquaculture | Australia | 2008 | 1 |
| Cell Aquaculture raises $1.2m | Aquaculture | Australia | 2007 | 1 |
| 2008 Aquaculture Forum Jan. 19 | Aquaculture | USA | 2007 | 0 |
| Hunt is on for top learners; express careers | Aquaculture | Scotland | 2007 | 0 |
| Open ocean farms could feed the world; Food: Official at aquaculture conference warns we need a replacement for dwindling fish stocks | Aquaculture | Canada | 2007 | 1 |
| Aquaculture industry looks at developments; Technology Conference explores ways of moving cages further offshore | Aquaculture | Canada | 2007 | 1 |
| Aquaculture's Â£400m boost for economy | Aquaculture | Scotland | 2007 | 1 |
| Aquaculture stance questioned | Aquaculture | New Zealand | 2007 | -1 |
| Offshore aquaculture the wave of the future; Lobbying: N.B. minister, counterparts seek long-term strategy | Aquaculture | Canada | 2007 | 1 |
| 'Aquaculture is the answer' | Aquaculture | Thailand | 2007 | 1 |
| Farmers learn tilapia rearing | Aquaculture | Australia | 2007 | 0 |
| Aquaculture expansion plan hits a snag | Aquaculture | USA | 2007 | -1 |
| Cell Aquaculture to buy out barramundi partner | Aquaculture | Australia | 2007 | 0 |
| Tap signs gas deal; New barra facility; Infrastructure fund; ASX fights legal action; Seek bags half share; Wind farm planned | Aquaculture | Australia | 2007 | 0 |
| Cell Aquaculture shuts down Mississippi operation | Aquaculture | USA | 2007 | -1 |
| Officials outnumber public at $180,000 aquaculture hui | Aquaculture | New Zealand | 2007 | 0 |
| Tax breaks for aqua farmers, investors | Aquaculture | South Africa | 2007 | 1 |
| Plan to encourage investment in aquaculture | Aquaculture | South Africa | 2007 | 1 |
| Cell Aquaculture announces barra venture in Malaysia | Aquaculture | Australia | 2007 | 1 |
| CELL ACQUACULTURE LAUNCHES MALAYSIAN BARRAMUNDI JV | Aquaculture | Australia | 2007 | 1 |
| Our coastal communities need more aquaculture | Aquaculture | Canada | 2007 | 1 |
| Clean sweep planned for Bay of Fundy beaches; Deer Island and Campobello Island to be targeted this summer, much of debris is aquaculture equipment | Aquaculture | Canada | 2007 | -1 |
| Meeting to look at aquaculture, past and future | Aquaculture | USA | 2007 | 0 |
| Strong debut for Western Kingfish | Aquaculture | Australia | 2007 | 1 |
| AUSTRALIS AQUACULTURE SECURES 12.6M FINGERLINGS PER YR FROM Viet NamNAM | Aquaculture | Australia | 2007 | 0 |
| UNB prof wants to use local aquaculture techniques to feed people globally | Aquaculture | Canada | 2007 | 1 |
| For details online about shrimp farming and aquaculture, go to: Ohio... | Aquaculture | USA | 2007 | 0 |
| Bringing food for thought to the table; Aquaculture Academics intend to generate viable solutions to developing nations' problems | Aquaculture | Canada | 2007 | 1 |
| Wakatu seafood arm eyes Australia venture | Aquaculture | New Zealand | 2007 | 1 |
| Billion dollar baby | Aquaculture | New Zealand | 2007 | 1 |
| Early settlement of Maori aquaculture claims sought | Aquaculture | New Zealand | 2007 | 0 |
| Marine farming's potential `immense' | Aquaculture | New Zealand | 2007 | 1 |
| CELL AQUACULTURE PROGRESSES FISH HATCHERY JV | Aquaculture | Australia | 2007 | 0 |
| Govt seeks to close aquaculture loophole | Aquaculture | New Zealand | 2007 | -1 |
| Maori ruling forces law change | Aquaculture | New Zealand | 2007 | 0 |
| Govt's $1b aquaculture plan applauded | Aquaculture | New Zealand | 2007 | 1 |
| Hooked on fish | Aquaculture | Australia | 2007 | 0 |
| Flow-on effect to employment | Aquaculture | Australia | 2007 | 1 |
| Industry prepares for future | Aquaculture | Australia | 2007 | 1 |
| Fisheries, fish processing & aquaculture goals | Aquaculture | USA | 2007 | 0 |
| CELL AQUACULTURE RECEIVES FIRST PAYMENT FROM MALAYSIAN JV PARTNER | Aquaculture | Australia | 2007 | 0 |
| Post graduate choices - 2007 - A life at sea is the life for me | Aquaculture | Ireland | 2007 | 0 |
| American fish farms going deep | Aquaculture | Canada | 2007 | 1 |
| ?The future is going to have aquaculture in it. It will have to, or we're not going to have enough fish. There's more demand than there is supply.? -- Gary Grant, Continental insurance agent and former fish grower | Aquaculture | USA | 2007 | 1 |
| Bohol positions as Visayas' aquaculture center | Aquaculture | Philippines | 2007 | 0 |
| Edinburgh showcase for leading players in fish farming | Aquaculture | Canada | 2007 | 1 |
| Steering new course for aquaculture | Aquaculture | New Zealand | 2007 | 0 |
| CELL AQUACULTURE AND TERENGGANU GOVERNMENT IN FINFISH JV | Aquaculture | Australia | 2007 | 0 |
| AUSTRALIS AQUACULTURE BARRAMUNDI ON US 'BEST CHOICES' LIST | Aquaculture | USA | 2007 | 1 |
| BAY RAIL | Aquaculture | USA | 2007 | 0 |
| NZ Aquaculture bids to secure funding | Aquaculture | New Zealand | 2007 | 0 |
| Aquaculture gains popularity in state | Aquaculture | USA | 2007 | 1 |
| Progress on aquaculture looms | Aquaculture | New Zealand | 2007 | 0 |
| Action needed on aquaculture | Aquaculture | Australia | 2007 | 0 |
| Vitarich research on aquaculture feeds to increase farmers' yields | Aquaculture | Japan | 2007 | 1 |
| Say on aquaculture welcome | Aquaculture | Australia | 2007 | 0 |
| WVU Extension forum to showcase aquaculture products, research | Aquaculture | USA | 2007 | 0 |
| New move at Glen | Aquaculture | New Zealand | 2007 | 0 |
| Winning respect in a tough arena | Aquaculture | New Zealand | 2007 | 0 |
| Aquaculture report urges growth, better regulation | Aquaculture | USA | 2007 | 1 |
| CPF to expand aquaculture in China | Aquaculture | Thailand | 2006 | 1 |
| CLINTONS INCREASE IN CELL AQUACULTURE | Aquaculture | Australia | 2006 | 0 |
| Aquaculture gets $2.9m from Govt | Aquaculture | New Zealand | 2006 | 1 |
| Aquaculture welcomes $2.9m funding | Aquaculture | New Zealand | 2006 | 1 |
| Aquaculture sector on tenterhooks | Aquaculture | Australia | 2006 | -1 |
| AQUACULTURE SALES GROW IN STATE | Aquaculture | USA | 2006 | 1 |
| Bringing some aquaculture | Aquaculture | USA | 2006 | 0 |
| Salmon recovery may be attributed to aquaculture | Aquaculture | Newfoundland | 2006 | 1 |
| Aquaculture council appoints new chief | Aquaculture | New Zealand | 2006 | 0 |
| The Future of Fish Farming; The Bush administration is right to push aquaculture, but it needs to push the right kind of aquaculture. | Aquaculture | USA | 2006 | 1 |
| Fish farming highlighted at meet | Aquaculture | Australia | 2006 | 1 |
| Fresh currents in fish farming | Aquaculture | South Africa | 2006 | 1 |
| Mote aquaculture project aims to keep seafood chefs working | Aquaculture | USA | 2006 | 1 |
| Aquaculture spawns serious concerns | Aquaculture | Canada | 2006 | -1 |
| Newfoundland: The 'promised land' of fishing; Company plans $135-million expansion of province's farmed salmon business | Aquaculture | Canada | 2006 | 1 |
| Marine farming `choked' | Aquaculture | New Zealand | 2006 | -1 |
| New minister schooled in fish farming; Aquaculture Ron Ouellette admits he's a guppy when it comes to the industry | Aquaculture | Canada | 2006 | -1 |
| Whirling disease hurts Utah aquaculture industry | Aquaculture | USA | 2006 | -1 |
| Whirling disease takes huge toll on fish business, census shows | Aquaculture | USA | 2006 | -1 |
| Report confirms Utah fish sales gutted | Aquaculture | USA | 2006 | -1 |
| CLINTON INVESTMENTS TRUST BUYS 1.8M CELL AQUACULTURE SHARES | Aquaculture | Australia | 2006 | 1 |
| CELL AQUACULTURE ENTERS ASIAN MARKET WITH MALAYSIAN HOA | Aquaculture | Australia | 2006 | 0 |
| Aquaculture part of the landscape: Anderton | Aquaculture | New Zealand | 2006 | 0 |
| Aquaculture industry struggles against odds; Billion-dollar vision clashes with reality so National MP wants legislative changes to ensure research can be done and incumbent farmers aren't disadvantaged | Aquaculture | New Zealand | 2006 | -1 |
| Oysters drive aquaculture in Marin | Aquaculture | USA | 2006 | 1 |
| Nelson home to new aquaculture organisation | Aquaculture | New Zealand | 2006 | 0 |
| Grand plan for aquaculture | Aquaculture | New Zealand | 2006 | 1 |
| Aquaculture dives into training | Aquaculture | New Zealand | 2006 | 0 |
| Aquaculture - environmentally friendly, ecologically responsible | Aquaculture | Australia | 2006 | 1 |
| Aquaculture - leading edge or bleeding edge | Aquaculture | Australia | 2006 | 0 |
| HUGE GROWTH May be worth $1bn in foreseeable future; Seafood set to become fast-lane winner | Aquaculture | Australia | 2006 | 0 |
| World view on way ahead | Aquaculture | Australia | 2006 | 0 |
| AQUACULTURE Buy-up of SA outlets begins; $50m for fish farm empire | Aquaculture | Australia | 2006 | 1 |
| Plenty of mussel in aquaculture; Leases snapped up | Aquaculture | Australia | 2006 | 1 |
| CELL AQUACULTURE BEGINS US BARRAMUNDI PRODUCTION | Aquaculture | Australia | 2006 | 0 |
| Fish or fowl? | Aquaculture | Canada | 2006 | -1 |
| Lupin fish fodder | Aquaculture | Australia | 2006 | 0 |
| CELL AQUACULTURE AIR-FREIGHTS MORE BARRAMUNDI TO HOLLAND | Aquaculture | Australia | 2006 | 0 |
| Marine battle rages on | Aquaculture | New Zealand | 2006 | -1 |
| Aquaculture workshops scheduled at WCCC | Aquaculture | USA | 2006 | 0 |
| Aquaculture sales chief | Aquaculture | Australia | 2006 | 0 |
| Marine farmers fall foul of aquaculture laws | Aquaculture | New Zealand | 2006 | -1 |
| Government tries to turn the tide with new Victorian aquaculture leases | Aquaculture | Australia | 2006 | 1 |
| Barra make safe landing | Aquaculture | Australia | 2006 | 1 |
| Barra babies flown out to Netherlands | Aquaculture | Australia | 2006 | 1 |
| CELL AQUACULTURE OPENS Netherlands FACILITIES | Aquaculture | Australia | 2006 | 1 |
| CELL AQUACULTURE IN JV WITH DELTA AQUACULTURE | Aquaculture | Australia | 2006 | 0 |
| The dangers of acquaculture | Aquaculture | Canada | 2006 | -1 |
| Aquaculture committee moves ahead | Aquaculture | Canada | 2006 | 1 |
| CELL AQUACULTURE BUYS INTO US AQUACULTURE COMPANY | Aquaculture | USA | 2006 | 1 |
| Currie fish farmers succeeding in aquaculture | Aquaculture | USA | 2006 | 1 |
| WVU Extension aquaculture forum to catch fish producers, draw public Jan. 21 | Aquaculture | USA | 2006 | 0 |
| City hails release of aquaculture leases | Aquaculture | Australia | 2006 | 1 |
| Fine kettle of fish | Aquaculture | Australia | 2006 | 1 |
| Canadian salmon firm expands Maine holdings | Aquaculture | Canada | 2006 | 1 |
| Lack of numbers forces aquaculture school to close | Aquaculture | Australia | 2005 | -1 |
| Minister proposes powers to protect freshwater fisheries | Aquaculture | Scotland | 2005 | 0 |
| RM5m hatchery in Tasik Kenyir | Aquaculture | Malaysia | 2005 | 0 |
| Ex-ISU biologist extols viability of aquaculture | Aquaculture | USA | 2005 | 1 |
| CELL AQUACULTURE TAKES 30% IN MARINE FARMS | Aquaculture | Australia | 2005 | 0 |
| CELL AQUACULTURE TO SELL LAND FOR $2.6M | Aquaculture | Australia | 2005 | 0 |
| CELL AQUACULTURE TO PROCEED WITH US JOINT VENTURE | Aquaculture | USA | 2005 | 0 |
| LEADING A CULTURED LIFE ..ZO: B -All Region | Aquaculture | Malaysia | 2005 | 0 |
| FISH FARMS NEED GOVT SUPPORT, CLEAN RIVERS | Aquaculture | Taiwan | 2005 | 1 |
| Support flows for aquaculture declaration | Aquaculture | Canada | 2005 | 1 |
| CELL AQUACULTURE APPOINTS ALTERNATE DIRECTOR | Aquaculture | Australia | 2005 | 0 |
| AUSTRALIS AQUACULTURE REPORTS 1.5M LOSS | Aquaculture | Australia | 2005 | -1 |
| CELL AQUACULTURE APPOINTS AGENT FOR CHINA, S-E ASIA EXPANSION | Aquaculture | Australia | 2005 | 0 |
| Canadians fly the flag at aquaculture show | Aquaculture | Canada | 2005 | 0 |
| CELL AQUACULTURE APPOINTS DIRECTORS | Aquaculture | Australia | 2005 | 0 |
| CELL AQUACULTURE SIGNS DEAL FOR HOLLAND JV | Aquaculture | Australia | 2005 | 0 |
| AUSTRALIS AQUACULTURE HAS $3.9M CASH | Aquaculture | USA | 2005 | 0 |
| TOKM has new obligation | Aquaculture | New Zealand | 2005 | 0 |
| Criterion | Aquaculture | Australia | 2005 | 0 |
| SA's fish farms angle for a new type of catch; SPECIAL REPORT - South AUSTRALIA | Aquaculture | Australia | 2005 | 0 |
| Ocean aquaculture | Aquaculture | USA | 2005 | 0 |
| Prime real estate | Aquaculture | Canada | 2005 | 0 |
| Tassie salmon market threats | Aquaculture | Australia | 2005 | -1 |
| Bigger would be better, report says | Aquaculture | Canada | 2005 | 1 |
| Aquaculture part of 'long-term plan' for Harbour Breton | Aquaculture | Newfoundland | 2005 | 1 |
| Aquaculture works | Aquaculture | USA | 2005 | 1 |
| Weston sells last fish unit | Aquaculture | Canada | 2005 | 0 |
| World is your oyster | Aquaculture | Australia | 2005 | 1 |
| Radical Bush plan boosts B.C. farmed fish industry: Cod, halibut, red snapper to be raised | Aquaculture | Canada | 2005 | 1 |
| Measure to encourage fish, shellfish farms in federal waters | Aquaculture | USA | 2005 | 1 |
| Plan Would Expand Ocean Fish Farming | Aquaculture | USA | 2005 | 1 |
| $1.4m development could revolutionise aquaculture | Aquaculture | Australia | 2005 | 1 |
| Marlborough pushes aquaculture industry | Aquaculture | New Zealand | 2005 | 1 |
| NEW FLOATS | Aquaculture | Australia | 2005 | 0 |
| Fishing For Answers | Aquaculture | USA | 2005 | -1 |
| New Floats | Aquaculture | Australia | 2005 | 0 |
| Fish farm supporters attack James | Aquaculture | Canada | 2005 | -1 |
| Conference will put focus on aquaculture | Aquaculture | United Kingdom | 2005 | 0 |
| THE AQUA CULTURE PHENOMENON | Aquaculture | USA | 2005 | 1 |
| Maori set for aquaculture boost | Aquaculture | New Zealand | 2005 | 1 |
| UBC and feds eye aquaculture | Aquaculture | Canada | 2005 | 1 |
| Education advertising feature;Sea change on the Apple Isle | Aquaculture | South Africa | 2005 | 0 |
| DNR QUICK TO LICENSE, SLOW TO EVALUATE AQUACULTURE IN WETLANDS | Aquaculture | USA | 2005 | -1 |
| Pro fish farm group fires back at report | Aquaculture | USA | 2005 | 0 |
| Minister lands lead role in Scottish aquaculture forum | Aquaculture | United Kingdom | 2005 | 0 |
| Bill aims to boost Maryland's $700 million seafood industry | Aquaculture | USA | 2005 | 1 |
| Forced to shell out more for licences, producers say aquaculture could sink; AGRIBUSINESS | Aquaculture | Australia | 2005 | -1 |
| Scots fish farmers can lead Europe, says aquaculture expert | Aquaculture | United Kingdom | 2005 | 1 |
| Shetland salmon farmer takes over as chairman of aquaculture group | Aquaculture | United Kingdom | 2005 | 0 |
| New marine harvest will employ 600 in Scotland | Aquaculture | United Kingdom | 2005 | 1 |
| Ex-minister to head salmon forum | Aquaculture | Canada | 2004 | 0 |
| Enrol 2005 advertising feature;Sign on for seachange | Aquaculture | South Africa | 2004 | 0 |
| Family aims to clean up with sea mullet | Aquaculture | Australia | 2004 | 1 |
| Seafood the main course in new TAFE aquaculture offering; EDUCATION | Aquaculture | Australia | 2004 | 0 |
| RESEARCH SHRIMP CROP IS A-COMIN'; A UF AG PROJECT UNVEILS AQUACULTURE POTENTIAL | Aquaculture | USA | 2004 | 1 |
| Loan guarantees for fish farmers | Aquaculture | Canada | 2004 | 1 |
| Booming NZ aquaculture industry awash with dissent | Aquaculture | New Zealand | 2004 | -1 |
| Oyster, mussel farm had seeds at RWU aquaculture program | Aquaculture | USA | 2004 | 0 |
| Aquaculture bill worries industry | Aquaculture | New Zealand | 2004 | -1 |
| Keep moratorium call | Aquaculture | New Zealand | 2004 | -1 |
| Pilot study puts spotlight on aquaculture industry | Aquaculture | Australia | 2004 | 1 |
| Oak Hill economic growth may rest with aquaculture | Aquaculture | USA | 2004 | 1 |
| Nothing fishy about this industry's sunny outlook; South AUSTRALIA | Aquaculture | Australia | 2004 | 1 |
| QUALITY OF LIFE INDEX ; Our times as told by statistics | Aquaculture | USA | 2004 | 0 |
| Govt sets nets for opposition lines | Aquaculture | New Zealand | 2004 | -1 |
| Sustaining Seafood Production In A Growing World | Aquaculture | USA | 2004 | 1 |
| Govt plan to jump in upsets seafood industry | Aquaculture | New Zealand | 2004 | 0 |
| Sealord sees hooks in Govt's marine farm plan for Maori | Aquaculture | New Zealand | 2004 | -1 |
| Flaunting fish farms; Port tipped for rise in aquaculture | Aquaculture | Australia | 2004 | 1 |
| Aquaculture industry asked for opinion | Aquaculture | United Kingdom | 2004 | 0 |
| Studying the abundance of the sea | Aquaculture | Australia | 2004 | 0 |
| Aquaculture set to scale new heights | Aquaculture | Australia | 2004 | 1 |
| Fish farms strategy a failure, say greens | Aquaculture | United Kingdom | 2004 | -1 |
| Fish farming is farming, not fishing, and Agriculture Canada should oversee it: report | Aquaculture | Canada | 2004 | -1 |
| Agriculture Canada should oversee fish farm ops: report | Aquaculture | Canada | 2004 | 0 |
| Pollution laws hurt fish farms, report says: Study encourages minister to free aquaculture industry from Fisheries Act oversight | Aquaculture | Canada | 2004 | -1 |
| Fish farming is farming, commissioner insists | Aquaculture | Canada | 2004 | 0 |
| GRANTS TO HELP FISHING AND AQUACULTURE | Aquaculture | Wales | 2004 | 1 |
| Lawmakers to consider aquaculture's future | Aquaculture | USA | 2004 | 0 |
| Rhode Island is ready to ride aquaculture wave | Aquaculture | USA | 2004 | 1 |
| Capital vs. labour | Aquaculture | Canada | 2004 | 0 |
| Aquaculture's promise | Aquaculture | USA | 2004 | 1 |
| An idea we can't throw back; The only way to meet the future demand for fish will be farming, says WILLIAM HOGARTH, the man responsible for America's coastal waters | Aquaculture | Canada | 2004 | 1 |
| Fraser report assails 'secrecy' in aquaculture | Aquaculture | Canada | 2004 | -1 |
| Aquaculture team slams Government failure | Aquaculture | New Zealand | 2004 | -1 |
| Aquaculture is fenced in by fish farmers' lack of property rights | Aquaculture | Canada | 2004 | -1 |
| First Nations hold aquaculture advantage | Aquaculture | Canada | 2003 | 1 |
| Freeze on marine farms extended | Aquaculture | New Zealand | 2003 | -1 |
| New Stirling Freshwater Research Unit Is Opened | Aquaculture | United Kingdom | 2003 | 0 |
| The boom in the dishy fish | Aquaculture | Australia | 2003 | 0 |
| Fishing for business: 'Blue revolution' could net a profitable future for B.C.'s fish farms | Aquaculture | Canada | 2003 | 1 |
| Fish farms may have it easier | Aquaculture | Canada | 2003 | 1 |
| Right-to-farm law change makes it easier to establish B.C. fish farms NDP | Aquaculture | Canada | 2003 | 1 |
| B.C. amends right-to-farm legislation for aquaculture | Aquaculture | Canada | 2003 | 1 |
| B.C. loosens up fish farming rules | Aquaculture | Canada | 2003 | 1 |
| AQUACULTURE WORTH $130 MILLION;A study released today assesses the total value of fish farming sales, wages and related spending in Maine. | Aquaculture | USA | 2003 | 1 |
| Booming business in fisheries sector | Aquaculture | Malaysia | 2003 | 0 |
| Net yourself a top spot in aquaculture | Aquaculture | Australia | 2003 | 1 |
| New regulations to boost fish farming | Aquaculture | Malaysia | 2003 | 1 |
| SORTED AND THE CITY: FISHING FOR EXTRA PROFIT | Aquaculture | United Kingdom | 2003 | 0 |
| Aquaculture Industry Policy Is Backed By Scottish Parliament | Aquaculture | United Kingdom | 2003 | 1 |
| Marine farming high and dry | Aquaculture | New Zealand | 2003 | -1 |
| Aquaculture holds much promise | Aquaculture | Malaysia | 2003 | 1 |
| Minister initiates environmental code for aquaculture | Aquaculture | Ireland | 2003 | 1 |
| Industry on the crest of export wave | Aquaculture | Australia | 2003 | 1 |
| The Fish on Our Plates, the Fish in the Sea | Aquaculture | USA | 2003 | 0 |
| Bills raise tension over management of fish farms;Emotions run high during public hearings on legislation that would affect aquaculture's future. | Aquaculture | USA | 2003 | -1 |
| Aquaculture committee has pro-industry bias, MP claims | Aquaculture | Canada | 2003 | -1 |
| Cummins unimpressed with aqua-report | Aquaculture | Canada | 2003 | -1 |
| Farmed fish provide a taste of aquaculture center future | Aquaculture | USA | 2003 | 0 |
| Hooked on seafood | Aquaculture | Australia | 2003 | 0 |
| Nets cast for Coast dividend | Aquaculture | Australia | 2003 | 0 |
| DFO requires fish-farm overhaul from Chretien | Aquaculture | Canada | 2003 | -1 |
| Demand drives aquaculture | Aquaculture | Australia | 2003 | 1 |
| MAS scales up to meet the commercial market | Aquaculture | Australia | 2003 | 1 |
| Hagen received industry donations, MacPhail says: He replaces van Dongen on aquaculture file | Aquaculture | Canada | 2003 | 0 |
| Cod aquaculture | Aquaculture | Scotland | 2003 | 0 |
| AROUND THE REGIONS;Fishing sector to net $650m | Aquaculture | South Africa | 2003 | 0 |
| Help needed to harvest a billion dollars | Aquaculture | Australia | 2003 | 0 |
| AQUACULTURE;Ocean banquet | Aquaculture | Australia | 2003 | 1 |
| Aquaculture a pearl for north, experts predict | Aquaculture | New Zealand | 2003 | 1 |
| Curbing the spread of disease in aquaculture | Aquaculture | Malaysia | 2003 | -1 |
| Aquaculture revenues drop for second year: Declining American market demand has big impact, Vancouver Sun reporter Bruce Constantineau finds | Aquaculture | Canada | 2003 | -1 |
| States to help develop aquaculture industrial zones | Aquaculture | Malaysia | 2003 | 1 |
| Billion dollar decision welcomes fish farms | Aquaculture | Canada | 2002 | 1 |
| Aquaculture industry still a zone of contention | Aquaculture | Australia | 2002 | -1 |
| Relaunch of aquaculture group | Aquaculture | United Kingdom | 2002 | 1 |
| Yorke Peninsula ;Aquaculture under review | Aquaculture | Australia | 2002 | -1 |
| Minister's push | Aquaculture | United Kingdom | 2002 | 1 |
| Fisheries pumps cash into aquaculture | Aquaculture | Canada | 2002 | 1 |
| New regulations in place to license inland aquaculture farms | Aquaculture | Malaysia | 2002 | 1 |
| Hebrides get new supremo to push aquaculture industry | Aquaculture | United Kingdom | 2002 | 1 |
| Cod trials' results | Aquaculture | United Kingdom | 2002 | 0 |
| State of Maryland considers task force on seafood industry | Aquaculture | USA | 2002 | 0 |
| Aquaculture Industry fears loss of $400m | Aquaculture | New Zealand | 2002 | -1 |
| Niwa to make most of marine farming pause | Aquaculture | New Zealand | 2002 | 0 |
| Govt marine farm freeze angers firm | Aquaculture | New Zealand | 2002 | -1 |
| Province; feds seek harmony | Aquaculture | Canada | 2001 | 0 |
| Aquaculture forum gets Website | Aquaculture | Canada | 2001 | 0 |
| College center is dedicated; Aquaculture facility already drawing praise from students | Aquaculture | USA | 2001 | 1 |
| Reaping a barra harvest; Northern Territory | Aquaculture | Australia | 2001 | 0 |
| University zoology lecturer to be laid to rest | Aquaculture | United Kingdom | 2001 | 0 |
| Aquaculture spells relief for fishery | Aquaculture | USA | 2001 | 1 |
| Two-day forum on fish farming set Oct. 26-27 | Aquaculture | Canada | 2001 | 0 |
| More research on aquaculture - Senate group: Major challenge is to achieve balance between competing users of environment | Aquaculture | Canada | 2001 | -1 |
| Fish farm industry scales up | Aquaculture | Australia | 2001 | 1 |
| Fishing for more | Aquaculture | Malaysia | 2001 | 0 |
| Fish farming worth risks, say MLA trio: Visser, Hagen and Belsey assess the situation | Aquaculture | Canada | 2001 | 1 |
| Aquaculture meeting | Aquaculture | United Kingdom | 2001 | 0 |
| Push to inject more muscle into water farming | Aquaculture | New Zealand | 2001 | 1 |
| Fisheries department ignorant of risks posed by farmed salmon, report says: Big gaps in knowledge criticized in federal audit | Aquaculture | Canada | 2001 | -1 |
| Mohegans Deal a New Hand: A Fishery | Aquaculture | USA | 2001 | 0 |
| From salmon to shellfish; Portland will host a regional aquaculture conference this weekend | Aquaculture | USA | 2001 | 0 |
| New aquaculture guidelines urged | Aquaculture | United Kingdom | 2001 | 0 |
| WA aquaculture boom | Aquaculture | Australia | 2000 | 1 |
| Aquaculture production to be raised six-fold | Aquaculture | Malaysia | 2000 | 1 |
| Aquaculture nets grant to launch it into future | Aquaculture | Canada | 2000 | 1 |
| Aquaculture industry to be strengthened by fund | Aquaculture | Canada | 2000 | 1 |
| Aquaculture plans snagged by red tape; SEAFOOD | Aquaculture | Australia | 2000 | -1 |
| Aquaculture regulations need overhaul, expert says: Annual conference: Lack of consistency hurting industry's growth | Aquaculture | Canada | 2000 | -1 |
| Aquaculture regulations must be overhauled: experts say | Aquaculture | Canada | 2000 | -1 |
| DFO abandoning wild salmon: environmentalists | Aquaculture | Canada | 2000 | -1 |
| Fish farming dreams a 'nightmare' to environmentalists: They claim Ottawa is silently deserting wild fishing, especially salmon, in favour of fish farming. | Aquaculture | Canada | 2000 | -1 |
| Fishermen fear federal vision for aquaculture: Environmentalists see contamination of wild fish stocks | Aquaculture | Canada | 2000 | -1 |
| DFO abandoning wild salmon in favour of fish farming: environmentalists | Aquaculture | Canada | 2000 | -1 |
| Sea change for cane farms | Aquaculture | Australia | 2000 | 0 |
| LAND COUNCIL PLANS $10M FISH FARM;MULTI-MILLION-DOLLAR AQUACULTURE VENTURE MAY GENERATE ANOTHER 500 JOBS FOR NAROOMA'" | Aquaculture | Australia | 2000 | 1 |
| Aquaculture - the pearl in our oyster | Aquaculture | Australia | 2000 | 1 |
| Field Notes | Aquaculture | Canada | 2000 | 0 |
| 1999 ROYAL ADELAIDE SHOW; A pearl of great price | Aquaculture | Australia | 2000 | 0 |
| Aquaculture way to ensure fish supply beyond 2000 | Aquaculture | Malaysia | 2000 | 1 |
| Cutting red tape on fish farming; VICTORIA - SEAFOOD | Aquaculture | Australia | 1999 | 1 |
| Salmon farm's new managers to visit Scotland | Aquaculture | United Kingdom | 1999 | 0 |
| Boost for aquaculture in Kenyir Lake | Aquaculture | Malaysia | 1999 | 1 |
| Aquaculture in demand | Aquaculture | Wales | 2011 | 1 |
| Aquaculture's ready to scale up production | Aquaculture | Australia | 1999 | 1 |
| Bubbling fish farms rival wine | Aquaculture | Australia | 1999 | 1 |
| A state with bigger fish to fry; SEAFOOD | Aquaculture | Australia | 1999 | 1 |
| 330,200ha of land, bodies of water suitable for aquaculture | Aquaculture | Malaysia | 1999 | 1 |
| Call of the wild salmon grows weaker by the year | Aquaculture | Canada | 1999 | -1 |
| Fish farmers seek clarity on marine park plans; VICTORIA | Aquaculture | Australia | 1999 | -1 |
| Prime coast earmarked for fish farming | Aquaculture | Australia | 1999 | 0 |
| Lobster farming a big step closer | Aquaculture | Australia | 1999 | 1 |
| Aquaculture offers a meal ticket; FEATURES | Aquaculture | Australia | 1999 | 1 |
| Bigger fish to fry for aquaculture business | Aquaculture | Australia | 1999 | 1 |
| FISH SCHOOL; Aquaculture classes at South Brunswick High raise, feed, and now, sell their own crops | Aquaculture | USA | 1999 | 0 |
| Aquaculture worth over (GBP) 6m | Aquaculture | United Kingdom | 1999 | 1 |
| State plans to double aquaculture output; VICTORIA | Aquaculture | Australia | 1999 | 1 |
| BRUNSWICK PEOPLE / Douglas Holland; Expert cultivates fish farming | Aquaculture | USA | 1999 | 1 |
| THE REGION; BRUNSWICK; College breaks ground on aquaculture center | Aquaculture | USA | 1999 | 1 |
| Aquaculture a good solution to boost fish supply | Aquaculture | Malaysia | 1999 | 1 |
| Sea of wisdom | Aquaculture | Australia | 1999 | 1 |
| Aquaculture may not be solution to increasing fish supply | Aquaculture | Malaysia | 1999 | -1 |
| JCU leads way in study of aquaculture | Aquaculture | Australia | 1999 | 0 |
| Aquaculture can help to bring down import bills' | Aquaculture | Malaysia | 1999 | 1 |
| Netting the profits of fish farming; North Queensland | Aquaculture | Australia | 1998 | 1 |
| CLAM FARMING COULD RESTORE STEAMER RANKS; A FEDERAL GRANT WILL FUND A CONTROVERSIAL AQUACULTURE PROGRAM. | Aquaculture | USA | 1998 | 0 |
| Book on local aquaculture practices launched | Aquaculture | Malaysia | 1998 | 0 |
| Dr Sulaiman: Have more aquaculture activities | Aquaculture | Malaysia | 1998 | 1 |
| Warning on future of aquaculture | Aquaculture | United Kingdom | 1998 | -1 |
| Farmers of the future | Aquaculture | Australia | 1998 | 0 |
| Aquaculture to help meet demand for protein food | Aquaculture | Malaysia | 1998 | 1 |
| North centre of aquaculture | Aquaculture | Australia | 1998 | 0 |
| Aquaculture sector has bright future' | Aquaculture | Malaysia | 1998 | 1 |
| The Blue revolution | Aquaculture | Australia | 1998 | 1 |
| JOBS, PROFITS AND INNOVATION...South AUSTRALIA GOES FISHING;AND COMES UP WITH SOME IMPRESSIVE CATCHES; new industry to;reel in millions | Aquaculture | South Africa | 1998 | 1 |
| Aquaculture booms in state | Aquaculture | Australia | 1998 | 1 |
| Aquaculture aims for $200m annual value; Fish farming's;funds push | Aquaculture | Australia | 1998 | 1 |
| Aquaculture way to boost fish stocks | Aquaculture | Malaysia | 1998 | 1 |
| Study will assess jobs potential of aquaculture at Shannon industry | Aquaculture | Ireland | 1998 | 0 |
| May 14 hearing for petition to wind up prawn company | Aquaculture | Malaysia | 1998 | 0 |
| Offering fishermen aquaculture option to boost catch | Aquaculture | Malaysia | 1998 | 1 |
| FISH FARMING AND NEW BILL | Aquaculture | Ireland | 1998 | 0 |
| State battling to foster fish farming | Aquaculture | Australia | 1996 | -1 |
| AQUACULTURE ON A ROLL | Aquaculture | Australia | 1996 | 1 |
| Environ-friendly aquaculture projects guideline | Aquaculture | Malaysia | 1996 | 1 |
| LETTERS | Aquaculture | USA | 1996 | 0 |
| Fished-out New England turns to commercial farms | Aquaculture | Canada | 1996 | 1 |
| These fish are a well-cultured lot | Aquaculture | Canada | 1996 | 1 |
| SA casts for more fish exports | Aquaculture | Australia | 1996 | 0 |
| Move to regulate aquaculture activities | Aquaculture | Malaysia | 1996 | 0 |
| Adverse effects of mangrove loss | Aquaculture | Malaysia | 1996 | -1 |
| Regulating use of mangrove forests for aquaculture | Aquaculture | Malaysia | 1996 | -1 |
| Fish that are reared to be main source of supply | Aquaculture | Malaysia | 1996 | 0 |
| GROWING FISH FARMS FILL IMPORTANT ROLE IN STATE ECONOMY;AQUACULTURE EVOLVES INTO A $ 43 MILLION-A-YEAR INDUSTRY AND AN ECONOMIC PILLAR;IN DOWN EAST TOWNS. | Aquaculture | USA | 1996 | 1 |
| Fish farming set for boom Size to rival wine industry,;Premier tips | Aquaculture | Australia | 1996 | 1 |
| Fish food option for grain growers | Aquaculture | Australia | 1996 | 0 |
| CAP calls for review of aquaculture projects | Aquaculture | Malaysia | 1996 | 0 |
| Should Canada be casting nets to potential of fish farming? | Aquaculture | Canada | 1996 | -1 |
| Fish farms expansion will help create jobs | Aquaculture | Canada | 1996 | 1 |
| CAPTIVE RAW PRAWN IS HEADING FOR BOOMING FUTURE IN FAR NO | Aquaculture | Australia | 1996 | 1 |
| AQUACULTURE DFO helps set goals and map a plan Campbell River meeting helps provide a blueprint for the future | Aquaculture | Canada | 1993 | 0 |
| AQUACULTURE Fear of change presents only one obstacle Environmental concerns are being addressed, but opposition remains | Aquaculture | Canada | 1993 | -1 |
| AQUACULTURE The New Wave Aquaculture provides hope as commercial fisheries decline Government has dragged its feet in embracing unavoidable change It's not trying to displace fishing and it's not just fish farming. It's a thriving young industry that could grow to a $1-billion business in Canada by the end of the decade. | Aquaculture | Canada | 1993 | 1 |
| AQUACULTURE Canada follows growing trend Growth industry tied to increase in exports | Aquaculture | Canada | 1993 | 1 |
| Newfoundland curbs fish industry growth | Aquaculture | Canada | 1993 | -1 |
| SA THE WAY AHEAD A Special Series Boom times on way for;'farmers' | Aquaculture | Australia | 1993 | 1 |
| BANKING ON MILLIONS OF CLAMS Production goes high-tech in S.C. | Aquaculture | USA | 1993 | 1 |
| FISH FARMERS FACE BARRIERS | Aquaculture | Australia | 1992 | -1 |
| Fishing Lines: Smoking out giant salmon | Aquaculture | United Kingdom | 1992 | 0 |
| WHAT'S THE CATCH?;Aquaculture & Drugs: Wondering How Much Is Too Much | Aquaculture | USA | 1992 | -1 |
| Fishing Lines: Missing trout mystery | Aquaculture | United Kingdom | 1992 | 0 |
| Ottawa expect fish farming to boom over next decade Aquaculture now worth $100-million, official says | Aquaculture | Canada | 1990 | 1 |
| AQUACULTURE A BOOM INDUSTRY | Aquaculture | Australia | 1990 | 1 |
| GOVT ACCUSED OF WHITEWASH | Aquaculture | Australia | 1990 | -1 |
| AQUACULTURE CENTRE'S FIRST STAGE TO OPEN | Aquaculture | Australia | 1990 | 0 |
| Maryland Optimistic About Aquaculture Industry;New Law to Provide Benefits to Fish Farmers | Aquaculture | USA | 1990 | 1 |
| Aquaculture farms net $16 million sea harvest | Aquaculture | Canada | 1990 | 1 |
| NEW JERSEY JOURNAL;AQUACULTURE AND THE FUTURE | Aquaculture | USA | 1990 | 0 |
| UN OPTIMISTIC ON AQUACULTURE | Aquaculture | USA | 1990 | 1 |
| Fish on the Hoof;Aquaculture: Wave Of the Future? | Aquaculture | USA | 1990 | 1 |
| Action to build underwater harvests urged | Aquaculture | Canada | 1984 | 1 |
| Aquaculture: wave of the future rocks traditional fishery | Aquaculture | Canada | 1984 | 0 |
| Prawns a growing business in Port | Aquaculture | Australia | 2015 | 1 |
| Speech by SMS Desmond Lee at International Aquaculture Singapore Conference | Aquaculture | Singapore | 2015 | 0 |
| List of Fisheries for 2016 | Aquaculture | United States | 2015 | 0 |
| FEDERAL REGISTER: List of Fisheries for 2016 | Aquaculture | United States | 2015 | 0 |
| JCU bid for Ross Creek marine hub | Aquaculture | Australia | 2015 | 0 |
| SL's economy grows 6.7% in 2Q | Aquaculture | Sri Lanka | 2015 | 0 |
| Sri Lanka economy grew 6.7-pct in Q2 2015 | Aquaculture | Sri Lanka | 2015 | 0 |
| Media to descend on Cairns | Aquaculture | Australia | 2015 | 0 |
| Washington: King, National Park Service Officially Open Schoodic Woods Campground | Aquaculture | United States | 2015 | 0 |
| FEDERAL REGISTER: Endangered and Threatened Species: Final Rulemaking To Revise Critical Habitat for Hawaiian Monk Seals | Aquaculture | United States | 2015 | 0 |
| Whole fish approach \| Plant would process the entire fish to produce fish chow, fish oil - maybe even fertilizer. | Aquaculture | United States | 2015 | 0 |
| Spain challenges subsidy on Turkish sea products, exporters upset | Aquaculture | Turkey | 2015 | -1 |
| Coastal resources face threat | Aquaculture | Australia | 2015 | -1 |
| Something fishy is netting a profit in schoolgirl's life Youngster's idea turns into a thriving | Aquaculture | South Africa | 2015 | 1 |
| Small fry at first maybe, but teen's fish farm's growing; Tilapia waste feeds the hydroponic vegetables that float her business in an innovative recycling system | Aquaculture | South Africa | 2015 | 1 |
| Shrimp Export up by 56 Per Cent in State | Aquaculture | India | 2015 | 1 |
| Environmental Impact Statements; Availability, etc.; Weekly Receipts | Aquaculture | United States | 2015 | 0 |
| Don't come raw prawn, fraud told over $19m tax bill | Aquaculture | Australia | 2015 | -1 |
| Hayleys Agriculture wins AREA Award in Social Empowerment | Aquaculture | Sri Lanka | 2015 | 0 |
| Washington: S. Rept. 114-66 - DEPARTMENTS OF COMMERCE AND JUSTICE, AND SCIENCE, AND RELATED AGENCIES APPROPRIATIONS BILL, 2016 | Aquaculture | United States | 2015 | 0 |
| FEDERAL REGISTER: Spring 2015 Semiannual Agenda of Regulations | Aquaculture | United States | 2015 | 0 |
| More aquaculture challenges | Aquaculture | New Zealand | 2015 | -1 |
| Coastal areas ecosystem must for blue economy | Aquaculture | Bangladesh | 2015 | 0 |
| B.C. salmon farm appeals Federal Court order | Aquaculture | Canada | 2015 | -1 |
| Salmon farm appeals Federal Court ruling | Aquaculture | Canada | 2015 | -1 |
| New twist in controversial fish farm plan | Aquaculture | South Africa | 2015 | -1 |
| SA is open for business as a smart investment | Aquaculture | South Africa | 2015 | 1 |
| US Contract Notice: Department of Agriculture Issues Solicitation for Astaxathin & Canthazanthin Analysis"" | Aquaculture | United States | 2015 | 0 |
| Marine teachers go back to fishy school | Aquaculture | Australia | 2015 | 0 |
| Alaska: Introduction to Shellfish Aquaculture - Module 2 | Aquaculture | United States | 2015 | 0 |
| Centre not issuing funds for Sindh marine products export | Aquaculture | Pakistan | 2015 | 0 |
| EU move to boost PH fishing targets | Aquaculture | Philippines | 2015 | 0 |
| Lifting of 'yellow card' to generate $300-M fishery exports to EU | Aquaculture | Philippines | 2015 | 1 |
| Rep. Huffman Introduces Fishing Economy Improvement Act | Aquaculture | United States | 2015 | 1 |
| Costa-Pierce Talks on Ocean Food Systems at Harvard | Aquaculture | United States | 2015 | 0 |
| Rs 107-cr seafood park in Deras | Aquaculture | India | 2015 | 0 |
| Super diet promises a prawn bounty | Aquaculture | Australia | 2015 | 1 |
| Luncheon lives up to the hype | Aquaculture | Australia | 2015 | 0 |
| Real cost of injury in the farm sector | Aquaculture | Australia | 2015 | -1 |
| Colleges asked to push practical skills | Aquaculture | China | 2015 | 0 |
| Fish farmers restock as water quality appears improved; Some move their stocks back into open waters after recent fish deaths | Aquaculture | Singapore | 2015 | -1 |
| injury on farms massive; Time lost | Aquaculture | Canada | 2015 | -1 |
| Asian touch to salmon; Easy to cook and packed with nutrients, roasted salmon fillet is a quick favourite | Aquaculture | Singapore | 2015 | 1 |
| Prahok put to the quality test | Aquaculture | Cambodia | 2015 | 0 |
| Mass fish deaths hit Changi farmers hard | Aquaculture | Thailand | 2015 | -1 |
| Mass fish deaths overnight hit Changi farmers hard | Aquaculture | Singapore | 2015 | -1 |
| Washington: Endangered and Threatened Species; Critical Habitat for Endangered North Atlantic Right Whale | Aquaculture | United States | 2015 | 0 |
| Arizona: Endangered and Threatened Species: Critical Habitat for Endangered North Atlantic Right Whale | Aquaculture | United States | 2015 | 0 |
| Following fish | Aquaculture | Pakistan | 2015 | 0 |
| Corporate: Barramundi Asia plans global expansion, backed by A*STAR and local entrepreneur | Aquaculture | Singapore | 2015 | 1 |
| Pakistan: Benefits of tilapia farming highlighted | Aquaculture | Pakistan | 2015 | 1 |
| Benefits of tilapia farming highlighted | Aquaculture | Pakistan | 2015 | 1 |
| Johor eyes position as hub for marine studies | Aquaculture | Malaysia | 2015 | 0 |
| 'Marine Pollution Posing Challenge To Fish Trade, Human Health' | Aquaculture | India | 2015 | -1 |
| New Hampshire: N.H. Marine Rule Changes for 2015 | Aquaculture | United States | 2015 | 0 |
| Marine pollution posing challenge to fish trade, human health: Experts | Aquaculture | India | 2015 | -1 |
| List of Fisheries for 2015 | Aquaculture | United States | 2014 | 0 |
| Washington: List of Fisheries for 2015 | Aquaculture | United States | 2014 | 0 |
| Washington: Fall 2014 Semiannual Agenda of Regulations | Aquaculture | United States | 2014 | 0 |
| Washington: Introduction to the Unified Agenda of Federal Regulatory and Deregulatory Actions | Aquaculture | United States | 2014 | 0 |
| China invites PHL in world's first fish 'spot market' | Aquaculture | Philippines | 2014 | 0 |
| Lifeline for offshore marine research unit; St John's Island lab, which was beset by rising operating costs, finds a way to stay open | Aquaculture | Singapore | 2014 | 0 |
| Fuzhou Special: Fuzhou set to build a 21st Century Maritime Silk Road hub | Aquaculture | China | 2014 | 0 |
| 'Blue Revolution' to Revitalise India's Fisheries Sector | Aquaculture | India | 2014 | 0 |
| Washington: Assistant Professor of Risk Communication | Aquaculture | United States | 2014 | 0 |
| You may have heard of a group of fast food restaurants | Aquaculture | Australia | 2014 | 0 |
| Halloween gives me the creeps | Aquaculture | Australia | 2014 | 0 |
| Fish farmers not keen on waste collection services | Aquaculture | Singapore | 2014 | -1 |
| Rising tide | Aquaculture | Thailand | 2014 | -1 |
| Rising tide of litter on S'pore's shorelines; One group nets 14,400kg worth so far this year | Aquaculture | Singapore | 2014 | -1 |
| Hot spell takes a toll on coastal fish farms | Aquaculture | Singapore | 2014 | -1 |
| Wainui: A marvellous spot for spat | Aquaculture | New Zealand | 2014 | 1 |
| Students ride wave of support | Aquaculture | Australia | 2014 | 1 |
| SA's past prudence will drive economic renewal | Aquaculture | South Africa | 2014 | 1 |
| Beverage, food firms boost sales, partnership | Aquaculture | Philippines | 2014 | 1 |
| List of Fisheries for 2015 | Aquaculture | United States | 2014 | 0 |
| EC looks to lead 'blue economy' | Aquaculture | South Africa | 2014 | 1 |
| Coega plan for aquafarming | Aquaculture | South Africa | 2014 | 1 |
| Pimp My Cause matches marketing pros with causes that need their help | Aquaculture | United States | 2014 | 0 |
| CBD on the world stage | Aquaculture | Australia | 2014 | 0 |
| MONTARA'S FORGOTTEN VICTIMS"" | Aquaculture | Australia | 2014 | -1 |
| Croc, beer, hat and prawn Queen Mary | Aquaculture | Australia | 2014 | 0 |
| State schedules second meeting about controversial Bagaduce aquaculture | Aquaculture | United States | 2014 | -1 |
| A really \|good oil \|and gas find means jobs | Aquaculture | South Africa | 2014 | 1 |
| Confident that SA can beat global downturn | Aquaculture | South Africa | 2014 | 1 |
| Focusing on marine economy | Aquaculture | South Africa | 2014 | 1 |
| Mississippi: Presley: Agricultural Utility Sales Tax Exemption | Aquaculture | United States | 2014 | 0 |
| Plastic Waste Can Cause N2 Trillion Financial Damage Annually - Unep | Aquaculture | Kenya | 2014 | -1 |
| Water babies; S.E.A. Aquarium at Resorts World Sentosa has bred more than 2,000 marine creatures, including sharks | Aquaculture | Singapore | 2014 | 0 |
| Finding fun in northern delights | Aquaculture | Australia | 2014 | 1 |
| OAM awarded for service to prawn industry | Aquaculture | Australia | 2014 | 1 |
| Turning the tide of our ocean economy could net us (EURO)6.5bn; In a time of world recession, few products can boast the market growth that farmed salmon is experiencing, writes expert Jan Feenstra | Aquaculture | Ireland | 2014 | 1 |
| China sets up 10 bln yuan fund to support development of maritime Silk Road | Aquaculture | China | 2014 | 1 |
| Farmers still reeling from losses after fish deaths | Aquaculture | Singapore | 2014 | -1 |
| Fund to boost China's maritime Silk Road development | Aquaculture | China | 2014 | 1 |
| Poor Sanitation Causes Animal Infertility [column] | Aquaculture | Namibia | 2014 | -1 |
| Seafood canner takes in strategic investor | Aquaculture | South America | 2014 | 0 |
| No 'shellshock' as prawns weather Ita | Aquaculture | Australia | 2014 | 0 |
| Welcome to the world's largest garbage dump | Aquaculture | China | 2014 | -1 |
| Eyes wide open tour | Aquaculture | Australia | 2014 | 0 |
| Improving feed sustainability for marine aquaculture in Vietnam and Australia | Aquaculture | Australia | 2014 | 1 |
| Decline in seafood export | Aquaculture | United Arab Emirates | 2014 | -1 |
| Marine Harvest sells Scottish assets to Cook Acquaculture | Aquaculture | Scotland | 2014 | 0 |
| Prawn star hits gold | Aquaculture | Australia | 2014 | 1 |
| Burtynsky's big ideas translate into big pictures; A Terrible Beauty, on display at VAG, captures humanity's impact on the Earth through the lens of aerial photographs | Aquaculture | Canada | 2014 | -1 |
| Best Free Reference | Aquaculture | United States | 2014 | 0 |
| Cheaper sensor to check water quality; Device being developed in S'pore can help local fish farmers | Aquaculture | Singapore | 2014 | 0 |
| Fish deaths a double whammy; Losses add to woes for farmer facing competition from Malaysia | Aquaculture | Singapore | 2014 | -1 |
| 160 tonnes of dead fish found in farms along Johor Straits | Aquaculture | Singapore | 2014 | -1 |
| Fish producer waits for health certificate to export | Aquaculture | South Africa | 2014 | 0 |
| Many things unchanged on quiet St John's; Allure of hilly island continues to reel in a steady stream of nature lovers | Aquaculture | Singapore | 2014 | 0 |
| Shots in the dark; prawns | Aquaculture | Australia | 2014 | 0 |
| GAFRD: Egypt does not produce healthy fish | Aquaculture | Egypt | 2014 | -1 |
| Traders: Better prospects in second half of year | Aquaculture | Philippines | 2014 | 1 |
| Marine insurance firms announce merger plans | Aquaculture | United Kingdom | 2013 | 0 |
| ICMIF: Member News: Proposed Merger for Sunderland Marine | Aquaculture | India | 2013 | 0 |
| Merger deal would create one of world's largest marine insurance operations | Aquaculture | Canada | 2013 | 0 |
| List of Fisheries for 2014 | Aquaculture | United States | 2013 | 0 |
| A journey of history and treats | Aquaculture | Canada | 2013 | 0 |
| EU and Ministry of International Cooperation sign agreement | Aquaculture | Egypt | 2013 | 0 |
| Festive celebrations mean the end of the year looms | Aquaculture | Australia | 2013 | 0 |
| Tull star says: I'm not too old to rock'n'roll | Aquaculture | United Kingdom | 2013 | 0 |
| Guests spin good yarns | Aquaculture | Canada | 2013 | 0 |
| Next trip, try the prawns | Aquaculture | Australia | 2013 | 1 |
| Namibians Must Build Partnerships With Chinese | Aquaculture | Namibia | 2013 | 0 |
| Look back in time to wrap up busy year | Aquaculture | Australia | 2013 | 0 |
| THANKS TO TANKS | Aquaculture | South Africa | 2013 | 1 |
| People's Government of Jilin Province: Sino Russian Economic and Trade Cooperation Project Matchmaking Was Held | Aquaculture | China | 2013 | 0 |
| Europe, Asia recovery to boost export growth | Aquaculture | Philippines | 2013 | 1 |
| Researchers mastering the challenge of our warming oceans | Aquaculture | Australia | 2013 | 1 |
| Dalian Municipal Government: First Wind Index Marine Aquaculture Insurance Initiated | Aquaculture | China | 2013 | 1 |
| US Patent granted to Bradley Innovation Group, LLC (Indiana) on August 13 titled as Process and system for growing crustaceans and other fish"" | Aquaculture | United States | 2013 | 1 |
| Fish studies expand; Research boost a win for marine life | Aquaculture | Australia | 2013 | 1 |
| Dive and fishing business will have you hooked | Aquaculture | New Zealand | 2013 | 1 |
| Seals of disapproval | Aquaculture | Australia | 2013 | -1 |
| Oceans of praise for excellence | Aquaculture | Australia | 2013 | 1 |
| MTI awards $1.6 million to 27 innovative Maine companies | Aquaculture | United States | 2013 | 1 |
| Winemaker banks on a new vintage; DRINKS | Aquaculture | Australia | 2013 | 0 |
| Top prawn shows its breeding | Aquaculture | Australia | 2013 | 1 |
| Oceanpick to donate safety gear to Trinco fishermen | Aquaculture | Sri Lanka | 2013 | 0 |
| The secret power of seaweed; How award-winning research into an old wives' tale is helping psoriasis and eczema sufferers. By Stephanie Bell | Aquaculture | Ireland | 2013 | 1 |
| Trio of awards for MHS | Aquaculture | Scotland | 2013 | 1 |
| Shellfish chief recognised | Aquaculture | Scotland | 2013 | 1 |
| College of Fisheries project team develops solar-biomass hybrid dryer | Aquaculture | India | 2013 | 0 |
| Bid to breed 'iconic' NZ seahorses | Aquaculture | New Zealand | 2013 | 0 |
| DEAKIN University's new Professor of Marine Science | Aquaculture | Australia | 2013 | 0 |
| Finalists vie for awards after record entries received | Aquaculture | Scotland | 2013 | 0 |
| Cooke Aquaculture to pay $490,000 after illegal pesticides kill lobsters in Canada | Aquaculture | United States | 2013 | -1 |
| RAJIV GANDHI CENTRE FOR AQUACULTURE PRODUCES SPECIFIC PATHOGEN FREE L. VANNAMEI BROODSTOCK FOR 1ST TIME IN INDIA | Aquaculture | India | 2013 | 1 |
| NARA to develop aquaculture | Aquaculture | Sri Lanka | 2013 | 1 |
| DEAKIN University is hoping to develop stronger relationships | Aquaculture | Australia | 2013 | 0 |
| We do like to adapt beside the seaside; COMMERCIAL REPORT: MORISONS LLP City legal team goes rural with success, finds Rick Wilson | Aquaculture | United Kingdom | 2013 | 0 |
| Prawn lunch draws big crowd | Aquaculture | Australia | 2013 | 1 |
| Haulier clinches fish feed contract | Aquaculture | Scotland | 2013 | 0 |
| Deadline for Scottish awards | Aquaculture | Scotland | 2013 | 0 |
| City 'summit' eyes waterfront growth | Aquaculture | United States | 2013 | 0 |
| Seal scarer device' backed by MSPs | Aquaculture | Scotland | 2013 | 1 |
| Pakistan: Banned nets cause severe harm to marine fisheries | Aquaculture | Pakistan | 2013 | -1 |
| Shellfishermen trying to cope with sea ducks | Aquaculture | United States | 2012 | 0 |
| Concerns over size of proposed new salmon farm in Galway Bay | Aquaculture | Ireland | 2012 | -1 |
| This Atlantic salt has a sweet future | Aquaculture | Ireland | 2012 | 1 |
| Sustainability at the top level | Aquaculture | Australia | 2012 | 1 |
| Marine worker to set sail for US study | Aquaculture | United Kingdom | 2012 | 0 |
| Salmon report shows the effect of politics dictating policy | Aquaculture | Canada | 2012 | 0 |
| A vision takes root at sculpture garden | Aquaculture | United States | 2012 | 0 |
| FORMER Governor-General Michael Jeffery (pictured) has be | Aquaculture | Australia | 2012 | 0 |
| Herbst congratulated | Aquaculture | Australia | 2012 | 0 |
| Fish waste study on chemicals | Aquaculture | United Kingdom | 2012 | -1 |
| Sea bird guides research vessel | Aquaculture | Australia | 2012 | 0 |
| UniÃƒ+s growth spurt | Aquaculture | Australia | 2012 | 0 |
| New Mexico, agricultural history to be celebrated at NMSU's Leyendecker science center | Aquaculture | United States | 2012 | 0 |
| Seatrout Population Enhancement Cooperative releasing thousands of seatrout fingerlings in St. Louis Bay | Aquaculture | United States | 2012 | 1 |
| The Central People's Government of the People's Republic of China: More Relief Funding as Tropical Storm Nears | Aquaculture | China | 2012 | 0 |
| Marine course popular | Aquaculture | Australia | 2012 | 0 |
| Selling the prawn farm | Aquaculture | Australia | 2012 | 0 |
| Indonesia - Namibia - Growing Together [opinion] | Aquaculture | Australia | 2012 | 0 |
| Marine park gets ready for opening; Oceanarium's construction complete; animals arriving here daily from overseas | Aquaculture | Singapore | 2012 | 0 |
| Butterworth's ANC stalwart fought for the poor | Aquaculture | South Africa | 2012 | 0 |
| Students get their hands wet | Aquaculture | Australia | 2012 | 0 |
| NARA sets action plan to develop fish industry | Aquaculture | Sri Lanka | 2012 | 1 |
| Fisheries Could Stimulate Economy | Aquaculture | Namibia | 2012 | 1 |
| Land distribution target bared for E. Visayas | Aquaculture | Philippines | 2012 | 0 |
| Fish farming claims are disputed by IFA | Aquaculture | Ireland | 2012 | -1 |
| Kalimbeza Fish Farm Thriving | Aquaculture | Namibia | 2012 | 1 |
| Years of volunteering not unnoticed | Aquaculture | United States | 2012 | 0 |
| Alternative energy project under way | Aquaculture | United States | 2012 | 0 |
| Mariculture is not easy | Aquaculture | Australia | 2012 | -1 |
| Factory and land | Aquaculture | Australia | 2012 | 0 |
| Prawn farmer at last prepared to enjoy a very happy Christmas | Aquaculture | Australia | 2012 | 1 |
| New report on mariculture urges cautious approach | Aquaculture | Australia | 2012 | -1 |
| NETWORKING PRAWN LUNCH | Aquaculture | Australia | 2012 | 0 |
| Bill could add Â£20m to costs | Aquaculture | Scotland | 2012 | 0 |
| Local lobbyists swimming against political tide | Aquaculture | Ireland | 2012 | -1 |
| Fusion Marine Awarded Â£500,000 for Tilapia Farming | Aquaculture | Zambia | 2012 | 1 |
| Prawns hook big catch | Aquaculture | Australia | 2012 | 1 |
| Researchers learn from our local industries | Aquaculture | Australia | 2012 | 0 |
| COMMON FISHERIES POLICY : COMMISSION SPELLS OUT SOCIAL DIMENSION OF CFP REFORM | Aquaculture | United Kingdom | 2012 | 0 |
| Walvis Bay Is Mariculture Oyster | Aquaculture | Namibia | 2012 | 0 |
| Scientists to showcase new subsea sensor system | Aquaculture | Scotland | 2012 | 0 |
| Tilapia King of Hardap Eco Fishing Farm | Aquaculture | Namibia | 2012 | 1 |
| Quick bites | Aquaculture | Australia | 2012 | 0 |
| people true north North Queensland characters: Nell Braley Spicing up island markets | Aquaculture | Australia | 2012 | 0 |
| Denmark: Innovative Multi-purpose off-shore platforms: planning, Design and operation (MERMAID) | Aquaculture | Denmark | 2012 | 1 |
| Rising development increases environmental woes | Aquaculture | Middle East | 2012 | -1 |
| Prawns galore, with one catch | Aquaculture | Australia | 2011 | 0 |
| 'Green tape' delays prawn industry growth | Aquaculture | Australia | 2011 | -1 |
| Norway: Innovative Rearing and Stunning of Farmed Turbot and Sole to Meet Future Challenges Regarding Quality of Production and Animal Welfare (MAXIMUS) | Aquaculture | United Kingdom | 2011 | 0 |
| POUNDS 10K FINE TO PROTECT FISH | Aquaculture | United Kingdom | 2011 | -1 |
| Fine for flouting fish rules may rise | Aquaculture | Scotland | 2011 | -1 |
| Anglers and fish farmers may face Â£10,000 fines under new legislation | Aquaculture | United Kingdom | 2011 | -1 |
| Fisheries, a dynamic export | Aquaculture | Sri Lanka | 2011 | 0 |
| SEAFOOD farmers and scientists are battling | Aquaculture | Australia | 2011 | -1 |
| SA's bold energy future | Aquaculture | South Africa | 2011 | 0 |
| Coral farmer to teach Unity College students how to grow delicate saltwater organism | Aquaculture | United States | 2011 | 1 |
| Plan hatched, but fish and shrimps didn't | Aquaculture | Pakistan | 2011 | -1 |
| Fish farmers unite to be heard; Group of 38 banking on numbers to bring down costs, raise industry issues to govt | Aquaculture | Singapore | 2011 | 1 |
| Fish farm co-op under probe; Registry of Cooperative Societies acting on feedback about the way co-op operates | Aquaculture | Singapore | 2011 | -1 |
| Award-winning fish farm in pollution row | Aquaculture | Scotland | 2011 | -1 |
| Malaysia has potential to be global leader in arowana production | Aquaculture | Malaysia | 2011 | 1 |
| Fish farm co-op fails to reel in big numbers; Original target did not factor in infrastructure and manpower issues | Aquaculture | Singapore | 2011 | -1 |
| Brazil: Southern oceans education and development | Aquaculture | Mozambique | 2011 | 0 |
| Deakin's $5m boost | Aquaculture | Australia | 2011 | 1 |
| Vision for 2000 full-time uni students on campus | Aquaculture | Australia | 2011 | 0 |
| Deakin University will invest $5 million | Aquaculture | Australia | 2011 | 1 |
| Week Ahead | Aquaculture | United States | 2011 | 0 |
| Thank your public-sector unions | Aquaculture | Canada | 2011 | 0 |
| Industry worth $300m | Aquaculture | Australia | 2011 | 0 |
| Biofouling reduction in marine aquaculture | Aquaculture | United Kingdom | 2011 | 1 |
| The Blue Revolution - a two part popular science tv-documentary on marine aquaculture | Aquaculture | Norway | 2011 | 1 |
| Due South; When you are short of time but want a beach vacation, look no farther than the Southern Islands of Singapore, a quick boat ride away | Aquaculture | Singapore | 2011 | 0 |
| Funding boost for fish fry, rice research; Projects to develop bigger fish, superior rice are among three to get total of up to $30m | Aquaculture | Singapore | 2011 | 1 |
| New England in Brief | Aquaculture | United States | 2011 | 0 |
| Network for the development of phage therapy in aquaculture | Aquaculture | Greece | 2011 | 1 |
| Virus that struck Chilean fish farming tied to salmon eggs from Norway; More than $2 billion lost and 26,000 workers laid off after 2007 outbreak | Aquaculture | Chile | 2011 | -1 |
| Thai traders, SMEDA discuss joint ventures in SMEs | Aquaculture | Pakistan | 2011 | 0 |
| Pakistan: Thai traders, SMEDA discuss joint ventures in SMEs | Aquaculture | Pakistan | 2011 | 0 |
| Firms fined after catalogue of errors led to deaths | Aquaculture | Scotland | 2011 | -1 |
| Anglers reel in free bumper catch; Sea bass that escaped from govt fish farm likely ended up on dinner tables | Aquaculture | Singapore | 2011 | 1 |
| Indonesian students' aquatic adventure | Aquaculture | Australia | 2011 | 0 |
| Indonesian students pay a visit | Aquaculture | Australia | 2011 | 0 |
| Brazil: Southern oceans education and development Project | Aquaculture | Brazil | 2011 | 0 |
| Coega earmarked for finfish venture | Aquaculture | South Africa | 2011 | 1 |
| Brazil: Southern oceans education and development | Aquaculture | Mozambique | 2011 | 0 |
| 'Marine aquaculture vital for Oman' | Aquaculture | Oman | 2011 | 1 |
| Finalists for Crown honours revealed | Aquaculture | Scotland | 2011 | 0 |
| Aquarium fish becoming a problem in Atlantic waters | Aquaculture | United States | 2011 | -1 |
| Prawns glorious prawns | Aquaculture | Australia | 2011 | 1 |
| TUCKING INTO PRAWN FEST | Aquaculture | Australia | 2011 | 0 |
| Breakthrough in tuna farming | Aquaculture | Australia | 2011 | 1 |
| First fish in sea for tuna breeder | Aquaculture | Australia | 2011 | 1 |
| Book early for annual prawn lunch | Aquaculture | Australia | 2011 | 0 |
| Fish farms aim to net huge harvest; Co-op will help them get consistent supplies of fish fry and feed | Aquaculture | Singapore | 2011 | 1 |
| A promising partnership of plants and pompano | Aquaculture | United States | 2011 | 0 |
| Emergency watch system - now at sea; Initiative launched by 18 fish farmers aims to provide quick, efficient help | Aquaculture | Singapore | 2011 | 1 |
| Africa fish-farm success | Aquaculture | Africa | 2011 | 1 |
| Business | Aquaculture | Australia | 2010 | 0 |
| Â£15m Aberdeen marine research centre hailed as world class | Aquaculture | Scotland | 2010 | 1 |
| Seize the day, harness the sea | Aquaculture | Norway | 2010 | 1 |
| Confusion on national body's role | Aquaculture | New Zealand | 2010 | 0 |
| All food production affects the environment; Closed-containment salmon farms not the panacea they are said to be | Aquaculture | Canada | 2010 | -1 |
| AIMS tour guides share reef knowledge | Aquaculture | Australia | 2010 | 0 |
| Fish farmers reeling in researchers; High-tech methods sought to protect the environment and boost harvests | Aquaculture | Australia | 2010 | 1 |
| High hopes of fish farmers risk being dead in the water | Aquaculture | Ireland | 2010 | -1 |
| Master prawn on menu | Aquaculture | Australia | 2010 | 0 |
| Fisheries Must Deal With Threats, Opportunities | Aquaculture | Africa | 2010 | -1 |
| Man proposes 50-acre Trenton oyster farm | Aquaculture | United States | 2010 | 0 |
| Thai traders, SMEDA discuss joint ventures in SMEs | Aquaculture | Pakistan | 2010 | 0 |
| 50,000 fingerlings released into Qatari waters | Aquaculture | Qatar | 2010 | 0 |
| Australian scientists develop the perfect prawn | Aquaculture | Australia | 2010 | 1 |
| Small-scale fisherman seeking bigger benefits from minister | Aquaculture | South Africa | 2010 | 0 |
| Could this be the ultimate prawn star? | Aquaculture | Australia | 2010 | 1 |
| Alliance Tuna to start due diligence on Hiep Thanh | Aquaculture | Viet Nam | 2010 | 0 |
| Australian super prawns are born | Aquaculture | Australia | 2010 | 1 |
| Prawn breeding breakthrough | Aquaculture | Australia | 2010 | 1 |
| AQUACULTURE Success for CSIRO breeding program The perfect prawn | Aquaculture | Australia | 2010 | 1 |
| Net Value: The state of Malaysian biotechnology | Aquaculture | Malaysia | 2010 | 0 |
| College open | Aquaculture | United Kingdom | 2010 | 0 |
| Youth in Print School catches six new fish | Aquaculture | Australia | 2010 | 0 |
| Among nation's best | Aquaculture | Australia | 2010 | 0 |
| Cambodian DPM calls on PM Lee and other leaders | Aquaculture | Singapore | 2010 | 0 |
| Trade deficit with S'pore has to be addressed: Cambodia | Aquaculture | Singapore | 2010 | 0 |
| Budget Speech | Aquaculture | India | 2010 | 0 |
| Buoyant Q4, full year for Oceanus; Key factors: fair value gains, higher sales, absence of goodwill write-off | Aquaculture | Singapore | 2010 | 1 |
| Meet the super sea bass; Farmed-in-Singapore barramundi makes a splash as an alternative to regular sea bass | Aquaculture | Australia | 2010 | 1 |
| Buying among directors up, selling remains low | Aquaculture | Singapore | 2010 | 0 |
| Fisheries in a flap | Aquaculture | South Africa | 2010 | 0 |
| Marine farms a risk for boaties | Aquaculture | New Zealand | 2010 | -1 |
| 200,000 fish in farms off Pasir Ris dead; Plankton bloom causes losses reaching hundreds of thousands of dollars | Aquaculture | Singapore | 2010 | -1 |
| Cost-effective, eco-friendly feed for marine farming | Aquaculture | Malaysia | 2009 | 1 |
| A quiet session ahead of year-end holidays | Aquaculture | Singapore | 2009 | 0 |
| Moving of government furniture all but complete; New departments, others split | Aquaculture | South Africa | 2009 | 0 |
| More S'pore farmed fish on menu; Republic's largest commercial fish farm a sign of things to come | Aquaculture | Singapore | 2009 | 1 |
| Bangladesh:Oceans would be able to feed growing world population in future | Aquaculture | Pakistan | 2009 | 1 |
| UNCW to pay more for facility | Aquaculture | United States | 2009 | 0 |
| Barr wins bid to build GBP11m fish study centre in Aberdeen | Aquaculture | Scotland | 2009 | 0 |
| Injured turtle released back into the wild | Aquaculture | Cyprus | 2009 | 1 |
| Heat is on for school project | Aquaculture | Australia | 2009 | 0 |
| Wanted: tough outdoor types up to challenge of extreme fish farming Staff will live offshore on 'iceberg' barges | Aquaculture | Scotland | 2009 | 0 |
| Borneo Aqua Harvest will continue to uptrend | Aquaculture | Malaysia | 2009 | 1 |
| Family split threatens success of abalone farm | Aquaculture | South Africa | 2009 | 0 |
| School challenge - ABC to PHD: Advertising feature | Aquaculture | Australia | 2009 | 0 |
| Youth In Print Experiments with science | Aquaculture | Australia | 2009 | 0 |
| Prawn secrets to be probed | Aquaculture | Australia | 2009 | -1 |
| Co-op gives local fish stocks a boost; Five fish farms here link up and 10 others keen to join cooperative | Aquaculture | Singapore | 2009 | 1 |
| Cambodia to get over 33m developmental aid from Japan | Aquaculture | Cambodia | 2009 | 1 |
| Director, institutional shareholder activity flat | Aquaculture | Singapore | 2009 | 0 |
| Evidence of fish farm risks to salmon mounts | Aquaculture | Canada | 2009 | -1 |
| Mississippi lawmakers approve funding | Aquaculture | United States | 2009 | 1 |
| Award for Loch Roag system | Aquaculture | Scotland | 2009 | 0 |
| Flat trading by directors; Buyback, shareholder activity also flat, but the week sees several significant filings by institutional shareholders, reports ROBERT HALILI | Aquaculture | Singapore | 2009 | 0 |
| Shetland success at annual aquaculture industry accolades | Aquaculture | Scotland | 2009 | 1 |
| Developer adamant to progress Trinity Wharf plan | Aquaculture | Ireland | 2009 | 0 |
| Farmers hail the raw prawn | Aquaculture | Australia | 2009 | 1 |
| Plague of 'jackets | Aquaculture | Australia | 2009 | -1 |
| Big V for Vino at Sydney show | Aquaculture | Australia | 2009 | 0 |
| open season | Aquaculture | Australia | 2009 | 0 |
| Major marine scientific expedition off Sabah | Aquaculture | Australia | 2009 | 0 |
| Sea's fruits are from the farm | Aquaculture | Australia | 2009 | 1 |
| M&F shows signs of catching governance bug | Aquaculture | South Africa | 2009 | -1 |
| Lawmakers hear about aquaculture; UNCW officials, fish farmers talk to new committee in Raleigh | Aquaculture | United States | 2009 | 0 |
| Prawn farmer in line for show's major award | Aquaculture | Australia | 2009 | 1 |
| Easter prawns priced too high - Seafood chief says shops not passing on savings | Aquaculture | Australia | 2009 | -1 |
| Abandoned turtle rescued | Aquaculture | Cyprus | 2009 | 1 |
| Fund manager may inject US $15m into Oceanus | Aquaculture | Singapore | 2009 | 1 |
| Va. agricultural exports soar 27 percent in 2008; The value of the state's crops rises to $2.22 billion as variety pays dividends | Aquaculture | United States | 2009 | 0 |
| Treating wild and farmed salmon equally | Aquaculture | Canada | 2009 | 1 |
| Huon tempts your appetite | Aquaculture | Australia | 2009 | 1 |
| Joint resource dev't pushed | Aquaculture | Philippines | 2009 | 1 |
| Minister unscrupulous and callous - perlemoen fishers | Aquaculture | South Africa | 2009 | -1 |
| 'government fails to deliver' | Aquaculture | South Africa | 2009 | -1 |
| Yellowtail ranching project to help fishermen could miss boat | Aquaculture | South Africa | 2009 | -1 |
| Â£300,000 expansion plans | Aquaculture | United Kingdom | 2009 | 1 |
| Fishermen hope for Gregg s help at Commerce post | Aquaculture | United States | 2009 | 0 |
| Seller thought zoo could handle cow nose rays; 'We assumed there was a level of expertise' | Aquaculture | Canada | 2009 | 0 |
| Gulf No Place For Fish Farms | Aquaculture | United States | 2009 | -1 |
| FISHING for FUNDS; FISH HATCHERY PLAN: Bid for stimulus cash could help Mote Marine grow | Aquaculture | United States | 2009 | 1 |
| EW plan gives aqua-farmers their chances | Aquaculture | New Zealand | 2008 | 1 |
| Johor to produce organic food | Aquaculture | Malaysia | 2008 | 0 |
| Abalone dilemma | Aquaculture | South Africa | 2008 | -1 |
| Big Splash; Some city-bred Singapore entrepreneurs have fallen hook , line and sinker for the simple life in kelongs | Aquaculture | Singapore | 2008 | 0 |
| B.C.'s salmon farmers given a free ride by our provincial 'regulators' | Aquaculture | Canada | 2008 | -1 |
| R.I. Tree Council to host Beijing university professor | Aquaculture | United States | 2008 | 0 |
| GOLD COAST HONOURS 2008 All our finalists | Aquaculture | Australia | 2008 | 0 |
| GOLD COAST HONOURS NOMINEES | Aquaculture | Australia | 2008 | 0 |
| Prawns sweeter than sugarcane | Aquaculture | Australia | 2008 | 1 |
| Special panel studying nursing shortage in R.I. | Aquaculture | United States | 2008 | 0 |
| Aquaculture facilities get funds to expand; Beals, Franklin sites of research centers | Aquaculture | United States | 2008 | 1 |
| Anglers condemn debris left behind by fish farms giant Marine Harvest | Aquaculture | Scotland | 2008 | -1 |
| Tiger prawns earn stripes | Aquaculture | Australia | 2008 | 1 |
| From the kelong to your table - the cobia; Premium fish used in yusheng now farmed here as part of AVA's project to cut reliance on imports | Aquaculture | Singapore | 2008 | 1 |
| Affordable skin-on fillets that'll stand up to grilling | Aquaculture | United States | 2008 | 1 |
| Fish farms offer hope of cheap easy catch | Aquaculture | Australia | 2008 | 1 |
| Revolutionary Bio-Energy From Algae | Aquaculture | Korea | 2008 | 1 |
| Pressure groups say poor are getting a raw deal from government's abalone strategy | Aquaculture | South Africa | 2008 | -1 |
| Climate change to impact on fisheries | Aquaculture | Scotland | 2008 | -1 |
| Oceanus expansion plans may lead to dividend payouts | Aquaculture | Singapore | 2008 | 1 |
| Fish escape prompts new calls for change; Critics contend only closed-containment will repair problem | Aquaculture | Canada | 2008 | -1 |
| Joy: Ben aims to do swimmingly in dream job | Aquaculture | United Kingdom | 2008 | 0 |
| Marine college student lands dream job with resort aquarium; A cornwall College student has landed his dream job - before completing his own degree. | Aquaculture | United Kingdom | 2008 | 0 |
| Environmental program open to public | Aquaculture | United States | 2008 | 0 |
| Oceanus makes Catalist debut today | Aquaculture | Singapore | 2008 | 0 |
| SEAFOOD DEAL $100m U.S. export bonanza Eyre abalone coup | Aquaculture | Australia | 2008 | 1 |
| Post-war pact to the rescue of coastguard | Aquaculture | Ireland | 2008 | 0 |
| Luncheon appealing | Aquaculture | Australia | 2008 | 0 |
| Lunch draws crowd | Aquaculture | Australia | 2008 | 0 |
| Career Beacon.com job listings | Aquaculture | Canada | 2008 | 0 |
| Marine farm plans sunk | Aquaculture | New Zealand | 2008 | -1 |
| Group critical of salmon farming impact on Bay; Conservation group say more stringent regulations, policies needed to protect Bay of Fundy | Aquaculture | Canada | 2008 | -1 |
| Tagging the big fish | Aquaculture | Australia | 2008 | 0 |
| Q&A with Dr. Martha Dunagin Saunders, U of Southern Mississippi president | Aquaculture | United States | 2008 | 0 |
| Prawn lunch | Aquaculture | Australia | 2008 | 0 |
| Under microscope | Aquaculture | Australia | 2008 | 0 |
| Black fishing investors face stormy waters | Aquaculture | South Africa | 2008 | -1 |
| Adventurer found success on land and sea | Aquaculture | United States | 2008 | 1 |
| Fishing | Aquaculture | South Africa | 2008 | 0 |
| Danger signal - alien invader threatens salmon, trout stocks | Aquaculture | United Kingdom | 2007 | -1 |
| UNLIKELY ALLIES TEAM UP AGAINST FISH FARMING AT HEARING | Aquaculture | United States | 2007 | -1 |
| UNLIKELY ALLIES OPPOSE GULF FISH FARMING | Aquaculture | United States | 2007 | -1 |
| Bright sparks lock onto 'Genera onto 'Generation Y'; Two new universities are set to feel the benefit of a scheme to harness the talents of bright young things in business, says Stephen Naysmith | Aquaculture | Scotland | 2007 | 0 |
| Support for marine management plan | Aquaculture | New Zealand | 2007 | 1 |
| Philippines poised to revisit expired fishing pacts with Indonesia | Aquaculture | Philippines | 2007 | 0 |
| Sea projects to soften ban on abalone | Aquaculture | South Africa | 2007 | 1 |
| Government to inject R100m into marine fish farms | Aquaculture | South Africa | 2007 | 1 |
| Ban postponed - but abalone court bid set to go ahead | Aquaculture | South Africa | 2007 | -1 |
| 'real issue is perlemoen poachers' | Aquaculture | South Africa | 2007 | -1 |
| A chance to mussel in on aquaculture; Shellfish farming company's empowerment project offers nine fishermen a fresh start | Aquaculture | South Africa | 2007 | 1 |
| Fishing pacts with Pacific nations sought | Aquaculture | Philippines | 2007 | 0 |
| Fingerlings to finger-licking good | Aquaculture | Singapore | 2007 | 1 |
| S'pore's very own super sea bass; Hatched in AVA's research tanks, the fast-growing, hardy fry were fattened on an offshore farm in Riau | Aquaculture | Singapore | 2007 | 1 |
| FAO SUPPORTS REHABILITATION OF AGRICULTURAL, LIVESTOCK, FISHERIES AND LIVELIHOODS OF \ CYCLONE AFFECTED COMMUNITIES OF BALOCHISTAN | Aquaculture | Pakistan | 2007 | 1 |
| B.c. Salmon industry in rough water; Agriculture; Skilled workers cut bait rather than face the 'politics' | Aquaculture | Australia | 2007 | -1 |
| Show gets bigger | Aquaculture | Australia | 2007 | 0 |
| Hotel's student contract | Aquaculture | United Kingdom | 2007 | 0 |
| Highlights of Northern Corridor Economic Region (NCER) | Aquaculture | Malaysia | 2007 | 0 |
| Popular course | Aquaculture | United Kingdom | 2007 | 0 |
| Imported raw prawns in risk to marine life | Aquaculture | Australia | 2007 | -1 |
| Salt study boon for abalone | Aquaculture | Australia | 2007 | 1 |
| UPSHOT; Chinese economic invasion | Aquaculture | China | 2007 | 0 |
| Tiny freshwater creatures can grow into tasty delights far from any ocean; Raising shrimp is big business in Ohio ponds | Aquaculture | United States | 2007 | 1 |
| MOTE'S RACE AGAINST THE CLOCK; Rush is on to complete rebuilding of fire-damaged sturgeon facility | Aquaculture | United States | 2007 | 0 |
| Anti fish farm ads take aim at grocery chain | Aquaculture | Canada | 2007 | -1 |
| A barefoot boy's; golden harvest | Aquaculture | Australia | 2007 | 1 |
| Seaweed farming points the way for food production | Aquaculture | South Africa | 2007 | 1 |
| Kleinschmidt honored for AquaPod project | Aquaculture | United States | 2007 | 1 |
| Courses on offer | Aquaculture | United Kingdom | 2007 | 1 |
| Climate change threatens tiny krill, leaving Antarctic ecosystem at risk | Aquaculture | United States | 2007 | -1 |
| US fishes for subsidy support | Aquaculture | South Africa | 2007 | 0 |
| Fish farm group will trawl for youngsters | Aquaculture | Australia | 2007 | 0 |
| Fishy find: Children discover unusual visitor | Aquaculture | United Kingdom | 2007 | 0 |
| Warm-water puffer fish found on beach | Aquaculture | United Kingdom | 2007 | 0 |
| Aquaculture growing dramatically here and worldwide | Aquaculture | United States | 2007 | 1 |
| Stop foreign poaching | Aquaculture | South Africa | 2007 | 0 |
| Something fishy | Aquaculture | South Africa | 2007 | -1 |
| Small-scale effort to beat fishery blues | Aquaculture | South Africa | 2007 | 0 |
| Center to target salmon research; Genetics focus of new facility in Franklin | Aquaculture | United States | 2007 | 0 |
| Feast of prawns worthy of seconds | Aquaculture | Australia | 2007 | 1 |
| Locals protest plans for fish farm | Aquaculture | Nova Scotia | 2007 | -1 |
| (Briefs) | Aquaculture | United States | 2007 | 0 |
| Prawns, prawns, glorious prawns | Aquaculture | Australia | 2007 | 1 |
| Extra long lunch to cater for demand; Prawns net a crowd | Aquaculture | Australia | 2007 | 1 |
| Prawns on the move | Aquaculture | Australia | 2007 | 0 |
| Prawn prices face rise on import blitz | Aquaculture | Australia | 2007 | 0 |
| World Wetlands Day; Guided tour of Okato reserve | Aquaculture | New Zealand | 2007 | 0 |
| Ahern announces EUR 600m fisheries plan | Aquaculture | Ireland | 2007 | 0 |
| Fish farm death dive 'broke all rules' | Aquaculture | United Kingdom | 2007 | -1 |
| Farmed fish swim to the fore; How we handle aquaculture may determine the future of the planet | Aquaculture | United States | 2007 | 1 |
| Far more choice than the three Rs; BACK TO SCHOOL - A Newcastle Herald Advertising Feature | Aquaculture | Australia | 2007 | 0 |
| IN BRIEF... | Aquaculture | Australia | 2007 | 0 |
| Fish farms in the ocean? Group pushes Congress to pass tough rules. | Aquaculture | United States | 2007 | -1 |
| LEGAL NOTICES - 17 OF 17 | Aquaculture | United States | 2006 | 0 |
| St John's Island | Aquaculture | Singapore | 2006 | 0 |
| Guyana to set up fisheries committee | Aquaculture | Guyana | 2006 | 0 |
| Teens stuck at sea as trip goes wrong | Aquaculture | Australia | 2006 | 0 |
| Wildlife learning centre | Aquaculture | United Kingdom | 2006 | 0 |
| 5 things you didn't know about... | Aquaculture | Singapore | 2006 | 0 |
| Maritime opportunity | Aquaculture | Australia | 2006 | 1 |
| Campus' size has doubled | Aquaculture | United Kingdom | 2006 | 0 |
| P5-M fisheries lab to be set up in GenSan | Aquaculture | Philippines | 2006 | 0 |
| Shop wisely or 'go fish' | Aquaculture | Australia | 2006 | 0 |
| College staff and students inspect new building | Aquaculture | United Kingdom | 2006 | 0 |
| Students get hands-on reservoir experience | Aquaculture | United Kingdom | 2006 | 0 |
| College presents its zoological research | Aquaculture | United Kingdom | 2006 | 0 |
| Fish farm educational hook for keen students | Aquaculture | Australia | 2006 | 1 |
| Students are hooked on harvesting fish | Aquaculture | Australia | 2006 | 1 |
| College welcomes new headteacher to campus | Aquaculture | United Kingdom | 2006 | 0 |
| SA 'aims to be one of top 10 destinations' | Aquaculture | South Africa | 2006 | 0 |
| friday fishing | Aquaculture | Australia | 2006 | 0 |
| Pioneering project to help the king of fish | Aquaculture | United Kingdom | 2006 | 1 |
| State losing ground in bioscience jobs | Aquaculture | United States | 2006 | -1 |
| Farm lands prawn solution | Aquaculture | Australia | 2006 | 1 |
| Namibian president urges Swedish businessmen to invest in country | Aquaculture | Namibia | 2006 | 0 |
| in the zone | Aquaculture | South Africa | 2006 | 0 |
| Lean pickings under new marine farm laws | Aquaculture | New Zealand | 2006 | -1 |
| Course recruits sample life beneath the waves | Aquaculture | United Kingdom | 2006 | 0 |
| The fishing's fine at Northam | Aquaculture | Australia | 2006 | 0 |
| Funds for marine science students | Aquaculture | Australia | 2006 | 0 |
| Govt pulls plug on Port fish farm | Aquaculture | Australia | 2006 | -1 |
| Cawthron role `exciting challenge' | Aquaculture | New Zealand | 2006 | 0 |
| Sea food, just; had to eat it | Aquaculture | Australia | 2006 | 0 |
| Malaysian firm helps turn Namibia around | Aquaculture | Malaysia | 2006 | 1 |
| Blueprint to beat skills shortage | Aquaculture | Australia | 2006 | 0 |
| Zoo gains training praise | Aquaculture | United Kingdom | 2006 | 0 |
| Students uncover undersea world of opportunities | Aquaculture | Canada | 2006 | 1 |
| Western Isles salmon firm to trial greener production | Aquaculture | Scotland | 2006 | 1 |
| College bids to expand with new Â£1.9m centre | Aquaculture | United Kingdom | 2006 | 0 |
| Seafood trail map | Aquaculture | New Zealand | 2006 | 0 |
| Plan for organic farm joint venture in Pangkor | Aquaculture | Malaysia | 2006 | 0 |
| LEGAL NOTICES - 3 OF 3 | Aquaculture | United States | 2005 | 0 |
| Scientists win $10,000 grant | Aquaculture | Australia | 2005 | 0 |
| Expert touts plan to boost R&D spending | Aquaculture | United States | 2005 | 0 |
| Claims of benefits to raising fish in sea are full of snags | Aquaculture | United States | 2005 | -1 |
| Company Briefs | Aquaculture | Singapore | 2005 | 0 |
| Corea Heath changes hands in land transfer | Aquaculture | United States | 2005 | 0 |
| Red tape sinks tuna farm plan | Aquaculture | Australia | 2005 | -1 |
| Tuna off the menu in Esperance | Aquaculture | Australia | 2005 | -1 |
| Rising student levels spark expansion plan | Aquaculture | United Kingdom | 2005 | 0 |
| HOLD THAT PROJECT;As fuel and material costs spiral upward, a winter hiatus can yield big savings for builders and clients | Aquaculture | United States | 2005 | -1 |
| Oceanus in research tie-up with NUS body;MOU to further aquaculture research in Singapore | Aquaculture | Singapore | 2005 | 1 |
| Chinese fish-breeder casts IPO net towards Singapore;Oceanus lodges prospectus with the central bank for a mainboard listing | Aquaculture | Singapore | 2005 | 0 |
| Mussel farm at Eastern Bay discussed; Decision on proposal by Tenants Harbor firm delayed so flats can be studied | Aquaculture | United States | 2005 | 0 |
| Oak Hill pines to annex southern parcel | Aquaculture | United States | 2005 | 0 |
| A healthy dose of IT | Aquaculture | Australia | 2005 | 0 |
| GOING FOR LISTING: Borneo Aqua expanding | Aquaculture | Malaysia | 2005 | 0 |
| All Silver and Smiles: Aquaculture brings hope, economic stability to remote Klemtu and its First Nations residents | Aquaculture | Canada | 2005 | 1 |
| Fish farms stay under microscope: Industry has its share of supporters and detractors. In B.C., 4,000 workers earn a living in aquaculture. | Aquaculture | Canada | 2005 | -1 |
| THINK TANK | Aquaculture | Australia | 2005 | 0 |
| Confidence in aquaculture | Aquaculture | Australia | 2005 | 1 |
| Breeder plans 10 fish farms off Ubin;Swee Chioh Aquaculture's move to yield 500 tonnes of fish annually for local market | Aquaculture | Singapore | 2005 | 1 |
| Judging who's the big cheese | Aquaculture | Australia | 2005 | 0 |
| New shellfish land will need additional rules | Aquaculture | Canada | 2005 | -1 |
| Fisheries college jobs to go after backing reduced | Aquaculture | Scotland | 2005 | 0 |
| Sustainable seafood; Marine life ECO | Aquaculture | Australia | 2004 | 0 |
| Research project aims to dispel fears over Scottish farmed salmon | Aquaculture | Scotland | 2004 | 1 |
| Business: Call to tap into lucrative fish-farm industry;Use aquaculture know-how to expand into the region to meet rising global demand for fish, says minister | Aquaculture | Singapore | 2004 | 1 |
| Holy flounder; Tank-raised halibut, ugly yet valuable, could pad Mainer's pocketbooks | Aquaculture | United States | 2004 | 1 |
| $30m fund to take inventions to market;Entrepreneurial arm of NUS aims to help 100 promising start-ups in the next three to five years, including foreign ones hatched here | Aquaculture | Singapore | 2004 | 1 |
| Small fish big bucks;Fish farming goes from sunset industry to knowledge-driven sector | Aquaculture | Singapore | 2004 | 1 |
| Get bitten by research bug | Aquaculture | Malaysia | 2004 | 0 |
| Tech@Work: All the right connections | Aquaculture | Singapore | 2004 | 0 |
| Making its mark at home and abroad | Aquaculture | Malaysia | 2004 | 0 |
| Helping to re-stock the camel | Aquaculture | United Kingdom | 2004 | 0 |
| AVA-bred fish to go on sale in stores here;The first batch of pompano is from deep net-cage farms, which yield more than other coastal fish farms | Aquaculture | Singapore | 2004 | 1 |
| Curtin broadens its appeal | Aquaculture | Australia | 2004 | 0 |
| Oyster paradise | Aquaculture | Australia | 2004 | 1 |
| Singapore global business fair will be annual affair;Spurred by success of inaugural Global Entrepolis last year, EDB hopes to see 25,000 participants at this year's event | Aquaculture | Singapore | 2004 | 0 |
| Scientists show invasion of the sea creatures;A survey reveals 18 non-native species in just three places on Casco Bay. | Aquaculture | United States | 2004 | -1 |
| Camel gets :2m clean-up | Aquaculture | United Kingdom | 2004 | 0 |
| :2m clean-up of river after lobster deaths | Aquaculture | United Kingdom | 2004 | -1 |
| Ocean report has long reach | Aquaculture | United States | 2004 | 0 |
| UMS courses relevant to nation's need | Aquaculture | Malaysia | 2004 | 0 |
| S'pore should be angling for more bucks in fish trade | Aquaculture | Singapore | 2004 | 0 |
| UMS makes waves globally | Aquaculture | Malaysia | 2004 | 0 |
| Overseas investors snap up fish farm after failure to make NSX | Aquaculture | Australia | 2003 | 1 |
| Across The West | Aquaculture | United Kingdom | 2003 | 0 |
| University Campus Opens | Aquaculture | United Kingdom | 2003 | 0 |
| Research plays important role at KUSTEM | Aquaculture | Malaysia | 2003 | 0 |
| UCC appoints new dean of math, tech | Aquaculture | Canada | 2003 | 0 |
| Row Flares Over Fish Unit'S Future | Aquaculture | Scotland | 2003 | -1 |
| Canadian firms hired to analyze aquaculture | Aquaculture | United States | 2003 | 0 |
| Plenty of fish in the sea? Think again...;Fishermen and consumers are feeling the pinch as fish stocks are depleted through over-fishing | Aquaculture | Singapore | 2003 | 0 |
| Only nine but UMS forges ahead | Aquaculture | Malaysia | 2003 | 0 |
| Hopes Rise For Future Of Ardtoe Marine Research Unit | Aquaculture | Scotland | 2003 | 0 |
| Schools' learning curve - Biggest curriculum shake-up for 30 years | Aquaculture | Australia | 2003 | 0 |
| New marine aquaculture goes after bigger fish | Aquaculture | Singapore | 2003 | 1 |
| Equally delicious, steamed or fried | Aquaculture | Singapore | 2003 | 1 |
| Small fry? Think again...it's big plan to feed S'poreans;High-tech breeding facility on St John's Island aims to produce almost half the fish people eat here | Aquaculture | Singapore | 2003 | 1 |
| Healthy oceans Pew report signals need for further remedies | Aquaculture | United States | 2003 | -1 |
| TOPICS TODAY | Aquaculture | Australia | 2003 | 0 |
| Port Ed hatchery closes its doors: q Marine Harvest Canada mothballs state-of-art $8 million facility; blames feds, misinformation | Aquaculture | Canada | 2003 | -1 |
| Government grants | Aquaculture | Australia | 2003 | 0 |
| Blair Is Dragged Into Ardtoe Row | Aquaculture | Scotland | 2003 | 0 |
| Fish farming for Bay sites | Aquaculture | Australia | 2003 | 0 |
| Investors lured by fish farm shares | Aquaculture | Australia | 2003 | 1 |
| Ubin prawns going places;Farm expanding to become fish hatchery, as well as producing new dried prawn snack using small prawns no one wants | Aquaculture | Singapore | 2003 | 1 |
| Better chance for say | Aquaculture | Australia | 2002 | 0 |
| Plan for abalone farm put on show | Aquaculture | Australia | 2002 | 1 |
| UE unit clinches $200m PUB contract | Aquaculture | Singapore | 2002 | 0 |
| Raise aquaculture production, marine scientists urged | Aquaculture | Malaysia | 2002 | 1 |
| Fish farm in $2.5m listing bid | Aquaculture | Australia | 2002 | 0 |
| Breaking away from the herd | Aquaculture | Australia | 2002 | 0 |
| Ofex wants to challenge ASX with trans-Tasman market; Investing | Aquaculture | Australia | 2002 | 0 |
| Regional firms in the limelight; Hunter Region Export Awards 2002 A Newcastle Herald Advertising Feature | Aquaculture | Australia | 2002 | 0 |
| WHAT A WHOPPER!;ANGLESEY NETS POUNDS 19M EURO FISH FARM DEAL | Aquaculture | Wales | 2002 | 1 |
| In step with the community | Aquaculture | Malaysia | 2002 | 0 |
| Transforming the image - CENTRAL COAST: MOVING FORWARD | Aquaculture | Australia | 2002 | 0 |
| Taking aquaculture and marine science to new heights | Aquaculture | Malaysia | 2002 | 1 |
| Primelife chief hits back over `foul play'; MANAGEMENT | Aquaculture | Australia | 2001 | 0 |
| Something's fishy on this farm | Aquaculture | Japan | 2001 | -1 |
| Marine research centre floated | Aquaculture | Canada | 2001 | 0 |
| Farmers hatch a new plan that may well grow on us; FISHING | Aquaculture | Australia | 2001 | 1 |
| Farm-fresh fish on menu;Banker becomes snapper breeder | Aquaculture | Australia | 2001 | 1 |
| Fortune begins to favour the salmon;The reel thing | Aquaculture | Scotland | 2001 | 1 |
| Two schools of thought on fish farm go-ahead | Aquaculture | Australia | 2001 | 0 |
| Plan for deep-sea fish farming hits snag | Aquaculture | Singapore | 2001 | -1 |
| Business People | Aquaculture | United States | 2001 | 0 |
| Fears spark crusade to 'save' Lough Swilly;New group will battle fish farming plans | Aquaculture | United Kingdom | 2001 | -1 |
| Anglers bite at fish park bid | Aquaculture | Australia | 2001 | -1 |
| Agency to tackle fish-farm waste | Aquaculture | Scotland | 2001 | -1 |
| Primed for prawns | Aquaculture | Australia | 2000 | 1 |
| Offshore investment hooks ex-banker | Aquaculture | Australia | 2000 | 1 |
| Seafood body slams marine park proposal; SEAFOOD | Aquaculture | Australia | 2000 | -1 |
| AVA -- that's PPD's name as a stat board | Aquaculture | Singapore | 2000 | 0 |
| Tassie divers' safety splash | Aquaculture | Australia | 2000 | 0 |
| Justice and democracy will disappear | Aquaculture | United States | 2000 | 0 |
| WHAT'S NEW; VICTORIA | Aquaculture | Australia | 1999 | 0 |
| Leisure and tourism will become more important in the economy of the South West | Aquaculture | United Kingdom | 1999 | 0 |
| Move to boost fish, vegetable supply | Aquaculture | Singapore | 1999 | 1 |
| Asia claws back onto the local economic agenda | Aquaculture | Australia | 1999 | 0 |
| A project going swimmingly / THE HUNTER REGION | Aquaculture | Australia | 1999 | 1 |
| Council nets fish farms | Aquaculture | Australia | 1999 | 0 |
| Sorry, tuna is calling the tune | Aquaculture | Australia | 1999 | 0 |
| A city fights back | Aquaculture | Australia | 1999 | -1 |
| Sarawak optimistic about robust economic prospects | Aquaculture | Malaysia | 1999 | 1 |
| Barbed wire, fences go up at St John's | Aquaculture | Singapore | 1999 | 0 |
| Your views | Aquaculture | Canada | 1999 | 0 |
| A snapper of the fingerlings | Aquaculture | Australia | 1999 | 0 |
| St John's to have marine research centre | Aquaculture | Singapore | 1999 | 0 |
| Sea cages yield first commercial harvests; FISHING | Aquaculture | Australia | 1998 | 1 |
| POLITICS: BLAIR POISED TO MEET TRIMBLE FOR POLICY TALKS | Aquaculture | Ireland | 1998 | 0 |
| Blair unveils UK-Irish body in Dublin | Aquaculture | United Kingdom | 1998 | 0 |
| Blair to unveil UK-Irish forum | Aquaculture | United Kingdom | 1998 | 0 |
| Industry, nature can co-exist | Aquaculture | United States | 1998 | 1 |
| Our tainted coast; Report pinpoints pollution and the;solution | Aquaculture | Australia | 1998 | -1 |
| Fortune in flying fish | Aquaculture | Australia | 1998 | 0 |
| Peace Corps Worker Killed in Philippines | Aquaculture | United States | 1998 | 0 |
| NEWS IN BRIEF | Aquaculture | Philippines | 1998 | 0 |
| Mum, can we have fish? | Aquaculture | Singapore | 1998 | 0 |
| Call to release secret report on aquacentreDecision to close college has been hotly disputed | Aquaculture | Scotland | 1998 | 0 |
| NBT continues focus on core areas | Aquaculture | Malaysia | 1998 | 0 |
| Fishing for complements | Aquaculture | Australia | 1997 | 0 |
| Norwegian study of our aquaculture potential | Aquaculture | Malaysia | 1997 | 1 |
| Lucas Heights | Aquaculture | Australia | 1997 | 0 |
| Business groups main goal of growth study | Aquaculture | Australia | 1997 | 0 |
| AS AGE OF AQUACULTURE DAWNS, WE CAN LEARN FROM OTHERS' MISTAKES; VIGILANCE OVER DISEASE AND NUTRIENT POLLUTION ARE ESSENTIAL. | Aquaculture | United States | 1997 | 0 |
| Fiji aims for increase in visitors | Aquaculture | Australia | 1997 | 0 |
| Tuna farms 'threat' to KI penguins | Aquaculture | Australia | 1997 | -1 |
| State inquiry says no to oyster farms | Aquaculture | Australia | 1996 | -1 |
| Opportunities in global halal' market | Aquaculture | Malaysia | 1996 | 1 |
| Minister with a true flair for stating the blindingly obvious | Aquaculture | Ireland | 1996 | 0 |
| A mixture of satisfaction and fear | Aquaculture | Malaysia | 1996 | 0 |
| Agency turns ideas into cash for coast A million clams will make them as happy as . . . | Aquaculture | United States | 1996 | 1 |
| Breeding grouper on the farm | Aquaculture | Singapore | 1996 | 1 |
| Promoting R & D | Aquaculture | Singapore | 1996 | 0 |
| Mystery virus attack | Aquaculture | Singapore | 1996 | -1 |
| BALLAST WATER ORGANISMS AN ENVIRONMENTAL THREAT | Aquaculture | Australia | 1996 | -1 |
| IVF WHITING RESEARCH TO HELP DECLINING NUMBERS | Aquaculture | Australia | 1996 | 0 |
| Oysters hailed as solution to feeding Africa's hungry | Aquaculture | Canada | 1996 | 1 |
| Aquaculture centre officially opened | Aquaculture | Australia | 1996 | 1 |
| Intellectual Property India Publishes Patent Application for 'Offshore Aquaculture System' Filed by Sea Control Holdings | Aquaculture | India | 2015 | 0 |
| Catalina Sea Ranch project moves ahead | Aquaculture | USA | 2014 | 1 |
| Making mussels | Aquaculture | USA | 2014 | 1 |
| Capital gets Maritime Plan 2030 for sustainable development | Aquaculture | United Arab Emirates | 2014 | 1 |
| Who Is Conservation For? | Aquaculture | USA | 2013 | -1 |
| Mussel beds planned near wind farm | Aquaculture | USA | 2013 | 1 |
| Seafood worker Hong Kong-bound | Aquaculture | China | 2013 | 1 |
| Oyster beds off Crowes Pasture hit again | Aquaculture | USA | 2013 | -1 |
| Judge says Marin oyster farm must obey Coastal Commission order | Aquaculture | USA | 2013 | -1 |
| Port's waters no good to explore | Aquaculture | Australia | 2013 | -1 |
| New lawsuit claims state wrongly put sanctions on Drakes Bay oyster farm | Aquaculture | USA | 2013 | 0 |
| Point Reyes oyster farm dealt another setback | Aquaculture | USA | 2013 | -1 |
| MARITIME POLICY : ATLANTIC ARC AT HEART OF NEW MACRO-REGIONAL STRATEGY | Aquaculture | Portugal | 2011 | 0 |
| Ireland 'suffered' over fisheries policy | Aquaculture | Ireland | 2011 | -1 |
| Irish food drive serves up tasty results | Aquaculture | Ireland | 2011 | 1 |
| Bluefin tuna fingerlings sea trial survival rates hailed | Aquaculture | Australia | 2011 | 1 |
| Clean Seas buoyed by tuna trials | Aquaculture | Australia | 2011 | 1 |
| Tuna trial victory | Aquaculture | Australia | 2011 | 1 |
| Raising tuna in a tank closer to reality, researcher says | Aquaculture | USA | 2011 | 1 |
| No fish, no drill marine national park | Aquaculture | Australia | 2011 | -1 |
| Humongous fish farms are industrializing our oceans | Aquaculture | USA | 2011 | -1 |
| Bluefin breeding on track | Aquaculture | Australia | 2011 | 1 |
| Marsh likely to be gone by 2100 | Aquaculture | USA | 2010 | -1 |
| Maine sites considered for testing wave energy; Resolute Marine's CEO envisions its converters using the same areas as floating wind turbines. | Aquaculture | USA | 2010 | 1 |
| Go slow, mussel-breeders urge speeding ferry boats | Aquaculture | Malaysia | 2009 | 0 |
| TIME FOR A GREEN POWER TRIP; Renewable energy technologies offer not only huge commercial opportunities but also some pretty novel science | Aquaculture | United Kingdom | 2009 | 1 |
| CEO plans added value for exports | Aquaculture | Australia | 2009 | 1 |
| Farming the ocean as we farm the land | Aquaculture | USA | 2009 | 1 |
| Fish farming gets OK for fed waters | Aquaculture | USA | 2009 | 1 |
| Reefs get $3.3 million stimulus: Staghorn, elkhorn nurseries to boost reef restoration | Aquaculture | USA | 2009 | 1 |
| Rove pick doesn't negate group's work | Aquaculture | USA | 2009 | 1 |
| Secrets of the deep blue sea | Aquaculture | Ireland | 2008 | 1 |
| FISH FARMS IN GULF TOO RISKY | Aquaculture | USA | 2008 | -1 |
| Exxon spill awards could be held up in court for several weeks | Aquaculture | USA | 2008 | -1 |
| 100,000 sea breams to be released into sea | Aquaculture | Bahrain | 2008 | 1 |
| OUTDOORS NOTES | Aquaculture | USA | 2008 | 0 |
| Clean Seas husbandry a tuna aquaculture breakthrough - South Australia: Special Report | Aquaculture | Australia | 2008 | 1 |
| Salmon Farming May Doom Wild Populations, Study Says | Aquaculture | USA | 2007 | -1 |
| Marine advisory council to discuss oil spill | Aquaculture | USA | 2007 | -1 |
| Maine island fall cleanups deserve praise; Adventure club meets Oct. 30 | Aquaculture | USA | 2007 | 1 |
| Threats of fish farming | Aquaculture | USA | 2007 | -1 |
| Deep-water aquaculture firm attracts vencap investment | Aquaculture | Canada | 2007 | 1 |
| LEGAL NOTICES | Aquaculture | USA | 2007 | 0 |
| U.S. is wary of ocean fish farms despite flood of foreign seafood; But experts say open-ocean aquaculture is an issue of when and how -- not if | Aquaculture | USA | 2007 | -1 |
| IMPORTS FUEL PUSH FOR U.S. OCEAN FISH FARMS | Aquaculture | USA | 2007 | 1 |
| A changing province; By 2050, Scott Simpson and Gordon Hamilton report, projections indicate global warming will make the British Columbia we know a vastly different place. | Aquaculture | Canada | 2007 | -1 |
| Aquaculture advance hailed | Aquaculture | United Kingdom | 2007 | 0 |
| How many little fish does it take to grow a big fish?; Aquaculture's abundant promise must be buttressed by appropriate safeguards. | Aquaculture | USA | 2007 | -1 |
| Feds eye deep-sea fish farms; Similar operations already allowed in U.S. | Aquaculture | Canada | 2007 | 1 |
| Ottawa interested in U.S. plan for deep-sea fish farms | Aquaculture | Canada | 2007 | 1 |
| Dire Forecast for the Oceans | Aquaculture | USA | 2006 | -1 |
| OUTDOORS NOTES | Aquaculture | USA | 2006 | 0 |
| N.C. still opposing offshore oil drilling | Aquaculture | USA | 2006 | -1 |
| DETERMINATION 'Half-illegal immigrant' an SA giant; 'Can do' the only way to succeed | Aquaculture | Australia | 2006 | -1 |
| New leases will add mussel to state's industry | Aquaculture | Australia | 2006 | 1 |
| KINGFISH Hope of expanding European market; Fishing giant targets Russia in export trial | Aquaculture | Australia | 2006 | 1 |
| Green to the Gills | Aquaculture | USA | 2006 | 1 |
| Stevens casts net in offshore farm-fishing permit debate | Aquaculture | USA | 2006 | 0 |
| Massachusetts governor gets veto power in wind farm bill | Aquaculture | USA | 2006 | 0 |
| Delegates set policy direction for soybean group Sioux Falls Ã Directors of the South Dakota Soybean Association (SDSA) joined other producers from around ... | Aquaculture | USA | 2006 | 0 |
| FISH OF THE FUTURE?; Hawaiian fish farm begins harvest of Kona Kampachi | Aquaculture | USA | 2006 | 1 |
| Fish of the future?; HAWAIIAN COMPANY RAISES STANDARDS ON DEEP-SEA FARM | Aquaculture | USA | 2006 | 1 |
| BIG SHOT | Aquaculture | Australia | 2006 | 0 |
| I hear and accept others' viewpoints | Aquaculture | Canada | 2005 | 0 |
| OFF SHORE AND OFF TARGET | Aquaculture | USA | 2005 | -1 |
| White House Seeks to Boost Fish Farms by Expanding Into Open Waters | Aquaculture | USA | 2005 | 1 |
| Alaskans attack fish farms | Aquaculture | Canada | 2005 | -1 |
| Catch a big one in Albany | Aquaculture | Australia | 2005 | 1 |
| Marine Life Complicates Removal of Old Oil Rigs | Aquaculture | USA | 2005 | 0 |
| Hopes raised for deep-sea fish farms; White House proposes removing some 'obstacles' | Aquaculture | Ireland | 2005 | 1 |
| Offshore energy a bridge to future | Aquaculture | Canada | 2005 | 1 |
| Fish cages vital for a marine revolution, says BIM | Aquaculture | Ireland | 2005 | 1 |
| Ideas hauled in out of the blue | Aquaculture | Australia | 2005 | 0 |
| Canadians with that long-distance feeling; Campobello Islanders have long considered themselves more like Americans, but 9/11 and now the rise of Stephen Harper seem to be changing all that, SHAWNA RICHER reports | Aquaculture | Canada | 2004 | 0 |
| Bird lovers count beaks on holiday | Aquaculture | USA | 2004 | 0 |
| Plans for offshore fish farm rejected | Aquaculture | USA | 2004 | -1 |
| Panel recommends denial of gulf aquaculture permit | Aquaculture | USA | 2004 | -1 |
| Casting doubt on fish farming | Aquaculture | USA | 2004 | -1 |
| Public shouldn't have to pay for the power grid | Aquaculture | USA | 2004 | -1 |
| RAISING MORE FISH IN CAGES SEEMS A CERTAINTY | Aquaculture | USA | 2004 | 1 |
| New boat era for fish farms | Aquaculture | Australia | 2004 | 1 |
| Turkish spinoffs possible | Aquaculture | USA | 2004 | 0 |
| Fish farm seen as danger to seals | Aquaculture | Australia | 2004 | -1 |
| Maine Salmon Face Upstream Battle as Species; Returns Dwindle as Aquaculture Booms | Aquaculture | USA | 2004 | -1 |
| Fishing for growth | Aquaculture | Australia | 2004 | 1 |
| Storm Swirls Over Aboriginal Salmon In Maine's Rivers | Aquaculture | USA | 2004 | -1 |
| Scientific dreams of a sustainable future; Nature Report | Aquaculture | Australia | 1994 | 1 |
| Prawns a growing business in Port | Marine Aquaculture | Australia | 2015 | 1 |
| Speech by SMS Desmond Lee at International Aquaculture Singapore Conference | Marine Aquaculture | Singapore | 2015 | 0 |
| List of Fisheries for 2016 | Marine Aquaculture | United States | 2015 | 0 |
| FEDERAL REGISTER: List of Fisheries for 2016 | Marine Aquaculture | United States | 2015 | 0 |
| JCU bid for Ross Creek marine hub | Marine Aquaculture | Australia | 2015 | 0 |
| SL's economy grows 6.7% in 2Q | Marine Aquaculture | Sri Lanka | 2015 | 0 |
| Sri Lanka economy grew 6.7-pct in Q2 2015 | Marine Aquaculture | Sri Lanka | 2015 | 0 |
| Media to descend on Cairns | Marine Aquaculture | Australia | 2015 | 0 |
| N$12 Million for Aquaculture | Marine Aquaculture | Namibia | 2015 | 1 |
| Washington: King, National Park Service Officially Open Schoodic Woods Campground | Marine Aquaculture | United States | 2015 | 0 |
| Fisheries Launches Plan to Bridge Inequality | Marine Aquaculture | Namibia | 2015 | 0 |
| DNV GL Announces Acquisition of Noomas Certification | Marine Aquaculture | India | 2015 | 0 |
| Project could give boost to marine aquaculture | Marine Aquaculture | United States | 2015 | 1 |
| Aqua Culture Master Plan Unveiled | Marine Aquaculture | Namibia | 2015 | 0 |
| FEDERAL REGISTER: Endangered and Threatened Species: Final Rulemaking To Revise Critical Habitat for Hawaiian Monk Seals | Marine Aquaculture | United States | 2015 | 0 |
| Whole fish approach \| Plant would process the entire fish to produce fish chow, fish oil - maybe even fertilizer. | Marine Aquaculture | United States | 2015 | 0 |
| Spain challenges subsidy on Turkish sea products, exporters upset | Marine Aquaculture | Turkey | 2015 | -1 |
| Coastal resources face threat | Marine Aquaculture | Australia | 2015 | -1 |
| Something fishy is netting a profit in schoolgirl's life Youngster's idea turns into a thriving | Marine Aquaculture | South Africa | 2015 | 1 |
| Small fry at first maybe, but teen's fish farm's growing; Tilapia waste feeds the hydroponic vegetables that float her business in an innovative recycling system | Marine Aquaculture | South Africa | 2015 | 1 |
| Shrimp Export up by 56 Per Cent in State | Marine Aquaculture | India | 2015 | 1 |
| Environmental Impact Statements; Availability, etc.; Weekly Receipts | Marine Aquaculture | United States | 2015 | 0 |
| Don't come raw prawn, fraud told over $19m tax bill | Marine Aquaculture | Australia | 2015 | -1 |
| Hayleys Agriculture wins AREA Award in Social Empowerment | Marine Aquaculture | Sri Lanka | 2015 | 0 |
| Washington: S. Rept. 114-66 - DEPARTMENTS OF COMMERCE AND JUSTICE, AND SCIENCE, AND RELATED AGENCIES APPROPRIATIONS BILL, 2016 | Marine Aquaculture | United States | 2015 | 0 |
| FEDERAL REGISTER: Spring 2015 Semiannual Agenda of Regulations | Marine Aquaculture | United States | 2015 | 0 |
| More aquaculture challenges | Marine Aquaculture | New Zealand | 2015 | -1 |
| Coastal areas ecosystem must for blue economy | Marine Aquaculture | Bangladesh | 2015 | 0 |
| B.C. salmon farm appeals Federal Court order | Marine Aquaculture | Canada | 2015 | -1 |
| Aquaculture firm appealing protection order | Marine Aquaculture | Canada | 2015 | -1 |
| Salmon farm appeals Federal Court ruling | Marine Aquaculture | Canada | 2015 | -1 |
| New twist in controversial fish farm plan | Marine Aquaculture | South Africa | 2015 | -1 |
| SA is open for business as a smart investment | Marine Aquaculture | South Africa | 2015 | 1 |
| US Contract Notice: Department of Agriculture Issues Solicitation for Astaxathin & Canthazanthin Analysis"" | Marine Aquaculture | United States | 2015 | 0 |
| Marine teachers go back to fishy school | Marine Aquaculture | Australia | 2015 | 0 |
| Alaska: Introduction to Shellfish Aquaculture - Module 2 | Marine Aquaculture | United States | 2015 | 0 |
| Centre not issuing funds for Sindh marine products export | Marine Aquaculture | Pakistan | 2015 | 0 |
| EU move to boost PH fishing targets | Marine Aquaculture | Philippines | 2015 | 0 |
| Lifting of 'yellow card' to generate $300-M fishery exports to EU | Marine Aquaculture | Philippines | 2015 | 1 |
| Rep. Huffman Introduces Fishing Economy Improvement Act | Marine Aquaculture | United States | 2015 | 1 |
| Costa-Pierce Talks on Ocean Food Systems at Harvard | Marine Aquaculture | United States | 2015 | 0 |
| Rs 107-cr seafood park in Deras | Marine Aquaculture | India | 2015 | 0 |
| Super diet promises a prawn bounty | Marine Aquaculture | Australia | 2015 | 1 |
| Luncheon lives up to the hype | Marine Aquaculture | Australia | 2015 | 0 |
| Real cost of injury in the farm sector | Marine Aquaculture | Australia | 2015 | -1 |
| Colleges asked to push practical skills | Marine Aquaculture | China | 2015 | 0 |
| Fish farmers restock as water quality appears improved; Some move their stocks back into open waters after recent fish deaths | Marine Aquaculture | Singapore | 2015 | -1 |
| injury on farms massive; Time lost | Marine Aquaculture | Canada | 2015 | -1 |
| Asian touch to salmon; Easy to cook and packed with nutrients, roasted salmon fillet is a quick favourite | Marine Aquaculture | Singapore | 2015 | 1 |
| Prahok put to the quality test | Marine Aquaculture | Cambodia | 2015 | 0 |
| New Facilities to Boost Aquaculture Production | Marine Aquaculture | Namibia | 2015 | 1 |
| Mass fish deaths hit Changi farmers hard | Marine Aquaculture | Thailand | 2015 | -1 |
| Mass fish deaths overnight hit Changi farmers hard | Marine Aquaculture | Singapore | 2015 | -1 |
| Washington: Endangered and Threatened Species; Critical Habitat for Endangered North Atlantic Right Whale | Marine Aquaculture | United States | 2015 | 0 |
| Arizona: Endangered and Threatened Species: Critical Habitat for Endangered North Atlantic Right Whale | Marine Aquaculture | United States | 2015 | 0 |
| Following fish | Marine Aquaculture | Pakistan | 2015 | 0 |
| NAQUA presented with 'Best Aquaculture Practices' certificate | Marine Aquaculture | United States | 2015 | 1 |
| Corporate: Barramundi Asia plans global expansion, backed by A*STAR and local entrepreneur | Marine Aquaculture | Singapore | 2015 | 1 |
| Pakistan: Benefits of tilapia farming highlighted | Marine Aquaculture | Pakistan | 2015 | 1 |
| Benefits of tilapia farming highlighted | Marine Aquaculture | Pakistan | 2015 | 1 |
| Johor eyes position as hub for marine studies | Marine Aquaculture | Malaysia | 2015 | 0 |
| 'Marine Pollution Posing Challenge To Fish Trade, Human Health' | Marine Aquaculture | India | 2015 | -1 |
| New Hampshire: N.H. Marine Rule Changes for 2015 | Marine Aquaculture | United States | 2015 | 0 |
| Marine pollution posing challenge to fish trade, human health: Experts | Marine Aquaculture | India | 2015 | -1 |
| List of Fisheries for 2015 | Marine Aquaculture | United States | 2014 | 0 |
| Washington: List of Fisheries for 2015 | Marine Aquaculture | United States | 2014 | 0 |
| Washington: Fall 2014 Semiannual Agenda of Regulations | Marine Aquaculture | United States | 2014 | 0 |
| Washington: Introduction to the Unified Agenda of Federal Regulatory and Deregulatory Actions | Marine Aquaculture | United States | 2014 | 0 |
| China invites PHL in world's first fish 'spot market' | Marine Aquaculture | Philippines | 2014 | 0 |
| Marine Aquaculture Development in the Sultanate of Oman/ Symposium | Marine Aquaculture | Oman | 2014 | 0 |
| Lifeline for offshore marine research unit; St John's Island lab, which was beset by rising operating costs, finds a way to stay open | Marine Aquaculture | Singapore | 2014 | 0 |
| Fuzhou Special: Fuzhou set to build a 21st Century Maritime Silk Road hub | Marine Aquaculture | China | 2014 | 0 |
| 'Blue Revolution' to Revitalise India's Fisheries Sector | Marine Aquaculture | India | 2014 | 0 |
| Fisheries of the Caribbean, Gulf, and South Atlantic; Aquaculture | Marine Aquaculture | United States | 2014 | 0 |
| Washington: Assistant Professor of Risk Communication | Marine Aquaculture | United States | 2014 | 0 |
| You may have heard of a group of fast food restaurants | Marine Aquaculture | Australia | 2014 | 0 |
| Halloween gives me the creeps | Marine Aquaculture | Australia | 2014 | 0 |
| Fish farmers not keen on waste collection services | Marine Aquaculture | Singapore | 2014 | -1 |
| Rising tide | Marine Aquaculture | Thailand | 2014 | -1 |
| Rising tide of litter on S'pore's shorelines; One group nets 14,400kg worth so far this year | Marine Aquaculture | Singapore | 2014 | -1 |
| Hot spell takes a toll on coastal fish farms | Marine Aquaculture | Singapore | 2014 | -1 |
| Appointments to the Climate and Aquaculture Task Forces by the Marine Fisheries Advisory Committee | Marine Aquaculture | United States | 2014 | 0 |
| Queensland to reap benefits of aquaculture boom | Marine Aquaculture | Canada | 2014 | 1 |
| Wainui: A marvellous spot for spat | Marine Aquaculture | New Zealand | 2014 | 1 |
| Students ride wave of support | Marine Aquaculture | Australia | 2014 | 1 |
| SA's past prudence will drive economic renewal | Marine Aquaculture | South Africa | 2014 | 1 |
| Beverage, food firms boost sales, partnership | Marine Aquaculture | Philippines | 2014 | 1 |
| Fisheries of the Caribbean, Gulf, and South Atlantic; Aquaculture | Marine Aquaculture | United States | 2014 | 0 |
| List of Fisheries for 2015 | Marine Aquaculture | United States | 2014 | 0 |
| EC looks to lead 'blue economy' | Marine Aquaculture | South Africa | 2014 | 1 |
| Coega plan for aquafarming | Marine Aquaculture | South Africa | 2014 | 1 |
| Pimp My Cause matches marketing pros with causes that need their help | Marine Aquaculture | United States | 2014 | 0 |
| CBD on the world stage | Marine Aquaculture | Australia | 2014 | 0 |
| MONTARA'S FORGOTTEN VICTIMS"" | Marine Aquaculture | Australia | 2014 | -1 |
| Croc, beer, hat and prawn Queen Mary | Marine Aquaculture | Australia | 2014 | 0 |
| State schedules second meeting about controversial Bagaduce aquaculture | Marine Aquaculture | United States | 2014 | -1 |
| A really \|good oil \|and gas find means jobs | Marine Aquaculture | South Africa | 2014 | 1 |
| Confident that SA can beat global downturn | Marine Aquaculture | South Africa | 2014 | 1 |
| Focusing on marine economy | Marine Aquaculture | South Africa | 2014 | 1 |
| Mississippi: Presley: Agricultural Utility Sales Tax Exemption | Marine Aquaculture | United States | 2014 | 0 |
| Plastic Waste Can Cause N2 Trillion Financial Damage Annually - Unep | Marine Aquaculture | Kenya | 2014 | -1 |
| Water babies; S.E.A. Aquarium at Resorts World Sentosa has bred more than 2,000 marine creatures, including sharks | Marine Aquaculture | Singapore | 2014 | 0 |
| Finding fun in northern delights | Marine Aquaculture | Australia | 2014 | 1 |
| OAM awarded for service to prawn industry | Marine Aquaculture | Australia | 2014 | 1 |
| Turning the tide of our ocean economy could net us (EURO)6.5bn; In a time of world recession, few products can boast the market growth that farmed salmon is experiencing, writes expert Jan Feenstra | Marine Aquaculture | Ireland | 2014 | 1 |
| China sets up 10 bln yuan fund to support development of maritime Silk Road | Marine Aquaculture | China | 2014 | 1 |
| Farmers still reeling from losses after fish deaths | Marine Aquaculture | Singapore | 2014 | -1 |
| Fund to boost China's maritime Silk Road development | Marine Aquaculture | China | 2014 | 1 |
| LIFE-AQUASEF - ECO-EFFICIENT TECHNOLOGIES DEVELOPMENT FOR ENVIRONMENTAL IMPROVEMENT OF AQUACULTURE, AQUASEF | Marine Aquaculture | Spain | 2014 | 1 |
| Poor Sanitation Causes Animal Infertility [column] | Marine Aquaculture | Namibia | 2014 | -1 |
| Seafood canner takes in strategic investor | Marine Aquaculture | South America | 2014 | 0 |
| No 'shellshock' as prawns weather Ita | Marine Aquaculture | Australia | 2014 | 0 |
| Welcome to the world's largest garbage dump | Marine Aquaculture | China | 2014 | -1 |
| Eyes wide open tour | Marine Aquaculture | Australia | 2014 | 0 |
| Improving feed sustainability for marine aquaculture in Vietnam and Australia | Marine Aquaculture | Australia | 2014 | 1 |
| Decline in seafood export | Marine Aquaculture | United Arab Emirates | 2014 | -1 |
| Marine Harvest sells Scottish assets to Cook Acquaculture | Marine Aquaculture | Scotland | 2014 | 0 |
| Prawn star hits gold | Marine Aquaculture | Australia | 2014 | 1 |
| Burtynsky's big ideas translate into big pictures; A Terrible Beauty, on display at VAG, captures humanity's impact on the Earth through the lens of aerial photographs | Marine Aquaculture | Canada | 2014 | -1 |
| Research and Markets: India Commercial Aquafeed Market Outlook 2018 Provides Market Forecast of 2014 & 2018 for Aquaculture Production by the Type of Finfish | Marine Aquaculture | India | 2014 | 0 |
| Best Free Reference | Marine Aquaculture | United States | 2014 | 0 |
| Acquaculture Fish Harvest Low | Marine Aquaculture | Namibia | 2014 | -1 |
| Cheaper sensor to check water quality; Device being developed in S'pore can help local fish farmers | Marine Aquaculture | Singapore | 2014 | 0 |
| Fish deaths a double whammy; Losses add to woes for farmer facing competition from Malaysia | Marine Aquaculture | Singapore | 2014 | -1 |
| 160 tonnes of dead fish found in farms along Johor Straits | Marine Aquaculture | Singapore | 2014 | -1 |
| Fish producer waits for health certificate to export | Marine Aquaculture | South Africa | 2014 | 0 |
| Many things unchanged on quiet St John's; Allure of hilly island continues to reel in a steady stream of nature lovers | Marine Aquaculture | Singapore | 2014 | 0 |
| Shots in the dark; prawns | Marine Aquaculture | Australia | 2014 | 0 |
| GAFRD: Egypt does not produce healthy fish | Marine Aquaculture | Egypt | 2014 | -1 |
| Traders: Better prospects in second half of year | Marine Aquaculture | Philippines | 2014 | 1 |
| Marine insurance firms announce merger plans | Marine Aquaculture | United Kingdom | 2013 | 0 |
| ICMIF: Member News: Proposed Merger for Sunderland Marine | Marine Aquaculture | India | 2013 | 0 |
| Merger deal would create one of world's largest marine insurance operations | Marine Aquaculture | Canada | 2013 | 0 |
| List of Fisheries for 2014 | Marine Aquaculture | United States | 2013 | 0 |
| A journey of history and treats | Marine Aquaculture | Canada | 2013 | 0 |
| EU and Ministry of International Cooperation sign agreement | Marine Aquaculture | Egypt | 2013 | 0 |
| Festive celebrations mean the end of the year looms | Marine Aquaculture | Australia | 2013 | 0 |
| Tull star says: I'm not too old to rock'n'roll | Marine Aquaculture | United Kingdom | 2013 | 0 |
| Guests spin good yarns | Marine Aquaculture | Canada | 2013 | 0 |
| Next trip, try the prawns | Marine Aquaculture | Australia | 2013 | 1 |
| Namibians Must Build Partnerships With Chinese | Marine Aquaculture | Namibia | 2013 | 0 |
| Look back in time to wrap up busy year | Marine Aquaculture | Australia | 2013 | 0 |
| THANKS TO TANKS | Marine Aquaculture | South Africa | 2013 | 1 |
| People's Government of Jilin Province: Sino Russian Economic and Trade Cooperation Project Matchmaking Was Held | Marine Aquaculture | China | 2013 | 0 |
| Europe, Asia recovery to boost export growth | Marine Aquaculture | Philippines | 2013 | 1 |
| Researchers mastering the challenge of our warming oceans | Marine Aquaculture | Australia | 2013 | 1 |
| Dalian Municipal Government: First Wind Index Marine Aquaculture Insurance Initiated | Marine Aquaculture | China | 2013 | 1 |
| US Patent granted to Bradley Innovation Group, LLC (Indiana) on August 13 titled as Process and system for growing crustaceans and other fish"" | Marine Aquaculture | United States | 2013 | 1 |
| Fish studies expand; Research boost a win for marine life | Marine Aquaculture | Australia | 2013 | 1 |
| Dive and fishing business will have you hooked | Marine Aquaculture | New Zealand | 2013 | 1 |
| Seals of disapproval | Marine Aquaculture | Australia | 2013 | -1 |
| Oceans of praise for excellence | Marine Aquaculture | Australia | 2013 | 1 |
| MTI awards $1.6 million to 27 innovative Maine companies | Marine Aquaculture | United States | 2013 | 1 |
| Winemaker banks on a new vintage; DRINKS | Marine Aquaculture | Australia | 2013 | 0 |
| Top prawn shows its breeding | Marine Aquaculture | Australia | 2013 | 1 |
| Oceanpick to donate safety gear to Trinco fishermen | Marine Aquaculture | Sri Lanka | 2013 | 0 |
| The secret power of seaweed; How award-winning research into an old wives' tale is helping psoriasis and eczema sufferers. By Stephanie Bell | Marine Aquaculture | Ireland | 2013 | 1 |
| Trio of awards for MHS | Marine Aquaculture | Scotland | 2013 | 1 |
| Shellfish chief recognised | Marine Aquaculture | Scotland | 2013 | 1 |
| College of Fisheries project team develops solar-biomass hybrid dryer | Marine Aquaculture | India | 2013 | 0 |
| Bid to breed 'iconic' NZ seahorses | Marine Aquaculture | New Zealand | 2013 | 0 |
| DEAKIN University's new Professor of Marine Science | Marine Aquaculture | Australia | 2013 | 0 |
| Finalists vie for awards after record entries received | Marine Aquaculture | Scotland | 2013 | 0 |
| Cooke Aquaculture to pay $490,000 after illegal pesticides kill lobsters in Canada | Marine Aquaculture | United States | 2013 | -1 |
| RAJIV GANDHI CENTRE FOR AQUACULTURE PRODUCES SPECIFIC PATHOGEN FREE L. VANNAMEI BROODSTOCK FOR 1ST TIME IN INDIA | Marine Aquaculture | India | 2013 | 1 |
| NARA to develop aquaculture | Marine Aquaculture | Sri Lanka | 2013 | 1 |
| DEAKIN University is hoping to develop stronger relationships | Marine Aquaculture | Australia | 2013 | 0 |
| SA moves to nurture aquaculture sector | Marine Aquaculture | South Africa | 2013 | 1 |
| We do like to adapt beside the seaside; COMMERCIAL REPORT: MORISONS LLP City legal team goes rural with success, finds Rick Wilson | Marine Aquaculture | United Kingdom | 2013 | 0 |
| Prawn lunch draws big crowd | Marine Aquaculture | Australia | 2013 | 1 |
| Haulier clinches fish feed contract | Marine Aquaculture | Scotland | 2013 | 0 |
| Deadline for Scottish awards | Marine Aquaculture | Scotland | 2013 | 0 |
| City 'summit' eyes waterfront growth | Marine Aquaculture | United States | 2013 | 0 |
| Seal scarer device' backed by MSPs | Marine Aquaculture | Scotland | 2013 | 1 |
| Pakistan: Banned nets cause severe harm to marine fisheries | Marine Aquaculture | Pakistan | 2013 | -1 |
| Anti-aquaculture body 'aiding fish farm growth' | Marine Aquaculture | United Kingdom | 2013 | 0 |
| Shellfishermen trying to cope with sea ducks | Marine Aquaculture | United States | 2012 | 0 |
| Concerns over size of proposed new salmon farm in Galway Bay | Marine Aquaculture | Ireland | 2012 | -1 |
| This Atlantic salt has a sweet future | Marine Aquaculture | Ireland | 2012 | 1 |
| Sustainability at the top level | Marine Aquaculture | Australia | 2012 | 1 |
| Marine worker to set sail for US study | Marine Aquaculture | United Kingdom | 2012 | 0 |
| Salmon report shows the effect of politics dictating policy | Marine Aquaculture | Canada | 2012 | 0 |
| A vision takes root at sculpture garden | Marine Aquaculture | United States | 2012 | 0 |
| FORMER Governor-General Michael Jeffery (pictured) has be | Marine Aquaculture | Australia | 2012 | 0 |
| Herbst congratulated | Marine Aquaculture | Australia | 2012 | 0 |
| Fish waste study on chemicals | Marine Aquaculture | United Kingdom | 2012 | -1 |
| Sea bird guides research vessel | Marine Aquaculture | Australia | 2012 | 0 |
| Catch 22 | Marine Aquaculture | Australia | 2012 | 0 |
| UniÃƒ+s growth spurt | Marine Aquaculture | Australia | 2012 | 0 |
| New Mexico, agricultural history to be celebrated at NMSU's Leyendecker science center | Marine Aquaculture | United States | 2012 | 0 |
| Seatrout Population Enhancement Cooperative releasing thousands of seatrout fingerlings in St. Louis Bay | Marine Aquaculture | United States | 2012 | 1 |
| The Central People's Government of the People's Republic of China: More Relief Funding as Tropical Storm Nears | Marine Aquaculture | China | 2012 | 0 |
| Marine course popular | Marine Aquaculture | Australia | 2012 | 0 |
| Selling the prawn farm | Marine Aquaculture | Australia | 2012 | 0 |
| Indonesia - Namibia - Growing Together [opinion] | Marine Aquaculture | Australia | 2012 | 0 |
| Marine park gets ready for opening; Oceanarium's construction complete; animals arriving here daily from overseas | Marine Aquaculture | Singapore | 2012 | 0 |
| Butterworth's ANC stalwart fought for the poor | Marine Aquaculture | South Africa | 2012 | 0 |
| Students get their hands wet | Marine Aquaculture | Australia | 2012 | 0 |
| NARA sets action plan to develop fish industry | Marine Aquaculture | Sri Lanka | 2012 | 1 |
| Fisheries Could Stimulate Economy | Marine Aquaculture | Namibia | 2012 | 1 |
| Land distribution target bared for E. Visayas | Marine Aquaculture | Philippines | 2012 | 0 |
| Fish farming claims are disputed by IFA | Marine Aquaculture | Ireland | 2012 | -1 |
| Kalimbeza Fish Farm Thriving | Marine Aquaculture | Namibia | 2012 | 1 |
| Years of volunteering not unnoticed | Marine Aquaculture | United States | 2012 | 0 |
| Alternative energy project under way | Marine Aquaculture | United States | 2012 | 0 |
| Mariculture is not easy | Marine Aquaculture | Australia | 2012 | -1 |
| Factory and land | Marine Aquaculture | Australia | 2012 | 0 |
| Prawn farmer at last prepared to enjoy a very happy Christmas | Marine Aquaculture | Australia | 2012 | 1 |
| Salmon farm considered at former Navy site in Corea | Marine Aquaculture | United States | 2012 | 0 |
| New report on mariculture urges cautious approach | Marine Aquaculture | Australia | 2012 | -1 |
| NETWORKING PRAWN LUNCH | Marine Aquaculture | Australia | 2012 | 0 |
| Bill could add Â£20m to costs | Marine Aquaculture | Scotland | 2012 | 0 |
| Local lobbyists swimming against political tide | Marine Aquaculture | Ireland | 2012 | -1 |
| Fusion Marine Awarded Â£500,000 for Tilapia Farming | Marine Aquaculture | Zambia | 2012 | 1 |
| Prawns hook big catch | Marine Aquaculture | Australia | 2012 | 1 |
| Researchers learn from our local industries | Marine Aquaculture | Australia | 2012 | 0 |
| China : Demonstration Project on Promotion of the Ecological Aquaculture Mode to Reduce Land-based Pollution (CPR/SGP/OP5/CORE/IW/11/01) Project | Marine Aquaculture | Taiwan | 2012 | 1 |
| COMMON FISHERIES POLICY : COMMISSION SPELLS OUT SOCIAL DIMENSION OF CFP REFORM | Marine Aquaculture | United Kingdom | 2012 | 0 |
| Walvis Bay Is Mariculture Oyster | Marine Aquaculture | Namibia | 2012 | 0 |
| Scientists to showcase new subsea sensor system | Marine Aquaculture | Scotland | 2012 | 0 |
| Tilapia King of Hardap Eco Fishing Farm | Marine Aquaculture | Namibia | 2012 | 1 |
| Quick bites | Marine Aquaculture | Australia | 2012 | 0 |
| people true north North Queensland characters: Nell Braley Spicing up island markets | Marine Aquaculture | Australia | 2012 | 0 |
| Denmark: Innovative Multi-purpose off-shore platforms: planning, Design and operation (MERMAID) | Marine Aquaculture | Denmark | 2012 | 1 |
| Rising development increases environmental woes | Marine Aquaculture | Middle East | 2012 | -1 |
| Prawns galore, with one catch | Marine Aquaculture | Australia | 2011 | 0 |
| 'Green tape' delays prawn industry growth | Marine Aquaculture | Australia | 2011 | -1 |
| Norway: Innovative Rearing and Stunning of Farmed Turbot and Sole to Meet Future Challenges Regarding Quality of Production and Animal Welfare (MAXIMUS) | Marine Aquaculture | United Kingdom | 2011 | 0 |
| POUNDS 10K FINE TO PROTECT FISH | Marine Aquaculture | United Kingdom | 2011 | -1 |
| Fine for flouting fish rules may rise | Marine Aquaculture | Scotland | 2011 | -1 |
| Anglers and fish farmers may face Â£10,000 fines under new legislation | Marine Aquaculture | United Kingdom | 2011 | -1 |
| Fisheries, a dynamic export | Marine Aquaculture | Sri Lanka | 2011 | 0 |
| SEAFOOD farmers and scientists are battling | Marine Aquaculture | Australia | 2011 | -1 |
| SA's bold energy future | Marine Aquaculture | South Africa | 2011 | 0 |
| Coral farmer to teach Unity College students how to grow delicate saltwater organism | Marine Aquaculture | United States | 2011 | 1 |
| Plan hatched, but fish and shrimps didn't | Marine Aquaculture | Pakistan | 2011 | -1 |
| Fish farmers unite to be heard; Group of 38 banking on numbers to bring down costs, raise industry issues to govt | Marine Aquaculture | Singapore | 2011 | 1 |
| Fish farm co-op under probe; Registry of Cooperative Societies acting on feedback about the way co-op operates | Marine Aquaculture | Singapore | 2011 | -1 |
| A pearler of a plan released | Marine Aquaculture | Australia | 2011 | 1 |
| Award-winning fish farm in pollution row | Marine Aquaculture | Scotland | 2011 | -1 |
| Malaysia has potential to be global leader in arowana production | Marine Aquaculture | Malaysia | 2011 | 1 |
| Fish farm co-op fails to reel in big numbers; Original target did not factor in infrastructure and manpower issues | Marine Aquaculture | Singapore | 2011 | -1 |
| Brazil: Southern oceans education and development | Marine Aquaculture | Mozambique | 2011 | 0 |
| Deakin's $5m boost | Marine Aquaculture | Australia | 2011 | 1 |
| Vision for 2000 full-time uni students on campus | Marine Aquaculture | Australia | 2011 | 0 |
| Deakin University will invest $5 million | Marine Aquaculture | Australia | 2011 | 1 |
| Week Ahead | Marine Aquaculture | United States | 2011 | 0 |
| Government Promotes Aquaculture | Marine Aquaculture | Mozambique | 2011 | 1 |
| Thank your public-sector unions | Marine Aquaculture | Canada | 2011 | 0 |
| Industry worth $300m | Marine Aquaculture | Australia | 2011 | 0 |
| Biofouling reduction in marine aquaculture | Marine Aquaculture | United Kingdom | 2011 | 1 |
| The Blue Revolution - a two part popular science tv-documentary on marine aquaculture | Marine Aquaculture | Norway | 2011 | 1 |
| Due South; When you are short of time but want a beach vacation, look no farther than the Southern Islands of Singapore, a quick boat ride away | Marine Aquaculture | Singapore | 2011 | 0 |
| Funding boost for fish fry, rice research; Projects to develop bigger fish, superior rice are among three to get total of up to $30m | Marine Aquaculture | Singapore | 2011 | 1 |
| New England in Brief | Marine Aquaculture | United States | 2011 | 0 |
| Network for the development of phage therapy in aquaculture | Marine Aquaculture | Greece | 2011 | 1 |
| Virus that struck Chilean fish farming tied to salmon eggs from Norway; More than $2 billion lost and 26,000 workers laid off after 2007 outbreak | Marine Aquaculture | Chile | 2011 | -1 |
| Thai traders, SMEDA discuss joint ventures in SMEs | Marine Aquaculture | Pakistan | 2011 | 0 |
| Pakistan: Thai traders, SMEDA discuss joint ventures in SMEs | Marine Aquaculture | Pakistan | 2011 | 0 |
| A 'Blue Revolution' to fight hunger in Haiti and world; Amid cropland and freshwater shortages, deep-water 'free-range' fish farming gives people protein - and jobs. Modern marine aquaculture could put Haiti on the cutting edge of the fastest-growing global food industry. | Marine Aquaculture | Haiti | 2011 | 1 |
| Firms fined after catalogue of errors led to deaths | Marine Aquaculture | Scotland | 2011 | -1 |
| Anglers reel in free bumper catch; Sea bass that escaped from govt fish farm likely ended up on dinner tables | Marine Aquaculture | Singapore | 2011 | 1 |
| Indonesian students' aquatic adventure | Marine Aquaculture | Australia | 2011 | 0 |
| Indonesian students pay a visit | Marine Aquaculture | Australia | 2011 | 0 |
| Commerce eyes push to beef up aquaculture | Marine Aquaculture | United States | 2011 | 1 |
| Leaders in fish farming crowned | Marine Aquaculture | Scotland | 2011 | 1 |
| Brazil: Southern oceans education and development Project | Marine Aquaculture | Brazil | 2011 | 0 |
| Coega earmarked for finfish venture | Marine Aquaculture | South Africa | 2011 | 1 |
| Brazil: Southern oceans education and development | Marine Aquaculture | Mozambique | 2011 | 0 |
| 'Marine aquaculture vital for Oman' | Marine Aquaculture | Oman | 2011 | 1 |
| Finalists for Crown honours revealed | Marine Aquaculture | Scotland | 2011 | 0 |
| Aquarium fish becoming a problem in Atlantic waters | Marine Aquaculture | United States | 2011 | -1 |
| Prawns glorious prawns | Marine Aquaculture | Australia | 2011 | 1 |
| TUCKING INTO PRAWN FEST | Marine Aquaculture | Australia | 2011 | 0 |
| Breakthrough in tuna farming | Marine Aquaculture | Australia | 2011 | 1 |
| First fish in sea for tuna breeder | Marine Aquaculture | Australia | 2011 | 1 |
| U.S. Proposes Aquaculture Guidelines | Marine Aquaculture | United States | 2011 | 1 |
| Finding the pot of gold in a fish pond | Marine Aquaculture | South Africa | 2011 | 1 |
| Book early for annual prawn lunch | Marine Aquaculture | Australia | 2011 | 0 |
| Fish farms aim to net huge harvest; Co-op will help them get consistent supplies of fish fry and feed | Marine Aquaculture | Singapore | 2011 | 1 |
| A promising partnership of plants and pompano | Marine Aquaculture | United States | 2011 | 0 |
| Emergency watch system - now at sea; Initiative launched by 18 fish farmers aims to provide quick, efficient help | Marine Aquaculture | Singapore | 2011 | 1 |
| Africa fish-farm success | Marine Aquaculture | Africa | 2011 | 1 |
| Business | Marine Aquaculture | Australia | 2010 | 0 |
| Â£15m Aberdeen marine research centre hailed as world class | Marine Aquaculture | Scotland | 2010 | 1 |
| Seize the day, harness the sea | Marine Aquaculture | Norway | 2010 | 1 |
| Confusion on national body's role | Marine Aquaculture | New Zealand | 2010 | 0 |
| All food production affects the environment; Closed-containment salmon farms not the panacea they are said to be | Marine Aquaculture | Canada | 2010 | -1 |
| AIMS tour guides share reef knowledge | Marine Aquaculture | Australia | 2010 | 0 |
| Fish farmers reeling in researchers; High-tech methods sought to protect the environment and boost harvests | Marine Aquaculture | Australia | 2010 | 1 |
| High hopes of fish farmers risk being dead in the water | Marine Aquaculture | Ireland | 2010 | -1 |
| Sassi aquaculture guide a big mistake | Marine Aquaculture | South Africa | 2010 | -1 |
| Worthy Sassi list leads consumers into sea of confusion | Marine Aquaculture | South Africa | 2010 | -1 |
| Master prawn on menu | Marine Aquaculture | Australia | 2010 | 0 |
| Fisheries Must Deal With Threats, Opportunities | Marine Aquaculture | Africa | 2010 | -1 |
| Man proposes 50-acre Trenton oyster farm | Marine Aquaculture | United States | 2010 | 0 |
| Thai traders, SMEDA discuss joint ventures in SMEs | Marine Aquaculture | Pakistan | 2010 | 0 |
| 50,000 fingerlings released into Qatari waters | Marine Aquaculture | Qatar | 2010 | 0 |
| Australian scientists develop the perfect prawn | Marine Aquaculture | Australia | 2010 | 1 |
| Small-scale fisherman seeking bigger benefits from minister | Marine Aquaculture | South Africa | 2010 | 0 |
| Could this be the ultimate prawn star? | Marine Aquaculture | Australia | 2010 | 1 |
| Alliance Tuna to start due diligence on Hiep Thanh | Marine Aquaculture | Viet Nam | 2010 | 0 |
| Australian super prawns are born | Marine Aquaculture | Australia | 2010 | 1 |
| Prawn breeding breakthrough | Marine Aquaculture | Australia | 2010 | 1 |
| A cool time on sweltering St John's; Delegates from around the world are here for the four-day World Cities Summit, which began yesterday. Guests were taken on 'learning journeys' to see how Singapore tackles urban development issues. Rachel Au-Yong and Bryan Toh discover their reactions. | Marine Aquaculture | Singapore | 2010 | 0 |
| AQUACULTURE Success for CSIRO breeding program The perfect prawn | Marine Aquaculture | Australia | 2010 | 1 |
| Net Value: The state of Malaysian biotechnology | Marine Aquaculture | Malaysia | 2010 | 0 |
| College open | Marine Aquaculture | United Kingdom | 2010 | 0 |
| Youth in Print School catches six new fish | Marine Aquaculture | Australia | 2010 | 0 |
| Among nation's best | Marine Aquaculture | Australia | 2010 | 0 |
| Cambodian DPM calls on PM Lee and other leaders | Marine Aquaculture | Singapore | 2010 | 0 |
| Trade deficit with S'pore has to be addressed: Cambodia | Marine Aquaculture | Singapore | 2010 | 0 |
| Budget Speech | Marine Aquaculture | India | 2010 | 0 |
| Buoyant Q4, full year for Oceanus; Key factors: fair value gains, higher sales, absence of goodwill write-off | Marine Aquaculture | Singapore | 2010 | 1 |
| Meet the super sea bass; Farmed-in-Singapore barramundi makes a splash as an alternative to regular sea bass | Marine Aquaculture | Australia | 2010 | 1 |
| Buying among directors up, selling remains low | Marine Aquaculture | Singapore | 2010 | 0 |
| Fisheries in a flap | Marine Aquaculture | South Africa | 2010 | 0 |
| Marine farms a risk for boaties | Marine Aquaculture | New Zealand | 2010 | -1 |
| 200,000 fish in farms off Pasir Ris dead; Plankton bloom causes losses reaching hundreds of thousands of dollars | Marine Aquaculture | Singapore | 2010 | -1 |
| Cost-effective, eco-friendly feed for marine farming | Marine Aquaculture | Malaysia | 2009 | 1 |
| A quiet session ahead of year-end holidays | Marine Aquaculture | Singapore | 2009 | 0 |
| Moving of government furniture all but complete; New departments, others split | Marine Aquaculture | South Africa | 2009 | 0 |
| More S'pore farmed fish on menu; Republic's largest commercial fish farm a sign of things to come | Marine Aquaculture | Singapore | 2009 | 1 |
| Bangladesh:Oceans would be able to feed growing world population in future | Marine Aquaculture | Pakistan | 2009 | 1 |
| UNCW to pay more for facility | Marine Aquaculture | United States | 2009 | 0 |
| Barr wins bid to build GBP11m fish study centre in Aberdeen | Marine Aquaculture | Scotland | 2009 | 0 |
| Injured turtle released back into the wild | Marine Aquaculture | Cyprus | 2009 | 1 |
| Heat is on for school project | Marine Aquaculture | Australia | 2009 | 0 |
| Wanted: tough outdoor types up to challenge of extreme fish farming Staff will live offshore on 'iceberg' barges | Marine Aquaculture | Scotland | 2009 | 0 |
| Borneo Aqua Harvest will continue to uptrend | Marine Aquaculture | Malaysia | 2009 | 1 |
| Rules Guiding Fish Farming In the Gulf Are Readied | Marine Aquaculture | United States | 2009 | 0 |
| Family split threatens success of abalone farm | Marine Aquaculture | South Africa | 2009 | 0 |
| School challenge - ABC to PHD: Advertising feature | Marine Aquaculture | Australia | 2009 | 0 |
| Youth In Print Experiments with science | Marine Aquaculture | Australia | 2009 | 0 |
| Prawn secrets to be probed | Marine Aquaculture | Australia | 2009 | -1 |
| Co-op gives local fish stocks a boost; Five fish farms here link up and 10 others keen to join cooperative | Marine Aquaculture | Singapore | 2009 | 1 |
| Cambodia to get over 33m developmental aid from Japan | Marine Aquaculture | Cambodia | 2009 | 1 |
| Director, institutional shareholder activity flat | Marine Aquaculture | Singapore | 2009 | 0 |
| Evidence of fish farm risks to salmon mounts | Marine Aquaculture | Canada | 2009 | -1 |
| Mississippi lawmakers approve funding | Marine Aquaculture | United States | 2009 | 1 |
| Award for Loch Roag system | Marine Aquaculture | Scotland | 2009 | 0 |
| Flat trading by directors; Buyback, shareholder activity also flat, but the week sees several significant filings by institutional shareholders, reports ROBERT HALILI | Marine Aquaculture | Singapore | 2009 | 0 |
| Shetland success at annual aquaculture industry accolades | Marine Aquaculture | Scotland | 2009 | 1 |
| Developer adamant to progress Trinity Wharf plan | Marine Aquaculture | Ireland | 2009 | 0 |
| Farmers hail the raw prawn | Marine Aquaculture | Australia | 2009 | 1 |
| Plague of 'jackets | Marine Aquaculture | Australia | 2009 | -1 |
| Big V for Vino at Sydney show | Marine Aquaculture | Australia | 2009 | 0 |
| open season | Marine Aquaculture | Australia | 2009 | 0 |
| Major marine scientific expedition off Sabah | Marine Aquaculture | Australia | 2009 | 0 |
| Sea's fruits are from the farm | Marine Aquaculture | Australia | 2009 | 1 |
| M&F shows signs of catching governance bug | Marine Aquaculture | South Africa | 2009 | -1 |
| Lawmakers hear about aquaculture; UNCW officials, fish farmers talk to new committee in Raleigh | Marine Aquaculture | United States | 2009 | 0 |
| Prawn farmer in line for show's major award | Marine Aquaculture | Australia | 2009 | 1 |
| Easter prawns priced too high - Seafood chief says shops not passing on savings | Marine Aquaculture | Australia | 2009 | -1 |
| Abandoned turtle rescued | Marine Aquaculture | Cyprus | 2009 | 1 |
| Fund manager may inject US $15m into Oceanus | Marine Aquaculture | Singapore | 2009 | 1 |
| Va. agricultural exports soar 27 percent in 2008; The value of the state's crops rises to $2.22 billion as variety pays dividends | Marine Aquaculture | United States | 2009 | 0 |
| Treating wild and farmed salmon equally | Marine Aquaculture | Canada | 2009 | 1 |
| Huon tempts your appetite | Marine Aquaculture | Australia | 2009 | 1 |
| Joint resource dev't pushed | Marine Aquaculture | Philippines | 2009 | 1 |
| Minister unscrupulous and callous - perlemoen fishers | Marine Aquaculture | South Africa | 2009 | -1 |
| 'government fails to deliver' | Marine Aquaculture | South Africa | 2009 | -1 |
| Yellowtail ranching project to help fishermen could miss boat | Marine Aquaculture | South Africa | 2009 | -1 |
| Â£300,000 expansion plans | Marine Aquaculture | United Kingdom | 2009 | 1 |
| Fishermen hope for Gregg s help at Commerce post | Marine Aquaculture | United States | 2009 | 0 |
| Seller thought zoo could handle cow nose rays; 'We assumed there was a level of expertise' | Marine Aquaculture | Canada | 2009 | 0 |
| Gulf No Place For Fish Farms | Marine Aquaculture | United States | 2009 | -1 |
| FISHING for FUNDS; FISH HATCHERY PLAN: Bid for stimulus cash could help Mote Marine grow | Marine Aquaculture | United States | 2009 | 1 |
| EW plan gives aqua-farmers their chances | Marine Aquaculture | New Zealand | 2008 | 1 |
| Johor to produce organic food | Marine Aquaculture | Malaysia | 2008 | 0 |
| Abalone dilemma | Marine Aquaculture | South Africa | 2008 | -1 |
| Big Splash; Some city-bred Singapore entrepreneurs have fallen hook , line and sinker for the simple life in kelongs | Marine Aquaculture | Singapore | 2008 | 0 |
| B.C.'s salmon farmers given a free ride by our provincial 'regulators' | Marine Aquaculture | Canada | 2008 | -1 |
| R.I. Tree Council to host Beijing university professor | Marine Aquaculture | United States | 2008 | 0 |
| GOLD COAST HONOURS 2008 All our finalists | Marine Aquaculture | Australia | 2008 | 0 |
| GOLD COAST HONOURS NOMINEES | Marine Aquaculture | Australia | 2008 | 0 |
| Prawns sweeter than sugarcane | Marine Aquaculture | Australia | 2008 | 1 |
| Special panel studying nursing shortage in R.I. | Marine Aquaculture | United States | 2008 | 0 |
| Aquaculture facilities get funds to expand; Beals, Franklin sites of research centers | Marine Aquaculture | United States | 2008 | 1 |
| Anglers condemn debris left behind by fish farms giant Marine Harvest | Marine Aquaculture | Scotland | 2008 | -1 |
| Tiger prawns earn stripes | Marine Aquaculture | Australia | 2008 | 1 |
| From the kelong to your table - the cobia; Premium fish used in yusheng now farmed here as part of AVA's project to cut reliance on imports | Marine Aquaculture | Singapore | 2008 | 1 |
| Affordable skin-on fillets that'll stand up to grilling | Marine Aquaculture | United States | 2008 | 1 |
| Fish farms offer hope of cheap easy catch | Marine Aquaculture | Australia | 2008 | 1 |
| Revolutionary Bio-Energy From Algae | Marine Aquaculture | Korea | 2008 | 1 |
| Pressure groups say poor are getting a raw deal from government's abalone strategy | Marine Aquaculture | South Africa | 2008 | -1 |
| Climate change to impact on fisheries | Marine Aquaculture | Scotland | 2008 | -1 |
| Oceanus expansion plans may lead to dividend payouts | Marine Aquaculture | Singapore | 2008 | 1 |
| Fish escape prompts new calls for change; Critics contend only closed-containment will repair problem | Marine Aquaculture | Canada | 2008 | -1 |
| Joy: Ben aims to do swimmingly in dream job | Marine Aquaculture | United Kingdom | 2008 | 0 |
| Marine college student lands dream job with resort aquarium; A cornwall College student has landed his dream job - before completing his own degree. | Marine Aquaculture | United Kingdom | 2008 | 0 |
| Environmental program open to public | Marine Aquaculture | United States | 2008 | 0 |
| 'Great expectations'; Aquaculture facility in Franklin celebrates mission to raise quality salmon | Marine Aquaculture | United States | 2008 | 1 |
| Vying for awards in aquaculture sector | Marine Aquaculture | Scotland | 2008 | 0 |
| Oceanus makes Catalist debut today | Marine Aquaculture | Singapore | 2008 | 0 |
| SEAFOOD DEAL $100m U.S. export bonanza Eyre abalone coup | Marine Aquaculture | Australia | 2008 | 1 |
| Post-war pact to the rescue of coastguard | Marine Aquaculture | Ireland | 2008 | 0 |
| Luncheon appealing | Marine Aquaculture | Australia | 2008 | 0 |
| Awards scheme will now cover entire aquaculture sector | Marine Aquaculture | Scotland | 2008 | 1 |
| Lunch draws crowd | Marine Aquaculture | Australia | 2008 | 0 |
| Career Beacon.com job listings | Marine Aquaculture | Canada | 2008 | 0 |
| Marine farm plans sunk | Marine Aquaculture | New Zealand | 2008 | -1 |
| Group critical of salmon farming impact on Bay; Conservation group say more stringent regulations, policies needed to protect Bay of Fundy | Marine Aquaculture | Canada | 2008 | -1 |
| Tagging the big fish | Marine Aquaculture | Australia | 2008 | 0 |
| Q&A with Dr. Martha Dunagin Saunders, U of Southern Mississippi president | Marine Aquaculture | United States | 2008 | 0 |
| Prawn lunch | Marine Aquaculture | Australia | 2008 | 0 |
| Under microscope | Marine Aquaculture | Australia | 2008 | 0 |
| Black fishing investors face stormy waters | Marine Aquaculture | South Africa | 2008 | -1 |
| Adventurer found success on land and sea | Marine Aquaculture | United States | 2008 | 1 |
| Fishing | Marine Aquaculture | South Africa | 2008 | 0 |
| Danger signal - alien invader threatens salmon, trout stocks | Marine Aquaculture | United Kingdom | 2007 | -1 |
| UNLIKELY ALLIES TEAM UP AGAINST FISH FARMING AT HEARING | Marine Aquaculture | United States | 2007 | -1 |
| UNLIKELY ALLIES OPPOSE GULF FISH FARMING | Marine Aquaculture | United States | 2007 | -1 |
| Bright sparks lock onto 'Genera onto 'Generation Y'; Two new universities are set to feel the benefit of a scheme to harness the talents of bright young things in business, says Stephen Naysmith | Marine Aquaculture | Scotland | 2007 | 0 |
| Open ocean farms could feed the world; Food: Official at aquaculture conference warns we need a replacement for dwindling fish stocks | Marine Aquaculture | Canada | 2007 | 1 |
| Support for marine management plan | Marine Aquaculture | New Zealand | 2007 | 1 |
| Philippines poised to revisit expired fishing pacts with Indonesia | Marine Aquaculture | Philippines | 2007 | 0 |
| Sea projects to soften ban on abalone | Marine Aquaculture | South Africa | 2007 | 1 |
| Government to inject R100m into marine fish farms | Marine Aquaculture | South Africa | 2007 | 1 |
| Ban postponed - but abalone court bid set to go ahead | Marine Aquaculture | South Africa | 2007 | -1 |
| 'real issue is perlemoen poachers' | Marine Aquaculture | South Africa | 2007 | -1 |
| A chance to mussel in on aquaculture; Shellfish farming company's empowerment project offers nine fishermen a fresh start | Marine Aquaculture | South Africa | 2007 | 1 |
| Fishing pacts with Pacific nations sought | Marine Aquaculture | Philippines | 2007 | 0 |
| Fingerlings to finger-licking good | Marine Aquaculture | Singapore | 2007 | 1 |
| S'pore's very own super sea bass; Hatched in AVA's research tanks, the fast-growing, hardy fry were fattened on an offshore farm in Riau | Marine Aquaculture | Singapore | 2007 | 1 |
| Tax breaks for aqua farmers, investors | Marine Aquaculture | South Africa | 2007 | 1 |
| Plan to encourage investment in aquaculture | Marine Aquaculture | South Africa | 2007 | 1 |
| FAO SUPPORTS REHABILITATION OF AGRICULTURAL, LIVESTOCK, FISHERIES AND LIVELIHOODS OF \ CYCLONE AFFECTED COMMUNITIES OF BALOCHISTAN | Marine Aquaculture | Pakistan | 2007 | 1 |
| B.c. Salmon industry in rough water; Agriculture; Skilled workers cut bait rather than face the 'politics' | Marine Aquaculture | Australia | 2007 | -1 |
| Show gets bigger | Marine Aquaculture | Australia | 2007 | 0 |
| Hotel's student contract | Marine Aquaculture | United Kingdom | 2007 | 0 |
| Highlights of Northern Corridor Economic Region (NCER) | Marine Aquaculture | Malaysia | 2007 | 0 |
| Popular course | Marine Aquaculture | United Kingdom | 2007 | 0 |
| Imported raw prawns in risk to marine life | Marine Aquaculture | Australia | 2007 | -1 |
| Salt study boon for abalone | Marine Aquaculture | Australia | 2007 | 1 |
| UPSHOT; Chinese economic invasion | Marine Aquaculture | China | 2007 | 0 |
| Tiny freshwater creatures can grow into tasty delights far from any ocean; Raising shrimp is big business in Ohio ponds | Marine Aquaculture | United States | 2007 | 1 |
| MOTE'S RACE AGAINST THE CLOCK; Rush is on to complete rebuilding of fire-damaged sturgeon facility | Marine Aquaculture | United States | 2007 | 0 |
| Anti fish farm ads take aim at grocery chain | Marine Aquaculture | Canada | 2007 | -1 |
| A barefoot boy's; golden harvest | Marine Aquaculture | Australia | 2007 | 1 |
| Seaweed farming points the way for food production | Marine Aquaculture | South Africa | 2007 | 1 |
| Kleinschmidt honored for AquaPod project | Marine Aquaculture | United States | 2007 | 1 |
| Courses on offer | Marine Aquaculture | United Kingdom | 2007 | 1 |
| Climate change threatens tiny krill, leaving Antarctic ecosystem at risk | Marine Aquaculture | United States | 2007 | -1 |
| US fishes for subsidy support | Marine Aquaculture | South Africa | 2007 | 0 |
| Fish farm group will trawl for youngsters | Marine Aquaculture | Australia | 2007 | 0 |
| Fishy find: Children discover unusual visitor | Marine Aquaculture | United Kingdom | 2007 | 0 |
| Warm-water puffer fish found on beach | Marine Aquaculture | United Kingdom | 2007 | 0 |
| Industry prepares for future | Marine Aquaculture | Australia | 2007 | 1 |
| Aquaculture growing dramatically here and worldwide | Marine Aquaculture | United States | 2007 | 1 |
| Stop foreign poaching | Marine Aquaculture | South Africa | 2007 | 0 |
| Something fishy | Marine Aquaculture | South Africa | 2007 | -1 |
| Small-scale effort to beat fishery blues | Marine Aquaculture | South Africa | 2007 | 0 |
| Center to target salmon research; Genetics focus of new facility in Franklin | Marine Aquaculture | United States | 2007 | 0 |
| Feast of prawns worthy of seconds | Marine Aquaculture | Australia | 2007 | 1 |
| Locals protest plans for fish farm | Marine Aquaculture | Nova Scotia | 2007 | -1 |
| (Briefs) | Marine Aquaculture | United States | 2007 | 0 |
| Prawns, prawns, glorious prawns | Marine Aquaculture | Australia | 2007 | 1 |
| Extra long lunch to cater for demand; Prawns net a crowd | Marine Aquaculture | Australia | 2007 | 1 |
| Prawns on the move | Marine Aquaculture | Australia | 2007 | 0 |
| Prawn prices face rise on import blitz | Marine Aquaculture | Australia | 2007 | 0 |
| World Wetlands Day; Guided tour of Okato reserve | Marine Aquaculture | New Zealand | 2007 | 0 |
| Ahern announces EUR 600m fisheries plan | Marine Aquaculture | Ireland | 2007 | 0 |
| Letters | Marine Aquaculture | United States | 2007 | 0 |
| Fish farm death dive 'broke all rules' | Marine Aquaculture | United Kingdom | 2007 | -1 |
| Action needed on aquaculture | Marine Aquaculture | Australia | 2007 | -1 |
| Say on aquaculture welcome | Marine Aquaculture | Australia | 2007 | 0 |
| Farmed fish swim to the fore; How we handle aquaculture may determine the future of the planet | Marine Aquaculture | United States | 2007 | 1 |
| Far more choice than the three Rs; BACK TO SCHOOL - A Newcastle Herald Advertising Feature | Marine Aquaculture | Australia | 2007 | 0 |
| IN BRIEF... | Marine Aquaculture | Australia | 2007 | 0 |
| Fish farms in the ocean? Group pushes Congress to pass tough rules. | Marine Aquaculture | United States | 2007 | -1 |
| Aquaculture report urges growth, better regulation | Marine Aquaculture | United States | 2007 | 1 |
| LEGAL NOTICES - 17 OF 17 | Marine Aquaculture | United States | 2006 | 0 |
| St John's Island | Marine Aquaculture | Singapore | 2006 | 0 |
| LETTERS TO THE EDITOR | Marine Aquaculture | Australia | 2006 | 0 |
| Guyana to set up fisheries committee | Marine Aquaculture | Guyana | 2006 | 0 |
| Teens stuck at sea as trip goes wrong | Marine Aquaculture | Australia | 2006 | 0 |
| Wildlife learning centre | Marine Aquaculture | United Kingdom | 2006 | 0 |
| 5 things you didn't know about... | Marine Aquaculture | Singapore | 2006 | 0 |
| Maritime opportunity | Marine Aquaculture | Australia | 2006 | 1 |
| Campus' size has doubled | Marine Aquaculture | United Kingdom | 2006 | 0 |
| P5-M fisheries lab to be set up in GenSan | Marine Aquaculture | Philippines | 2006 | 0 |
| Shop wisely or 'go fish' | Marine Aquaculture | Australia | 2006 | 0 |
| College staff and students inspect new building | Marine Aquaculture | United Kingdom | 2006 | 0 |
| Students get hands-on reservoir experience | Marine Aquaculture | United Kingdom | 2006 | 0 |
| College presents its zoological research | Marine Aquaculture | United Kingdom | 2006 | 0 |
| Fish farm educational hook for keen students | Marine Aquaculture | Australia | 2006 | 1 |
| Students are hooked on harvesting fish | Marine Aquaculture | Australia | 2006 | 1 |
| College welcomes new headteacher to campus | Marine Aquaculture | United Kingdom | 2006 | 0 |
| SA 'aims to be one of top 10 destinations' | Marine Aquaculture | South Africa | 2006 | 0 |
| friday fishing | Marine Aquaculture | Australia | 2006 | 0 |
| Pioneering project to help the king of fish | Marine Aquaculture | United Kingdom | 2006 | 1 |
| State losing ground in bioscience jobs | Marine Aquaculture | United States | 2006 | -1 |
| Farm lands prawn solution | Marine Aquaculture | Australia | 2006 | 1 |
| Namibian president urges Swedish businessmen to invest in country | Marine Aquaculture | Namibia | 2006 | 0 |
| in the zone | Marine Aquaculture | South Africa | 2006 | 0 |
| Lean pickings under new marine farm laws | Marine Aquaculture | New Zealand | 2006 | -1 |
| Course recruits sample life beneath the waves | Marine Aquaculture | United Kingdom | 2006 | 0 |
| The fishing's fine at Northam | Marine Aquaculture | Australia | 2006 | 0 |
| Funds for marine science students | Marine Aquaculture | Australia | 2006 | 0 |
| Govt pulls plug on Port fish farm | Marine Aquaculture | Australia | 2006 | -1 |
| Cawthron role `exciting challenge' | Marine Aquaculture | New Zealand | 2006 | 0 |
| Sea food, just; had to eat it | Marine Aquaculture | Australia | 2006 | 0 |
| Malaysian firm helps turn Namibia around | Marine Aquaculture | Malaysia | 2006 | 1 |
| Blueprint to beat skills shortage | Marine Aquaculture | Australia | 2006 | 0 |
| Zoo gains training praise | Marine Aquaculture | United Kingdom | 2006 | 0 |
| Students uncover undersea world of opportunities | Marine Aquaculture | Canada | 2006 | 1 |
| Western Isles salmon firm to trial greener production | Marine Aquaculture | Scotland | 2006 | 1 |
| College bids to expand with new Â£1.9m centre | Marine Aquaculture | United Kingdom | 2006 | 0 |
| Seafood trail map | Marine Aquaculture | New Zealand | 2006 | 0 |
| Plan for organic farm joint venture in Pangkor | Marine Aquaculture | Malaysia | 2006 | 0 |
| LEGAL NOTICES - 3 OF 3 | Marine Aquaculture | United States | 2005 | 0 |
| Scientists win $10,000 grant | Marine Aquaculture | Australia | 2005 | 0 |
| Expert touts plan to boost R&D spending | Marine Aquaculture | United States | 2005 | 0 |
| Claims of benefits to raising fish in sea are full of snags | Marine Aquaculture | United States | 2005 | -1 |
| Company Briefs | Marine Aquaculture | Singapore | 2005 | 0 |
| Corea Heath changes hands in land transfer | Marine Aquaculture | United States | 2005 | 0 |
| Red tape sinks tuna farm plan | Marine Aquaculture | Australia | 2005 | -1 |
| Tuna off the menu in Esperance | Marine Aquaculture | Australia | 2005 | -1 |
| Rising student levels spark expansion plan | Marine Aquaculture | United Kingdom | 2005 | 0 |
| HOLD THAT PROJECT;As fuel and material costs spiral upward, a winter hiatus can yield big savings for builders and clients | Marine Aquaculture | United States | 2005 | -1 |
| Oceanus in research tie-up with NUS body;MOU to further aquaculture research in Singapore | Marine Aquaculture | Singapore | 2005 | 1 |
| Chinese fish-breeder casts IPO net towards Singapore;Oceanus lodges prospectus with the central bank for a mainboard listing | Marine Aquaculture | Singapore | 2005 | 0 |
| Mussel farm at Eastern Bay discussed; Decision on proposal by Tenants Harbor firm delayed so flats can be studied | Marine Aquaculture | United States | 2005 | 0 |
| Oak Hill pines to annex southern parcel | Marine Aquaculture | United States | 2005 | 0 |
| A healthy dose of IT | Marine Aquaculture | Australia | 2005 | 0 |
| GOING FOR LISTING: Borneo Aqua expanding | Marine Aquaculture | Malaysia | 2005 | 0 |
| All Silver and Smiles: Aquaculture brings hope, economic stability to remote Klemtu and its First Nations residents | Marine Aquaculture | Canada | 2005 | 1 |
| Fish farms stay under microscope: Industry has its share of supporters and detractors. In B.C., 4,000 workers earn a living in aquaculture. | Marine Aquaculture | Canada | 2005 | -1 |
| THINK TANK | Marine Aquaculture | Australia | 2005 | 0 |
| Ocean aquaculture | Marine Aquaculture | United States | 2005 | 0 |
| Radical Bush plan boosts B.C. farmed fish industry: Cod, halibut, red snapper to be raised | Marine Aquaculture | Canada | 2005 | 1 |
| Confidence in aquaculture | Marine Aquaculture | Australia | 2005 | 1 |
| Breeder plans 10 fish farms off Ubin;Swee Chioh Aquaculture's move to yield 500 tonnes of fish annually for local market | Marine Aquaculture | Singapore | 2005 | 1 |
| Judging who's the big cheese | Marine Aquaculture | Australia | 2005 | 0 |
| New shellfish land will need additional rules | Marine Aquaculture | Canada | 2005 | -1 |
| Fisheries college jobs to go after backing reduced | Marine Aquaculture | Scotland | 2005 | 0 |
| Sustainable seafood; Marine life ECO | Marine Aquaculture | Australia | 2004 | 0 |
| Research project aims to dispel fears over Scottish farmed salmon | Marine Aquaculture | Scotland | 2004 | 1 |
| Business: Call to tap into lucrative fish-farm industry;Use aquaculture know-how to expand into the region to meet rising global demand for fish, says minister | Marine Aquaculture | Singapore | 2004 | 1 |
| Holy flounder; Tank-raised halibut, ugly yet valuable, could pad Mainer's pocketbooks | Marine Aquaculture | United States | 2004 | 1 |
| $30m fund to take inventions to market;Entrepreneurial arm of NUS aims to help 100 promising start-ups in the next three to five years, including foreign ones hatched here | Marine Aquaculture | Singapore | 2004 | 1 |
| Small fish big bucks;Fish farming goes from sunset industry to knowledge-driven sector | Marine Aquaculture | Singapore | 2004 | 1 |
| Get bitten by research bug | Marine Aquaculture | Malaysia | 2004 | 0 |
| Tech@Work: All the right connections | Marine Aquaculture | Singapore | 2004 | 0 |
| Making its mark at home and abroad | Marine Aquaculture | Malaysia | 2004 | 0 |
| Helping to re-stock the camel | Marine Aquaculture | United Kingdom | 2004 | 0 |
| AVA-bred fish to go on sale in stores here;The first batch of pompano is from deep net-cage farms, which yield more than other coastal fish farms | Marine Aquaculture | Singapore | 2004 | 1 |
| Curtin broadens its appeal | Marine Aquaculture | Australia | 2004 | 0 |
| Oyster paradise | Marine Aquaculture | Australia | 2004 | 1 |
| Singapore global business fair will be annual affair;Spurred by success of inaugural Global Entrepolis last year, EDB hopes to see 25,000 participants at this year's event | Marine Aquaculture | Singapore | 2004 | 0 |
| Scientists show invasion of the sea creatures;A survey reveals 18 non-native species in just three places on Casco Bay. | Marine Aquaculture | United States | 2004 | -1 |
| Camel gets :2m clean-up | Marine Aquaculture | United Kingdom | 2004 | 0 |
| Aquaculture set to scale new heights | Marine Aquaculture | Australia | 2004 | 1 |
| :2m clean-up of river after lobster deaths | Marine Aquaculture | United Kingdom | 2004 | -1 |
| Ocean report has long reach | Marine Aquaculture | United States | 2004 | 0 |
| UMS courses relevant to nation's need | Marine Aquaculture | Malaysia | 2004 | 0 |
| S'pore should be angling for more bucks in fish trade | Marine Aquaculture | Singapore | 2004 | 0 |
| Lawmakers to consider aquaculture's future | Marine Aquaculture | United States | 2004 | 0 |
| UMS makes waves globally | Marine Aquaculture | Malaysia | 2004 | 0 |
| Overseas investors snap up fish farm after failure to make NSX | Marine Aquaculture | Australia | 2003 | 1 |
| Across The West | Marine Aquaculture | United Kingdom | 2003 | 0 |
| University Campus Opens | Marine Aquaculture | United Kingdom | 2003 | 0 |
| Research plays important role at KUSTEM | Marine Aquaculture | Malaysia | 2003 | 0 |
| UCC appoints new dean of math, tech | Marine Aquaculture | Canada | 2003 | 0 |
| Row Flares Over Fish Unit'S Future | Marine Aquaculture | Scotland | 2003 | -1 |
| Canadian firms hired to analyze aquaculture | Marine Aquaculture | United States | 2003 | 0 |
| Plenty of fish in the sea? Think again...;Fishermen and consumers are feeling the pinch as fish stocks are depleted through over-fishing | Marine Aquaculture | Singapore | 2003 | 0 |
| Only nine but UMS forges ahead | Marine Aquaculture | Malaysia | 2003 | 0 |
| Hopes Rise For Future Of Ardtoe Marine Research Unit | Marine Aquaculture | Scotland | 2003 | 0 |
| Schools' learning curve - Biggest curriculum shake-up for 30 years | Marine Aquaculture | Australia | 2003 | 0 |
| New marine aquaculture goes after bigger fish | Marine Aquaculture | Singapore | 2003 | 1 |
| Equally delicious, steamed or fried | Marine Aquaculture | Singapore | 2003 | 1 |
| Small fry? Think again...it's big plan to feed S'poreans;High-tech breeding facility on St John's Island aims to produce almost half the fish people eat here | Marine Aquaculture | Singapore | 2003 | 1 |
| Healthy oceans Pew report signals need for further remedies | Marine Aquaculture | United States | 2003 | -1 |
| TOPICS TODAY | Marine Aquaculture | Australia | 2003 | 0 |
| Port Ed hatchery closes its doors: q Marine Harvest Canada mothballs state-of-art $8 million facility; blames feds, misinformation | Marine Aquaculture | Canada | 2003 | -1 |
| Government grants | Marine Aquaculture | Australia | 2003 | 0 |
| Blair Is Dragged Into Ardtoe Row | Marine Aquaculture | Scotland | 2003 | 0 |
| Fish farming for Bay sites | Marine Aquaculture | Australia | 2003 | 0 |
| Investors lured by fish farm shares | Marine Aquaculture | Australia | 2003 | 1 |
| Ubin prawns going places;Farm expanding to become fish hatchery, as well as producing new dried prawn snack using small prawns no one wants | Marine Aquaculture | Singapore | 2003 | 1 |
| Better chance for say | Marine Aquaculture | Australia | 2002 | 0 |
| Plan for abalone farm put on show | Marine Aquaculture | Australia | 2002 | 1 |
| UE unit clinches $200m PUB contract | Marine Aquaculture | Singapore | 2002 | 0 |
| Raise aquaculture production, marine scientists urged | Marine Aquaculture | Malaysia | 2002 | 1 |
| Fish farm in $2.5m listing bid | Marine Aquaculture | Australia | 2002 | 0 |
| Breaking away from the herd | Marine Aquaculture | Australia | 2002 | 0 |
| Ofex wants to challenge ASX with trans-Tasman market; Investing | Marine Aquaculture | Australia | 2002 | 0 |
| Regional firms in the limelight; Hunter Region Export Awards 2002 A Newcastle Herald Advertising Feature | Marine Aquaculture | Australia | 2002 | 0 |
| Aquaculture industry still a zone of contention | Marine Aquaculture | Australia | 2002 | -1 |
| WHAT A WHOPPER!;ANGLESEY NETS POUNDS 19M EURO FISH FARM DEAL | Marine Aquaculture | Wales | 2002 | 1 |
| In step with the community | Marine Aquaculture | Malaysia | 2002 | 0 |
| Transforming the image - CENTRAL COAST: MOVING FORWARD | Marine Aquaculture | Australia | 2002 | 0 |
| Cod trials' results | Marine Aquaculture | Scotland | 2002 | 0 |
| Taking aquaculture and marine science to new heights | Marine Aquaculture | Malaysia | 2002 | 1 |
| Primelife chief hits back over `foul play'; MANAGEMENT | Marine Aquaculture | Australia | 2001 | 0 |
| Something's fishy on this farm | Marine Aquaculture | Japan | 2001 | -1 |
| Marine research centre floated | Marine Aquaculture | Canada | 2001 | 0 |
| Farmers hatch a new plan that may well grow on us; FISHING | Marine Aquaculture | Australia | 2001 | 1 |
| Farm-fresh fish on menu;Banker becomes snapper breeder | Marine Aquaculture | Australia | 2001 | 1 |
| Fortune begins to favour the salmon;The reel thing | Marine Aquaculture | Scotland | 2001 | 1 |
| Two schools of thought on fish farm go-ahead | Marine Aquaculture | Australia | 2001 | 0 |
| Plan for deep-sea fish farming hits snag | Marine Aquaculture | Singapore | 2001 | -1 |
| Fishing for more | Marine Aquaculture | Malaysia | 2001 | 0 |
| Business People | Marine Aquaculture | United States | 2001 | 0 |
| Fears spark crusade to 'save' Lough Swilly;New group will battle fish farming plans | Marine Aquaculture | United Kingdom | 2001 | -1 |
| Anglers bite at fish park bid | Marine Aquaculture | Australia | 2001 | -1 |
| Agency to tackle fish-farm waste | Marine Aquaculture | Scotland | 2001 | -1 |
| New aquaculture guidelines urged | Marine Aquaculture | Scotland | 2001 | -1 |
| Aquaculture production to be raised six-fold | Marine Aquaculture | Malaysia | 2001 | 1 |
| Aquaculture plans snagged by red tape; SEAFOOD | Marine Aquaculture | Australia | 2000 | -1 |
| Primed for prawns | Marine Aquaculture | Australia | 2000 | 1 |
| Offshore investment hooks ex-banker | Marine Aquaculture | Australia | 2000 | 1 |
| Seafood body slams marine park proposal; SEAFOOD | Marine Aquaculture | Australia | 2000 | -1 |
| AVA -- that's PPD's name as a stat board | Marine Aquaculture | Singapore | 2000 | 0 |
| Tassie divers' safety splash | Marine Aquaculture | Australia | 2000 | 0 |
| Justice and democracy will disappear | Marine Aquaculture | United States | 2000 | 0 |
| WHAT'S NEW; VICTORIA | Marine Aquaculture | Australia | 1999 | 0 |
| Leisure and tourism will become more important in the economy of the South West | Marine Aquaculture | United Kingdom | 1999 | 0 |
| 1999 ROYAL ADELAIDE SHOW; A pearl of great price | Marine Aquaculture | Australia | 1999 | 0 |
| Move to boost fish, vegetable supply | Marine Aquaculture | Singapore | 1999 | 1 |
| Asia claws back onto the local economic agenda | Marine Aquaculture | Australia | 1999 | 0 |
| Cutting red tape on fish farming; VICTORIA - SEAFOOD | Marine Aquaculture | Australia | 1999 | 1 |
| A project going swimmingly / THE HUNTER REGION | Marine Aquaculture | Australia | 1999 | 1 |
| Council nets fish farms | Marine Aquaculture | Australia | 1999 | 0 |
| Sorry, tuna is calling the tune | Marine Aquaculture | Australia | 1999 | 0 |
| A state with bigger fish to fry; SEAFOOD | Marine Aquaculture | Australia | 1999 | 0 |
| A city fights back | Marine Aquaculture | Australia | 1999 | -1 |
| Sarawak optimistic about robust economic prospects | Marine Aquaculture | Malaysia | 1999 | 1 |
| Barbed wire, fences go up at St John's | Marine Aquaculture | Singapore | 1999 | 0 |
| Your views | Marine Aquaculture | Canada | 1999 | 0 |
| A snapper of the fingerlings | Marine Aquaculture | Australia | 1999 | 0 |
| St John's to have marine research centre | Marine Aquaculture | Singapore | 1999 | 0 |
| Fish farmers seek clarity on marine park plans; VICTORIA | Marine Aquaculture | Australia | 1999 | -1 |
| Sea cages yield first commercial harvests; FISHING | Marine Aquaculture | Australia | 1998 | 1 |
| POLITICS: BLAIR POISED TO MEET TRIMBLE FOR POLICY TALKS | Marine Aquaculture | Ireland | 1998 | 0 |
| Blair unveils UK-Irish body in Dublin | Marine Aquaculture | United Kingdom | 1998 | 0 |
| Blair to unveil UK-Irish forum | Marine Aquaculture | United Kingdom | 1998 | 0 |
| BRUNSWICK PEOPLE / Douglas Holland; Expert cultivates fish farming | Marine Aquaculture | United States | 1998 | 1 |
| Industry, nature can co-exist | Marine Aquaculture | United States | 1998 | 1 |
| Our tainted coast; Report pinpoints pollution and the;solution | Marine Aquaculture | Australia | 1998 | -1 |
| Fortune in flying fish | Marine Aquaculture | Australia | 1998 | 0 |
| Peace Corps Worker Killed in Philippines | Marine Aquaculture | United States | 1998 | 0 |
| NEWS IN BRIEF | Marine Aquaculture | Philippines | 1998 | 0 |
| Mum, can we have fish? | Marine Aquaculture | Singapore | 1998 | 0 |
| Call to release secret report on aquacentreDecision to close college has been hotly disputed | Marine Aquaculture | Scotland | 1998 | 0 |
| NBT continues focus on core areas | Marine Aquaculture | Malaysia | 1998 | 0 |
| Fishing for complements | Marine Aquaculture | Australia | 1997 | 0 |
| Norwegian study of our aquaculture potential | Marine Aquaculture | Malaysia | 1997 | 1 |
| Lucas Heights | Marine Aquaculture | Australia | 1997 | 0 |
| Business groups main goal of growth study | Marine Aquaculture | Australia | 1997 | 0 |
| AS AGE OF AQUACULTURE DAWNS, WE CAN LEARN FROM OTHERS' MISTAKES; VIGILANCE OVER DISEASE AND NUTRIENT POLLUTION ARE ESSENTIAL. | Marine Aquaculture | United States | 1997 | 0 |
| Fiji aims for increase in visitors | Marine Aquaculture | Australia | 1997 | 0 |
| Tuna farms 'threat' to KI penguins | Marine Aquaculture | Australia | 1997 | -1 |
| State inquiry says no to oyster farms | Marine Aquaculture | Australia | 1996 | -1 |
| Opportunities in global halal' market | Marine Aquaculture | Malaysia | 1996 | 1 |
| Minister with a true flair for stating the blindingly obvious | Marine Aquaculture | Ireland | 1996 | 0 |
| A mixture of satisfaction and fear | Marine Aquaculture | Malaysia | 1996 | 0 |
| Fish that are reared to be main source of supply | Marine Aquaculture | Malaysia | 1996 | 0 |
| Agency turns ideas into cash for coast A million clams will make them as happy as . . . | Marine Aquaculture | United States | 1996 | 1 |
| Breeding grouper on the farm | Marine Aquaculture | Singapore | 1996 | 1 |
| Promoting R & D | Marine Aquaculture | Singapore | 1996 | 0 |
| Mystery virus attack | Marine Aquaculture | Singapore | 1996 | -1 |
| BALLAST WATER ORGANISMS AN ENVIRONMENTAL THREAT | Marine Aquaculture | Australia | 1996 | -1 |
| IVF WHITING RESEARCH TO HELP DECLINING NUMBERS | Marine Aquaculture | Australia | 1996 | 0 |
| Oysters hailed as solution to feeding Africa's hungry | Marine Aquaculture | Canada | 1996 | 1 |
| Aquaculture centre officially opened | Marine Aquaculture | Australia | 1996 | 1 |
| Intellectual Property India Publishes Patent Application for 'Offshore Aquaculture System' Filed by Sea Control Holdings | Offshore Aquaculture | India | 2015 | 0 |
| Catalina Sea Ranch project moves ahead | Offshore Aquaculture | USA | 2014 | 1 |
| Making mussels | Offshore Aquaculture | USA | 2014 | 1 |
| Capital gets Maritime Plan 2030 for sustainable development | Offshore Aquaculture | United Arab Emirates | 2014 | 1 |
| Investors eye Gulf aquaculture boom | Offshore Aquaculture | Oman | 2014 | 1 |
| Oman, Saudi Arabia ride new wave of investment in aquaculture industry | Offshore Aquaculture | Oman | 2014 | 1 |
| Who Is Conservation For? | Offshore Aquaculture | USA | 2013 | -1 |
| Mussel beds planned near wind farm | Offshore Aquaculture | USA | 2013 | 1 |
| Seafood worker Hong Kong-bound | Offshore Aquaculture | China | 2013 | 1 |
| Oyster beds off Crowes Pasture hit again | Offshore Aquaculture | USA | 2013 | -1 |
| Judge says Marin oyster farm must obey Coastal Commission order | Offshore Aquaculture | USA | 2013 | -1 |
| Port's waters no good to explore | Offshore Aquaculture | Australia | 2013 | -1 |
| New lawsuit claims state wrongly put sanctions on Drakes Bay oyster farm | Offshore Aquaculture | USA | 2013 | 0 |
| Point Reyes oyster farm dealt another setback | Offshore Aquaculture | USA | 2013 | -1 |
| Markey eyes fishing, opposes NOAA aquaculture | Offshore Aquaculture | USA | 2013 | -1 |
| Catch 22 | Offshore Aquaculture | Australia | 2012 | -1 |
| MARITIME POLICY : ATLANTIC ARC AT HEART OF NEW MACRO-REGIONAL STRATEGY | Offshore Aquaculture | Portugal | 2011 | 0 |
| Ireland 'suffered' over fisheries policy | Offshore Aquaculture | Ireland | 2011 | -1 |
| Irish food drive serves up tasty results | Offshore Aquaculture | Ireland | 2011 | 1 |
| Bluefin tuna fingerlings sea trial survival rates hailed | Offshore Aquaculture | Australia | 2011 | 1 |
| Clean Seas buoyed by tuna trials | Offshore Aquaculture | Australia | 2011 | 1 |
| Tuna trial victory | Offshore Aquaculture | Australia | 2011 | 1 |
| Raising tuna in a tank closer to reality, researcher says | Offshore Aquaculture | USA | 2011 | 1 |
| No fish, no drill marine national park | Offshore Aquaculture | Australia | 2011 | -1 |
| Humongous fish farms are industrializing our oceans | Offshore Aquaculture | USA | 2011 | -1 |
| Bluefin breeding on track | Offshore Aquaculture | Australia | 2011 | 1 |
| Marsh likely to be gone by 2100 | Offshore Aquaculture | USA | 2010 | -1 |
| Fishing Policy Must Be Consistent | Offshore Aquaculture | USA | 2010 | -1 |
| NOAA launches aquaculture plan, economic incentives | Offshore Aquaculture | USA | 2010 | 1 |
| Aquaculture viable if planned responsibly; U.S. bill aims to set standards to ensure environment is respected | Offshore Aquaculture | USA | 2010 | 1 |
| Maine sites considered for testing wave energy; Resolute Marine's CEO envisions its converters using the same areas as floating wind turbines. | Offshore Aquaculture | USA | 2010 | 1 |
| Go slow, mussel-breeders urge speeding ferry boats | Offshore Aquaculture | Malaysia | 2009 | 0 |
| TIME FOR A GREEN POWER TRIP; Renewable energy technologies offer not only huge commercial opportunities but also some pretty novel science | Offshore Aquaculture | United Kingdom | 2009 | 1 |
| CEO plans added value for exports | Offshore Aquaculture | Australia | 2009 | 1 |
| Farming the ocean as we farm the land | Offshore Aquaculture | USA | 2009 | 1 |
| Fish farming gets OK for fed waters | Offshore Aquaculture | USA | 2009 | 1 |
| Reefs get $3.3 million stimulus: Staghorn, elkhorn nurseries to boost reef restoration | Offshore Aquaculture | USA | 2009 | 1 |
| Rove pick doesn't negate group's work | Offshore Aquaculture | USA | 2009 | 1 |
| Secrets of the deep blue sea | Offshore Aquaculture | Ireland | 2008 | 1 |
| FISH FARMS IN GULF TOO RISKY | Offshore Aquaculture | USA | 2008 | -1 |
| Exxon spill awards could be held up in court for several weeks | Offshore Aquaculture | USA | 2008 | -1 |
| Special panel studying nursing shortage in R.I. | Offshore Aquaculture | USA | 2008 | 0 |
| 100,000 sea breams to be released into sea | Offshore Aquaculture | Bahrain | 2008 | 1 |
| Environmental program open to public | Offshore Aquaculture | USA | 2008 | 0 |
| OUTDOORS NOTES | Offshore Aquaculture | USA | 2008 | 0 |
| Clean Seas husbandry a tuna aquaculture breakthrough - South Australia: Special Report | Offshore Aquaculture | Australia | 2008 | 1 |
| Salmon Farming May Doom Wild Populations, Study Says | Offshore Aquaculture | USA | 2007 | -1 |
| Open ocean farms could feed the world; Food: Official at aquaculture conference warns we need a replacement for dwindling fish stocks | Offshore Aquaculture | Canada | 2007 | 1 |
| Aquaculture industry looks at developments; Technology Conference explores ways of moving cages further offshore | Offshore Aquaculture | Canada | 2007 | 1 |
| Offshore aquaculture the wave of the future; Lobbying: N.B. minister, counterparts seek long-term strategy | Offshore Aquaculture | Canada | 2007 | 1 |
| Marine advisory council to discuss oil spill | Offshore Aquaculture | USA | 2007 | -1 |
| Maine island fall cleanups deserve praise; Adventure club meets Oct. 30 | Offshore Aquaculture | USA | 2007 | 1 |
| Threats of fish farming | Offshore Aquaculture | USA | 2007 | -1 |
| Deep-water aquaculture firm attracts vencap investment | Offshore Aquaculture | Canada | 2007 | 1 |
| Strong debut for Western Kingfish | Offshore Aquaculture | Australia | 2007 | 1 |
| LEGAL NOTICES | Offshore Aquaculture | USA | 2007 | 0 |
| U.S. is wary of ocean fish farms despite flood of foreign seafood; But experts say open-ocean aquaculture is an issue of when and how -- not if | Offshore Aquaculture | USA | 2007 | -1 |
| IMPORTS FUEL PUSH FOR U.S. OCEAN FISH FARMS | Offshore Aquaculture | USA | 2007 | 1 |
| Billion dollar baby | Offshore Aquaculture | New Zealand | 2007 | 1 |
| A changing province; By 2050, Scott Simpson and Gordon Hamilton report, projections indicate global warming will make the British Columbia we know a vastly different place. | Offshore Aquaculture | Canada | 2007 | -1 |
| Aquaculture advance hailed | Offshore Aquaculture | United Kingdom | 2007 | 0 |
| How many little fish does it take to grow a big fish?; Aquaculture's abundant promise must be buttressed by appropriate safeguards. | Offshore Aquaculture | USA | 2007 | -1 |
| Feds eye deep-sea fish farms; Similar operations already allowed in U.S. | Offshore Aquaculture | Canada | 2007 | 1 |
| American fish farms going deep | Offshore Aquaculture | Canada | 2007 | 1 |
| Ottawa interested in U.S. plan for deep-sea fish farms | Offshore Aquaculture | Canada | 2007 | 1 |
| Letters | Offshore Aquaculture | USA | 2007 | 0 |
| Fish farms in the ocean? Group pushes Congress to pass tough rules. | Offshore Aquaculture | USA | 2007 | -1 |
| Dire Forecast for the Oceans | Offshore Aquaculture | USA | 2006 | -1 |
| OUTDOORS NOTES | Offshore Aquaculture | USA | 2006 | 0 |
| N.C. still opposing offshore oil drilling | Offshore Aquaculture | USA | 2006 | -1 |
| DETERMINATION 'Half-illegal immigrant' an SA giant; 'Can do' the only way to succeed | Offshore Aquaculture | Australia | 2006 | -1 |
| New leases will add mussel to state's industry | Offshore Aquaculture | Australia | 2006 | 1 |
| KINGFISH Hope of expanding European market; Fishing giant targets Russia in export trial | Offshore Aquaculture | Australia | 2006 | 1 |
| Green to the Gills | Offshore Aquaculture | USA | 2006 | 1 |
| Stevens casts net in offshore farm-fishing permit debate | Offshore Aquaculture | USA | 2006 | 0 |
| Massachusetts governor gets veto power in wind farm bill | Offshore Aquaculture | USA | 2006 | 0 |
| Delegates set policy direction for soybean group Sioux Falls Ã Directors of the South Dakota Soybean Association (SDSA) joined other producers from around ... | Offshore Aquaculture | USA | 2006 | 0 |
| FISH OF THE FUTURE?; Hawaiian fish farm begins harvest of Kona Kampachi | Offshore Aquaculture | USA | 2006 | 1 |
| Fish of the future?; HAWAIIAN COMPANY RAISES STANDARDS ON DEEP-SEA FARM | Offshore Aquaculture | USA | 2006 | 1 |
| BIG SHOT | Offshore Aquaculture | Australia | 2006 | 0 |
| Ocean aquaculture | Offshore Aquaculture | USA | 2005 | 0 |
| I hear and accept others' viewpoints | Offshore Aquaculture | Canada | 2005 | 0 |
| Aquaculture works | Offshore Aquaculture | USA | 2005 | 1 |
| OFF SHORE AND OFF TARGET | Offshore Aquaculture | USA | 2005 | -1 |
| Measure to encourage fish, shellfish farms in federal waters | Offshore Aquaculture | USA | 2005 | 1 |
| Radical Bush plan boosts B.C. farmed fish industry: Cod, halibut, red snapper to be raised | Offshore Aquaculture | Canada | 2005 | 1 |
| White House Seeks to Boost Fish Farms by Expanding Into Open Waters | Offshore Aquaculture | USA | 2005 | 1 |
| Plan Would Expand Ocean Fish Farming | Offshore Aquaculture | USA | 2005 | 1 |
| Alaskans attack fish farms | Offshore Aquaculture | Canada | 2005 | -1 |
| Catch a big one in Albany | Offshore Aquaculture | Australia | 2005 | 1 |
| Marine Life Complicates Removal of Old Oil Rigs | Offshore Aquaculture | USA | 2005 | 0 |
| Hopes raised for deep-sea fish farms; White House proposes removing some 'obstacles' | Offshore Aquaculture | Ireland | 2005 | 1 |
| Offshore energy a bridge to future | Offshore Aquaculture | Canada | 2005 | 1 |
| Fish cages vital for a marine revolution, says BIM | Offshore Aquaculture | Ireland | 2005 | 1 |
| Ideas hauled in out of the blue | Offshore Aquaculture | Australia | 2005 | 0 |
| Canadians with that long-distance feeling; Campobello Islanders have long considered themselves more like Americans, but 9/11 and now the rise of Stephen Harper seem to be changing all that, SHAWNA RICHER reports | Offshore Aquaculture | Canada | 2004 | 0 |
| Bird lovers count beaks on holiday | Offshore Aquaculture | USA | 2004 | 0 |
| Plans for offshore fish farm rejected | Offshore Aquaculture | USA | 2004 | -1 |
| Panel recommends denial of gulf aquaculture permit | Offshore Aquaculture | USA | 2004 | -1 |
| Casting doubt on fish farming | Offshore Aquaculture | USA | 2004 | -1 |
| Public shouldn't have to pay for the power grid | Offshore Aquaculture | USA | 2004 | -1 |
| RAISING MORE FISH IN CAGES SEEMS A CERTAINTY | Offshore Aquaculture | USA | 2004 | 1 |
| New boat era for fish farms | Offshore Aquaculture | Australia | 2004 | 1 |
| Turkish spinoffs possible | Offshore Aquaculture | USA | 2004 | 0 |
| Fish farm seen as danger to seals | Offshore Aquaculture | Australia | 2004 | -1 |
| Maine Salmon Face Upstream Battle as Species; Returns Dwindle as Aquaculture Booms | Offshore Aquaculture | USA | 2004 | -1 |
| Fishing for growth | Offshore Aquaculture | Australia | 2004 | 1 |
| Aquaculture way to boost fish stocks | Offshore Aquaculture | Malaysia | 2004 | 1 |
| Storm Swirls Over Aboriginal Salmon In Maine's Rivers | Offshore Aquaculture | USA | 2004 | -1 |
| Scientific dreams of a sustainable future; Nature Report | Offshore Aquaculture | Australia | 1994 | 1 |
