# Supplementary material for: Public Perceptions of Aquaculture: Evaluating Spatiotemporal Patterns of Sentiment around the World
Source: PLoS One. 2017 Jan 3;12(1):e0169281. doi: 10.1371/journal.pone.0169281 (PMC5207524; doi:10.1371/journal.pone.0169281)
Supplement: S2 Table — (DOCX) [file pone.0169281.s002.docx]

**S2 Table List of added negative and positive terms specific to aquaculture.**

| **Aquaculture Term** | **Sentiment** |
| --- | --- |
| antibiotic | Negative |
| chemical | Negative |
| competition | Negative |
| cost | Negative |
| disease | Negative |
| effluent | Negative |
| endangered | Negative |
| entangle | Negative |
| escapes | Negative |
| expensive | Negative |
| fishing | Negative |
| food | Positive |
| impact | Negative |
| invasive | Negative |
| liability | Negative |
| lice | Negative |
| mortality | Negative |
| nonindigenious | Negative |
| oil | Negative |
| oppose | Negative |
| overfish | Negative |
| parasite | Negative |
| pesticide | Negative |
| pollution | Negative |
| prohibited | Negative |
| seafood | Positive |
| serious | Negative |
| spill | Negative |
| sustainable | Positive |
| undue | Negative |
| whaling | Negative |
