# Supplementary material for: Public Perceptions of Aquaculture: Evaluating Spatiotemporal Patterns of Sentiment around the World
Source: PLoS One. 2017 Jan 3;12(1):e0169281. doi: 10.1371/journal.pone.0169281 (PMC5207524; doi:10.1371/journal.pone.0169281)
Supplement: S3 Table — Included in the table are the words extracted from all government documents, the total frequency of use, and the ‘opinion lexicon’ assigned sentiment associations (positive, negative, and neutral). Documents include the 2011 USA Marine Aquaculture Policy, 2008 USA Offshore Aquaculture Gulf of Mexico (GOM) regulatory plan, 2011 NZ Policy Legislation Bill (No. 3) for marine farming, and the 2009 NZ expansion plan for salmon farming in Marlborough marine waters. (DOCX) [file pone.0169281.s003.docx]

**S3 Table Complete list of words from government public comments.** Included in the table are the words extracted from all government documents, the total frequency of use, and the ‘opinion lexicon’ assigned sentiment associations (positive, negative, and neutral). Documents include the 2011 USA Marine Aquaculture Policy, 2008 USA Offshore Aquaculture Gulf of Mexico (GOM) regulatory plan, 2011 NZ Policy Legislation Bill (No. 3) for marine farming, and the 2009 NZ expansion plan for salmon farming in Marlborough marine waters.

| **Source** | **Word** | **Frequency** | **Sentiment** |
| --- | --- | --- | --- |
| NZ Mngt | cost | 31 | Negative |
| NZ Mngt | issue | 27 | Negative |
| NZ Mngt | wildlife | 27 | Negative |
| NZ Mngt | concern | 20 | Negative |
| NZ Mngt | competition | 18 | Negative |
| NZ Mngt | oppose | 18 | Negative |
| NZ Mngt | exclude | 17 | Negative |
| NZ Mngt | risk | 13 | Negative |
| NZ Mngt | fishing | 11 | Negative |
| NZ Mngt | limited | 10 | Negative |
| NZ Mngt | collapse | 8 | Negative |
| NZ Mngt | impact | 8 | Negative |
| NZ Mngt | decline | 6 | Negative |
| NZ Mngt | deny | 6 | Negative |
| NZ Mngt | disadvantage | 5 | Negative |
| NZ Mngt | lapses | 5 | Negative |
| NZ Mngt | pollution | 5 | Negative |
| NZ Mngt | unlikely | 5 | Negative |
| NZ Mngt | damage | 4 | Negative |
| NZ Mngt | disastrous | 4 | Negative |
| NZ Mngt | disenfranchise | 4 | Negative |
| NZ Mngt | fails | 4 | Negative |
| NZ Mngt | hard | 4 | Negative |
| NZ Mngt | ignore | 4 | Negative |
| NZ Mngt | inappropriate | 4 | Negative |
| NZ Mngt | prohibit | 4 | Negative |
| NZ Mngt | unpopular | 4 | Negative |
| NZ Mngt | adverse | 3 | Negative |
| NZ Mngt | deficiencies | 3 | Negative |
| NZ Mngt | exploit | 3 | Negative |
| NZ Mngt | lack | 3 | Negative |
| NZ Mngt | limit | 3 | Negative |
| NZ Mngt | naive | 3 | Negative |
| NZ Mngt | object | 3 | Negative |
| NZ Mngt | poor | 3 | Negative |
| NZ Mngt | unable | 3 | Negative |
| NZ Mngt | anger | 2 | Negative |
| NZ Mngt | appalled | 2 | Negative |
| NZ Mngt | bad | 2 | Negative |
| NZ Mngt | blind | 2 | Negative |
| NZ Mngt | conflict | 2 | Negative |
| NZ Mngt | critical | 2 | Negative |
| NZ Mngt | detract | 2 | Negative |
| NZ Mngt | detriment | 2 | Negative |
| NZ Mngt | dirty | 2 | Negative |
| NZ Mngt | discontent | 2 | Negative |
| NZ Mngt | dismayed | 2 | Negative |
| NZ Mngt | drag | 2 | Negative |
| NZ Mngt | drastically | 2 | Negative |
| NZ Mngt | dump | 2 | Negative |
| NZ Mngt | expensive | 2 | Negative |
| NZ Mngt | faeces | 2 | Negative |
| NZ Mngt | fragmented | 2 | Negative |
| NZ Mngt | frustration | 2 | Negative |
| NZ Mngt | hell | 2 | Negative |
| NZ Mngt | inability | 2 | Negative |
| NZ Mngt | injustice | 2 | Negative |
| NZ Mngt | mess | 2 | Negative |
| NZ Mngt | misappropriation | 2 | Negative |
| NZ Mngt | mistakes | 2 | Negative |
| NZ Mngt | odd | 2 | Negative |
| NZ Mngt | oversight | 2 | Negative |
| NZ Mngt | pleas | 2 | Negative |
| NZ Mngt | polluters | 2 | Negative |
| NZ Mngt | refuse | 2 | Negative |
| NZ Mngt | reject | 2 | Negative |
| NZ Mngt | rubbish | 2 | Negative |
| NZ Mngt | ruined | 2 | Negative |
| NZ Mngt | serious | 2 | Negative |
| NZ Mngt | struggle | 2 | Negative |
| NZ Mngt | suspect | 2 | Negative |
| NZ Mngt | undue | 2 | Negative |
| NZ Mngt | unforeseen | 2 | Negative |
| NZ Mngt | unfortunately | 2 | Negative |
| NZ Mngt | unknown | 2 | Negative |
| NZ Mngt | unscrupulous | 2 | Negative |
| NZ Mngt | waste | 2 | Negative |
| NZ Mngt | wrong | 2 | Negative |
| NZ Mngt | lose | 2 | Negative |
| NZ Mngt | rumble | 2 | Negative |
| NZ Mngt | whaling | 2 | Negative |
| NZ Mngt | plan | 506 | Neutral |
| NZ Mngt | change | 457 | Neutral |
| NZ Mngt | ama | 240 | Neutral |
| NZ Mngt | council | 233 | Neutral |
| NZ Mngt | area | 189 | Neutral |
| NZ Mngt | allocate | 171 | Neutral |
| NZ Mngt | sounds | 142 | Neutral |
| NZ Mngt | private | 130 | Neutral |
| NZ Mngt | marlborough | 113 | Neutral |
| NZ Mngt | management | 111 | Neutral |
| NZ Mngt | method | 110 | Neutral |
| NZ Mngt | process | 107 | Neutral |
| NZ Mngt | coastal | 105 | Neutral |
| NZ Mngt | consider | 101 | Neutral |
| NZ Mngt | public | 98 | Neutral |
| NZ Mngt | resource | 98 | Neutral |
| NZ Mngt | authorisations | 89 | Neutral |
| NZ Mngt | applicant | 87 | Neutral |
| NZ Mngt | industry | 77 | Neutral |
| NZ Mngt | alternative | 70 | Neutral |
| NZ Mngt | environment | 66 | Neutral |
| NZ Mngt | space | 63 | Neutral |
| NZ Mngt | develop | 61 | Neutral |
| NZ Mngt | community | 57 | Neutral |
| NZ Mngt | act | 53 | Neutral |
| NZ Mngt | apply | 51 | Neutral |
| NZ Mngt | submission | 50 | Neutral |
| NZ Mngt | part | 49 | Neutral |
| NZ Mngt | establish | 48 | Neutral |
| NZ Mngt | use | 47 | Neutral |
| NZ Mngt | created | 44 | Neutral |
| NZ Mngt | make | 41 | Neutral |
| NZ Mngt | include | 40 | Neutral |
| NZ Mngt | rules | 40 | Neutral |
| NZ Mngt | effect | 38 | Neutral |
| NZ Mngt | decide | 37 | Neutral |
| NZ Mngt | salmon | 37 | Neutral |
| NZ Mngt | give | 36 | Neutral |
| NZ Mngt | policy | 36 | Neutral |
| NZ Mngt | plan | 35 | Neutral |
| NZ Mngt | relate | 35 | Neutral |
| NZ Mngt | zones | 35 | Neutral |
| NZ Mngt | allow | 34 | Neutral |
| NZ Mngt | pelorus | 33 | Neutral |
| NZ Mngt | requests | 33 | Neutral |
| NZ Mngt | require | 33 | Neutral |
| NZ Mngt | specific | 33 | Neutral |
| NZ Mngt | tender | 33 | Neutral |
| NZ Mngt | commercial | 32 | Neutral |
| NZ Mngt | company | 32 | Neutral |
| NZ Mngt | settlement | 31 | Neutral |
| NZ Mngt | zone | 31 | Neutral |
| NZ Mngt | bay | 30 | Neutral |
| NZ Mngt | need | 30 | Neutral |
| NZ Mngt | sound | 30 | Neutral |
| NZ Mngt | seek | 29 | Neutral |
| NZ Mngt | current | 28 | Neutral |
| NZ Mngt | standard | 28 | Neutral |
| NZ Mngt | consultation | 27 | Neutral |
| NZ Mngt | deleted | 27 | Neutral |
| NZ Mngt | exist | 27 | Neutral |
| NZ Mngt | follow | 27 | Neutral |
| NZ Mngt | considered | 26 | Neutral |
| NZ Mngt | iwi | 26 | Neutral |
| NZ Mngt | request | 26 | Neutral |
| NZ Mngt | take | 26 | Neutral |
| NZ Mngt | government | 25 | Neutral |
| NZ Mngt | king | 25 | Neutral |
| NZ Mngt | provide | 25 | Neutral |
| NZ Mngt | zealand | 25 | Neutral |
| NZ Mngt | implement | 24 | Neutral |
| NZ Mngt | likely | 24 | Neutral |
| NZ Mngt | local | 24 | Neutral |
| NZ Mngt | volume | 24 | Neutral |
| NZ Mngt | approach | 23 | Neutral |
| NZ Mngt | ensure | 23 | Neutral |
| NZ Mngt | place | 23 | Neutral |
| NZ Mngt | rma | 23 | Neutral |
| NZ Mngt | authoris | 22 | Neutral |
| NZ Mngt | district | 22 | Neutral |
| NZ Mngt | long | 22 | Neutral |
| NZ Mngt | reasons | 22 | Neutral |
| NZ Mngt | certainty | 21 | Neutral |
| NZ Mngt | maintain | 21 | Neutral |
| NZ Mngt | people | 21 | Neutral |
| NZ Mngt | permit | 21 | Neutral |
| NZ Mngt | provisions | 21 | Neutral |
| NZ Mngt | term | 21 | Neutral |
| NZ Mngt | consent | 20 | Neutral |
| NZ Mngt | default | 20 | Neutral |
| NZ Mngt | involve | 20 | Neutral |
| NZ Mngt | maori | 20 | Neutral |
| NZ Mngt | natural | 20 | Neutral |
| NZ Mngt | ohu | 20 | Neutral |
| NZ Mngt | outcome | 20 | Neutral |
| NZ Mngt | want | 20 | Neutral |
| NZ Mngt | become | 19 | Neutral |
| NZ Mngt | economic | 19 | Neutral |
| NZ Mngt | friends | 19 | Neutral |
| NZ Mngt | general | 19 | Neutral |
| NZ Mngt | particular | 19 | Neutral |
| NZ Mngt | plans | 19 | Neutral |
| NZ Mngt | water | 19 | Neutral |
| NZ Mngt | activity | 18 | Neutral |
| NZ Mngt | stakeholder | 18 | Neutral |
| NZ Mngt | without | 18 | Neutral |
| NZ Mngt | believe | 17 | Neutral |
| NZ Mngt | claims | 17 | Neutral |
| NZ Mngt | future | 17 | Neutral |
| NZ Mngt | intended | 17 | Neutral |
| NZ Mngt | must | 17 | Neutral |
| NZ Mngt | processes | 17 | Neutral |
| NZ Mngt | proposal | 17 | Neutral |
| NZ Mngt | address | 16 | Neutral |
| NZ Mngt | basis | 16 | Neutral |
| NZ Mngt | create | 16 | Neutral |
| NZ Mngt | identify | 16 | Neutral |
| NZ Mngt | individual | 16 | Neutral |
| NZ Mngt | organisation | 16 | Neutral |
| NZ Mngt | tourism | 16 | Neutral |
| NZ Mngt | treaty | 16 | Neutral |
| NZ Mngt | authorisation | 15 | Neutral |
| NZ Mngt | consideration | 15 | Neutral |
| NZ Mngt | deemed | 15 | Neutral |
| NZ Mngt | initiate | 15 | Neutral |
| NZ Mngt | made | 15 | Neutral |
| NZ Mngt | needs | 15 | Neutral |
| NZ Mngt | objective | 15 | Neutral |
| NZ Mngt | potential | 15 | Neutral |
| NZ Mngt | provides | 15 | Neutral |
| NZ Mngt | receive | 15 | Neutral |
| NZ Mngt | trustee | 15 | Neutral |
| NZ Mngt | add | 14 | Neutral |
| NZ Mngt | business | 14 | Neutral |
| NZ Mngt | businesses | 14 | Neutral |
| NZ Mngt | framework | 14 | Neutral |
| NZ Mngt | person | 14 | Neutral |
| NZ Mngt | policies | 14 | Neutral |
| NZ Mngt | possible | 14 | Neutral |
| NZ Mngt | put | 14 | Neutral |
| NZ Mngt | set | 14 | Neutral |
| NZ Mngt | amend | 13 | Neutral |
| NZ Mngt | chapter | 13 | Neutral |
| NZ Mngt | however | 13 | Neutral |
| NZ Mngt | intent | 13 | Neutral |
| NZ Mngt | legislation | 13 | Neutral |
| NZ Mngt | project | 13 | Neutral |
| NZ Mngt | recognise | 13 | Neutral |
| NZ Mngt | aquaculture | 12 | Neutral |
| NZ Mngt | ahead | 12 | Neutral |
| NZ Mngt | another | 12 | Neutral |
| NZ Mngt | control | 12 | Neutral |
| NZ Mngt | councilinitiated | 12 | Neutral |
| NZ Mngt | detailed | 12 | Neutral |
| NZ Mngt | done | 12 | Neutral |
| NZ Mngt | east | 12 | Neutral |
| NZ Mngt | economy | 12 | Neutral |
| NZ Mngt | enable | 12 | Neutral |
| NZ Mngt | fisheries | 12 | Neutral |
| NZ Mngt | growth | 12 | Neutral |
| NZ Mngt | introduce | 12 | Neutral |
| NZ Mngt | later | 12 | Neutral |
| NZ Mngt | manage | 12 | Neutral |
| NZ Mngt | matter | 12 | Neutral |
| NZ Mngt | principle | 12 | Neutral |
| NZ Mngt | recreational | 12 | Neutral |
| NZ Mngt | sought | 12 | Neutral |
| NZ Mngt | subsequent | 12 | Neutral |
| NZ Mngt | therefore | 12 | Neutral |
| NZ Mngt | agree | 11 | Neutral |
| NZ Mngt | island | 11 | Neutral |
| NZ Mngt | look | 11 | Neutral |
| NZ Mngt | multinational | 11 | Neutral |
| NZ Mngt | remain | 11 | Neutral |
| NZ Mngt | removal | 11 | Neutral |
| NZ Mngt | short | 11 | Neutral |
| NZ Mngt | states | 11 | Neutral |
| NZ Mngt | tend | 11 | Neutral |
| NZ Mngt | unfair | 11 | Neutral |
| NZ Mngt | whether | 11 | Neutral |
| NZ Mngt | wider | 11 | Neutral |
| NZ Mngt | wish | 11 | Neutral |
| NZ Mngt | accept | 10 | Neutral |
| NZ Mngt | achieve | 10 | Neutral |
| NZ Mngt | affect | 10 | Neutral |
| NZ Mngt | currently | 10 | Neutral |
| NZ Mngt | dolphin | 10 | Neutral |
| NZ Mngt | expand | 10 | Neutral |
| NZ Mngt | farmers | 10 | Neutral |
| NZ Mngt | happen | 10 | Neutral |
| NZ Mngt | note | 10 | Neutral |
| NZ Mngt | port | 10 | Neutral |
| NZ Mngt | prohibited | 10 | Neutral |
| NZ Mngt | requested | 10 | Neutral |
| NZ Mngt | rid | 10 | Neutral |
| NZ Mngt | undertaken | 10 | Neutral |
| NZ Mngt | wairauawatere | 10 | Neutral |
| NZ Mngt | able | 9 | Neutral |
| NZ Mngt | acknowledged | 9 | Neutral |
| NZ Mngt | case | 9 | Neutral |
| NZ Mngt | circumstances | 9 | Neutral |
| NZ Mngt | consider | 9 | Neutral |
| NZ Mngt | doc | 9 | Neutral |
| NZ Mngt | end | 9 | Neutral |
| NZ Mngt | every | 9 | Neutral |
| NZ Mngt | fact | 9 | Neutral |
| NZ Mngt | fund | 9 | Neutral |
| NZ Mngt | granted | 9 | Neutral |
| NZ Mngt | know | 9 | Neutral |
| NZ Mngt | large | 9 | Neutral |
| NZ Mngt | mechanism | 9 | Neutral |
| NZ Mngt | money | 9 | Neutral |
| NZ Mngt | mussel | 9 | Neutral |
| NZ Mngt | native | 9 | Neutral |
| NZ Mngt | nature | 9 | Neutral |
| NZ Mngt | offer | 9 | Neutral |
| NZ Mngt | opportunity | 9 | Neutral |
| NZ Mngt | order | 9 | Neutral |
| NZ Mngt | prior | 9 | Neutral |
| NZ Mngt | procurement | 9 | Neutral |
| NZ Mngt | region | 9 | Neutral |
| NZ Mngt | regional | 9 | Neutral |
| NZ Mngt | review | 9 | Neutral |
| NZ Mngt | rush | 9 | Neutral |
| NZ Mngt | sanctuaries | 9 | Neutral |
| NZ Mngt | shall | 9 | Neutral |
| NZ Mngt | smaller | 9 | Neutral |
| NZ Mngt | strategic | 9 | Neutral |
| NZ Mngt | terms | 9 | Neutral |
| NZ Mngt | unless | 9 | Neutral |
| NZ Mngt | upon | 9 | Neutral |
| NZ Mngt | user | 9 | Neutral |
| NZ Mngt | already | 8 | Neutral |
| NZ Mngt | blenheim | 8 | Neutral |
| NZ Mngt | conservation | 8 | Neutral |
| NZ Mngt | considerable | 8 | Neutral |
| NZ Mngt | court | 8 | Neutral |
| NZ Mngt | delete | 8 | Neutral |
| NZ Mngt | demand | 8 | Neutral |
| NZ Mngt | especially | 8 | Neutral |
| NZ Mngt | full | 8 | Neutral |
| NZ Mngt | ippc | 8 | Neutral |
| NZ Mngt | law | 8 | Neutral |
| NZ Mngt | level | 8 | Neutral |
| NZ Mngt | means | 8 | Neutral |
| NZ Mngt | minor | 8 | Neutral |
| NZ Mngt | monoculture | 8 | Neutral |
| NZ Mngt | offered | 8 | Neutral |
| NZ Mngt | ongo | 8 | Neutral |
| NZ Mngt | operative | 8 | Neutral |
| NZ Mngt | outer | 8 | Neutral |
| NZ Mngt | owner | 8 | Neutral |
| NZ Mngt | paragraph | 8 | Neutral |
| NZ Mngt | provided | 8 | Neutral |
| NZ Mngt | provid | 8 | Neutral |
| NZ Mngt | rate | 8 | Neutral |
| NZ Mngt | rights | 8 | Neutral |
| NZ Mngt | schedule | 8 | Neutral |
| NZ Mngt | see | 8 | Neutral |
| NZ Mngt | shown | 8 | Neutral |
| NZ Mngt | stage | 8 | Neutral |
| NZ Mngt | statement | 8 | Neutral |
| NZ Mngt | surety | 8 | Neutral |
| NZ Mngt | text | 8 | Neutral |
| NZ Mngt | zon | 8 | Neutral |
| NZ Mngt | zon | 8 | Neutral |
| NZ Mngt | april | 7 | Neutral |
| NZ Mngt | assets | 7 | Neutral |
| NZ Mngt | balance | 7 | Neutral |
| NZ Mngt | based | 7 | Neutral |
| NZ Mngt | biggest | 7 | Neutral |
| NZ Mngt | capital | 7 | Neutral |
| NZ Mngt | come | 7 | Neutral |
| NZ Mngt | culture | 7 | Neutral |
| NZ Mngt | dont | 7 | Neutral |
| NZ Mngt | exercise | 7 | Neutral |
| NZ Mngt | expect | 7 | Neutral |
| NZ Mngt | follow | 7 | Neutral |
| NZ Mngt | form | 7 | Neutral |
| NZ Mngt | group | 7 | Neutral |
| NZ Mngt | heard | 7 | Neutral |
| NZ Mngt | increase | 7 | Neutral |
| NZ Mngt | invest | 7 | Neutral |
| NZ Mngt | msrmp | 7 | Neutral |
| NZ Mngt | ofthe | 7 | Neutral |
| NZ Mngt | parts | 7 | Neutral |
| NZ Mngt | payers | 7 | Neutral |
| NZ Mngt | principles | 7 | Neutral |
| NZ Mngt | received | 7 | Neutral |
| NZ Mngt | residents | 7 | Neutral |
| NZ Mngt | seabed | 7 | Neutral |
| NZ Mngt | strategy | 7 | Neutral |
| NZ Mngt | system | 7 | Neutral |
| NZ Mngt | value | 7 | Neutral |
| NZ Mngt | appear | 6 | Neutral |
| NZ Mngt | assess | 6 | Neutral |
| NZ Mngt | big | 6 | Neutral |
| NZ Mngt | blue | 6 | Neutral |
| NZ Mngt | borne | 6 | Neutral |
| NZ Mngt | boroughs | 6 | Neutral |
| NZ Mngt | character | 6 | Neutral |
| NZ Mngt | charlotte | 6 | Neutral |
| NZ Mngt | cod | 6 | Neutral |
| NZ Mngt | complete | 6 | Neutral |
| NZ Mngt | consequence | 6 | Neutral |
| NZ Mngt | contain | 6 | Neutral |
| NZ Mngt | contrary | 6 | Neutral |
| NZ Mngt | covered | 6 | Neutral |
| NZ Mngt | criteria | 6 | Neutral |
| NZ Mngt | different | 6 | Neutral |
| NZ Mngt | ecological | 6 | Neutral |
| NZ Mngt | effort | 6 | Neutral |
| NZ Mngt | evaluat | 6 | Neutral |
| NZ Mngt | guardians | 6 | Neutral |
| NZ Mngt | inserted | 6 | Neutral |
| NZ Mngt | instead | 6 | Neutral |
| NZ Mngt | invited | 6 | Neutral |
| NZ Mngt | learn | 6 | Neutral |
| NZ Mngt | marl | 6 | Neutral |
| NZ Mngt | minister | 6 | Neutral |
| NZ Mngt | necessary | 6 | Neutral |
| NZ Mngt | notification | 6 | Neutral |
| NZ Mngt | operation | 6 | Neutral |
| NZ Mngt | others | 6 | Neutral |
| NZ Mngt | ownership | 6 | Neutral |
| NZ Mngt | permits | 6 | Neutral |
| NZ Mngt | players | 6 | Neutral |
| NZ Mngt | potentially | 6 | Neutral |
| NZ Mngt | preference | 6 | Neutral |
| NZ Mngt | procedure | 6 | Neutral |
| NZ Mngt | property | 6 | Neutral |
| NZ Mngt | queen | 6 | Neutral |
| NZ Mngt | reason | 6 | Neutral |
| NZ Mngt | regard | 6 | Neutral |
| NZ Mngt | report | 6 | Neutral |
| NZ Mngt | research | 6 | Neutral |
| NZ Mngt | role | 6 | Neutral |
| NZ Mngt | second | 6 | Neutral |
| NZ Mngt | sections | 6 | Neutral |
| NZ Mngt | sighted | 6 | Neutral |
| NZ Mngt | spac | 6 | Neutral |
| NZ Mngt | state | 6 | Neutral |
| NZ Mngt | sub | 6 | Neutral |
| NZ Mngt | think | 6 | Neutral |
| NZ Mngt | tourists | 6 | Neutral |
| NZ Mngt | vendor | 6 | Neutral |
| NZ Mngt | word | 6 | Neutral |
| NZ Mngt | access | 5 | Neutral |
| NZ Mngt | argument | 5 | Neutral |
| NZ Mngt | aris | 5 | Neutral |
| NZ Mngt | assessment | 5 | Neutral |
| NZ Mngt | assist | 5 | Neutral |
| NZ Mngt | attached | 5 | Neutral |
| NZ Mngt | automatically | 5 | Neutral |
| NZ Mngt | biodiversity | 5 | Neutral |
| NZ Mngt | box | 5 | Neutral |
| NZ Mngt | busi | 5 | Neutral |
| NZ Mngt | cart | 5 | Neutral |
| NZ Mngt | certainly | 5 | Neutral |
| NZ Mngt | consulted | 5 | Neutral |
| NZ Mngt | context | 5 | Neutral |
| NZ Mngt | cumulative | 5 | Neutral |
| NZ Mngt | deal | 5 | Neutral |
| NZ Mngt | dealt | 5 | Neutral |
| NZ Mngt | determine | 5 | Neutral |
| NZ Mngt | direct | 5 | Neutral |
| NZ Mngt | ecosystem | 5 | Neutral |
| NZ Mngt | february | 5 | Neutral |
| NZ Mngt | go | 5 | Neutral |
| NZ Mngt | habitat | 5 | Neutral |
| NZ Mngt | help | 5 | Neutral |
| NZ Mngt | historic | 5 | Neutral |
| NZ Mngt | history | 5 | Neutral |
| NZ Mngt | hoc | 5 | Neutral |
| NZ Mngt | holistic | 5 | Neutral |
| NZ Mngt | initial | 5 | Neutral |
| NZ Mngt | initiatives | 5 | Neutral |
| NZ Mngt | land | 5 | Neutral |
| NZ Mngt | lawyers | 5 | Neutral |
| NZ Mngt | maintenance | 5 | Neutral |
| NZ Mngt | manag | 5 | Neutral |
| NZ Mngt | meet | 5 | Neutral |
| NZ Mngt | members | 5 | Neutral |
| NZ Mngt | never | 5 | Neutral |
| NZ Mngt | numerous | 5 | Neutral |
| NZ Mngt | owned | 5 | Neutral |
| NZ Mngt | parties | 5 | Neutral |
| NZ Mngt | please | 5 | Neutral |
| NZ Mngt | position | 5 | Neutral |
| NZ Mngt | precautionary | 5 | Neutral |
| NZ Mngt | present | 5 | Neutral |
| NZ Mngt | preserve | 5 | Neutral |
| NZ Mngt | previous | 5 | Neutral |
| NZ Mngt | priority | 5 | Neutral |
| NZ Mngt | quota | 5 | Neutral |
| NZ Mngt | regulatory | 5 | Neutral |
| NZ Mngt | remember | 5 | Neutral |
| NZ Mngt | sea | 5 | Neutral |
| NZ Mngt | since | 5 | Neutral |
| NZ Mngt | site | 5 | Neutral |
| NZ Mngt | statutory | 5 | Neutral |
| NZ Mngt | subheading | 5 | Neutral |
| NZ Mngt | told | 5 | Neutral |
| NZ Mngt | totaranui | 5 | Neutral |
| NZ Mngt | undertake | 5 | Neutral |
| NZ Mngt | values | 5 | Neutral |
| NZ Mngt | vital | 5 | Neutral |
| NZ Mngt | years | 5 | Neutral |
| NZ Mngt | yet | 5 | Neutral |
| NZ Mngt | accommodate | 4 | Neutral |
| NZ Mngt | accordance | 4 | Neutral |
| NZ Mngt | account | 4 | Neutral |
| NZ Mngt | along | 4 | Neutral |
| NZ Mngt | amount | 4 | Neutral |
| NZ Mngt | avoid | 4 | Neutral |
| NZ Mngt | bag | 4 | Neutral |
| NZ Mngt | begin | 4 | Neutral |
| NZ Mngt | behalf | 4 | Neutral |
| NZ Mngt | bill | 4 | Neutral |
| NZ Mngt | bird | 4 | Neutral |
| NZ Mngt | cant | 4 | Neutral |
| NZ Mngt | channel | 4 | Neutral |
| NZ Mngt | cheque | 4 | Neutral |
| NZ Mngt | choose | 4 | Neutral |
| NZ Mngt | closed | 4 | Neutral |
| NZ Mngt | collaborated | 4 | Neutral |
| NZ Mngt | committee | 4 | Neutral |
| NZ Mngt | common | 4 | Neutral |
| NZ Mngt | compliance | 4 | Neutral |
| NZ Mngt | conducive | 4 | Neutral |
| NZ Mngt | considerations | 4 | Neutral |
| NZ Mngt | continued | 4 | Neutral |
| NZ Mngt | crucial | 4 | Neutral |
| NZ Mngt | dataplan | 4 | Neutral |
| NZ Mngt | delet | 4 | Neutral |
| NZ Mngt | distribution | 4 | Neutral |
| NZ Mngt | dollar | 4 | Neutral |
| NZ Mngt | due | 4 | Neutral |
| NZ Mngt | durville | 4 | Neutral |
| NZ Mngt | earliest | 4 | Neutral |
| NZ Mngt | eco | 4 | Neutral |
| NZ Mngt | economi | 4 | Neutral |
| NZ Mngt | employ | 4 | Neutral |
| NZ Mngt | ensure | 4 | Neutral |
| NZ Mngt | entire | 4 | Neutral |
| NZ Mngt | ever | 4 | Neutral |
| NZ Mngt | exactly | 4 | Neutral |
| NZ Mngt | example | 4 | Neutral |
| NZ Mngt | field | 4 | Neutral |
| NZ Mngt | financial | 4 | Neutral |
| NZ Mngt | fisheri | 4 | Neutral |
| NZ Mngt | forward | 4 | Neutral |
| NZ Mngt | fund | 4 | Neutral |
| NZ Mngt | gas | 4 | Neutral |
| NZ Mngt | get | 4 | Neutral |
| NZ Mngt | goal | 4 | Neutral |
| NZ Mngt | gore | 4 | Neutral |
| NZ Mngt | hearing | 4 | Neutral |
| NZ Mngt | horse | 4 | Neutral |
| NZ Mngt | huge | 4 | Neutral |
| NZ Mngt | indigenous | 4 | Neutral |
| NZ Mngt | internationally | 4 | Neutral |
| NZ Mngt | left | 4 | Neutral |
| NZ Mngt | letter | 4 | Neutral |
| NZ Mngt | line | 4 | Neutral |
| NZ Mngt | logg | 4 | Neutral |
| NZ Mngt | manner | 4 | Neutral |
| NZ Mngt | maps | 4 | Neutral |
| NZ Mngt | mark | 4 | Neutral |
| NZ Mngt | might | 4 | Neutral |
| NZ Mngt | ministry | 4 | Neutral |
| NZ Mngt | monitor | 4 | Neutral |
| NZ Mngt | move | 4 | Neutral |
| NZ Mngt | nationally | 4 | Neutral |
| NZ Mngt | nzks | 4 | Neutral |
| NZ Mngt | obtained | 4 | Neutral |
| NZ Mngt | operators | 4 | Neutral |
| NZ Mngt | overall | 4 | Neutral |
| NZ Mngt | pag | 4 | Neutral |
| NZ Mngt | para | 4 | Neutral |
| NZ Mngt | park | 4 | Neutral |
| NZ Mngt | party | 4 | Neutral |
| NZ Mngt | pastoral | 4 | Neutral |
| NZ Mngt | peoples | 4 | Neutral |
| NZ Mngt | pick | 4 | Neutral |
| NZ Mngt | play | 4 | Neutral |
| NZ Mngt | point | 4 | Neutral |
| NZ Mngt | preparation | 4 | Neutral |
| NZ Mngt | pressure | 4 | Neutral |
| NZ Mngt | priorities | 4 | Neutral |
| NZ Mngt | privatisation | 4 | Neutral |
| NZ Mngt | processed | 4 | Neutral |
| NZ Mngt | profits | 4 | Neutral |
| NZ Mngt | promoted | 4 | Neutral |
| NZ Mngt | propose | 4 | Neutral |
| NZ Mngt | putt | 4 | Neutral |
| NZ Mngt | rather | 4 | Neutral |
| NZ Mngt | react | 4 | Neutral |
| NZ Mngt | recent | 4 | Neutral |
| NZ Mngt | recognition | 4 | Neutral |
| NZ Mngt | reflect | 4 | Neutral |
| NZ Mngt | regime | 4 | Neutral |
| NZ Mngt | reselve | 4 | Neutral |
| NZ Mngt | restore | 4 | Neutral |
| NZ Mngt | revised | 4 | Neutral |
| NZ Mngt | round | 4 | Neutral |
| NZ Mngt | rps | 4 | Neutral |
| NZ Mngt | seals | 4 | Neutral |
| NZ Mngt | selected | 4 | Neutral |
| NZ Mngt | sheltered | 4 | Neutral |
| NZ Mngt | sight | 4 | Neutral |
| NZ Mngt | similar | 4 | Neutral |
| NZ Mngt | situation | 4 | Neutral |
| NZ Mngt | social | 4 | Neutral |
| NZ Mngt | soon | 4 | Neutral |
| NZ Mngt | started | 4 | Neutral |
| NZ Mngt | subparts | 4 | Neutral |
| NZ Mngt | sure | 4 | Neutral |
| NZ Mngt | tania | 4 | Neutral |
| NZ Mngt | tons | 4 | Neutral |
| NZ Mngt | tour | 4 | Neutral |
| NZ Mngt | try | 4 | Neutral |
| NZ Mngt | upgrade | 4 | Neutral |
| NZ Mngt | variation | 4 | Neutral |
| NZ Mngt | vegetation | 4 | Neutral |
| NZ Mngt | view | 4 | Neutral |
| NZ Mngt | vision | 4 | Neutral |
| NZ Mngt | whole | 4 | Neutral |
| NZ Mngt | wont | 4 | Neutral |
| NZ Mngt | yrs | 4 | Neutral |
| NZ Mngt | aim | 3 | Neutral |
| NZ Mngt | alloc | 3 | Neutral |
| NZ Mngt | allotted | 3 | Neutral |
| NZ Mngt | alocate | 3 | Neutral |
| NZ Mngt | although | 3 | Neutral |
| NZ Mngt | arapawa | 3 | Neutral |
| NZ Mngt | arguments | 3 | Neutral |
| NZ Mngt | aspects | 3 | Neutral |
| NZ Mngt | associated | 3 | Neutral |
| NZ Mngt | association | 3 | Neutral |
| NZ Mngt | atiawa | 3 | Neutral |
| NZ Mngt | attitude | 3 | Neutral |
| NZ Mngt | author | 3 | Neutral |
| NZ Mngt | aware | 3 | Neutral |
| NZ Mngt | away | 3 | Neutral |
| NZ Mngt | begin | 3 | Neutral |
| NZ Mngt | boat | 3 | Neutral |
| NZ Mngt | bush | 3 | Neutral |
| NZ Mngt | buy | 3 | Neutral |
| NZ Mngt | ceastal | 3 | Neutral |
| NZ Mngt | central | 3 | Neutral |
| NZ Mngt | centre | 3 | Neutral |
| NZ Mngt | certain | 3 | Neutral |
| NZ Mngt | chance | 3 | Neutral |
| NZ Mngt | clause | 3 | Neutral |
| NZ Mngt | coastline | 3 | Neutral |
| NZ Mngt | codicils | 3 | Neutral |
| NZ Mngt | commercialise | 3 | Neutral |
| NZ Mngt | compell | 3 | Neutral |
| NZ Mngt | consultative | 3 | Neutral |
| NZ Mngt | counter | 3 | Neutral |
| NZ Mngt | crowns | 3 | Neutral |
| NZ Mngt | dear | 3 | Neutral |
| NZ Mngt | dependant | 3 | Neutral |
| NZ Mngt | described | 3 | Neutral |
| NZ Mngt | designation | 3 | Neutral |
| NZ Mngt | desire | 3 | Neutral |
| NZ Mngt | determine | 3 | Neutral |
| NZ Mngt | discussions | 3 | Neutral |
| NZ Mngt | disincentive | 3 | Neutral |
| NZ Mngt | enacted | 3 | Neutral |
| NZ Mngt | enormous | 3 | Neutral |
| NZ Mngt | event | 3 | Neutral |
| NZ Mngt | everyone | 3 | Neutral |
| NZ Mngt | existence | 3 | Neutral |
| NZ Mngt | extent | 3 | Neutral |
| NZ Mngt | fanns | 3 | Neutral |
| NZ Mngt | fax | 3 | Neutral |
| NZ Mngt | fishery | 3 | Neutral |
| NZ Mngt | flood | 3 | Neutral |
| NZ Mngt | foreign | 3 | Neutral |
| NZ Mngt | found | 3 | Neutral |
| NZ Mngt | fundamentally | 3 | Neutral |
| NZ Mngt | grant | 3 | Neutral |
| NZ Mngt | high | 3 | Neutral |
| NZ Mngt | human | 3 | Neutral |
| NZ Mngt | imperative | 3 | Neutral |
| NZ Mngt | inc | 3 | Neutral |
| NZ Mngt | incentive | 3 | Neutral |
| NZ Mngt | insert | 3 | Neutral |
| NZ Mngt | instance | 3 | Neutral |
| NZ Mngt | inviting | 3 | Neutral |
| NZ Mngt | known | 3 | Neutral |
| NZ Mngt | kopi | 3 | Neutral |
| NZ Mngt | landscape | 3 | Neutral |
| NZ Mngt | lease | 3 | Neutral |
| NZ Mngt | less | 3 | Neutral |
| NZ Mngt | locally | 3 | Neutral |
| NZ Mngt | locals | 3 | Neutral |
| NZ Mngt | look | 3 | Neutral |
| NZ Mngt | ltd | 3 | Neutral |
| NZ Mngt | lwi | 3 | Neutral |
| NZ Mngt | malaysian | 3 | Neutral |
| NZ Mngt | manager | 3 | Neutral |
| NZ Mngt | massive | 3 | Neutral |
| NZ Mngt | mean | 3 | Neutral |
| NZ Mngt | met | 3 | Neutral |
| NZ Mngt | moment | 3 | Neutral |
| NZ Mngt | notified | 3 | Neutral |
| NZ Mngt | obtain | 3 | Neutral |
| NZ Mngt | occur | 3 | Neutral |
| NZ Mngt | oper | 3 | Neutral |
| NZ Mngt | pace | 3 | Neutral |
| NZ Mngt | participation | 3 | Neutral |
| NZ Mngt | pay | 3 | Neutral |
| NZ Mngt | perhaps | 3 | Neutral |
| NZ Mngt | permanent | 3 | Neutral |
| NZ Mngt | phases | 3 | Neutral |
| NZ Mngt | placed | 3 | Neutral |
| NZ Mngt | preamble | 3 | Neutral |
| NZ Mngt | premature | 3 | Neutral |
| NZ Mngt | prepared | 3 | Neutral |
| NZ Mngt | probably | 3 | Neutral |
| NZ Mngt | production | 3 | Neutral |
| NZ Mngt | products | 3 | Neutral |
| NZ Mngt | protected | 3 | Neutral |
| NZ Mngt | provid | 3 | Neutral |
| NZ Mngt | provision | 3 | Neutral |
| NZ Mngt | publicly | 3 | Neutral |
| NZ Mngt | raised | 3 | Neutral |
| NZ Mngt | ratepayer | 3 | Neutral |
| NZ Mngt | realisation | 3 | Neutral |
| NZ Mngt | realise | 3 | Neutral |
| NZ Mngt | recreation | 3 | Neutral |
| NZ Mngt | regarded | 3 | Neutral |
| NZ Mngt | regardless | 3 | Neutral |
| NZ Mngt | relevant | 3 | Neutral |
| NZ Mngt | rul | 3 | Neutral |
| NZ Mngt | schedules | 3 | Neutral |
| NZ Mngt | secondly | 3 | Neutral |
| NZ Mngt | seek | 3 | Neutral |
| NZ Mngt | september | 3 | Neutral |
| NZ Mngt | several | 3 | Neutral |
| NZ Mngt | simply | 3 | Neutral |
| NZ Mngt | sincer | 3 | Neutral |
| NZ Mngt | small | 3 | Neutral |
| NZ Mngt | solution | 3 | Neutral |
| NZ Mngt | special | 3 | Neutral |
| NZ Mngt | stop | 3 | Neutral |
| NZ Mngt | strongly | 3 | Neutral |
| NZ Mngt | ths | 3 | Neutral |
| NZ Mngt | timeconsuming | 3 | Neutral |
| NZ Mngt | time | 3 | Neutral |
| NZ Mngt | tory | 3 | Neutral |
| NZ Mngt | transfer | 3 | Neutral |
| NZ Mngt | transferred | 3 | Neutral |
| NZ Mngt | understand | 3 | Neutral |
| NZ Mngt | understand | 3 | Neutral |
| NZ Mngt | undertak | 3 | Neutral |
| NZ Mngt | underwood | 3 | Neutral |
| NZ Mngt | various | 3 | Neutral |
| NZ Mngt | views | 3 | Neutral |
| NZ Mngt | want | 3 | Neutral |
| NZ Mngt | ways | 3 | Neutral |
| NZ Mngt | wherever | 3 | Neutral |
| NZ Mngt | whose | 3 | Neutral |
| NZ Mngt | widely | 3 | Neutral |
| NZ Mngt | absorbed | 2 | Neutral |
| NZ Mngt | acceptable | 2 | Neutral |
| NZ Mngt | accountability | 2 | Neutral |
| NZ Mngt | ace | 2 | Neutral |
| NZ Mngt | acted | 2 | Neutral |
| NZ Mngt | actives | 2 | Neutral |
| NZ Mngt | administer | 2 | Neutral |
| NZ Mngt | aesthetic | 2 | Neutral |
| NZ Mngt | almost | 2 | Neutral |
| NZ Mngt | ambulance | 2 | Neutral |
| NZ Mngt | among | 2 | Neutral |
| NZ Mngt | anchorage | 2 | Neutral |
| NZ Mngt | anywhere | 2 | Neutral |
| NZ Mngt | apparent | 2 | Neutral |
| NZ Mngt | appeals | 2 | Neutral |
| NZ Mngt | architect | 2 | Neutral |
| NZ Mngt | arise | 2 | Neutral |
| NZ Mngt | ask | 2 | Neutral |
| NZ Mngt | assess | 2 | Neutral |
| NZ Mngt | assured | 2 | Neutral |
| NZ Mngt | assuredness | 2 | Neutral |
| NZ Mngt | authority | 2 | Neutral |
| NZ Mngt | authorization | 2 | Neutral |
| NZ Mngt | avoids | 2 | Neutral |
| NZ Mngt | bank | 2 | Neutral |
| NZ Mngt | beech | 2 | Neutral |
| NZ Mngt | believes | 2 | Neutral |
| NZ Mngt | bid | 2 | Neutral |
| NZ Mngt | birds | 2 | Neutral |
| NZ Mngt | boaties | 2 | Neutral |
| NZ Mngt | boats | 2 | Neutral |
| NZ Mngt | book | 2 | Neutral |
| NZ Mngt | books | 2 | Neutral |
| NZ Mngt | bottom | 2 | Neutral |
| NZ Mngt | bray | 2 | Neutral |
| NZ Mngt | br | 2 | Neutral |
| NZ Mngt | broader | 2 | Neutral |
| NZ Mngt | bromide | 2 | Neutral |
| NZ Mngt | built | 2 | Neutral |
| NZ Mngt | bullied | 2 | Neutral |
| NZ Mngt | came | 2 | Neutral |
| NZ Mngt | canada | 2 | Neutral |
| NZ Mngt | cash | 2 | Neutral |
| NZ Mngt | cencil | 2 | Neutral |
| NZ Mngt | challenge | 2 | Neutral |
| NZ Mngt | channel | 2 | Neutral |
| NZ Mngt | chile | 2 | Neutral |
| NZ Mngt | cliff | 2 | Neutral |
| NZ Mngt | clitch | 2 | Neutral |
| NZ Mngt | cmz | 2 | Neutral |
| NZ Mngt | coal | 2 | Neutral |
| NZ Mngt | column | 2 | Neutral |
| NZ Mngt | combined | 2 | Neutral |
| NZ Mngt | comment | 2 | Neutral |
| NZ Mngt | commissioner | 2 | Neutral |
| NZ Mngt | completely | 2 | Neutral |
| NZ Mngt | condoned | 2 | Neutral |
| NZ Mngt | confer | 2 | Neutral |
| NZ Mngt | confirm | 2 | Neutral |
| NZ Mngt | conserve | 2 | Neutral |
| NZ Mngt | constantly | 2 | Neutral |
| NZ Mngt | consult | 2 | Neutral |
| NZ Mngt | consultants | 2 | Neutral |
| NZ Mngt | contents | 2 | Neutral |
| NZ Mngt | contestability | 2 | Neutral |
| NZ Mngt | contested | 2 | Neutral |
| NZ Mngt | continually | 2 | Neutral |
| NZ Mngt | continues | 2 | Neutral |
| NZ Mngt | convince | 2 | Neutral |
| NZ Mngt | cope | 2 | Neutral |
| NZ Mngt | corrects | 2 | Neutral |
| NZ Mngt | couple | 2 | Neutral |
| NZ Mngt | course | 2 | Neutral |
| NZ Mngt | cove | 2 | Neutral |
| NZ Mngt | cover | 2 | Neutral |
| NZ Mngt | crayfish | 2 | Neutral |
| NZ Mngt | crossreference | 2 | Neutral |
| NZ Mngt | cruis | 2 | Neutral |
| NZ Mngt | customary | 2 | Neutral |
| NZ Mngt | dad | 2 | Neutral |
| NZ Mngt | dated | 2 | Neutral |
| NZ Mngt | david | 2 | Neutral |
| NZ Mngt | day | 2 | Neutral |
| NZ Mngt | december | 2 | Neutral |
| NZ Mngt | defined | 2 | Neutral |
| NZ Mngt | definitely | 2 | Neutral |
| NZ Mngt | deletion | 2 | Neutral |
| NZ Mngt | denise | 2 | Neutral |
| NZ Mngt | designed | 2 | Neutral |
| NZ Mngt | determination | 2 | Neutral |
| NZ Mngt | determined | 2 | Neutral |
| NZ Mngt | developers | 2 | Neutral |
| NZ Mngt | diluted | 2 | Neutral |
| NZ Mngt | disast | 2 | Neutral |
| NZ Mngt | discretionary | 2 | Neutral |
| NZ Mngt | dispersed | 2 | Neutral |
| NZ Mngt | distributed | 2 | Neutral |
| NZ Mngt | divers | 2 | Neutral |
| NZ Mngt | docs | 2 | Neutral |
| NZ Mngt | dollars | 2 | Neutral |
| NZ Mngt | domain | 2 | Neutral |
| NZ Mngt | draft | 2 | Neutral |
| NZ Mngt | drafted | 2 | Neutral |
| NZ Mngt | dredg | 2 | Neutral |
| NZ Mngt | driven | 2 | Neutral |
| NZ Mngt | duty | 2 | Neutral |
| NZ Mngt | early | 2 | Neutral |
| NZ Mngt | earner | 2 | Neutral |
| NZ Mngt | ebcs | 2 | Neutral |
| NZ Mngt | eight | 2 | Neutral |
| NZ Mngt | either | 2 | Neutral |
| NZ Mngt | elaims | 2 | Neutral |
| NZ Mngt | eliminate | 2 | Neutral |
| NZ Mngt | enables | 2 | Neutral |
| NZ Mngt | enabl | 2 | Neutral |
| NZ Mngt | enactment | 2 | Neutral |
| NZ Mngt | endangered | 2 | Neutral |
| NZ Mngt | entirety | 2 | Neutral |
| NZ Mngt | entities | 2 | Neutral |
| NZ Mngt | entry | 2 | Neutral |
| NZ Mngt | equity | 2 | Neutral |
| NZ Mngt | equivalent | 2 | Neutral |
| NZ Mngt | eradication | 2 | Neutral |
| NZ Mngt | ermit | 2 | Neutral |
| NZ Mngt | essential | 2 | Neutral |
| NZ Mngt | etc | 2 | Neutral |
| NZ Mngt | europe | 2 | Neutral |
| NZ Mngt | evaluated | 2 | Neutral |
| NZ Mngt | evaluation | 2 | Neutral |
| NZ Mngt | evidence | 2 | Neutral |
| NZ Mngt | examination | 2 | Neutral |
| NZ Mngt | execution | 2 | Neutral |
| NZ Mngt | expectation | 2 | Neutral |
| NZ Mngt | expert | 2 | Neutral |
| NZ Mngt | exported | 2 | Neutral |
| NZ Mngt | extensions | 2 | Neutral |
| NZ Mngt | extensive | 2 | Neutral |
| NZ Mngt | factories | 2 | Neutral |
| NZ Mngt | factor | 2 | Neutral |
| NZ Mngt | family | 2 | Neutral |
| NZ Mngt | fanning | 2 | Neutral |
| NZ Mngt | farmer | 2 | Neutral |
| NZ Mngt | fauna | 2 | Neutral |
| NZ Mngt | features | 2 | Neutral |
| NZ Mngt | feed | 2 | Neutral |
| NZ Mngt | fer | 2 | Neutral |
| NZ Mngt | ferries | 2 | Neutral |
| NZ Mngt | fester | 2 | Neutral |
| NZ Mngt | fin | 2 | Neutral |
| NZ Mngt | finalised | 2 | Neutral |
| NZ Mngt | find | 2 | Neutral |
| NZ Mngt | firstly | 2 | Neutral |
| NZ Mngt | fishermen | 2 | Neutral |
| NZ Mngt | flow | 2 | Neutral |
| NZ Mngt | folly | 2 | Neutral |
| NZ Mngt | forebears | 2 | Neutral |
| NZ Mngt | foreshadow | 2 | Neutral |
| NZ Mngt | forestry | 2 | Neutral |
| NZ Mngt | framing | 2 | Neutral |
| NZ Mngt | fully | 2 | Neutral |
| NZ Mngt | funded | 2 | Neutral |
| NZ Mngt | gclient | 2 | Neutral |
| NZ Mngt | gener | 2 | Neutral |
| NZ Mngt | generates | 2 | Neutral |
| NZ Mngt | goes | 2 | Neutral |
| NZ Mngt | governance | 2 | Neutral |
| NZ Mngt | govern | 2 | Neutral |
| NZ Mngt | grandchildren | 2 | Neutral |
| NZ Mngt | grant | 2 | Neutral |
| NZ Mngt | greatly | 2 | Neutral |
| NZ Mngt | grow | 2 | Neutral |
| NZ Mngt | harvest | 2 | Neutral |
| NZ Mngt | hearts | 2 | Neutral |
| NZ Mngt | heavily | 2 | Neutral |
| NZ Mngt | hectors | 2 | Neutral |
| NZ Mngt | held | 2 | Neutral |
| NZ Mngt | heritage | 2 | Neutral |
| NZ Mngt | histerie | 2 | Neutral |
| NZ Mngt | holder | 2 | Neutral |
| NZ Mngt | holders | 2 | Neutral |
| NZ Mngt | holes | 2 | Neutral |
| NZ Mngt | hors | 2 | Neutral |
| NZ Mngt | hotly | 2 | Neutral |
| NZ Mngt | idly | 2 | Neutral |
| NZ Mngt | immediately | 2 | Neutral |
| NZ Mngt | implore | 2 | Neutral |
| NZ Mngt | indeed | 2 | Neutral |
| NZ Mngt | influence | 2 | Neutral |
| NZ Mngt | informed | 2 | Neutral |
| NZ Mngt | inlets | 2 | Neutral |
| NZ Mngt | input | 2 | Neutral |
| NZ Mngt | instigated | 2 | Neutral |
| NZ Mngt | instructed | 2 | Neutral |
| NZ Mngt | integrity | 2 | Neutral |
| NZ Mngt | interim | 2 | Neutral |
| NZ Mngt | international | 2 | Neutral |
| NZ Mngt | jockey | 2 | Neutral |
| NZ Mngt | kelp | 2 | Neutral |
| NZ Mngt | key | 2 | Neutral |
| NZ Mngt | knowledge | 2 | Neutral |
| NZ Mngt | layer | 2 | Neutral |
| NZ Mngt | leav | 2 | Neutral |
| NZ Mngt | legal | 2 | Neutral |
| NZ Mngt | let | 2 | Neutral |
| NZ Mngt | lets | 2 | Neutral |
| NZ Mngt | lifetime | 2 | Neutral |
| NZ Mngt | light | 2 | Neutral |
| NZ Mngt | litigation | 2 | Neutral |
| NZ Mngt | little | 2 | Neutral |
| NZ Mngt | live | 2 | Neutral |
| NZ Mngt | located | 2 | Neutral |
| NZ Mngt | longer | 2 | Neutral |
| NZ Mngt | longterm | 2 | Neutral |
| NZ Mngt | loop | 2 | Neutral |
| NZ Mngt | lternative | 2 | Neutral |
| NZ Mngt | main | 2 | Neutral |
| NZ Mngt | manawhenua | 2 | Neutral |
| NZ Mngt | marinas | 2 | Neutral |
| NZ Mngt | market | 2 | Neutral |
| NZ Mngt | match | 2 | Neutral |
| NZ Mngt | meg | 2 | Neutral |
| NZ Mngt | methane | 2 | Neutral |
| NZ Mngt | methyl | 2 | Neutral |
| NZ Mngt | milford | 2 | Neutral |
| NZ Mngt | minds | 2 | Neutral |
| NZ Mngt | minimises | 2 | Neutral |
| NZ Mngt | minority | 2 | Neutral |
| NZ Mngt | moana | 2 | Neutral |
| NZ Mngt | mono | 2 | Neutral |
| NZ Mngt | monopoli | 2 | Neutral |
| NZ Mngt | moor | 2 | Neutral |
| NZ Mngt | motuara | 2 | Neutral |
| NZ Mngt | mum | 2 | Neutral |
| NZ Mngt | needed | 2 | Neutral |
| NZ Mngt | nitrogen | 2 | Neutral |
| NZ Mngt | non | 2 | Neutral |
| NZ Mngt | normal | 2 | Neutral |
| NZ Mngt | north | 2 | Neutral |
| NZ Mngt | notice | 2 | Neutral |
| NZ Mngt | numbers | 2 | Neutral |
| NZ Mngt | obligations | 2 | Neutral |
| NZ Mngt | occupation | 2 | Neutral |
| NZ Mngt | ocean | 2 | Neutral |
| NZ Mngt | oerson | 2 | Neutral |
| NZ Mngt | ofnew | 2 | Neutral |
| NZ Mngt | ofpublic | 2 | Neutral |
| NZ Mngt | operational | 2 | Neutral |
| NZ Mngt | outside | 2 | Neutral |
| NZ Mngt | overdraft | 2 | Neutral |
| NZ Mngt | overseas | 2 | Neutral |
| NZ Mngt | overturned | 2 | Neutral |
| NZ Mngt | parliament | 2 | Neutral |
| NZ Mngt | parliamentary | 2 | Neutral |
| NZ Mngt | participants | 2 | Neutral |
| NZ Mngt | partner | 2 | Neutral |
| NZ Mngt | pass | 2 | Neutral |
| NZ Mngt | paua | 2 | Neutral |
| NZ Mngt | pays | 2 | Neutral |
| NZ Mngt | peer | 2 | Neutral |
| NZ Mngt | perceived | 2 | Neutral |
| NZ Mngt | pete | 2 | Neutral |
| NZ Mngt | phas | 2 | Neutral |
| NZ Mngt | phosphate | 2 | Neutral |
| NZ Mngt | picton | 2 | Neutral |
| NZ Mngt | pike | 2 | Neutral |
| NZ Mngt | pine | 2 | Neutral |
| NZ Mngt | pioneered | 2 | Neutral |
| NZ Mngt | pipe | 2 | Neutral |
| NZ Mngt | placate | 2 | Neutral |
| NZ Mngt | places | 2 | Neutral |
| NZ Mngt | planned | 2 | Neutral |
| NZ Mngt | planner | 2 | Neutral |
| NZ Mngt | play | 2 | Neutral |
| NZ Mngt | points | 2 | Neutral |
| NZ Mngt | possibility | 2 | Neutral |
| NZ Mngt | powder | 2 | Neutral |
| NZ Mngt | practices | 2 | Neutral |
| NZ Mngt | predisposed | 2 | Neutral |
| NZ Mngt | preferential | 2 | Neutral |
| NZ Mngt | preliminary | 2 | Neutral |
| NZ Mngt | presence | 2 | Neutral |
| NZ Mngt | presumption | 2 | Neutral |
| NZ Mngt | primarily | 2 | Neutral |
| NZ Mngt | pristine | 2 | Neutral |
| NZ Mngt | proceed | 2 | Neutral |
| NZ Mngt | proceed | 2 | Neutral |
| NZ Mngt | product | 2 | Neutral |
| NZ Mngt | propos | 2 | Neutral |
| NZ Mngt | provinces | 2 | Neutral |
| NZ Mngt | provis | 2 | Neutral |
| NZ Mngt | publics | 2 | Neutral |
| NZ Mngt | publish | 2 | Neutral |
| NZ Mngt | purpose | 2 | Neutral |
| NZ Mngt | pursue | 2 | Neutral |
| NZ Mngt | push | 2 | Neutral |
| NZ Mngt | quality | 2 | Neutral |
| NZ Mngt | quite | 2 | Neutral |
| NZ Mngt | raise | 2 | Neutral |
| NZ Mngt | reach | 2 | Neutral |
| NZ Mngt | reader | 2 | Neutral |
| NZ Mngt | recovers | 2 | Neutral |
| NZ Mngt | reduc | 2 | Neutral |
| NZ Mngt | reengineered | 2 | Neutral |
| NZ Mngt | references | 2 | Neutral |
| NZ Mngt | regulate | 2 | Neutral |
| NZ Mngt | release | 2 | Neutral |
| NZ Mngt | rely | 2 | Neutral |
| NZ Mngt | replacement | 2 | Neutral |
| NZ Mngt | representative | 2 | Neutral |
| NZ Mngt | representativeness | 2 | Neutral |
| NZ Mngt | represents | 2 | Neutral |
| NZ Mngt | reservations | 2 | Neutral |
| NZ Mngt | resort | 2 | Neutral |
| NZ Mngt | responsibility | 2 | Neutral |
| NZ Mngt | retain | 2 | Neutral |
| NZ Mngt | reversed | 2 | Neutral |
| NZ Mngt | reviews | 2 | Neutral |
| NZ Mngt | rewrite | 2 | Neutral |
| NZ Mngt | river | 2 | Neutral |
| NZ Mngt | roles | 2 | Neutral |
| NZ Mngt | rule | 2 | Neutral |
| NZ Mngt | run | 2 | Neutral |
| NZ Mngt | runs | 2 | Neutral |
| NZ Mngt | saddens | 2 | Neutral |
| NZ Mngt | sanctuary | 2 | Neutral |
| NZ Mngt | sanfords | 2 | Neutral |
| NZ Mngt | seal | 2 | Neutral |
| NZ Mngt | sealords | 2 | Neutral |
| NZ Mngt | seems | 2 | Neutral |
| NZ Mngt | select | 2 | Neutral |
| NZ Mngt | sell | 2 | Neutral |
| NZ Mngt | sent | 2 | Neutral |
| NZ Mngt | series | 2 | Neutral |
| NZ Mngt | served | 2 | Neutral |
| NZ Mngt | settle | 2 | Neutral |
| NZ Mngt | share | 2 | Neutral |
| NZ Mngt | shift | 2 | Neutral |
| NZ Mngt | ship | 2 | Neutral |
| NZ Mngt | shore | 2 | Neutral |
| NZ Mngt | sights | 2 | Neutral |
| NZ Mngt | silt | 2 | Neutral |
| NZ Mngt | sit | 2 | Neutral |
| NZ Mngt | size | 2 | Neutral |
| NZ Mngt | societi | 2 | Neutral |
| NZ Mngt | society | 2 | Neutral |
| NZ Mngt | somehow | 2 | Neutral |
| NZ Mngt | soundsare | 2 | Neutral |
| NZ Mngt | speak | 2 | Neutral |
| NZ Mngt | spent | 2 | Neutral |
| NZ Mngt | sqace | 2 | Neutral |
| NZ Mngt | staff | 2 | Neutral |
| NZ Mngt | stagger | 2 | Neutral |
| NZ Mngt | start | 2 | Neutral |
| NZ Mngt | stated | 2 | Neutral |
| NZ Mngt | steps | 2 | Neutral |
| NZ Mngt | street | 2 | Neutral |
| NZ Mngt | stuff | 2 | Neutral |
| NZ Mngt | subdivision | 2 | Neutral |
| NZ Mngt | subiect | 2 | Neutral |
| NZ Mngt | subsidise | 2 | Neutral |
| NZ Mngt | succession | 2 | Neutral |
| NZ Mngt | suggestion | 2 | Neutral |
| NZ Mngt | summari | 2 | Neutral |
| NZ Mngt | summer | 2 | Neutral |
| NZ Mngt | supply | 2 | Neutral |
| NZ Mngt | surround | 2 | Neutral |
| NZ Mngt | talcum | 2 | Neutral |
| NZ Mngt | talleys | 2 | Neutral |
| NZ Mngt | task | 2 | Neutral |
| NZ Mngt | tau | 2 | Neutral |
| NZ Mngt | technology | 2 | Neutral |
| NZ Mngt | tel | 2 | Neutral |
| NZ Mngt | temporary | 2 | Neutral |
| NZ Mngt | tena | 2 | Neutral |
| NZ Mngt | tenders | 2 | Neutral |
| NZ Mngt | test | 2 | Neutral |
| NZ Mngt | thereby | 2 | Neutral |
| NZ Mngt | thorough | 2 | Neutral |
| NZ Mngt | thousands | 2 | Neutral |
| NZ Mngt | throu | 2 | Neutral |
| NZ Mngt | thus | 2 | Neutral |
| NZ Mngt | tidal | 2 | Neutral |
| NZ Mngt | tile | 2 | Neutral |
| NZ Mngt | timeframe | 2 | Neutral |
| NZ Mngt | tlie | 2 | Neutral |
| NZ Mngt | towards | 2 | Neutral |
| NZ Mngt | town | 2 | Neutral |
| NZ Mngt | transparency | 2 | Neutral |
| NZ Mngt | trawl | 2 | Neutral |
| NZ Mngt | trucks | 2 | Neutral |
| NZ Mngt | try | 2 | Neutral |
| NZ Mngt | uaculture | 2 | Neutral |
| NZ Mngt | undergone | 2 | Neutral |
| NZ Mngt | underway | 2 | Neutral |
| NZ Mngt | unduly | 2 | Neutral |
| NZ Mngt | unenviable | 2 | Neutral |
| NZ Mngt | unintended | 2 | Neutral |
| NZ Mngt | unique | 2 | Neutral |
| NZ Mngt | usa | 2 | Neutral |
| NZ Mngt | vast | 2 | Neutral |
| NZ Mngt | via | 2 | Neutral |
| NZ Mngt | village | 2 | Neutral |
| NZ Mngt | visual | 2 | Neutral |
| NZ Mngt | vol | 2 | Neutral |
| NZ Mngt | vve | 2 | Neutral |
| NZ Mngt | wairauawater | 2 | Neutral |
| NZ Mngt | waitangi | 2 | Neutral |
| NZ Mngt | walkers | 2 | Neutral |
| NZ Mngt | warmp | 2 | Neutral |
| NZ Mngt | wasnt | 2 | Neutral |
| NZ Mngt | waterways | 2 | Neutral |
| NZ Mngt | wave | 2 | Neutral |
| NZ Mngt | west | 2 | Neutral |
| NZ Mngt | wharf | 2 | Neutral |
| NZ Mngt | whatever | 2 | Neutral |
| NZ Mngt | whereby | 2 | Neutral |
| NZ Mngt | whilst | 2 | Neutral |
| NZ Mngt | witnessed | 2 | Neutral |
| NZ Mngt | witnesses | 2 | Neutral |
| NZ Mngt | wouldnt | 2 | Neutral |
| NZ Mngt | write | 2 | Neutral |
| NZ Mngt | right | 36 | Positive |
| NZ Mngt | interest | 32 | Positive |
| NZ Mngt | fair | 30 | Positive |
| NZ Mngt | support | 28 | Positive |
| NZ Mngt | available | 26 | Positive |
| NZ Mngt | appropriate | 22 | Positive |
| NZ Mngt | efficient | 21 | Positive |
| NZ Mngt | important | 19 | Positive |
| NZ Mngt | sustainable | 19 | Positive |
| NZ Mngt | reform | 18 | Positive |
| NZ Mngt | benefit | 16 | Positive |
| NZ Mngt | effective | 16 | Positive |
| NZ Mngt | trust | 15 | Positive |
| NZ Mngt | encourage | 14 | Positive |
| NZ Mngt | significant | 14 | Positive |
| NZ Mngt | best | 13 | Positive |
| NZ Mngt | better | 12 | Positive |
| NZ Mngt | effectively | 11 | Positive |
| NZ Mngt | approval | 10 | Positive |
| NZ Mngt | enough | 10 | Positive |
| NZ Mngt | proper | 10 | Positive |
| NZ Mngt | protect | 10 | Positive |
| NZ Mngt | successful | 10 | Positive |
| NZ Mngt | guidance | 8 | Positive |
| NZ Mngt | sufficient | 8 | Positive |
| NZ Mngt | beauty | 7 | Positive |
| NZ Mngt | great | 7 | Positive |
| NZ Mngt | lead | 7 | Positive |
| NZ Mngt | recommend | 7 | Positive |
| NZ Mngt | robust | 7 | Positive |
| NZ Mngt | favour | 6 | Positive |
| NZ Mngt | integrated | 6 | Positive |
| NZ Mngt | properly | 6 | Positive |
| NZ Mngt | succeed | 6 | Positive |
| NZ Mngt | wealthy | 6 | Positive |
| NZ Mngt | regard | 5 | Positive |
| NZ Mngt | respect | 5 | Positive |
| NZ Mngt | accurately | 4 | Positive |
| NZ Mngt | advocate | 4 | Positive |
| NZ Mngt | appeal | 4 | Positive |
| NZ Mngt | award | 4 | Positive |
| NZ Mngt | clearly | 4 | Positive |
| NZ Mngt | enhancement | 4 | Positive |
| NZ Mngt | facilitate | 4 | Positive |
| NZ Mngt | free | 4 | Positive |
| NZ Mngt | gold | 4 | Positive |
| NZ Mngt | improve | 4 | Positive |
| NZ Mngt | unfettered | 4 | Positive |
| NZ Mngt | wonderful | 4 | Positive |
| NZ Mngt | help | 3 | Positive |
| NZ Mngt | work | 3 | Positive |
| NZ Mngt | adequate | 3 | Positive |
| NZ Mngt | advantage | 3 | Positive |
| NZ Mngt | afford | 3 | Positive |
| NZ Mngt | amenity | 3 | Positive |
| NZ Mngt | appreciate | 3 | Positive |
| NZ Mngt | clean | 3 | Positive |
| NZ Mngt | comprehensive | 3 | Positive |
| NZ Mngt | happy | 3 | Positive |
| NZ Mngt | optimal | 3 | Positive |
| NZ Mngt | valuable | 3 | Positive |
| NZ Mngt | win | 3 | Positive |
| NZ Mngt | outstand | 2 | Positive |
| NZ Mngt | balanced | 2 | Positive |
| NZ Mngt | beneficial | 2 | Positive |
| NZ Mngt | boost | 2 | Positive |
| NZ Mngt | clear | 2 | Positive |
| NZ Mngt | consistent | 2 | Positive |
| NZ Mngt | correct | 2 | Positive |
| NZ Mngt | enhance | 2 | Positive |
| NZ Mngt | enjoy | 2 | Positive |
| NZ Mngt | exceeds | 2 | Positive |
| NZ Mngt | fast | 2 | Positive |
| NZ Mngt | guarantee | 2 | Positive |
| NZ Mngt | healthy | 2 | Positive |
| NZ Mngt | immense | 2 | Positive |
| NZ Mngt | lawfully | 2 | Positive |
| NZ Mngt | led | 2 | Positive |
| NZ Mngt | precious | 2 | Positive |
| NZ Mngt | protection | 2 | Positive |
| NZ Mngt | reasonable | 2 | Positive |
| NZ Mngt | recovery | 2 | Positive |
| NZ Mngt | relief | 2 | Positive |
| NZ Mngt | satisfy | 2 | Positive |
| NZ Mngt | secure | 2 | Positive |
| NZ Mngt | sensible | 2 | Positive |
| NZ Mngt | sensitive | 2 | Positive |
| NZ Mngt | success | 2 | Positive |
| NZ Mngt | variety | 2 | Positive |
| NZ Mngt | virtue | 2 | Positive |
| NZ Mngt | welcome | 2 | Positive |
| NZ Policy | fishing | 394 | Negative |
| NZ Policy | cost | 346 | Negative |
| NZ Policy | limited | 317 | Negative |
| NZ Policy | concern | 303 | Negative |
| NZ Policy | adverse | 231 | Negative |
| NZ Policy | impact | 194 | Negative |
| NZ Policy | undue | 134 | Negative |
| NZ Policy | competition | 129 | Negative |
| NZ Policy | oppose | 116 | Negative |
| NZ Policy | restrict | 103 | Negative |
| NZ Policy | risk | 102 | Negative |
| NZ Policy | problem | 65 | Negative |
| NZ Policy | wildlife | 64 | Negative |
| NZ Policy | difficult | 60 | Negative |
| NZ Policy | discharge | 51 | Negative |
| NZ Policy | lack | 50 | Negative |
| NZ Policy | cons | 46 | Negative |
| NZ Policy | objection | 45 | Negative |
| NZ Policy | conflict | 42 | Negative |
| NZ Policy | unnecessary | 42 | Negative |
| NZ Policy | decline | 40 | Negative |
| NZ Policy | inappropriate | 37 | Negative |
| NZ Policy | absence | 34 | Negative |
| NZ Policy | object | 33 | Negative |
| NZ Policy | serious | 32 | Negative |
| NZ Policy | loss | 31 | Negative |
| NZ Policy | lost | 31 | Negative |
| NZ Policy | moratorium | 31 | Negative |
| NZ Policy | delay | 29 | Negative |
| NZ Policy | unable | 29 | Negative |
| NZ Policy | complex | 27 | Negative |
| NZ Policy | critical | 25 | Negative |
| NZ Policy | delay | 25 | Negative |
| NZ Policy | pest | 25 | Negative |
| NZ Policy | unclear | 25 | Negative |
| NZ Policy | unlikely | 25 | Negative |
| NZ Policy | doubt | 24 | Negative |
| NZ Policy | inconsistent | 23 | Negative |
| NZ Policy | lapse | 23 | Negative |
| NZ Policy | dead | 22 | Negative |
| NZ Policy | hard | 21 | Negative |
| NZ Policy | fail | 19 | Negative |
| NZ Policy | poor | 19 | Negative |
| NZ Policy | prohibit | 19 | Negative |
| NZ Policy | oversight | 18 | Negative |
| NZ Policy | wrong | 18 | Negative |
| NZ Policy | failed | 17 | Negative |
| NZ Policy | draconian | 16 | Negative |
| NZ Policy | stress | 16 | Negative |
| NZ Policy | failure | 15 | Negative |
| NZ Policy | mad | 15 | Negative |
| NZ Policy | mortality | 15 | Negative |
| NZ Policy | expensive | 14 | Negative |
| NZ Policy | waste | 14 | Negative |
| NZ Policy | illegal | 13 | Negative |
| NZ Policy | insufficient | 13 | Negative |
| NZ Policy | negative | 13 | Negative |
| NZ Policy | uncertain | 13 | Negative |
| NZ Policy | unreasonable | 13 | Negative |
| NZ Policy | burden | 12 | Negative |
| NZ Policy | complicated | 12 | Negative |
| NZ Policy | frozen | 12 | Negative |
| NZ Policy | inadequate | 12 | Negative |
| NZ Policy | interfere | 12 | Negative |
| NZ Policy | unacceptable | 12 | Negative |
| NZ Policy | unknown | 12 | Negative |
| NZ Policy | damage | 11 | Negative |
| NZ Policy | death | 11 | Negative |
| NZ Policy | intrusive | 11 | Negative |
| NZ Policy | losses | 11 | Negative |
| NZ Policy | unworkable | 11 | Negative |
| NZ Policy | ignore | 10 | Negative |
| NZ Policy | inevitably | 10 | Negative |
| NZ Policy | limitation | 10 | Negative |
| NZ Policy | onerous | 10 | Negative |
| NZ Policy | pollution | 10 | Negative |
| NZ Policy | uncertainty | 10 | Negative |
| NZ Policy | undemocratic | 10 | Negative |
| NZ Policy | amend | 2122 | Neutral |
| NZ Policy | area | 1344 | Neutral |
| NZ Policy | bill | 1275 | Neutral |
| NZ Policy | coastal | 1170 | Neutral |
| NZ Policy | council | 1003 | Neutral |
| NZ Policy | consent | 999 | Neutral |
| NZ Policy | application | 888 | Neutral |
| NZ Policy | process | 875 | Neutral |
| NZ Policy | provide | 793 | Neutral |
| NZ Policy | decision | 775 | Neutral |
| NZ Policy | regional | 771 | Neutral |
| NZ Policy | develop | 758 | Neutral |
| NZ Policy | activity | 737 | Neutral |
| NZ Policy | plan | 732 | Neutral |
| NZ Policy | uae | 715 | Neutral |
| NZ Policy | act | 705 | Neutral |
| NZ Policy | require | 698 | Neutral |
| NZ Policy | change | 696 | Neutral |
| NZ Policy | environment | 684 | Neutral |
| NZ Policy | fisheries | 677 | Neutral |
| NZ Policy | quota | 669 | Neutral |
| NZ Policy | industry | 659 | Neutral |
| NZ Policy | resource | 648 | Neutral |
| NZ Policy | agree | 637 | Neutral |
| NZ Policy | rma | 606 | Neutral |
| NZ Policy | submission | 601 | Neutral |
| NZ Policy | management | 563 | Neutral |
| NZ Policy | zealand | 562 | Neutral |
| NZ Policy | negotiate | 560 | Neutral |
| NZ Policy | legislation | 553 | Neutral |
| NZ Policy | clause | 548 | Neutral |
| NZ Policy | applicant | 512 | Neutral |
| NZ Policy | space | 509 | Neutral |
| NZ Policy | include | 501 | Neutral |
| NZ Policy | effects | 500 | Neutral |
| NZ Policy | provision | 496 | Neutral |
| NZ Policy | owner | 490 | Neutral |
| NZ Policy | permit | 473 | Neutral |
| NZ Policy | minister | 457 | Neutral |
| NZ Policy | government | 433 | Neutral |
| NZ Policy | committee | 431 | Neutral |
| NZ Policy | exist | 431 | Neutral |
| NZ Policy | commercial | 404 | Neutral |
| NZ Policy | issue | 395 | Neutral |
| NZ Policy | must | 388 | Neutral |
| NZ Policy | allow | 376 | Neutral |
| NZ Policy | give | 372 | Neutral |
| NZ Policy | bay | 363 | Neutral |
| NZ Policy | mussel | 363 | Neutral |
| NZ Policy | made | 361 | Neutral |
| NZ Policy | part | 349 | Neutral |
| NZ Policy | effect | 339 | Neutral |
| NZ Policy | years | 333 | Neutral |
| NZ Policy | affect | 324 | Neutral |
| NZ Policy | policy | 318 | Neutral |
| NZ Policy | settlement | 316 | Neutral |
| NZ Policy | applied | 313 | Neutral |
| NZ Policy | use | 306 | Neutral |
| NZ Policy | allocate | 302 | Neutral |
| NZ Policy | court | 296 | Neutral |
| NZ Policy | local | 285 | Neutral |
| NZ Policy | add | 281 | Neutral |
| NZ Policy | follow | 280 | Neutral |
| NZ Policy | high | 280 | Neutral |
| NZ Policy | make | 279 | Neutral |
| NZ Policy | current | 277 | Neutral |
| NZ Policy | public | 273 | Neutral |
| NZ Policy | need | 265 | Neutral |
| NZ Policy | report | 263 | Neutral |
| NZ Policy | plans | 262 | Neutral |
| NZ Policy | group | 261 | Neutral |
| NZ Policy | tasman | 259 | Neutral |
| NZ Policy | however | 256 | Neutral |
| NZ Policy | marlborough | 254 | Neutral |
| NZ Policy | opportunity | 245 | Neutral |
| NZ Policy | determine | 243 | Neutral |
| NZ Policy | coromandel | 241 | Neutral |
| NZ Policy | ensure | 240 | Neutral |
| NZ Policy | sanford | 234 | Neutral |
| NZ Policy | work | 231 | Neutral |
| NZ Policy | farmer | 230 | Neutral |
| NZ Policy | particular | 229 | Neutral |
| NZ Policy | mfish | 228 | Neutral |
| NZ Policy | test | 227 | Neutral |
| NZ Policy | proposal | 225 | Neutral |
| NZ Policy | address | 224 | Neutral |
| NZ Policy | production | 222 | Neutral |
| NZ Policy | iwi | 219 | Neutral |
| NZ Policy | remove | 219 | Neutral |
| NZ Policy | request | 219 | Neutral |
| NZ Policy | plan | 213 | Neutral |
| NZ Policy | potential | 213 | Neutral |
| NZ Policy | salmon | 210 | Neutral |
| NZ Policy | stock | 209 | Neutral |
| NZ Policy | establish | 206 | Neutral |
| NZ Policy | sound | 205 | Neutral |
| NZ Policy | consider | 203 | Neutral |
| NZ Policy | relation | 195 | Neutral |
| NZ Policy | introduce | 194 | Neutral |
| NZ Policy | assessment | 192 | Neutral |
| NZ Policy | make | 191 | Neutral |
| NZ Policy | waikato | 191 | Neutral |
| NZ Policy | select | 190 | Neutral |
| NZ Policy | create | 186 | Neutral |
| NZ Policy | review | 185 | Neutral |
| NZ Policy | amas | 182 | Neutral |
| NZ Policy | submiss | 181 | Neutral |
| NZ Policy | district | 178 | Neutral |
| NZ Policy | whether | 178 | Neutral |
| NZ Policy | matters | 176 | Neutral |
| NZ Policy | able | 174 | Neutral |
| NZ Policy | recreational | 174 | Neutral |
| NZ Policy | process | 173 | Neutral |
| NZ Policy | authority | 172 | Neutral |
| NZ Policy | enable | 172 | Neutral |
| NZ Policy | executive | 172 | Neutral |
| NZ Policy | water | 172 | Neutral |
| NZ Policy | ministry | 171 | Neutral |
| NZ Policy | primary | 169 | Neutral |
| NZ Policy | provision | 167 | Neutral |
| NZ Policy | result | 166 | Neutral |
| NZ Policy | see | 166 | Neutral |
| NZ Policy | auckland | 165 | Neutral |
| NZ Policy | chief | 165 | Neutral |
| NZ Policy | mussel | 165 | Neutral |
| NZ Policy | approach | 164 | Neutral |
| NZ Policy | value | 162 | Neutral |
| NZ Policy | condition | 161 | Neutral |
| NZ Policy | reduce | 158 | Neutral |
| NZ Policy | continue | 154 | Neutral |
| NZ Policy | without | 153 | Neutral |
| NZ Policy | relevant | 152 | Neutral |
| NZ Policy | parties | 151 | Neutral |
| NZ Policy | seafic | 151 | Neutral |
| NZ Policy | used | 150 | Neutral |
| NZ Policy | case | 149 | Neutral |
| NZ Policy | rather | 148 | Neutral |
| NZ Policy | currently | 147 | Neutral |
| NZ Policy | regime | 147 | Neutral |
| NZ Policy | involve | 145 | Neutral |
| NZ Policy | power | 145 | Neutral |
| NZ Policy | large | 144 | Neutral |
| NZ Policy | order | 144 | Neutral |
| NZ Policy | detail | 143 | Neutral |
| NZ Policy | communal | 142 | Neutral |
| NZ Policy | set | 141 | Neutral |
| NZ Policy | submission | 141 | Neutral |
| NZ Policy | community | 140 | Neutral |
| NZ Policy | growth | 139 | Neutral |
| NZ Policy | method | 139 | Neutral |
| NZ Policy | occur | 138 | Neutral |
| NZ Policy | spat | 138 | Neutral |
| NZ Policy | economic | 137 | Neutral |
| NZ Policy | note | 137 | Neutral |
| NZ Policy | region | 137 | Neutral |
| NZ Policy | take | 135 | Neutral |
| NZ Policy | purpose | 134 | Neutral |
| NZ Policy | interest | 129 | Neutral |
| NZ Policy | likely | 129 | Neutral |
| NZ Policy | consideration | 127 | Neutral |
| NZ Policy | consider | 127 | Neutral |
| NZ Policy | law | 127 | Neutral |
| NZ Policy | stock | 127 | Neutral |
| NZ Policy | therefore | 127 | Neutral |
| NZ Policy | expand | 126 | Neutral |
| NZ Policy | heard | 126 | Neutral |
| NZ Policy | maori | 126 | Neutral |
| NZ Policy | believe | 125 | Neutral |
| NZ Policy | needs | 125 | Neutral |
| NZ Policy | option | 124 | Neutral |
| NZ Policy | crown | 123 | Neutral |
| NZ Policy | power | 123 | Neutral |
| NZ Policy | harvest | 122 | Neutral |
| NZ Policy | site | 122 | Neutral |
| NZ Policy | legal | 121 | Neutral |
| NZ Policy | mechanism | 121 | Neutral |
| NZ Policy | natural | 121 | Neutral |
| NZ Policy | prior | 121 | Neutral |
| NZ Policy | view | 121 | Neutral |
| NZ Policy | already | 120 | Neutral |
| NZ Policy | company | 120 | Neutral |
| NZ Policy | member | 120 | Neutral |
| NZ Policy | specific | 120 | Neutral |
| NZ Policy | demand | 119 | Neutral |
| NZ Policy | early | 119 | Neutral |
| NZ Policy | framework | 119 | Neutral |
| NZ Policy | sections | 119 | Neutral |
| NZ Policy | based | 118 | Neutral |
| NZ Policy | day | 118 | Neutral |
| NZ Policy | investment | 118 | Neutral |
| NZ Policy | considered | 117 | Neutral |
| NZ Policy | firth | 117 | Neutral |
| NZ Policy | potentially | 117 | Neutral |
| NZ Policy | achieve | 116 | Neutral |
| NZ Policy | itq | 116 | Neutral |
| NZ Policy | rules | 116 | Neutral |
| NZ Policy | ama | 115 | Neutral |
| NZ Policy | oyster | 115 | Neutral |
| NZ Policy | future | 114 | Neutral |
| NZ Policy | general | 114 | Neutral |
| NZ Policy | consultation | 113 | Neutral |
| NZ Policy | ohu | 113 | Neutral |
| NZ Policy | key | 112 | Neutral |
| NZ Policy | schedule | 112 | Neutral |
| NZ Policy | conservation | 111 | Neutral |
| NZ Policy | place | 111 | Neutral |
| NZ Policy | relate | 108 | Neutral |
| NZ Policy | holder | 107 | Neutral |
| NZ Policy | land | 107 | Neutral |
| NZ Policy | legislative | 107 | Neutral |
| NZ Policy | example | 106 | Neutral |
| NZ Policy | extend | 106 | Neutral |
| NZ Policy | outcome | 105 | Neutral |
| NZ Policy | small | 105 | Neutral |
| NZ Policy | access | 104 | Neutral |
| NZ Policy | king | 103 | Neutral |
| NZ Policy | operation | 103 | Neutral |
| NZ Policy | zone | 103 | Neutral |
| NZ Policy | subsection | 102 | Neutral |
| NZ Policy | terms | 100 | Neutral |
| NZ Policy | alternative | 99 | Neutral |
| NZ Policy | deliver | 99 | Neutral |
| NZ Policy | receive | 99 | Neutral |
| NZ Policy | term | 99 | Neutral |
| NZ Policy | control | 98 | Neutral |
| NZ Policy | intent | 98 | Neutral |
| NZ Policy | lwi | 98 | Neutral |
| NZ Policy | reach | 98 | Neutral |
| NZ Policy | together | 98 | Neutral |
| NZ Policy | avoid | 97 | Neutral |
| NZ Policy | position | 97 | Neutral |
| NZ Policy | recognise | 97 | Neutral |
| NZ Policy | become | 96 | Neutral |
| NZ Policy | central | 96 | Neutral |
| NZ Policy | collective | 96 | Neutral |
| NZ Policy | february | 96 | Neutral |
| NZ Policy | paragraph | 96 | Neutral |
| NZ Policy | box | 94 | Neutral |
| NZ Policy | island | 94 | Neutral |
| NZ Policy | process | 94 | Neutral |
| NZ Policy | assess | 93 | Neutral |
| NZ Policy | business | 93 | Neutral |
| NZ Policy | contain | 93 | Neutral |
| NZ Policy | hear | 93 | Neutral |
| NZ Policy | research | 93 | Neutral |
| NZ Policy | sector | 93 | Neutral |
| NZ Policy | transitional | 93 | Neutral |
| NZ Policy | similar | 92 | Neutral |
| NZ Policy | stand | 92 | Neutral |
| NZ Policy | retain | 91 | Neutral |
| NZ Policy | come | 90 | Neutral |
| NZ Policy | due | 90 | Neutral |
| NZ Policy | necessary | 90 | Neutral |
| NZ Policy | deemed | 89 | Neutral |
| NZ Policy | appendix | 88 | Neutral |
| NZ Policy | aspect | 88 | Neutral |
| NZ Policy | delete | 88 | Neutral |
| NZ Policy | either | 88 | Neutral |
| NZ Policy | friend | 88 | Neutral |
| NZ Policy | interim | 88 | Neutral |
| NZ Policy | catch | 87 | Neutral |
| NZ Policy | wish | 87 | Neutral |
| NZ Policy | different | 86 | Neutral |
| NZ Policy | less | 86 | Neutral |
| NZ Policy | organisation | 86 | Neutral |
| NZ Policy | granted | 85 | Neutral |
| NZ Policy | level | 85 | Neutral |
| NZ Policy | unless | 85 | Neutral |
| NZ Policy | regulations | 84 | Neutral |
| NZ Policy | relate | 84 | Neutral |
| NZ Policy | since | 84 | Neutral |
| NZ Policy | accept | 83 | Neutral |
| NZ Policy | association | 83 | Neutral |
| NZ Policy | long | 83 | Neutral |
| NZ Policy | major | 83 | Neutral |
| NZ Policy | parliament | 83 | Neutral |
| NZ Policy | principle | 83 | Neutral |
| NZ Policy | represent | 83 | Neutral |
| NZ Policy | role | 83 | Neutral |
| NZ Policy | strongly | 83 | Neutral |
| NZ Policy | wellton | 83 | Neutral |
| NZ Policy | appeals | 82 | Neutral |
| NZ Policy | finfish | 82 | Neutral |
| NZ Policy | focus | 82 | Neutral |
| NZ Policy | importance | 82 | Neutral |
| NZ Policy | manage | 82 | Neutral |
| NZ Policy | present | 82 | Neutral |
| NZ Policy | prohibited | 82 | Neutral |
| NZ Policy | seek | 82 | Neutral |
| NZ Policy | status | 82 | Neutral |
| NZ Policy | taken | 82 | Neutral |
| NZ Policy | pilchard | 81 | Neutral |
| NZ Policy | circumstance | 80 | Neutral |
| NZ Policy | occupation | 79 | Neutral |
| NZ Policy | port | 79 | Neutral |
| NZ Policy | reference | 79 | Neutral |
| NZ Policy | renewal | 79 | Neutral |
| NZ Policy | sea | 78 | Neutral |
| NZ Policy | undertaken | 78 | Neutral |
| NZ Policy | obtain | 77 | Neutral |
| NZ Policy | email | 76 | Neutral |
| NZ Policy | means | 76 | Neutral |
| NZ Policy | minimum | 76 | Neutral |
| NZ Policy | northland | 76 | Neutral |
| NZ Policy | notice | 76 | Neutral |
| NZ Policy | possible | 76 | Neutral |
| NZ Policy | previous | 76 | Neutral |
| NZ Policy | proceed | 76 | Neutral |
| NZ Policy | specified | 76 | Neutral |
| NZ Policy | system | 76 | Neutral |
| NZ Policy | basis | 75 | Neutral |
| NZ Policy | greater | 75 | Neutral |
| NZ Policy | grow | 75 | Neutral |
| NZ Policy | point | 75 | Neutral |
| NZ Policy | statutory | 75 | Neutral |
| NZ Policy | authorisation | 74 | Neutral |
| NZ Policy | eco | 74 | Neutral |
| NZ Policy | generally | 74 | Neutral |
| NZ Policy | industrial | 74 | Neutral |
| NZ Policy | reconsent | 74 | Neutral |
| NZ Policy | regulatory | 74 | Neutral |
| NZ Policy | values | 74 | Neutral |
| NZ Policy | clarify | 73 | Neutral |
| NZ Policy | private | 73 | Neutral |
| NZ Policy | fishery | 72 | Neutral |
| NZ Policy | notification | 72 | Neutral |
| NZ Policy | person | 72 | Neutral |
| NZ Policy | thames | 72 | Neutral |
| NZ Policy | agent | 71 | Neutral |
| NZ Policy | agr | 71 | Neutral |
| NZ Policy | criteria | 69 | Neutral |
| NZ Policy | fully | 69 | Neutral |
| NZ Policy | hauraki | 69 | Neutral |
| NZ Policy | scallop | 69 | Neutral |
| NZ Policy | threshold | 69 | Neutral |
| NZ Policy | cmfa | 68 | Neutral |
| NZ Policy | fact | 68 | Neutral |
| NZ Policy | fishers | 68 | Neutral |
| NZ Policy | individual | 68 | Neutral |
| NZ Policy | judicial | 68 | Neutral |
| NZ Policy | matter | 68 | Neutral |
| NZ Policy | participate | 68 | Neutral |
| NZ Policy | situation | 68 | Neutral |
| NZ Policy | balance | 67 | Neutral |
| NZ Policy | consequence | 67 | Neutral |
| NZ Policy | people | 67 | Neutral |
| NZ Policy | board | 66 | Neutral |
| NZ Policy | consult | 66 | Neutral |
| NZ Policy | ngati | 66 | Neutral |
| NZ Policy | action | 65 | Neutral |
| NZ Policy | deal | 65 | Neutral |
| NZ Policy | full | 65 | Neutral |
| NZ Policy | market | 65 | Neutral |
| NZ Policy | repeal | 65 | Neutral |
| NZ Policy | analysis | 64 | Neutral |
| NZ Policy | behalf | 64 | Neutral |
| NZ Policy | bird | 64 | Neutral |
| NZ Policy | far | 64 | Neutral |
| NZ Policy | least | 64 | Neutral |
| NZ Policy | operate | 64 | Neutral |
| NZ Policy | understand | 64 | Neutral |
| NZ Policy | make | 63 | Neutral |
| NZ Policy | society | 63 | Neutral |
| NZ Policy | sought | 63 | Neutral |
| NZ Policy | account | 62 | Neutral |
| NZ Policy | another | 62 | Neutral |
| NZ Policy | asset | 62 | Neutral |
| NZ Policy | complete | 62 | Neutral |
| NZ Policy | default | 62 | Neutral |
| NZ Policy | little | 62 | Neutral |
| NZ Policy | propose | 62 | Neutral |
| NZ Policy | definition | 61 | Neutral |
| NZ Policy | gazette | 61 | Neutral |
| NZ Policy | ltd | 61 | Neutral |
| NZ Policy | range | 61 | Neutral |
| NZ Policy | seek | 61 | Neutral |
| NZ Policy | share | 61 | Neutral |
| NZ Policy | depend | 60 | Neutral |
| NZ Policy | farmed | 60 | Neutral |
| NZ Policy | function | 60 | Neutral |
| NZ Policy | persons | 60 | Neutral |
| NZ Policy | sle | 60 | Neutral |
| NZ Policy | associated | 59 | Neutral |
| NZ Policy | cover | 59 | Neutral |
| NZ Policy | direct | 59 | Neutral |
| NZ Policy | final | 59 | Neutral |
| NZ Policy | nitrogen | 59 | Neutral |
| NZ Policy | statement | 59 | Neutral |
| NZ Policy | zealand | 59 | Neutral |
| NZ Policy | minister | 58 | Neutral |
| NZ Policy | month | 58 | Neutral |
| NZ Policy | outside | 58 | Neutral |
| NZ Policy | quality | 58 | Neutral |
| NZ Policy | certainty | 57 | Neutral |
| NZ Policy | contact | 57 | Neutral |
| NZ Policy | experience | 57 | Neutral |
| NZ Policy | fax | 57 | Neutral |
| NZ Policy | found | 57 | Neutral |
| NZ Policy | harbour | 57 | Neutral |
| NZ Policy | rationale | 57 | Neutral |
| NZ Policy | reasons | 57 | Neutral |
| NZ Policy | resolve | 57 | Neutral |
| NZ Policy | prohibition | 56 | Neutral |
| NZ Policy | sites | 56 | Neutral |
| NZ Policy | total | 56 | Neutral |
| NZ Policy | character | 55 | Neutral |
| NZ Policy | coast | 55 | Neutral |
| NZ Policy | directly | 55 | Neutral |
| NZ Policy | discretionary | 55 | Neutral |
| NZ Policy | discuss | 55 | Neutral |
| NZ Policy | expect | 55 | Neutral |
| NZ Policy | independent | 55 | Neutral |
| NZ Policy | manage | 55 | Neutral |
| NZ Policy | mfa | 55 | Neutral |
| NZ Policy | ministerial | 55 | Neutral |
| NZ Policy | often | 55 | Neutral |
| NZ Policy | permitted | 55 | Neutral |
| NZ Policy | processed | 55 | Neutral |
| NZ Policy | reason | 55 | Neutral |
| NZ Policy | reflect | 55 | Neutral |
| NZ Policy | submits | 55 | Neutral |
| NZ Policy | us | 55 | Neutral |
| NZ Policy | boat | 54 | Neutral |
| NZ Policy | customary | 54 | Neutral |
| NZ Policy | essential | 54 | Neutral |
| NZ Policy | measure | 54 | Neutral |
| NZ Policy | remain | 54 | Neutral |
| NZ Policy | situations | 54 | Neutral |
| NZ Policy | tcdc | 54 | Neutral |
| NZ Policy | appear | 53 | Neutral |
| NZ Policy | hear | 53 | Neutral |
| NZ Policy | mean | 53 | Neutral |
| NZ Policy | objective | 53 | Neutral |
| NZ Policy | officials | 53 | Neutral |
| NZ Policy | south | 53 | Neutral |
| NZ Policy | various | 53 | Neutral |
| NZ Policy | wishes | 53 | Neutral |
| NZ Policy | claims | 52 | Neutral |
| NZ Policy | decide | 52 | Neutral |
| NZ Policy | evidence | 52 | Neutral |
| NZ Policy | hectare | 52 | Neutral |
| NZ Policy | increase | 52 | Neutral |
| NZ Policy | lines | 52 | Neutral |
| NZ Policy | notified | 52 | Neutral |
| NZ Policy | offer | 52 | Neutral |
| NZ Policy | purpose | 52 | Neutral |
| NZ Policy | tonnes | 52 | Neutral |
| NZ Policy | commence | 51 | Neutral |
| NZ Policy | feed | 51 | Neutral |
| NZ Policy | form | 51 | Neutral |
| NZ Policy | hold | 51 | Neutral |
| NZ Policy | produce | 51 | Neutral |
| NZ Policy | put | 51 | Neutral |
| NZ Policy | regulation | 51 | Neutral |
| NZ Policy | summary | 51 | Neutral |
| NZ Policy | annual | 50 | Neutral |
| NZ Policy | common | 50 | Neutral |
| NZ Policy | feed | 50 | Neutral |
| NZ Policy | inc | 50 | Neutral |
| NZ Policy | incentive | 50 | Neutral |
| NZ Policy | mfp | 50 | Neutral |
| NZ Policy | might | 50 | Neutral |
| NZ Policy | model | 50 | Neutral |
| NZ Policy | responsibility | 50 | Neutral |
| NZ Policy | suggested | 50 | Neutral |
| NZ Policy | waters | 50 | Neutral |
| NZ Policy | whole | 50 | Neutral |
| NZ Policy | approximately | 49 | Neutral |
| NZ Policy | cumulative | 49 | Neutral |
| NZ Policy | dealt | 49 | Neutral |
| NZ Policy | ecological | 49 | Neutral |
| NZ Policy | etc | 49 | Neutral |
| NZ Policy | groups | 49 | Neutral |
| NZ Policy | intended | 49 | Neutral |
| NZ Policy | knowledge | 49 | Neutral |
| NZ Policy | manner | 49 | Neutral |
| NZ Policy | original | 49 | Neutral |
| NZ Policy | register | 49 | Neutral |
| NZ Policy | accord | 48 | Neutral |
| NZ Policy | actual | 48 | Neutral |
| NZ Policy | adopt | 48 | Neutral |
| NZ Policy | appoint | 48 | Neutral |
| NZ Policy | draft | 48 | Neutral |
| NZ Policy | every | 48 | Neutral |
| NZ Policy | facility | 48 | Neutral |
| NZ Policy | identified | 48 | Neutral |
| NZ Policy | overall | 48 | Neutral |
| NZ Policy | practice | 48 | Neutral |
| NZ Policy | prevent | 48 | Neutral |
| NZ Policy | shellfish | 48 | Neutral |
| NZ Policy | strategy | 48 | Neutral |
| NZ Policy | anticipated | 47 | Neutral |
| NZ Policy | authorise | 47 | Neutral |
| NZ Policy | cabinet | 47 | Neutral |
| NZ Policy | holder | 47 | Neutral |
| NZ Policy | needed | 47 | Neutral |
| NZ Policy | own | 47 | Neutral |
| NZ Policy | simply | 47 | Neutral |
| NZ Policy | submit | 47 | Neutral |
| NZ Policy | world | 47 | Neutral |
| NZ Policy | along | 46 | Neutral |
| NZ Policy | cma | 46 | Neutral |
| NZ Policy | proceed | 46 | Neutral |
| NZ Policy | raise | 46 | Neutral |
| NZ Policy | main | 45 | Neutral |
| NZ Policy | maintain | 45 | Neutral |
| NZ Policy | obligation | 45 | Neutral |
| NZ Policy | undertake | 45 | Neutral |
| NZ Policy | users | 45 | Neutral |
| NZ Policy | yet | 45 | Neutral |
| NZ Policy | build | 44 | Neutral |
| NZ Policy | consider | 44 | Neutral |
| NZ Policy | especially | 44 | Neutral |
| NZ Policy | governance | 44 | Neutral |
| NZ Policy | road | 44 | Neutral |
| NZ Policy | active | 43 | Neutral |
| NZ Policy | experimental | 43 | Neutral |
| NZ Policy | gore | 43 | Neutral |
| NZ Policy | january | 43 | Neutral |
| NZ Policy | mandatory | 43 | Neutral |
| NZ Policy | monitor | 43 | Neutral |
| NZ Policy | near | 43 | Neutral |
| NZ Policy | phytoplankton | 43 | Neutral |
| NZ Policy | priority | 43 | Neutral |
| NZ Policy | product | 43 | Neutral |
| NZ Policy | regions | 43 | Neutral |
| NZ Policy | representative | 43 | Neutral |
| NZ Policy | transaction | 43 | Neutral |
| NZ Policy | zooplankton | 43 | Neutral |
| NZ Policy | certain | 42 | Neutral |
| NZ Policy | context | 42 | Neutral |
| NZ Policy | diet | 42 | Neutral |
| NZ Policy | dolphin | 42 | Neutral |
| NZ Policy | forest | 42 | Neutral |
| NZ Policy | longer | 42 | Neutral |
| NZ Policy | low | 42 | Neutral |
| NZ Policy | obligation | 42 | Neutral |
| NZ Policy | panel | 42 | Neutral |
| NZ Policy | path | 42 | Neutral |
| NZ Policy | provisional | 42 | Neutral |
| NZ Policy | refer | 42 | Neutral |
| NZ Policy | release | 42 | Neutral |
| NZ Policy | reservation | 42 | Neutral |
| NZ Policy | arise | 41 | Neutral |
| NZ Policy | chang | 41 | Neutral |
| NZ Policy | compliance | 41 | Neutral |
| NZ Policy | condition | 41 | Neutral |
| NZ Policy | direction | 41 | Neutral |
| NZ Policy | draft | 41 | Neutral |
| NZ Policy | east | 41 | Neutral |
| NZ Policy | event | 41 | Neutral |
| NZ Policy | identify | 41 | Neutral |
| NZ Policy | input | 41 | Neutral |
| NZ Policy | participant | 41 | Neutral |
| NZ Policy | parts | 41 | Neutral |
| NZ Policy | pelorus | 41 | Neutral |
| NZ Policy | scenario | 41 | Neutral |
| NZ Policy | scope | 41 | Neutral |
| NZ Policy | share | 41 | Neutral |
| NZ Policy | submitted | 41 | Neutral |
| NZ Policy | technical | 41 | Neutral |
| NZ Policy | amount | 40 | Neutral |
| NZ Policy | gannet | 40 | Neutral |
| NZ Policy | material | 40 | Neutral |
| NZ Policy | other | 40 | Neutral |
| NZ Policy | override | 40 | Neutral |
| NZ Policy | oysters | 40 | Neutral |
| NZ Policy | plankton | 40 | Neutral |
| NZ Policy | publicly | 40 | Neutral |
| NZ Policy | seen | 40 | Neutral |
| NZ Policy | special | 40 | Neutral |
| NZ Policy | specifically | 40 | Neutral |
| NZ Policy | unit | 40 | Neutral |
| NZ Policy | web | 40 | Neutral |
| NZ Policy | although | 39 | Neutral |
| NZ Policy | done | 39 | Neutral |
| NZ Policy | explicit | 39 | Neutral |
| NZ Policy | forward | 39 | Neutral |
| NZ Policy | life | 39 | Neutral |
| NZ Policy | seabed | 39 | Neutral |
| NZ Policy | suspension | 39 | Neutral |
| NZ Policy | vote | 39 | Neutral |
| NZ Policy | admiralty | 38 | Neutral |
| NZ Policy | aware | 38 | Neutral |
| NZ Policy | exist | 38 | Neutral |
| NZ Policy | financial | 38 | Neutral |
| NZ Policy | implication | 38 | Neutral |
| NZ Policy | intention | 38 | Neutral |
| NZ Policy | mof | 38 | Neutral |
| NZ Policy | nature | 38 | Neutral |
| NZ Policy | notes | 38 | Neutral |
| NZ Policy | receipt | 38 | Neutral |
| NZ Policy | relationship | 38 | Neutral |
| NZ Policy | scale | 38 | Neutral |
| NZ Policy | seems | 38 | Neutral |
| NZ Policy | specify | 38 | Neutral |
| NZ Policy | structures | 38 | Neutral |
| NZ Policy | submitters | 38 | Neutral |
| NZ Policy | uncertainty | 38 | Neutral |
| NZ Policy | welcomes | 38 | Neutral |
| NZ Policy | assist | 37 | Neutral |
| NZ Policy | catch | 37 | Neutral |
| NZ Policy | effort | 37 | Neutral |
| NZ Policy | export | 37 | Neutral |
| NZ Policy | fed | 37 | Neutral |
| NZ Policy | hear | 37 | Neutral |
| NZ Policy | line | 37 | Neutral |
| NZ Policy | operative | 37 | Neutral |
| NZ Policy | paper | 37 | Neutral |
| NZ Policy | remains | 37 | Neutral |
| NZ Policy | seek | 37 | Neutral |
| NZ Policy | social | 37 | Neutral |
| NZ Policy | wider | 37 | Neutral |
| NZ Policy | across | 36 | Neutral |
| NZ Policy | aupouri | 36 | Neutral |
| NZ Policy | component | 36 | Neutral |
| NZ Policy | considerable | 36 | Neutral |
| NZ Policy | economy | 36 | Neutral |
| NZ Policy | end | 36 | Neutral |
| NZ Policy | exclusive | 36 | Neutral |
| NZ Policy | greenshell | 36 | Neutral |
| NZ Policy | help | 36 | Neutral |
| NZ Policy | implementation | 36 | Neutral |
| NZ Policy | leave | 36 | Neutral |
| NZ Policy | size | 36 | Neutral |
| NZ Policy | smw | 36 | Neutral |
| NZ Policy | suspend | 36 | Neutral |
| NZ Policy | appears | 35 | Neutral |
| NZ Policy | decisionmaking | 35 | Neutral |
| NZ Policy | doc | 35 | Neutral |
| NZ Policy | extensive | 35 | Neutral |
| NZ Policy | force | 35 | Neutral |
| NZ Policy | freshwater | 35 | Neutral |
| NZ Policy | integrity | 35 | Neutral |
| NZ Policy | objective | 35 | Neutral |
| NZ Policy | past | 35 | Neutral |
| NZ Policy | phone | 35 | Neutral |
| NZ Policy | prerequest | 35 | Neutral |
| NZ Policy | protected | 35 | Neutral |
| NZ Policy | second | 35 | Neutral |
| NZ Policy | secretariat | 35 | Neutral |
| NZ Policy | several | 35 | Neutral |
| NZ Policy | shorter | 35 | Neutral |
| NZ Policy | staff | 35 | Neutral |
| NZ Policy | standard | 35 | Neutral |
| NZ Policy | tourism | 35 | Neutral |
| NZ Policy | treated | 35 | Neutral |
| NZ Policy | trustee | 35 | Neutral |
| NZ Policy | upon | 35 | Neutral |
| NZ Policy | vital | 35 | Neutral |
| NZ Policy | acknowledge | 34 | Neutral |
| NZ Policy | bay | 34 | Neutral |
| NZ Policy | ecology | 34 | Neutral |
| NZ Policy | ecosystem | 34 | Neutral |
| NZ Policy | extent | 34 | Neutral |
| NZ Policy | factory | 34 | Neutral |
| NZ Policy | increase | 34 | Neutral |
| NZ Policy | response | 34 | Neutral |
| NZ Policy | return | 34 | Neutral |
| NZ Policy | separate | 34 | Neutral |
| NZ Policy | solutions | 34 | Neutral |
| NZ Policy | spac | 34 | Neutral |
| NZ Policy | stage | 34 | Neutral |
| NZ Policy | subsequent | 34 | Neutral |
| NZ Policy | tender | 34 | Neutral |
| NZ Policy | timeframe | 34 | Neutral |
| NZ Policy | wide | 34 | Neutral |
| NZ Policy | billion | 33 | Neutral |
| NZ Policy | consortium | 33 | Neutral |
| NZ Policy | equally | 33 | Neutral |
| NZ Policy | held | 33 | Neutral |
| NZ Policy | highly | 33 | Neutral |
| NZ Policy | hold | 33 | Neutral |
| NZ Policy | infrastructure | 33 | Neutral |
| NZ Policy | initial | 33 | Neutral |
| NZ Policy | known | 33 | Neutral |
| NZ Policy | notify | 33 | Neutral |
| NZ Policy | productivity | 33 | Neutral |
| NZ Policy | promote | 33 | Neutral |
| NZ Policy | responsible | 33 | Neutral |
| NZ Policy | sales | 33 | Neutral |
| NZ Policy | significantly | 33 | Neutral |
| NZ Policy | solution | 33 | Neutral |
| NZ Policy | standards | 33 | Neutral |
| NZ Policy | vote | 33 | Neutral |
| NZ Policy | drafted | 32 | Neutral |
| NZ Policy | going | 32 | Neutral |
| NZ Policy | incorporated | 32 | Neutral |
| NZ Policy | increased | 32 | Neutral |
| NZ Policy | industries | 32 | Neutral |
| NZ Policy | managed | 32 | Neutral |
| NZ Policy | otherwise | 32 | Neutral |
| NZ Policy | placed | 32 | Neutral |
| NZ Policy | please | 32 | Neutral |
| NZ Policy | question | 32 | Neutral |
| NZ Policy | recent | 32 | Neutral |
| NZ Policy | accordance | 31 | Neutral |
| NZ Policy | always | 31 | Neutral |
| NZ Policy | anchovy | 31 | Neutral |
| NZ Policy | andor | 31 | Neutral |
| NZ Policy | background | 31 | Neutral |
| NZ Policy | big | 31 | Neutral |
| NZ Policy | exclude | 31 | Neutral |
| NZ Policy | find | 31 | Neutral |
| NZ Policy | kingfish | 31 | Neutral |
| NZ Policy | lease | 31 | Neutral |
| NZ Policy | letter | 31 | Neutral |
| NZ Policy | mdc | 31 | Neutral |
| NZ Policy | negotiate | 31 | Neutral |
| NZ Policy | north | 31 | Neutral |
| NZ Policy | ongo | 31 | Neutral |
| NZ Policy | park | 31 | Neutral |
| NZ Policy | points | 31 | Neutral |
| NZ Policy | referred | 31 | Neutral |
| NZ Policy | sit | 31 | Neutral |
| NZ Policy | suggest | 31 | Neutral |
| NZ Policy | ynz | 31 | Neutral |
| NZ Policy | appealed | 30 | Neutral |
| NZ Policy | australia | 30 | Neutral |
| NZ Policy | blue | 30 | Neutral |
| NZ Policy | completely | 30 | Neutral |
| NZ Policy | fund | 30 | Neutral |
| NZ Policy | history | 30 | Neutral |
| NZ Policy | imposed | 30 | Neutral |
| NZ Policy | instead | 30 | Neutral |
| NZ Policy | intervention | 30 | Neutral |
| NZ Policy | islands | 30 | Neutral |
| NZ Policy | job | 30 | Neutral |
| NZ Policy | majority | 30 | Neutral |
| NZ Policy | march | 30 | Neutral |
| NZ Policy | moana | 30 | Neutral |
| NZ Policy | prepared | 30 | Neutral |
| NZ Policy | significance | 30 | Neutral |
| NZ Policy | spatial | 30 | Neutral |
| NZ Policy | state | 30 | Neutral |
| NZ Policy | turn | 30 | Neutral |
| NZ Policy | via | 30 | Neutral |
| NZ Policy | want | 30 | Neutral |
| NZ Policy | agency | 29 | Neutral |
| NZ Policy | beyond | 29 | Neutral |
| NZ Policy | conclude | 29 | Neutral |
| NZ Policy | huge | 29 | Neutral |
| NZ Policy | immediate | 29 | Neutral |
| NZ Policy | know | 29 | Neutral |
| NZ Policy | lease | 29 | Neutral |
| NZ Policy | property | 29 | Neutral |
| NZ Policy | read | 29 | Neutral |
| NZ Policy | sound | 29 | Neutral |
| NZ Policy | step | 29 | Neutral |
| NZ Policy | though | 29 | Neutral |
| NZ Policy | acceptable | 28 | Neutral |
| NZ Policy | accredited | 28 | Neutral |
| NZ Policy | atiawa | 28 | Neutral |
| NZ Policy | challenger | 28 | Neutral |
| NZ Policy | defined | 28 | Neutral |
| NZ Policy | factor | 28 | Neutral |
| NZ Policy | fish | 28 | Neutral |
| NZ Policy | fund | 28 | Neutral |
| NZ Policy | inshore | 28 | Neutral |
| NZ Policy | instance | 28 | Neutral |
| NZ Policy | justification | 28 | Neutral |
| NZ Policy | manage | 28 | Neutral |
| NZ Policy | never | 28 | Neutral |
| NZ Policy | perspective | 28 | Neutral |
| NZ Policy | pilchard | 28 | Neutral |
| NZ Policy | provis | 28 | Neutral |
| NZ Policy | sanford | 28 | Neutral |
| NZ Policy | school | 28 | Neutral |
| NZ Policy | smaller | 28 | Neutral |
| NZ Policy | source | 28 | Neutral |
| NZ Policy | take | 28 | Neutral |
| NZ Policy | uses | 28 | Neutral |
| NZ Policy | visual | 28 | Neutral |
| NZ Policy | written | 28 | Neutral |
| NZ Policy | adjacent | 27 | Neutral |
| NZ Policy | carried | 27 | Neutral |
| NZ Policy | conduct | 27 | Neutral |
| NZ Policy | december | 27 | Neutral |
| NZ Policy | discretion | 27 | Neutral |
| NZ Policy | enter | 27 | Neutral |
| NZ Policy | extremely | 27 | Neutral |
| NZ Policy | filter | 27 | Neutral |
| NZ Policy | five | 27 | Neutral |
| NZ Policy | formal | 27 | Neutral |
| NZ Policy | forum | 27 | Neutral |
| NZ Policy | four | 27 | Neutral |
| NZ Policy | july | 27 | Neutral |
| NZ Policy | location | 27 | Neutral |
| NZ Policy | lodged | 27 | Neutral |
| NZ Policy | mainly | 27 | Neutral |
| NZ Policy | minor | 27 | Neutral |
| NZ Policy | move | 27 | Neutral |
| NZ Policy | normal | 27 | Neutral |
| NZ Policy | obvious | 27 | Neutral |
| NZ Policy | plenty | 27 | Neutral |
| NZ Policy | protections | 27 | Neutral |
| NZ Policy | seafics | 27 | Neutral |
| NZ Policy | security | 27 | Neutral |
| NZ Policy | sheltered | 27 | Neutral |
| NZ Policy | shows | 27 | Neutral |
| NZ Policy | study | 27 | Neutral |
| NZ Policy | telephone | 27 | Neutral |
| NZ Policy | thus | 27 | Neutral |
| NZ Policy | treatment | 27 | Neutral |
| NZ Policy | type | 27 | Neutral |
| NZ Policy | word | 27 | Neutral |
| NZ Policy | contrary | 26 | Neutral |
| NZ Policy | cultural | 26 | Neutral |
| NZ Policy | except | 26 | Neutral |
| NZ Policy | higher | 26 | Neutral |
| NZ Policy | located | 26 | Neutral |
| NZ Policy | outlined | 26 | Neutral |
| NZ Policy | protect | 26 | Neutral |
| NZ Policy | real | 26 | Neutral |
| NZ Policy | shall | 26 | Neutral |
| NZ Policy | throughout | 26 | Neutral |
| NZ Policy | views | 26 | Neutral |
| NZ Policy | voluntary | 26 | Neutral |
| NZ Policy | western | 26 | Neutral |
| NZ Policy | zone | 26 | Neutral |
| NZ Policy | attempt | 25 | Neutral |
| NZ Policy | away | 25 | Neutral |
| NZ Policy | capital | 25 | Neutral |
| NZ Policy | charge | 25 | Neutral |
| NZ Policy | data | 25 | Neutral |
| NZ Policy | difference | 25 | Neutral |
| NZ Policy | haven | 25 | Neutral |
| NZ Policy | hectare | 25 | Neutral |
| NZ Policy | implement | 25 | Neutral |
| NZ Policy | indeed | 25 | Neutral |
| NZ Policy | issued | 25 | Neutral |
| NZ Policy | level | 25 | Neutral |
| NZ Policy | mean | 25 | Neutral |
| NZ Policy | nzcps | 25 | Neutral |
| NZ Policy | ocean | 25 | Neutral |
| NZ Policy | outer | 25 | Neutral |
| NZ Policy | pacific | 25 | Neutral |
| NZ Policy | party | 25 | Neutral |
| NZ Policy | propos | 25 | Neutral |
| NZ Policy | rush | 25 | Neutral |
| NZ Policy | served | 25 | Neutral |
| NZ Policy | shore | 25 | Neutral |
| NZ Policy | weight | 25 | Neutral |
| NZ Policy | adult | 24 | Neutral |
| NZ Policy | advice | 24 | Neutral |
| NZ Policy | animal | 24 | Neutral |
| NZ Policy | biodiversity | 24 | Neutral |
| NZ Policy | bring | 24 | Neutral |
| NZ Policy | carefully | 24 | Neutral |
| NZ Policy | certificate | 24 | Neutral |
| NZ Policy | choose | 24 | Neutral |
| NZ Policy | club | 24 | Neutral |
| NZ Policy | coastline | 24 | Neutral |
| NZ Policy | dear | 24 | Neutral |
| NZ Policy | department | 24 | Neutral |
| NZ Policy | fin | 24 | Neutral |
| NZ Policy | indigenous | 24 | Neutral |
| NZ Policy | invest | 24 | Neutral |
| NZ Policy | item | 24 | Neutral |
| NZ Policy | landbased | 24 | Neutral |
| NZ Policy | light | 24 | Neutral |
| NZ Policy | matt | 24 | Neutral |
| NZ Policy | meet | 24 | Neutral |
| NZ Policy | multiple | 24 | Neutral |
| NZ Policy | non | 24 | Neutral |
| NZ Policy | occupy | 24 | Neutral |
| NZ Policy | parallel | 24 | Neutral |
| NZ Policy | prospective | 24 | Neutral |
| NZ Policy | recently | 24 | Neutral |
| NZ Policy | sectors | 24 | Neutral |
| NZ Policy | sincer | 24 | Neutral |
| NZ Policy | streamline | 24 | Neutral |
| NZ Policy | treaty | 24 | Neutral |
| NZ Policy | widespread | 24 | Neutral |
| NZ Policy | advisory | 23 | Neutral |
| NZ Policy | boaties | 23 | Neutral |
| NZ Policy | boundary | 23 | Neutral |
| NZ Policy | challenge | 23 | Neutral |
| NZ Policy | commun | 23 | Neutral |
| NZ Policy | concept | 23 | Neutral |
| NZ Policy | constraints | 23 | Neutral |
| NZ Policy | copy | 23 | Neutral |
| NZ Policy | equivalent | 23 | Neutral |
| NZ Policy | fishermen | 23 | Neutral |
| NZ Policy | fundamental | 23 | Neutral |
| NZ Policy | gap | 23 | Neutral |
| NZ Policy | half | 23 | Neutral |
| NZ Policy | lapsing | 23 | Neutral |
| NZ Policy | later | 23 | Neutral |
| NZ Policy | litigation | 23 | Neutral |
| NZ Policy | money | 23 | Neutral |
| NZ Policy | office | 23 | Neutral |
| NZ Policy | old | 23 | Neutral |
| NZ Policy | organism | 23 | Neutral |
| NZ Policy | overlap | 23 | Neutral |
| NZ Policy | peninsula | 23 | Neutral |
| NZ Policy | prey | 23 | Neutral |
| NZ Policy | procedure | 23 | Neutral |
| NZ Policy | promote | 23 | Neutral |
| NZ Policy | rao | 23 | Neutral |
| NZ Policy | replacement | 23 | Neutral |
| NZ Policy | safeguards | 23 | Neutral |
| NZ Policy | settl | 23 | Neutral |
| NZ Policy | short | 23 | Neutral |
| NZ Policy | shown | 23 | Neutral |
| NZ Policy | speak | 23 | Neutral |
| NZ Policy | stakeholders | 23 | Neutral |
| NZ Policy | tama | 23 | Neutral |
| NZ Policy | tau | 23 | Neutral |
| NZ Policy | whose | 23 | Neutral |
| NZ Policy | almost | 22 | Neutral |
| NZ Policy | among | 22 | Neutral |
| NZ Policy | april | 22 | Neutral |
| NZ Policy | baitfish | 22 | Neutral |
| NZ Policy | cause | 22 | Neutral |
| NZ Policy | chlorophyll | 22 | Neutral |
| NZ Policy | column | 22 | Neutral |
| NZ Policy | councillor | 22 | Neutral |
| NZ Policy | course | 22 | Neutral |
| NZ Policy | deal | 22 | Neutral |
| NZ Policy | degree | 22 | Neutral |
| NZ Policy | detritus | 22 | Neutral |
| NZ Policy | director | 22 | Neutral |
| NZ Policy | duration | 22 | Neutral |
| NZ Policy | eggs | 22 | Neutral |
| NZ Policy | exercise | 22 | Neutral |
| NZ Policy | existence | 22 | Neutral |
| NZ Policy | figure | 22 | Neutral |
| NZ Policy | grant | 22 | Neutral |
| NZ Policy | grounds | 22 | Neutral |
| NZ Policy | leadership | 22 | Neutral |
| NZ Policy | list | 22 | Neutral |
| NZ Policy | materially | 22 | Neutral |
| NZ Policy | minority | 22 | Neutral |
| NZ Policy | normalisation | 22 | Neutral |
| NZ Policy | numerous | 22 | Neutral |
| NZ Policy | offsite | 22 | Neutral |
| NZ Policy | package | 22 | Neutral |
| NZ Policy | possibly | 22 | Neutral |
| NZ Policy | practical | 22 | Neutral |
| NZ Policy | products | 22 | Neutral |
| NZ Policy | quite | 22 | Neutral |
| NZ Policy | refers | 22 | Neutral |
| NZ Policy | relatively | 22 | Neutral |
| NZ Policy | revenue | 22 | Neutral |
| NZ Policy | sardinops | 22 | Neutral |
| NZ Policy | scientific | 22 | Neutral |
| NZ Policy | season | 22 | Neutral |
| NZ Policy | set | 22 | Neutral |
| NZ Policy | soon | 22 | Neutral |
| NZ Policy | stages | 22 | Neutral |
| NZ Policy | statements | 22 | Neutral |
| NZ Policy | states | 22 | Neutral |
| NZ Policy | street | 22 | Neutral |
| NZ Policy | submitter | 22 | Neutral |
| NZ Policy | take | 22 | Neutral |
| NZ Policy | tools | 22 | Neutral |
| NZ Policy | aim | 21 | Neutral |
| NZ Policy | canterbury | 21 | Neutral |
| NZ Policy | consideration | 21 | Neutral |
| NZ Policy | deadline | 21 | Neutral |
| NZ Policy | desire | 21 | Neutral |
| NZ Policy | diagram | 21 | Neutral |
| NZ Policy | equal | 21 | Neutral |
| NZ Policy | goal | 21 | Neutral |
| NZ Policy | grant | 21 | Neutral |
| NZ Policy | habitat | 21 | Neutral |
| NZ Policy | immediately | 21 | Neutral |
| NZ Policy | influence | 21 | Neutral |
| NZ Policy | interface | 21 | Neutral |
| NZ Policy | left | 21 | Neutral |
| NZ Policy | lower | 21 | Neutral |
| NZ Policy | maf | 21 | Neutral |
| NZ Policy | merits | 21 | Neutral |
| NZ Policy | met | 21 | Neutral |
| NZ Policy | navigation | 21 | Neutral |
| NZ Policy | november | 21 | Neutral |
| NZ Policy | opinion | 21 | Neutral |
| NZ Policy | pathway | 21 | Neutral |
| NZ Policy | populations | 21 | Neutral |
| NZ Policy | precedent | 21 | Neutral |
| NZ Policy | rate | 21 | Neutral |
| NZ Policy | reverse | 21 | Neutral |
| NZ Policy | sets | 21 | Neutral |
| NZ Policy | snapper | 21 | Neutral |
| NZ Policy | stand | 21 | Neutral |
| NZ Policy | sure | 21 | Neutral |
| NZ Policy | think | 21 | Neutral |
| NZ Policy | try | 21 | Neutral |
| NZ Policy | try | 21 | Neutral |
| NZ Policy | waka | 21 | Neutral |
| NZ Policy | whereas | 21 | Neutral |
| NZ Policy | actually | 20 | Neutral |
| NZ Policy | assumption | 20 | Neutral |
| NZ Policy | attribute | 20 | Neutral |
| NZ Policy | capacity | 20 | Neutral |
| NZ Policy | centre | 20 | Neutral |
| NZ Policy | cod | 20 | Neutral |
| NZ Policy | dont | 20 | Neutral |
| NZ Policy | elected | 20 | Neutral |
| NZ Policy | evaluation | 20 | Neutral |
| NZ Policy | expiry | 20 | Neutral |
| NZ Policy | intensity | 20 | Neutral |
| NZ Policy | international | 20 | Neutral |
| NZ Policy | joint | 20 | Neutral |
| NZ Policy | kaimoana | 20 | Neutral |
| NZ Policy | look | 20 | Neutral |
| NZ Policy | ona | 20 | Neutral |
| NZ Policy | overseas | 20 | Neutral |
| NZ Policy | ownership | 20 | Neutral |
| NZ Policy | ring | 20 | Neutral |
| NZ Policy | sealord | 20 | Neutral |
| NZ Policy | shareholder | 20 | Neutral |
| NZ Policy | streamline | 20 | Neutral |
| NZ Policy | suggests | 20 | Neutral |
| NZ Policy | table | 20 | Neutral |
| NZ Policy | technology | 20 | Neutral |
| NZ Policy | ultimately | 20 | Neutral |
| NZ Policy | website | 20 | Neutral |
| NZ Policy | acre | 19 | Neutral |
| NZ Policy | administrative | 19 | Neutral |
| NZ Policy | app | 19 | Neutral |
| NZ Policy | barrier | 19 | Neutral |
| NZ Policy | caught | 19 | Neutral |
| NZ Policy | cmz | 19 | Neutral |
| NZ Policy | contribute | 19 | Neutral |
| NZ Policy | exercised | 19 | Neutral |
| NZ Policy | expertise | 19 | Neutral |
| NZ Policy | forms | 19 | Neutral |
| NZ Policy | goes | 19 | Neutral |
| NZ Policy | industry | 19 | Neutral |
| NZ Policy | inquiry | 19 | Neutral |
| NZ Policy | integration | 19 | Neutral |
| NZ Policy | kuku | 19 | Neutral |
| NZ Policy | larvae | 19 | Neutral |
| NZ Policy | manager | 19 | Neutral |
| NZ Policy | mandated | 19 | Neutral |
| NZ Policy | markets | 19 | Neutral |
| NZ Policy | numbers | 19 | Neutral |
| NZ Policy | offshore | 19 | Neutral |
| NZ Policy | project | 19 | Neutral |
| NZ Policy | scrutiny | 19 | Neutral |
| NZ Policy | sense | 19 | Neutral |
| NZ Policy | stated | 19 | Neutral |
| NZ Policy | stewart | 19 | Neutral |
| NZ Policy | structure | 19 | Neutral |
| NZ Policy | subsequently | 19 | Neutral |
| NZ Policy | substantial | 19 | Neutral |
| NZ Policy | summari | 19 | Neutral |
| NZ Policy | system | 19 | Neutral |
| NZ Policy | timing | 19 | Neutral |
| NZ Policy | toka | 19 | Neutral |
| NZ Policy | transport | 19 | Neutral |
| NZ Policy | treat | 19 | Neutral |
| NZ Policy | trigger | 19 | Neutral |
| NZ Policy | whangarei | 19 | Neutral |
| NZ Policy | whenua | 19 | Neutral |
| NZ Policy | accreditation | 18 | Neutral |
| NZ Policy | apart | 18 | Neutral |
| NZ Policy | argument | 18 | Neutral |
| NZ Policy | attention | 18 | Neutral |
| NZ Policy | benthic | 18 | Neutral |
| NZ Policy | biosecurity | 18 | Neutral |
| NZ Policy | brown | 18 | Neutral |
| NZ Policy | chairman | 18 | Neutral |
| NZ Policy | closed | 18 | Neutral |
| NZ Policy | compensation | 18 | Neutral |
| NZ Policy | content | 18 | Neutral |
| NZ Policy | crucial | 18 | Neutral |
| NZ Policy | currents | 18 | Neutral |
| NZ Policy | davenport | 18 | Neutral |
| NZ Policy | despite | 18 | Neutral |
| NZ Policy | disease | 18 | Neutral |
| NZ Policy | dissent | 18 | Neutral |
| NZ Policy | elsewhere | 18 | Neutral |
| NZ Policy | entire | 18 | Neutral |
| NZ Policy | entirely | 18 | Neutral |
| NZ Policy | feb | 18 | Neutral |
| NZ Policy | flow | 18 | Neutral |
| NZ Policy | govern | 18 | Neutral |
| NZ Policy | maximum | 18 | Neutral |
| NZ Policy | minimal | 18 | Neutral |
| NZ Policy | plants | 18 | Neutral |
| NZ Policy | resident | 18 | Neutral |
| NZ Policy | resolution | 18 | Neutral |
| NZ Policy | sanctuaries | 18 | Neutral |
| NZ Policy | service | 18 | Neutral |
| NZ Policy | settlement | 18 | Neutral |
| NZ Policy | shell | 18 | Neutral |
| NZ Policy | simple | 18 | Neutral |
| NZ Policy | sprat | 18 | Neutral |
| NZ Policy | start | 18 | Neutral |
| NZ Policy | subpart | 18 | Neutral |
| NZ Policy | taylor | 18 | Neutral |
| NZ Policy | tenure | 18 | Neutral |
| NZ Policy | attached | 17 | Neutral |
| NZ Policy | base | 17 | Neutral |
| NZ Policy | behind | 17 | Neutral |
| NZ Policy | bind | 17 | Neutral |
| NZ Policy | compared | 17 | Neutral |
| NZ Policy | comparison | 17 | Neutral |
| NZ Policy | compulsory | 17 | Neutral |
| NZ Policy | consequently | 17 | Neutral |
| NZ Policy | designed | 17 | Neutral |
| NZ Policy | enacted | 17 | Neutral |
| NZ Policy | essentially | 17 | Neutral |
| NZ Policy | expert | 17 | Neutral |
| NZ Policy | extract | 17 | Neutral |
| NZ Policy | holds | 17 | Neutral |
| NZ Policy | inclusion | 17 | Neutral |
| NZ Policy | internet | 17 | Neutral |
| NZ Policy | licence | 17 | Neutral |
| NZ Policy | likelihood | 17 | Neutral |
| NZ Policy | locations | 17 | Neutral |
| NZ Policy | nationally | 17 | Neutral |
| NZ Policy | neither | 17 | Neutral |
| NZ Policy | otago | 17 | Neutral |
| NZ Policy | owned | 17 | Neutral |
| NZ Policy | penguins | 17 | Neutral |
| NZ Policy | precautionary | 17 | Neutral |
| NZ Policy | prepare | 17 | Neutral |
| NZ Policy | presented | 17 | Neutral |
| NZ Policy | prove | 17 | Neutral |
| NZ Policy | relative | 17 | Neutral |
| NZ Policy | respond | 17 | Neutral |
| NZ Policy | safety | 17 | Neutral |
| NZ Policy | seas | 17 | Neutral |
| NZ Policy | sensitivity | 17 | Neutral |
| NZ Policy | spent | 17 | Neutral |
| NZ Policy | steffan | 17 | Neutral |
| NZ Policy | target | 17 | Neutral |
| NZ Policy | toward | 17 | Neutral |
| NZ Policy | unduly | 17 | Neutral |
| NZ Policy | university | 17 | Neutral |
| NZ Policy | west | 17 | Neutral |
| NZ Policy | yacht | 17 | Neutral |
| NZ Policy | acts | 16 | Neutral |
| NZ Policy | agriculture | 16 | Neutral |
| NZ Policy | aquacultures | 16 | Neutral |
| NZ Policy | australasian | 16 | Neutral |
| NZ Policy | behaviour | 16 | Neutral |
| NZ Policy | biology | 16 | Neutral |
| NZ Policy | body | 16 | Neutral |
| NZ Policy | built | 16 | Neutral |
| NZ Policy | busi | 16 | Neutral |
| NZ Policy | called | 16 | Neutral |
| NZ Policy | chain | 16 | Neutral |
| NZ Policy | closely | 16 | Neutral |
| NZ Policy | commissioners | 16 | Neutral |
| NZ Policy | constituent | 16 | Neutral |
| NZ Policy | correspond | 16 | Neutral |
| NZ Policy | debate | 16 | Neutral |
| NZ Policy | described | 16 | Neutral |
| NZ Policy | designated | 16 | Neutral |
| NZ Policy | dollar | 16 | Neutral |
| NZ Policy | examples | 16 | Neutral |
| NZ Policy | express | 16 | Neutral |
| NZ Policy | green | 16 | Neutral |
| NZ Policy | grown | 16 | Neutral |
| NZ Policy | head | 16 | Neutral |
| NZ Policy | hundreds | 16 | Neutral |
| NZ Policy | interested | 16 | Neutral |
| NZ Policy | kai | 16 | Neutral |
| NZ Policy | largely | 16 | Neutral |
| NZ Policy | legitimate | 16 | Neutral |
| NZ Policy | manawhenua | 16 | Neutral |
| NZ Policy | mara | 16 | Neutral |
| NZ Policy | opotiki | 16 | Neutral |
| NZ Policy | outcom | 16 | Neutral |
| NZ Policy | paid | 16 | Neutral |
| NZ Policy | pay | 16 | Neutral |
| NZ Policy | plant | 16 | Neutral |
| NZ Policy | possibility | 16 | Neutral |
| NZ Policy | post | 16 | Neutral |
| NZ Policy | pre | 16 | Neutral |
| NZ Policy | probably | 16 | Neutral |
| NZ Policy | queen | 16 | Neutral |
| NZ Policy | rates | 16 | Neutral |
| NZ Policy | rest | 16 | Neutral |
| NZ Policy | run | 16 | Neutral |
| NZ Policy | seem | 16 | Neutral |
| NZ Policy | sequence | 16 | Neutral |
| NZ Policy | shags | 16 | Neutral |
| NZ Policy | side | 16 | Neutral |
| NZ Policy | similarly | 16 | Neutral |
| NZ Policy | six | 16 | Neutral |
| NZ Policy | togeth | 16 | Neutral |
| NZ Policy | advance | 15 | Neutral |
| NZ Policy | alter | 15 | Neutral |
| NZ Policy | biomarine | 15 | Neutral |
| NZ Policy | blanket | 15 | Neutral |
| NZ Policy | bottom | 15 | Neutral |
| NZ Policy | breed | 15 | Neutral |
| NZ Policy | cage | 15 | Neutral |
| NZ Policy | carry | 15 | Neutral |
| NZ Policy | characteristics | 15 | Neutral |
| NZ Policy | chosen | 15 | Neutral |
| NZ Policy | cmzl | 15 | Neutral |
| NZ Policy | convert | 15 | Neutral |
| NZ Policy | diversity | 15 | Neutral |
| NZ Policy | eds | 15 | Neutral |
| NZ Policy | elements | 15 | Neutral |
| NZ Policy | etal | 15 | Neutral |
| NZ Policy | exception | 15 | Neutral |
| NZ Policy | feeders | 15 | Neutral |
| NZ Policy | foreshore | 15 | Neutral |
| NZ Policy | greenshelltm | 15 | Neutral |
| NZ Policy | happen | 15 | Neutral |
| NZ Policy | harbours | 15 | Neutral |
| NZ Policy | highest | 15 | Neutral |
| NZ Policy | identify | 15 | Neutral |
| NZ Policy | indicated | 15 | Neutral |
| NZ Policy | kuri | 15 | Neutral |
| NZ Policy | lhu | 15 | Neutral |
| NZ Policy | longlines | 15 | Neutral |
| NZ Policy | market | 15 | Neutral |
| NZ Policy | maui | 15 | Neutral |
| NZ Policy | msrmp | 15 | Neutral |
| NZ Policy | narrow | 15 | Neutral |
| NZ Policy | noth | 15 | Neutral |
| NZ Policy | october | 15 | Neutral |
| NZ Policy | originally | 15 | Neutral |
| NZ Policy | percentage | 15 | Neutral |
| NZ Policy | perhaps | 15 | Neutral |
| NZ Policy | physical | 15 | Neutral |
| NZ Policy | places | 15 | Neutral |
| NZ Policy | qma | 15 | Neutral |
| NZ Policy | recorded | 15 | Neutral |
| NZ Policy | renew | 15 | Neutral |
| NZ Policy | smith | 15 | Neutral |
| NZ Policy | spread | 15 | Neutral |
| NZ Policy | stakeholder | 15 | Neutral |
| NZ Policy | surface | 15 | Neutral |
| NZ Policy | thresholds | 15 | Neutral |
| NZ Policy | tidal | 15 | Neutral |
| NZ Policy | tourist | 15 | Neutral |
| NZ Policy | visitors | 15 | Neutral |
| NZ Policy | westpac | 15 | Neutral |
| NZ Policy | whereby | 15 | Neutral |
| NZ Policy | alone | 14 | Neutral |
| NZ Policy | appropri | 14 | Neutral |
| NZ Policy | asked | 14 | Neutral |
| NZ Policy | challenges | 14 | Neutral |
| NZ Policy | choice | 14 | Neutral |
| NZ Policy | combined | 14 | Neutral |
| NZ Policy | dual | 14 | Neutral |
| NZ Policy | dusky | 14 | Neutral |
| NZ Policy | face | 14 | Neutral |
| NZ Policy | fit | 14 | Neutral |
| NZ Policy | forsyth | 14 | Neutral |
| NZ Policy | historical | 14 | Neutral |
| NZ Policy | implemented | 14 | Neutral |
| NZ Policy | informed | 14 | Neutral |
| NZ Policy | inserts | 14 | Neutral |
| NZ Policy | intervene | 14 | Neutral |
| NZ Policy | justice | 14 | Neutral |
| NZ Policy | let | 14 | Neutral |
| NZ Policy | maker | 14 | Neutral |
| NZ Policy | mitigate | 14 | Neutral |
| NZ Policy | neighbour | 14 | Neutral |
| NZ Policy | northern | 14 | Neutral |
| NZ Policy | nursery | 14 | Neutral |
| NZ Policy | ootc | 14 | Neutral |
| NZ Policy | paul | 14 | Neutral |
| NZ Policy | penguin | 14 | Neutral |
| NZ Policy | planned | 14 | Neutral |
| NZ Policy | political | 14 | Neutral |
| NZ Policy | posit | 14 | Neutral |
| NZ Policy | pressure | 14 | Neutral |
| NZ Policy | prime | 14 | Neutral |
| NZ Policy | protocol | 14 | Neutral |
| NZ Policy | purification | 14 | Neutral |
| NZ Policy | quickly | 14 | Neutral |
| NZ Policy | recreation | 14 | Neutral |
| NZ Policy | replace | 14 | Neutral |
| NZ Policy | rmla | 14 | Neutral |
| NZ Policy | rohe | 14 | Neutral |
| NZ Policy | route | 14 | Neutral |
| NZ Policy | science | 14 | Neutral |
| NZ Policy | seabirds | 14 | Neutral |
| NZ Policy | seafoods | 14 | Neutral |
| NZ Policy | seed | 14 | Neutral |
| NZ Policy | signed | 14 | Neutral |
| NZ Policy | specie | 14 | Neutral |
| NZ Policy | struik | 14 | Neutral |
| NZ Policy | transition | 14 | Neutral |
| NZ Policy | understands | 14 | Neutral |
| NZ Policy | unintended | 14 | Neutral |
| NZ Policy | urge | 14 | Neutral |
| NZ Policy | valid | 14 | Neutral |
| NZ Policy | vessels | 14 | Neutral |
| NZ Policy | viability | 14 | Neutral |
| NZ Policy | waitangi | 14 | Neutral |
| NZ Policy | wat | 14 | Neutral |
| NZ Policy | whatua | 14 | Neutral |
| NZ Policy | accord | 13 | Neutral |
| NZ Policy | act | 13 | Neutral |
| NZ Policy | apparent | 13 | Neutral |
| NZ Policy | ara | 13 | Neutral |
| NZ Policy | arbitration | 13 | Neutral |
| NZ Policy | assistance | 13 | Neutral |
| NZ Policy | became | 13 | Neutral |
| NZ Policy | cease | 13 | Neutral |
| NZ Policy | charlotte | 13 | Neutral |
| NZ Policy | china | 13 | Neutral |
| NZ Policy | cut | 13 | Neutral |
| NZ Policy | dated | 13 | Neutral |
| NZ Policy | depth | 13 | Neutral |
| NZ Policy | employment | 13 | Neutral |
| NZ Policy | fishstocks | 13 | Neutral |
| NZ Policy | flutter | 13 | Neutral |
| NZ Policy | formed | 13 | Neutral |
| NZ Policy | furthermore | 13 | Neutral |
| NZ Policy | global | 13 | Neutral |
| NZ Policy | guidelines | 13 | Neutral |
| NZ Policy | health | 13 | Neutral |
| NZ Policy | heavily | 13 | Neutral |
| NZ Policy | incorporation | 13 | Neutral |
| NZ Policy | indicative | 13 | Neutral |
| NZ Policy | integrate | 13 | Neutral |
| NZ Policy | irrespective | 13 | Neutral |
| NZ Policy | landscape | 13 | Neutral |
| NZ Policy | mitigate | 13 | Neutral |
| NZ Policy | noncommercial | 13 | Neutral |
| NZ Policy | perna | 13 | Neutral |
| NZ Policy | peter | 13 | Neutral |
| NZ Policy | principal | 13 | Neutral |
| NZ Policy | rcp | 13 | Neutral |
| NZ Policy | reported | 13 | Neutral |
| NZ Policy | seals | 13 | Neutral |
| NZ Policy | secretary | 13 | Neutral |
| NZ Policy | stat | 13 | Neutral |
| NZ Policy | studies | 13 | Neutral |
| NZ Policy | thamescoromandel | 13 | Neutral |
| NZ Policy | tide | 13 | Neutral |
| NZ Policy | took | 13 | Neutral |
| NZ Policy | tool | 13 | Neutral |
| NZ Policy | totally | 13 | Neutral |
| NZ Policy | unchanged | 13 | Neutral |
| NZ Policy | utilisation | 13 | Neutral |
| NZ Policy | variation | 13 | Neutral |
| NZ Policy | white | 13 | Neutral |
| NZ Policy | wish | 13 | Neutral |
| NZ Policy | acquisition | 12 | Neutral |
| NZ Policy | anchorage | 12 | Neutral |
| NZ Policy | anyone | 12 | Neutral |
| NZ Policy | aquatic | 12 | Neutral |
| NZ Policy | arrangements | 12 | Neutral |
| NZ Policy | beginn | 12 | Neutral |
| NZ Policy | biological | 12 | Neutral |
| NZ Policy | bluff | 12 | Neutral |
| NZ Policy | boundaries | 12 | Neutral |
| NZ Policy | brought | 12 | Neutral |
| NZ Policy | classes | 12 | Neutral |
| NZ Policy | collectively | 12 | Neutral |
| NZ Policy | compromise | 12 | Neutral |
| NZ Policy | counsel | 12 | Neutral |
| NZ Policy | cruis | 12 | Neutral |
| NZ Policy | decade | 12 | Neutral |
| NZ Policy | deep | 12 | Neutral |
| NZ Policy | defer | 12 | Neutral |
| NZ Policy | ecosystems | 12 | Neutral |
| NZ Policy | emphasis | 12 | Neutral |
| NZ Policy | endur | 12 | Neutral |
| NZ Policy | entitlements | 12 | Neutral |
| NZ Policy | eudyptula | 12 | Neutral |
| NZ Policy | expires | 12 | Neutral |
| NZ Policy | expressed | 12 | Neutral |
| NZ Policy | families | 12 | Neutral |
| NZ Policy | feel | 12 | Neutral |
| NZ Policy | gener | 12 | Neutral |
| NZ Policy | guide | 12 | Neutral |
| NZ Policy | hon | 12 | Neutral |
| NZ Policy | imperative | 12 | Neutral |
| NZ Policy | inlet | 12 | Neutral |
| NZ Policy | internal | 12 | Neutral |
| NZ Policy | invertebrates | 12 | Neutral |
| NZ Policy | june | 12 | Neutral |
| NZ Policy | justified | 12 | Neutral |
| NZ Policy | kahawai | 12 | Neutral |
| NZ Policy | lodg | 12 | Neutral |
| NZ Policy | mammals | 12 | Neutral |
| NZ Policy | metres | 12 | Neutral |
| NZ Policy | midbay | 12 | Neutral |
| NZ Policy | mitigation | 12 | Neutral |
| NZ Policy | neopilchardus | 12 | Neutral |
| NZ Policy | none | 12 | Neutral |
| NZ Policy | nonstatutory | 12 | Neutral |
| NZ Policy | ofthe | 12 | Neutral |
| NZ Policy | oper | 12 | Neutral |
| NZ Policy | overly | 12 | Neutral |
| NZ Policy | pacifica | 12 | Neutral |
| NZ Policy | partnership | 12 | Neutral |
| NZ Policy | plays | 12 | Neutral |
| NZ Policy | pow | 12 | Neutral |
| NZ Policy | preparation | 12 | Neutral |
| NZ Policy | presently | 12 | Neutral |
| NZ Policy | protocols | 12 | Neutral |
| NZ Policy | pursuant | 12 | Neutral |
| NZ Policy | rock | 12 | Neutral |
| NZ Policy | sediment | 12 | Neutral |
| NZ Policy | sent | 12 | Neutral |
| NZ Policy | september | 12 | Neutral |
| NZ Policy | services | 12 | Neutral |
| NZ Policy | shag | 12 | Neutral |
| NZ Policy | shared | 12 | Neutral |
| NZ Policy | something | 12 | Neutral |
| NZ Policy | southern | 12 | Neutral |
| NZ Policy | spotted | 12 | Neutral |
| NZ Policy | summarised | 12 | Neutral |
| NZ Policy | summer | 12 | Neutral |
| NZ Policy | supplementary | 12 | Neutral |
| NZ Policy | supply | 12 | Neutral |
| NZ Policy | tangata | 12 | Neutral |
| NZ Policy | traditional | 12 | Neutral |
| NZ Policy | unlike | 12 | Neutral |
| NZ Policy | viable | 12 | Neutral |
| NZ Policy | yacht | 12 | Neutral |
| NZ Policy | zealanders | 12 | Neutral |
| NZ Policy | ala | 11 | Neutral |
| NZ Policy | anticompetitive | 11 | Neutral |
| NZ Policy | aside | 11 | Neutral |
| NZ Policy | autonomy | 11 | Neutral |
| NZ Policy | avail | 11 | Neutral |
| NZ Policy | bag | 11 | Neutral |
| NZ Policy | baker | 11 | Neutral |
| NZ Policy | build | 11 | Neutral |
| NZ Policy | callin | 11 | Neutral |
| NZ Policy | capture | 11 | Neutral |
| NZ Policy | chance | 11 | Neutral |
| NZ Policy | checks | 11 | Neutral |
| NZ Policy | consequential | 11 | Neutral |
| NZ Policy | contrast | 11 | Neutral |
| NZ Policy | culture | 11 | Neutral |
| NZ Policy | declared | 11 | Neutral |
| NZ Policy | deed | 11 | Neutral |
| NZ Policy | derived | 11 | Neutral |
| NZ Policy | distribution | 11 | Neutral |
| NZ Policy | eastern | 11 | Neutral |
| NZ Policy | eventually | 11 | Neutral |
| NZ Policy | ever | 11 | Neutral |
| NZ Policy | everything | 11 | Neutral |
| NZ Policy | expense | 11 | Neutral |
| NZ Policy | exposed | 11 | Neutral |
| NZ Policy | feature | 11 | Neutral |
| NZ Policy | finally | 11 | Neutral |
| NZ Policy | fishmeal | 11 | Neutral |
| NZ Policy | forfeiture | 11 | Neutral |
| NZ Policy | get | 11 | Neutral |
| NZ Policy | gibbs | 11 | Neutral |
| NZ Policy | happens | 11 | Neutral |
| NZ Policy | hapu | 11 | Neutral |
| NZ Policy | heatley | 11 | Neutral |
| NZ Policy | horse | 11 | Neutral |
| NZ Policy | human | 11 | Neutral |
| NZ Policy | implies | 11 | Neutral |
| NZ Policy | indirectly | 11 | Neutral |
| NZ Policy | inner | 11 | Neutral |
| NZ Policy | insert | 11 | Neutral |
| NZ Policy | interpretation | 11 | Neutral |
| NZ Policy | investors | 11 | Neutral |
| NZ Policy | keep | 11 | Neutral |
| NZ Policy | kept | 11 | Neutral |
| NZ Policy | listed | 11 | Neutral |
| NZ Policy | lodge | 11 | Neutral |
| NZ Policy | looks | 11 | Neutral |
| NZ Policy | mann | 11 | Neutral |
| NZ Policy | membership | 11 | Neutral |
| NZ Policy | minimise | 11 | Neutral |
| NZ Policy | move | 11 | Neutral |
| NZ Policy | muriwhenua | 11 | Neutral |
| NZ Policy | mytilus | 11 | Neutral |
| NZ Policy | native | 11 | Neutral |
| NZ Policy | nearly | 11 | Neutral |
| NZ Policy | nutrient | 11 | Neutral |
| NZ Policy | nzenvc | 11 | Neutral |
| NZ Policy | organis | 11 | Neutral |
| NZ Policy | parengarenga | 11 | Neutral |
| NZ Policy | pass | 11 | Neutral |
| NZ Policy | pay | 11 | Neutral |
| NZ Policy | pend | 11 | Neutral |
| NZ Policy | postal | 11 | Neutral |
| NZ Policy | price | 11 | Neutral |
| NZ Policy | qms | 11 | Neutral |
| NZ Policy | ratified | 11 | Neutral |
| NZ Policy | repeat | 11 | Neutral |
| NZ Policy | retention | 11 | Neutral |
| NZ Policy | roles | 11 | Neutral |
| NZ Policy | serrator | 11 | Neutral |
| NZ Policy | shearwater | 11 | Neutral |
| NZ Policy | show | 11 | Neutral |
| NZ Policy | stomachs | 11 | Neutral |
| NZ Policy | surround | 11 | Neutral |
| NZ Policy | ten | 11 | Neutral |
| NZ Policy | thousands | 11 | Neutral |
| NZ Policy | topic | 11 | Neutral |
| NZ Policy | tourists | 11 | Neutral |
| NZ Policy | train | 11 | Neutral |
| NZ Policy | underpin | 11 | Neutral |
| NZ Policy | understood | 11 | Neutral |
| NZ Policy | undertake | 11 | Neutral |
| NZ Policy | unfair | 11 | Neutral |
| NZ Policy | unique | 11 | Neutral |
| NZ Policy | unnecessarily | 11 | Neutral |
| NZ Policy | vary | 11 | Neutral |
| NZ Policy | victoria | 11 | Neutral |
| NZ Policy | waikare | 11 | Neutral |
| NZ Policy | ways | 11 | Neutral |
| NZ Policy | wholly | 11 | Neutral |
| NZ Policy | word | 11 | Neutral |
| NZ Policy | zoned | 11 | Neutral |
| NZ Policy | align | 10 | Neutral |
| NZ Policy | aquaculturists | 10 | Neutral |
| NZ Policy | arbitrarily | 10 | Neutral |
| NZ Policy | asks | 10 | Neutral |
| NZ Policy | australian | 10 | Neutral |
| NZ Policy | automatically | 10 | Neutral |
| NZ Policy | basic | 10 | Neutral |
| NZ Policy | beatrix | 10 | Neutral |
| NZ Policy | bivalve | 10 | Neutral |
| NZ Policy | bodies | 10 | Neutral |
| NZ Policy | brabant | 10 | Neutral |
| NZ Policy | brief | 10 | Neutral |
| NZ Policy | chairperson | 10 | Neutral |
| NZ Policy | claim | 10 | Neutral |
| NZ Policy | committed | 10 | Neutral |
| NZ Policy | complexity | 10 | Neutral |
| NZ Policy | confirmed | 10 | Neutral |
| NZ Policy | contemplated | 10 | Neutral |
| NZ Policy | cove | 10 | Neutral |
| NZ Policy | cycle | 10 | Neutral |
| NZ Policy | declaration | 10 | Neutral |
| NZ Policy | design | 10 | Neutral |
| NZ Policy | distance | 10 | Neutral |
| NZ Policy | diversification | 10 | Neutral |
| NZ Policy | duplication | 10 | Neutral |
| NZ Policy | durville | 10 | Neutral |
| NZ Policy | edulis | 10 | Neutral |
| NZ Policy | employer | 10 | Neutral |
| NZ Policy | enforcement | 10 | Neutral |
| NZ Policy | entitled | 10 | Neutral |
| NZ Policy | equipment | 10 | Neutral |
| NZ Policy | estimated | 10 | Neutral |
| NZ Policy | exactly | 10 | Neutral |
| NZ Policy | explained | 10 | Neutral |
| NZ Policy | explanation | 10 | Neutral |
| NZ Policy | farm | 10 | Neutral |
| NZ Policy | france | 10 | Neutral |
| NZ Policy | frequently | 10 | Neutral |
| NZ Policy | funded | 10 | Neutral |
| NZ Policy | hand | 10 | Neutral |
| NZ Policy | hope | 10 | Neutral |
| NZ Policy | iama | 10 | Neutral |
| NZ Policy | idea | 10 | Neutral |
| NZ Policy | identification | 10 | Neutral |
| NZ Policy | initiated | 10 | Neutral |
| NZ Policy | jan | 10 | Neutral |
| NZ Policy | kaitiaki | 10 | Neutral |
| NZ Policy | kia | 10 | Neutral |
| NZ Policy | legally | 10 | Neutral |
| NZ Policy | live | 10 | Neutral |
| NZ Policy | look | 10 | Neutral |
| NZ Policy | lot | 10 | Neutral |
| NZ Policy | mail | 10 | Neutral |
| NZ Policy | marchant | 10 | Neutral |
| NZ Policy | meant | 10 | Neutral |
| NZ Policy | meet | 10 | Neutral |
| NZ Policy | mention | 10 | Neutral |
| NZ Policy | mesozooplankton | 10 | Neutral |
| NZ Policy | noncomply | 10 | Neutral |
| NZ Policy | open | 10 | Neutral |
| NZ Policy | overview | 10 | Neutral |
| NZ Policy | perceived | 10 | Neutral |
| NZ Policy | pests | 10 | Neutral |
| NZ Policy | picton | 10 | Neutral |
| NZ Policy | plac | 10 | Neutral |
| NZ Policy | population | 10 | Neutral |
| NZ Policy | portion | 10 | Neutral |
| NZ Policy | practicable | 10 | Neutral |
| NZ Policy | practices | 10 | Neutral |
| NZ Policy | presumably | 10 | Neutral |
| NZ Policy | programme | 10 | Neutral |
| NZ Policy | proportion | 10 | Neutral |
| NZ Policy | proximity | 10 | Neutral |
| NZ Policy | red | 10 | Neutral |
| NZ Policy | regularly | 10 | Neutral |
| NZ Policy | relevance | 10 | Neutral |
| NZ Policy | remuneration | 10 | Neutral |
| NZ Policy | rise | 10 | Neutral |
| NZ Policy | rotation | 10 | Neutral |
| NZ Policy | round | 10 | Neutral |
| NZ Policy | series | 10 | Neutral |
| NZ Policy | smws | 10 | Neutral |
| NZ Policy | specify | 10 | Neutral |
| NZ Policy | stands | 10 | Neutral |
| NZ Policy | started | 10 | Neutral |
| NZ Policy | subsections | 10 | Neutral |
| NZ Policy | talleys | 10 | Neutral |
| NZ Policy | techniques | 10 | Neutral |
| NZ Policy | tel | 10 | Neutral |
| NZ Policy | tham | 10 | Neutral |
| NZ Policy | thereby | 10 | Neutral |
| NZ Policy | thesis | 10 | Neutral |
| NZ Policy | third | 10 | Neutral |
| NZ Policy | title | 10 | Neutral |
| NZ Policy | unitary | 10 | Neutral |
| NZ Policy | usual | 10 | Neutral |
| NZ Policy | ventures | 10 | Neutral |
| NZ Policy | wdc | 10 | Neutral |
| NZ Policy | whilst | 10 | Neutral |
| NZ Policy | workers | 10 | Neutral |
| NZ Policy | support | 713 | Positive |
| NZ Policy | recommend | 610 | Positive |
| NZ Policy | right | 466 | Positive |
| NZ Policy | sustainable | 250 | Positive |
| NZ Policy | reform | 249 | Positive |
| NZ Policy | significant | 213 | Positive |
| NZ Policy | appropriate | 202 | Positive |
| NZ Policy | interests | 200 | Positive |
| NZ Policy | benefits | 194 | Positive |
| NZ Policy | available | 189 | Positive |
| NZ Policy | seafood | 182 | Positive |
| NZ Policy | important | 173 | Positive |
| NZ Policy | respect | 164 | Positive |
| NZ Policy | appeal | 149 | Positive |
| NZ Policy | food | 133 | Positive |
| NZ Policy | regard | 130 | Positive |
| NZ Policy | best | 128 | Positive |
| NZ Policy | work | 124 | Positive |
| NZ Policy | approve | 116 | Positive |
| NZ Policy | approval | 108 | Positive |
| NZ Policy | better | 101 | Positive |
| NZ Policy | efficient | 100 | Positive |
| NZ Policy | protection | 92 | Positive |
| NZ Policy | clear | 91 | Positive |
| NZ Policy | good | 83 | Positive |
| NZ Policy | adequate | 81 | Positive |
| NZ Policy | improve | 81 | Positive |
| NZ Policy | prefer | 76 | Positive |
| NZ Policy | consistent | 65 | Positive |
| NZ Policy | integrated | 58 | Positive |
| NZ Policy | trust | 57 | Positive |
| NZ Policy | flexibility | 55 | Positive |
| NZ Policy | enhance | 53 | Positive |
| NZ Policy | golden | 52 | Positive |
| NZ Policy | encourage | 49 | Positive |
| NZ Policy | protect | 49 | Positive |
| NZ Policy | effectively | 48 | Positive |
| NZ Policy | effective | 47 | Positive |
| NZ Policy | successful | 47 | Positive |
| NZ Policy | sufficient | 47 | Positive |
| NZ Policy | amenity | 44 | Positive |
| NZ Policy | great | 43 | Positive |
| NZ Policy | strong | 43 | Positive |
| NZ Policy | lead | 41 | Positive |
| NZ Policy | satisfied | 40 | Positive |
| NZ Policy | facilitate | 39 | Positive |
| NZ Policy | guidance | 39 | Positive |
| NZ Policy | suitable | 39 | Positive |
| NZ Policy | clearly | 36 | Positive |
| NZ Policy | progress | 35 | Positive |
| NZ Policy | enough | 32 | Positive |
| NZ Policy | success | 32 | Positive |
| NZ Policy | thank | 32 | Positive |
| NZ Policy | advantage | 30 | Positive |
| NZ Policy | proper | 30 | Positive |
| NZ Policy | properly | 30 | Positive |
| NZ Policy | reasonable | 29 | Positive |
| NZ Policy | confidence | 27 | Positive |
| NZ Policy | desirable | 27 | Positive |
| NZ Policy | positive | 27 | Positive |
| NZ Policy | relief | 26 | Positive |
| NZ Policy | free | 25 | Positive |
| NZ Policy | robust | 25 | Positive |
| NZ Policy | simplify | 25 | Positive |
| NZ Policy | transparent | 25 | Positive |
| NZ Policy | correct | 24 | Positive |
| NZ Policy | clean | 23 | Positive |
| NZ Policy | encourage | 23 | Positive |
| NZ Policy | fair | 23 | Positive |
| NZ Policy | reasonably | 23 | Positive |
| NZ Policy | top | 23 | Positive |
| NZ Policy | led | 22 | Positive |
| NZ Policy | clarity | 20 | Positive |
| NZ Policy | favour | 20 | Positive |
| NZ Policy | workable | 19 | Positive |
| NZ Policy | gain | 18 | Positive |
| NZ Policy | productive | 18 | Positive |
| NZ Policy | stronger | 18 | Positive |
| NZ Policy | worked | 18 | Positive |
| NZ Policy | enjoy | 16 | Positive |
| NZ Policy | gold | 16 | Positive |
| NZ Policy | tender | 16 | Positive |
| NZ Policy | outstanding | 15 | Positive |
| NZ Policy | streamlined | 15 | Positive |
| NZ Policy | timely | 15 | Positive |
| NZ Policy | useful | 15 | Positive |
| NZ Policy | faith | 14 | Positive |
| NZ Policy | glory | 14 | Positive |
| NZ Policy | innovation | 14 | Positive |
| NZ Policy | popular | 14 | Positive |
| NZ Policy | safe | 14 | Positive |
| NZ Policy | commitment | 13 | Positive |
| NZ Policy | contribution | 13 | Positive |
| NZ Policy | effectiveness | 13 | Positive |
| NZ Policy | ready | 13 | Positive |
| NZ Policy | stable | 13 | Positive |
| NZ Policy | adaptive | 12 | Positive |
| NZ Policy | proactive | 12 | Positive |
| NZ Policy | secure | 12 | Positive |
| NZ Policy | welcome | 12 | Positive |
| NZ Policy | abundance | 11 | Positive |
| NZ Policy | lead | 11 | Positive |
| NZ Policy | pleased | 11 | Positive |
| NZ Policy | readily | 11 | Positive |
| NZ Policy | remedy | 11 | Positive |
| NZ Policy | substantive | 11 | Positive |
| NZ Policy | works | 11 | Positive |
| NZ Policy | worth | 11 | Positive |
| NZ Policy | clearer | 10 | Positive |
| NZ Policy | endorsed | 10 | Positive |
| NZ Policy | excellent | 10 | Positive |
| NZ Policy | ideal | 10 | Positive |
| NZ Policy | integral | 10 | Positive |
| NZ Policy | sensible | 10 | Positive |
| NZ Policy | valuable | 10 | Positive |
| NZ Policy | variety | 10 | Positive |
| USA Mngt | impact | 3415 | Negative |
| USA Mngt | oil | 1643 | Negative |
| USA Mngt | concern | 1634 | Negative |
| USA Mngt | problem | 1589 | Negative |
| USA Mngt | serious | 1518 | Negative |
| USA Mngt | oppose | 1513 | Negative |
| USA Mngt | wildlife | 1173 | Negative |
| USA Mngt | fishing | 1025 | Negative |
| USA Mngt | escapes | 985 | Negative |
| USA Mngt | disease | 862 | Negative |
| USA Mngt | harm | 854 | Negative |
| USA Mngt | negative | 808 | Negative |
| USA Mngt | pollution | 796 | Negative |
| USA Mngt | spill | 778 | Negative |
| USA Mngt | parasite | 775 | Negative |
| USA Mngt | toxic | 762 | Negative |
| USA Mngt | hurricanes | 757 | Negative |
| USA Mngt | worst | 755 | Negative |
| USA Mngt | injury | 755 | Negative |
| USA Mngt | struggle | 752 | Negative |
| USA Mngt | hamper | 751 | Negative |
| USA Mngt | worsen | 750 | Negative |
| USA Mngt | dispersant | 750 | Negative |
| USA Mngt | unsure | 749 | Negative |
| USA Mngt | fail | 165 | Negative |
| USA Mngt | limit | 121 | Negative |
| USA Mngt | issue | 103 | Negative |
| USA Mngt | adverse | 92 | Negative |
| USA Mngt | risk | 145 | Negative |
| USA Mngt | overfish | 76 | Negative |
| USA Mngt | endangere | 76 | Negative |
| USA Mngt | violate | 73 | Negative |
| USA Mngt | critical | 58 | Negative |
| USA Mngt | conflict | 44 | Negative |
| USA Mngt | failure | 41 | Negative |
| USA Mngt | damage | 41 | Negative |
| USA Mngt | pathogen | 39 | Negative |
| USA Mngt | costs | 37 | Negative |
| USA Mngt | chemical | 36 | Negative |
| USA Mngt | waste | 34 | Negative |
| USA Mngt | mortality | 31 | Negative |
| USA Mngt | loss | 29 | Negative |
| USA Mngt | inconsistent | 29 | Negative |
| USA Mngt | competition | 25 | Negative |
| USA Mngt | restrict | 24 | Negative |
| USA Mngt | unreasonable | 23 | Negative |
| USA Mngt | resistance | 23 | Negative |
| USA Mngt | reject | 22 | Negative |
| USA Mngt | miss | 19 | Negative |
| USA Mngt | hard | 19 | Negative |
| USA Mngt | lack | 18 | Negative |
| USA Mngt | inadequate | 17 | Negative |
| USA Mngt | illegal | 17 | Negative |
| USA Mngt | difficult | 17 | Negative |
| USA Mngt | contamination | 17 | Negative |
| USA Mngt | contaminant | 17 | Negative |
| USA Mngt | revoke | 16 | Negative |
| USA Mngt | pressure | 15 | Negative |
| USA Mngt | disaster | 15 | Negative |
| USA Mngt | disapprove | 15 | Negative |
| USA Mngt | vague | 14 | Negative |
| USA Mngt | pesticides | 14 | Negative |
| USA Mngt | menhaden | 14 | Negative |
| USA Mngt | deficient | 14 | Negative |
| USA Mngt | unlikely | 13 | Negative |
| USA Mngt | reject | 13 | Negative |
| USA Mngt | interfere | 13 | Negative |
| USA Mngt | kill | 12 | Negative |
| USA Mngt | insufficient | 12 | Negative |
| USA Mngt | inappropriate | 12 | Negative |
| USA Mngt | entanglement | 12 | Negative |
| USA Mngt | deny | 12 | Negative |
| USA Mngt | dead | 12 | Negative |
| USA Mngt | arbitrary | 12 | Negative |
| USA Mngt | uncertainty | 11 | Negative |
| USA Mngt | threat | 11 | Negative |
| USA Mngt | suffer | 11 | Negative |
| USA Mngt | rouge | 11 | Negative |
| USA Mngt | questionable | 11 | Negative |
| USA Mngt | lice | 11 | Negative |
| USA Mngt | jeopardize | 11 | Negative |
| USA Mngt | emergency | 11 | Negative |
| USA Mngt | deficiencies | 11 | Negative |
| USA Mngt | bacteria | 11 | Negative |
| USA Mngt | unknown | 10 | Negative |
| USA Mngt | threaten | 10 | Negative |
| USA Mngt | prohibit | 10 | Negative |
| USA Mngt | oversight | 10 | Negative |
| USA Mngt | inevitable | 10 | Negative |
| USA Mngt | impose | 10 | Negative |
| USA Mngt | hardships | 10 | Negative |
| USA Mngt | detrimental | 10 | Negative |
| USA Mngt | ocean | 4666 | Neutral |
| USA Mngt | allow | 2425 | Neutral |
| USA Mngt | years | 1585 | Neutral |
| USA Mngt | facilities | 1128 | Neutral |
| USA Mngt | agency | 1122 | Neutral |
| USA Mngt | increase | 901 | Neutral |
| USA Mngt | farm | 882 | Neutral |
| USA Mngt | communities | 844 | Neutral |
| USA Mngt | human | 831 | Neutral |
| USA Mngt | local | 815 | Neutral |
| USA Mngt | follow | 811 | Neutral |
| USA Mngt | associated | 808 | Neutral |
| USA Mngt | already | 803 | Neutral |
| USA Mngt | people | 802 | Neutral |
| USA Mngt | exist | 800 | Neutral |
| USA Mngt | forward | 799 | Neutral |
| USA Mngt | longterm | 795 | Neutral |
| USA Mngt | yet | 781 | Neutral |
| USA Mngt | dear | 780 | Neutral |
| USA Mngt | particular | 779 | Neutral |
| USA Mngt | move | 778 | Neutral |
| USA Mngt | history | 776 | Neutral |
| USA Mngt | global | 775 | Neutral |
| USA Mngt | cover | 775 | Neutral |
| USA Mngt | recover | 759 | Neutral |
| USA Mngt | range | 759 | Neutral |
| USA Mngt | elsewhere | 759 | Neutral |
| USA Mngt | today | 757 | Neutral |
| USA Mngt | storm | 757 | Neutral |
| USA Mngt | roy | 757 | Neutral |
| USA Mngt | wide | 756 | Neutral |
| USA Mngt | tropical | 756 | Neutral |
| USA Mngt | thousand | 756 | Neutral |
| USA Mngt | fin | 756 | Neutral |
| USA Mngt | administr | 755 | Neutral |
| USA Mngt | voiced | 753 | Neutral |
| USA Mngt | continue | 753 | Neutral |
| USA Mngt | letter | 750 | Neutral |
| USA Mngt | plus | 748 | Neutral |
| USA Mngt | write | 742 | Neutral |
| USA Mngt | plan | 692 | Neutral |
| USA Mngt | regulate | 666 | Neutral |
| USA Mngt | nmfs | 609 | Neutral |
| USA Mngt | require | 589 | Neutral |
| USA Mngt | council | 542 | Neutral |
| USA Mngt | permit | 525 | Neutral |
| USA Mngt | environment | 516 | Neutral |
| USA Mngt | fmp | 514 | Neutral |
| USA Mngt | offshore | 460 | Neutral |
| USA Mngt | management | 355 | Neutral |
| USA Mngt | federal | 300 | Neutral |
| USA Mngt | fishery | 286 | Neutral |
| USA Mngt | fisheries | 285 | Neutral |
| USA Mngt | public | 284 | Neutral |
| USA Mngt | gom | 270 | Neutral |
| USA Mngt | include | 263 | Neutral |
| USA Mngt | develop | 245 | Neutral |
| USA Mngt | implement | 241 | Neutral |
| USA Mngt | operation | 225 | Neutral |
| USA Mngt | provide | 224 | Neutral |
| USA Mngt | water | 221 | Neutral |
| USA Mngt | add | 220 | Neutral |
| USA Mngt | consider | 219 | Neutral |
| USA Mngt | alternative | 205 | Neutral |
| USA Mngt | efforts | 201 | Neutral |
| USA Mngt | state | 199 | Neutral |
| USA Mngt | use | 196 | Neutral |
| USA Mngt | act | 188 | Neutral |
| USA Mngt | health | 185 | Neutral |
| USA Mngt | effects | 181 | Neutral |
| USA Mngt | system | 171 | Neutral |
| USA Mngt | see | 170 | Neutral |
| USA Mngt | action | 169 | Neutral |
| USA Mngt | site | 164 | Neutral |
| USA Mngt | salmon | 164 | Neutral |
| USA Mngt | nepa | 163 | Neutral |
| USA Mngt | economic | 163 | Neutral |
| USA Mngt | resources | 162 | Neutral |
| USA Mngt | industry | 161 | Neutral |
| USA Mngt | need | 159 | Neutral |
| USA Mngt | waters | 155 | Neutral |
| USA Mngt | authority | 152 | Neutral |
| USA Mngt | potential | 151 | Neutral |
| USA Mngt | msa | 150 | Neutral |
| USA Mngt | states | 146 | Neutral |
| USA Mngt | policy | 145 | Neutral |
| USA Mngt | define | 144 | Neutral |
| USA Mngt | standard | 143 | Neutral |
| USA Mngt | secretary | 143 | Neutral |
| USA Mngt | supra | 140 | Neutral |
| USA Mngt | usc | 139 | Neutral |
| USA Mngt | make | 132 | Neutral |
| USA Mngt | conservation | 131 | Neutral |
| USA Mngt | process | 129 | Neutral |
| USA Mngt | evaluate | 128 | Neutral |
| USA Mngt | habitat | 127 | Neutral |
| USA Mngt | note | 124 | Neutral |
| USA Mngt | specific | 122 | Neutral |
| USA Mngt | establish | 122 | Neutral |
| USA Mngt | commercial | 122 | Neutral |
| USA Mngt | review | 118 | Neutral |
| USA Mngt | change | 117 | Neutral |
| USA Mngt | production | 114 | Neutral |
| USA Mngt | regional | 113 | Neutral |
| USA Mngt | meet | 113 | Neutral |
| USA Mngt | measure | 112 | Neutral |
| USA Mngt | activity | 111 | Neutral |
| USA Mngt | used | 110 | Neutral |
| USA Mngt | likely | 107 | Neutral |
| USA Mngt | however | 107 | Neutral |
| USA Mngt | program | 106 | Neutral |
| USA Mngt | coastal | 105 | Neutral |
| USA Mngt | united_states | 104 | Neutral |
| USA Mngt | final | 103 | Neutral |
| USA Mngt | address | 103 | Neutral |
| USA Mngt | rules | 101 | Neutral |
| USA Mngt | therefore | 100 | Neutral |
| USA Mngt | purpose | 100 | Neutral |
| USA Mngt | give | 100 | Neutral |
| USA Mngt | ecosystem | 100 | Neutral |
| USA Mngt | affect | 99 | Neutral |
| USA Mngt | manage | 98 | Neutral |
| USA Mngt | without | 97 | Neutral |
| USA Mngt | determination | 96 | Neutral |
| USA Mngt | cage | 95 | Neutral |
| USA Mngt | population | 94 | Neutral |
| USA Mngt | harvest | 94 | Neutral |
| USA Mngt | feed | 93 | Neutral |
| USA Mngt | statement | 90 | Neutral |
| USA Mngt | necessary | 90 | Neutral |
| USA Mngt | law | 90 | Neutral |
| USA Mngt | large | 90 | Neutral |
| USA Mngt | plans | 88 | Neutral |
| USA Mngt | areas | 88 | Neutral |
| USA Mngt | aquatic | 87 | Neutral |
| USA Mngt | stocks | 86 | Neutral |
| USA Mngt | future | 84 | Neutral |
| USA Mngt | example | 84 | Neutral |
| USA Mngt | suite | 83 | Neutral |
| USA Mngt | south | 83 | Neutral |
| USA Mngt | service | 83 | Neutral |
| USA Mngt | animal | 83 | Neutral |
| USA Mngt | disease | 82 | Neutral |
| USA Mngt | current | 82 | Neutral |
| USA Mngt | based | 82 | Neutral |
| USA Mngt | industrial | 81 | Neutral |
| USA Mngt | ensure | 81 | Neutral |
| USA Mngt | analysis | 81 | Neutral |
| USA Mngt | watch | 80 | Neutral |
| USA Mngt | orleans | 80 | Neutral |
| USA Mngt | sea | 79 | Neutral |
| USA Mngt | esa | 78 | Neutral |
| USA Mngt | area | 78 | Neutral |
| USA Mngt | animals | 78 | Neutral |
| USA Mngt | monitor | 77 | Neutral |
| USA Mngt | general | 77 | Neutral |
| USA Mngt | draft | 77 | Neutral |
| USA Mngt | cultured | 77 | Neutral |
| USA Mngt | whether | 75 | Neutral |
| USA Mngt | take | 75 | Neutral |
| USA Mngt | fishermen | 75 | Neutral |
| USA Mngt | decision | 75 | Neutral |
| USA Mngt | result | 74 | Neutral |
| USA Mngt | drugs | 74 | Neutral |
| USA Mngt | quality | 70 | Neutral |
| USA Mngt | natural | 70 | Neutral |
| USA Mngt | conditions | 69 | Neutral |
| USA Mngt | create | 66 | Neutral |
| USA Mngt | assessment | 66 | Neutral |
| USA Mngt | submitted | 65 | Neutral |
| USA Mngt | report | 63 | Neutral |
| USA Mngt | boem | 63 | Neutral |
| USA Mngt | atlantic | 63 | Neutral |
| USA Mngt | research | 62 | Neutral |
| USA Mngt | point | 62 | Neutral |
| USA Mngt | fact | 61 | Neutral |
| USA Mngt | report | 60 | Neutral |
| USA Mngt | october | 60 | Neutral |
| USA Mngt | long | 60 | Neutral |
| USA Mngt | level | 60 | Neutral |
| USA Mngt | project | 59 | Neutral |
| USA Mngt | office | 59 | Neutral |
| USA Mngt | guideline | 59 | Neutral |
| USA Mngt | eez | 59 | Neutral |
| USA Mngt | washington | 58 | Neutral |
| USA Mngt | thus | 58 | Neutral |
| USA Mngt | science | 58 | Neutral |
| USA Mngt | pens | 58 | Neutral |
| USA Mngt | application | 58 | Neutral |
| USA Mngt | vessels | 57 | Neutral |
| USA Mngt | part | 57 | Neutral |
| USA Mngt | net | 57 | Neutral |
| USA Mngt | identify | 57 | Neutral |
| USA Mngt | direct | 57 | Neutral |
| USA Mngt | currently | 57 | Neutral |
| USA Mngt | action | 57 | Neutral |
| USA Mngt | cumulative | 56 | Neutral |
| USA Mngt | biological | 56 | Neutral |
| USA Mngt | authorize | 56 | Neutral |
| USA Mngt | amount | 56 | Neutral |
| USA Mngt | similar | 55 | Neutral |
| USA Mngt | opportunity | 55 | Neutral |
| USA Mngt | minimize | 55 | Neutral |
| USA Mngt | mandate | 55 | Neutral |
| USA Mngt | instead | 55 | Neutral |
| USA Mngt | demonstrate | 55 | Neutral |
| USA Mngt | recognize | 54 | Neutral |
| USA Mngt | percent | 54 | Neutral |
| USA Mngt | genetic | 54 | Neutral |
| USA Mngt | various | 53 | Neutral |
| USA Mngt | energy | 53 | Neutral |
| USA Mngt | complete | 53 | Neutral |
| USA Mngt | assess | 53 | Neutral |
| USA Mngt | prior | 52 | Neutral |
| USA Mngt | order | 52 | Neutral |
| USA Mngt | mean | 52 | Neutral |
| USA Mngt | inform | 52 | Neutral |
| USA Mngt | epa | 52 | Neutral |
| USA Mngt | population | 51 | Neutral |
| USA Mngt | street | 50 | Neutral |
| USA Mngt | rather | 50 | Neutral |
| USA Mngt | prevent | 50 | Neutral |
| USA Mngt | might | 50 | Neutral |
| USA Mngt | fmps | 50 | Neutral |
| USA Mngt | due | 50 | Neutral |
| USA Mngt | cir | 50 | Neutral |
| USA Mngt | stock | 49 | Neutral |
| USA Mngt | source | 49 | Neutral |
| USA Mngt | set | 49 | Neutral |
| USA Mngt | major | 49 | Neutral |
| USA Mngt | individual | 49 | Neutral |
| USA Mngt | florida | 49 | Neutral |
| USA Mngt | coast | 49 | Neutral |
| USA Mngt | access | 49 | Neutral |
| USA Mngt | occur | 48 | Neutral |
| USA Mngt | notice | 48 | Neutral |
| USA Mngt | listed | 48 | Neutral |
| USA Mngt | issued | 48 | Neutral |
| USA Mngt | finalize | 48 | Neutral |
| USA Mngt | avoid | 48 | Neutral |
| USA Mngt | antibiotic | 48 | Neutral |
| USA Mngt | zone | 47 | Neutral |
| USA Mngt | regard | 47 | Neutral |
| USA Mngt | modify | 47 | Neutral |
| USA Mngt | data | 47 | Neutral |
| USA Mngt | control | 47 | Neutral |
| USA Mngt | broodstock | 47 | Neutral |
| USA Mngt | beckstimpert | 47 | Neutral |
| USA Mngt | goal | 46 | Neutral |
| USA Mngt | finally | 46 | Neutral |
| USA Mngt | detail | 46 | Neutral |
| USA Mngt | department | 46 | Neutral |
| USA Mngt | applicant | 46 | Neutral |
| USA Mngt | several | 45 | Neutral |
| USA Mngt | recreational | 45 | Neutral |
| USA Mngt | petersburg | 45 | Neutral |
| USA Mngt | legal | 45 | Neutral |
| USA Mngt | gas | 45 | Neutral |
| USA Mngt | found | 45 | Neutral |
| USA Mngt | criteria | 45 | Neutral |
| USA Mngt | com | 45 | Neutral |
| USA Mngt | submitter | 44 | Neutral |
| USA Mngt | study | 44 | Neutral |
| USA Mngt | shall | 44 | Neutral |
| USA Mngt | numerous | 44 | Neutral |
| USA Mngt | native | 44 | Neutral |
| USA Mngt | business | 44 | Neutral |
| USA Mngt | believe | 44 | Neutral |
| USA Mngt | yield | 43 | Neutral |
| USA Mngt | mitigation | 43 | Neutral |
| USA Mngt | government | 43 | Neutral |
| USA Mngt | cause | 43 | Neutral |
| USA Mngt | term | 42 | Neutral |
| USA Mngt | stated | 42 | Neutral |
| USA Mngt | operator | 42 | Neutral |
| USA Mngt | indicate | 42 | Neutral |
| USA Mngt | ecological | 42 | Neutral |
| USA Mngt | congress | 42 | Neutral |
| USA Mngt | basis | 42 | Neutral |
| USA Mngt | another | 42 | Neutral |
| USA Mngt | amendment | 42 | Neutral |
| USA Mngt | uses | 41 | Neutral |
| USA Mngt | studies | 41 | Neutral |
| USA Mngt | statute | 41 | Neutral |
| USA Mngt | staff | 41 | Neutral |
| USA Mngt | present | 41 | Neutral |
| USA Mngt | essential | 41 | Neutral |
| USA Mngt | court | 41 | Neutral |
| USA Mngt | conduct | 41 | Neutral |
| USA Mngt | avenue | 41 | Neutral |
| USA Mngt | vessel | 40 | Neutral |
| USA Mngt | simply | 40 | Neutral |
| USA Mngt | prefer | 40 | Neutral |
| USA Mngt | ocs | 40 | Neutral |
| USA Mngt | extent | 40 | Neutral |
| USA Mngt | culture | 40 | Neutral |
| USA Mngt | become | 40 | Neutral |
| USA Mngt | proposal | 39 | Neutral |
| USA Mngt | possible | 39 | Neutral |
| USA Mngt | january | 39 | Neutral |
| USA Mngt | inc | 39 | Neutral |
| USA Mngt | caribbean | 39 | Neutral |
| USA Mngt | among | 39 | Neutral |
| USA Mngt | submit | 38 | Neutral |
| USA Mngt | statutory | 38 | Neutral |
| USA Mngt | safety | 38 | Neutral |
| USA Mngt | relevant | 38 | Neutral |
| USA Mngt | reference | 38 | Neutral |
| USA Mngt | please | 38 | Neutral |
| USA Mngt | commerce | 38 | Neutral |
| USA Mngt | aquacultur | 38 | Neutral |
| USA Mngt | reduce | 37 | Neutral |
| USA Mngt | produce | 37 | Neutral |
| USA Mngt | memo | 37 | Neutral |
| USA Mngt | market | 37 | Neutral |
| USA Mngt | investment | 37 | Neutral |
| USA Mngt | factor | 37 | Neutral |
| USA Mngt | attempt | 37 | Neutral |
| USA Mngt | upon | 36 | Neutral |
| USA Mngt | structure | 36 | Neutral |
| USA Mngt | southeast | 36 | Neutral |
| USA Mngt | procedures | 36 | Neutral |
| USA Mngt | others | 36 | Neutral |
| USA Mngt | oct | 36 | Neutral |
| USA Mngt | less | 36 | Neutral |
| USA Mngt | city | 36 | Neutral |
| USA Mngt | certain | 36 | Neutral |
| USA Mngt | benthic | 36 | Neutral |
| USA Mngt | beach | 36 | Neutral |
| USA Mngt | supplemental | 35 | Neutral |
| USA Mngt | since | 35 | Neutral |
| USA Mngt | place | 35 | Neutral |
| USA Mngt | otherwise | 35 | Neutral |
| USA Mngt | event | 35 | Neutral |
| USA Mngt | drive | 35 | Neutral |
| USA Mngt | consultation | 35 | Neutral |
| USA Mngt | approach | 35 | Neutral |
| USA Mngt | organization | 34 | Neutral |
| USA Mngt | look | 34 | Neutral |
| USA Mngt | least | 34 | Neutral |
| USA Mngt | inspection | 34 | Neutral |
| USA Mngt | full | 34 | Neutral |
| USA Mngt | finfish | 34 | Neutral |
| USA Mngt | case | 34 | Neutral |
| USA Mngt | record | 33 | Neutral |
| USA Mngt | programs | 33 | Neutral |
| USA Mngt | party | 33 | Neutral |
| USA Mngt | maximum | 33 | Neutral |
| USA Mngt | designed | 33 | Neutral |
| USA Mngt | apt | 33 | Neutral |
| USA Mngt | world | 32 | Neutral |
| USA Mngt | term | 32 | Neutral |
| USA Mngt | suggest | 32 | Neutral |
| USA Mngt | spring | 32 | Neutral |
| USA Mngt | scientific | 32 | Neutral |
| USA Mngt | practices | 32 | Neutral |
| USA Mngt | members | 32 | Neutral |
| USA Mngt | mammals | 32 | Neutral |
| USA Mngt | life | 32 | Neutral |
| USA Mngt | compliance | 32 | Neutral |
| USA Mngt | show | 31 | Neutral |
| USA Mngt | request | 31 | Neutral |
| USA Mngt | provisions | 31 | Neutral |
| USA Mngt | protected | 31 | Neutral |
| USA Mngt | organism | 31 | Neutral |
| USA Mngt | mercury | 31 | Neutral |
| USA Mngt | incorporate | 31 | Neutral |
| USA Mngt | fed | 31 | Neutral |
| USA Mngt | comply | 31 | Neutral |
| USA Mngt | box | 31 | Neutral |
| USA Mngt | apply | 31 | Neutral |
| USA Mngt | andor | 31 | Neutral |
| USA Mngt | zones | 30 | Neutral |
| USA Mngt | veterinary | 30 | Neutral |
| USA Mngt | threatened | 30 | Neutral |
| USA Mngt | reg | 30 | Neutral |
| USA Mngt | private | 30 | Neutral |
| USA Mngt | practicable | 30 | Neutral |
| USA Mngt | noaas | 30 | Neutral |
| USA Mngt | fully | 30 | Neutral |
| USA Mngt | exclusive | 30 | Neutral |
| USA Mngt | effect | 30 | Neutral |
| USA Mngt | caught | 30 | Neutral |
| USA Mngt | track | 29 | Neutral |
| USA Mngt | substantial | 29 | Neutral |
| USA Mngt | resource | 29 | Neutral |
| USA Mngt | raised | 29 | Neutral |
| USA Mngt | outside | 29 | Neutral |
| USA Mngt | mitigate | 29 | Neutral |
| USA Mngt | location | 29 | Neutral |
| USA Mngt | land | 29 | Neutral |
| USA Mngt | intended | 29 | Neutral |
| USA Mngt | high | 29 | Neutral |
| USA Mngt | find | 29 | Neutral |
| USA Mngt | either | 29 | Neutral |
| USA Mngt | corps | 29 | Neutral |
| USA Mngt | consumer | 29 | Neutral |
| USA Mngt | consequences | 29 | Neutral |
| USA Mngt | applicable | 29 | Neutral |
| USA Mngt | work | 28 | Neutral |
| USA Mngt | via | 28 | Neutral |
| USA Mngt | strongly | 28 | Neutral |
| USA Mngt | social | 28 | Neutral |
| USA Mngt | smith | 28 | Neutral |
| USA Mngt | product | 28 | Neutral |
| USA Mngt | primary | 28 | Neutral |
| USA Mngt | ofthe | 28 | Neutral |
| USA Mngt | msy | 28 | Neutral |
| USA Mngt | located | 28 | Neutral |
| USA Mngt | framework | 28 | Neutral |
| USA Mngt | far | 28 | Neutral |
| USA Mngt | doctrine | 28 | Neutral |
| USA Mngt | detailed | 28 | Neutral |
| USA Mngt | viable | 27 | Neutral |
| USA Mngt | taken | 27 | Neutral |
| USA Mngt | robert | 27 | Neutral |
| USA Mngt | linda | 27 | Neutral |
| USA Mngt | interpretation | 27 | Neutral |
| USA Mngt | growth | 27 | Neutral |
| USA Mngt | fishmeal | 27 | Neutral |
| USA Mngt | emphasis | 27 | Neutral |
| USA Mngt | different | 27 | Neutral |
| USA Mngt | center | 27 | Neutral |
| USA Mngt | annual | 27 | Neutral |
| USA Mngt | vote | 26 | Neutral |
| USA Mngt | sound | 26 | Neutral |
| USA Mngt | size | 26 | Neutral |
| USA Mngt | region | 26 | Neutral |
| USA Mngt | procedural | 26 | Neutral |
| USA Mngt | prepare | 26 | Neutral |
| USA Mngt | obtain | 26 | Neutral |
| USA Mngt | neither | 26 | Neutral |
| USA Mngt | maine | 26 | Neutral |
| USA Mngt | magnusonstevens | 26 | Neutral |
| USA Mngt | jobs | 26 | Neutral |
| USA Mngt | hatchery | 26 | Neutral |
| USA Mngt | gasrenewable | 26 | Neutral |
| USA Mngt | especially | 26 | Neutral |
| USA Mngt | bird | 26 | Neutral |
| USA Mngt | barbara | 26 | Neutral |
| USA Mngt | take | 25 | Neutral |
| USA Mngt | supply | 25 | Neutral |
| USA Mngt | spread | 25 | Neutral |
| USA Mngt | programmatic | 25 | Neutral |
| USA Mngt | opinion | 25 | Neutral |
| USA Mngt | legally | 25 | Neutral |
| USA Mngt | laws | 25 | Neutral |
| USA Mngt | institute | 25 | Neutral |
| USA Mngt | duties | 25 | Neutral |
| USA Mngt | contrary | 25 | Neutral |
| USA Mngt | april | 25 | Neutral |
| USA Mngt | amend | 25 | Neutral |
| USA Mngt | socioeconomic | 24 | Neutral |
| USA Mngt | published | 24 | Neutral |
| USA Mngt | nation | 24 | Neutral |
| USA Mngt | lisa | 24 | Neutral |
| USA Mngt | grant | 24 | Neutral |
| USA Mngt | foreseeable | 24 | Neutral |
| USA Mngt | estimated | 24 | Neutral |
| USA Mngt | discretion | 24 | Neutral |
| USA Mngt | demand | 24 | Neutral |
| USA Mngt | days | 24 | Neutral |
| USA Mngt | caused | 24 | Neutral |
| USA Mngt | actually | 24 | Neutral |
| USA Mngt | using | 23 | Neutral |
| USA Mngt | surround | 23 | Neutral |
| USA Mngt | status | 23 | Neutral |
| USA Mngt | short | 23 | Neutral |
| USA Mngt | seis | 23 | Neutral |
| USA Mngt | rin | 23 | Neutral |
| USA Mngt | pounds | 23 | Neutral |
| USA Mngt | posted | 23 | Neutral |
| USA Mngt | manner | 23 | Neutral |
| USA Mngt | lease | 23 | Neutral |
| USA Mngt | help | 23 | Neutral |
| USA Mngt | held | 23 | Neutral |
| USA Mngt | force | 23 | Neutral |
| USA Mngt | evidence | 23 | Neutral |
| USA Mngt | effluent | 23 | Neutral |
| USA Mngt | duration | 23 | Neutral |
| USA Mngt | shellfish | 22 | Neutral |
| USA Mngt | richard | 22 | Neutral |
| USA Mngt | responsibility | 22 | Neutral |
| USA Mngt | response | 22 | Neutral |
| USA Mngt | prepared | 22 | Neutral |
| USA Mngt | potentially | 22 | Neutral |
| USA Mngt | platforms | 22 | Neutral |
| USA Mngt | mississippi | 22 | Neutral |
| USA Mngt | jurisdiction | 22 | Neutral |
| USA Mngt | initial | 22 | Neutral |
| USA Mngt | higher | 22 | Neutral |
| USA Mngt | group | 22 | Neutral |
| USA Mngt | financial | 22 | Neutral |
| USA Mngt | director | 22 | Neutral |
| USA Mngt | contribute | 22 | Neutral |
| USA Mngt | clos | 22 | Neutral |
| USA Mngt | catch | 22 | Neutral |
| USA Mngt | casebycase | 22 | Neutral |
| USA Mngt | breed | 22 | Neutral |
| USA Mngt | bay | 22 | Neutral |
| USA Mngt | aphis | 22 | Neutral |
| USA Mngt | administrative | 22 | Neutral |
| USA Mngt | able | 22 | Neutral |
| USA Mngt | rfc | 21 | Neutral |
| USA Mngt | reduction | 21 | Neutral |
| USA Mngt | recent | 21 | Neutral |
| USA Mngt | products | 21 | Neutral |
| USA Mngt | participation | 21 | Neutral |
| USA Mngt | moreover | 21 | Neutral |
| USA Mngt | lands | 21 | Neutral |
| USA Mngt | hear | 21 | Neutral |
| USA Mngt | fda | 21 | Neutral |
| USA Mngt | experience | 21 | Neutral |
| USA Mngt | every | 21 | Neutral |
| USA Mngt | etc | 21 | Neutral |
| USA Mngt | domestic | 21 | Neutral |
| USA Mngt | ceq | 21 | Neutral |
| USA Mngt | august | 21 | Neutral |
| USA Mngt | american | 21 | Neutral |
| USA Mngt | although | 21 | Neutral |
| USA Mngt | account | 21 | Neutral |
| USA Mngt | value | 20 | Neutral |
| USA Mngt | together | 20 | Neutral |
| USA Mngt | texas | 20 | Neutral |
| USA Mngt | sharon | 20 | Neutral |
| USA Mngt | revised | 20 | Neutral |
| USA Mngt | responsible | 20 | Neutral |
| USA Mngt | register | 20 | Neutral |
| USA Mngt | promote | 20 | Neutral |
| USA Mngt | possibly | 20 | Neutral |
| USA Mngt | pen | 20 | Neutral |
| USA Mngt | past | 20 | Neutral |
| USA Mngt | oregon | 20 | Neutral |
| USA Mngt | often | 20 | Neutral |
| USA Mngt | north | 20 | Neutral |
| USA Mngt | metairie | 20 | Neutral |
| USA Mngt | list | 20 | Neutral |
| USA Mngt | involved | 20 | Neutral |
| USA Mngt | investor | 20 | Neutral |
| USA Mngt | ignores | 20 | Neutral |
| USA Mngt | grow | 20 | Neutral |
| USA Mngt | grounds | 20 | Neutral |
| USA Mngt | furthermore | 20 | Neutral |
| USA Mngt | fold | 20 | Neutral |
| USA Mngt | consult | 20 | Neutral |
| USA Mngt | beyond | 20 | Neutral |
| USA Mngt | accord | 20 | Neutral |
| USA Mngt | west | 19 | Neutral |
| USA Mngt | throughout | 19 | Neutral |
| USA Mngt | technology | 19 | Neutral |
| USA Mngt | technologies | 19 | Neutral |
| USA Mngt | task | 19 | Neutral |
| USA Mngt | result | 19 | Neutral |
| USA Mngt | price | 19 | Neutral |
| USA Mngt | previously | 19 | Neutral |
| USA Mngt | plan | 19 | Neutral |
| USA Mngt | near | 19 | Neutral |
| USA Mngt | minimum | 19 | Neutral |
| USA Mngt | mention | 19 | Neutral |
| USA Mngt | martin | 19 | Neutral |
| USA Mngt | light | 19 | Neutral |
| USA Mngt | interior | 19 | Neutral |
| USA Mngt | carol | 19 | Neutral |
| USA Mngt | bycatch | 19 | Neutral |
| USA Mngt | speis | 18 | Neutral |
| USA Mngt | small | 18 | Neutral |
| USA Mngt | rod | 18 | Neutral |
| USA Mngt | restrictions | 18 | Neutral |
| USA Mngt | rely | 18 | Neutral |
| USA Mngt | recirculate | 18 | Neutral |
| USA Mngt | pursuant | 18 | Neutral |
| USA Mngt | property | 18 | Neutral |
| USA Mngt | organic | 18 | Neutral |
| USA Mngt | optimum | 18 | Neutral |
| USA Mngt | opportunities | 18 | Neutral |
| USA Mngt | narrow | 18 | Neutral |
| USA Mngt | miles | 18 | Neutral |
| USA Mngt | interactions | 18 | Neutral |
| USA Mngt | highly | 18 | Neutral |
| USA Mngt | explicitly | 18 | Neutral |
| USA Mngt | expert | 18 | Neutral |
| USA Mngt | decrease | 18 | Neutral |
| USA Mngt | czma | 18 | Neutral |
| USA Mngt | company | 18 | Neutral |
| USA Mngt | collected | 18 | Neutral |
| USA Mngt | circle | 18 | Neutral |
| USA Mngt | chapter | 18 | Neutral |
| USA Mngt | capacity | 18 | Neutral |
| USA Mngt | austin | 18 | Neutral |
| USA Mngt | altered | 18 | Neutral |
| USA Mngt | agriculture | 18 | Neutral |
| USA Mngt | unless | 17 | Neutral |
| USA Mngt | stock | 17 | Neutral |
| USA Mngt | stating | 17 | Neutral |
| USA Mngt | society | 17 | Neutral |
| USA Mngt | scale | 17 | Neutral |
| USA Mngt | sandra | 17 | Neutral |
| USA Mngt | sanctuary | 17 | Neutral |
| USA Mngt | removal | 17 | Neutral |
| USA Mngt | questions | 17 | Neutral |
| USA Mngt | put | 17 | Neutral |
| USA Mngt | pose | 17 | Neutral |
| USA Mngt | objective | 17 | Neutral |
| USA Mngt | migratory | 17 | Neutral |
| USA Mngt | mentioned | 17 | Neutral |
| USA Mngt | little | 17 | Neutral |
| USA Mngt | june | 17 | Neutral |
| USA Mngt | international | 17 | Neutral |
| USA Mngt | indeed | 17 | Neutral |
| USA Mngt | gordon | 17 | Neutral |
| USA Mngt | goals | 17 | Neutral |
| USA Mngt | equipment | 17 | Neutral |
| USA Mngt | early | 17 | Neutral |
| USA Mngt | counsel | 17 | Neutral |
| USA Mngt | context | 17 | Neutral |
| USA Mngt | conserve | 17 | Neutral |
| USA Mngt | conclusion | 17 | Neutral |
| USA Mngt | cases | 17 | Neutral |
| USA Mngt | attached | 17 | Neutral |
| USA Mngt | veterinarians | 16 | Neutral |
| USA Mngt | traditional | 16 | Neutral |
| USA Mngt | test | 16 | Neutral |
| USA Mngt | supplement | 16 | Neutral |
| USA Mngt | sediments | 16 | Neutral |
| USA Mngt | reduced | 16 | Neutral |
| USA Mngt | reason | 16 | Neutral |
| USA Mngt | provision | 16 | Neutral |
| USA Mngt | produced | 16 | Neutral |
| USA Mngt | privileges | 16 | Neutral |
| USA Mngt | methods | 16 | Neutral |
| USA Mngt | lower | 16 | Neutral |
| USA Mngt | lake | 16 | Neutral |
| USA Mngt | importance | 16 | Neutral |
| USA Mngt | generations | 16 | Neutral |
| USA Mngt | generally | 16 | Neutral |
| USA Mngt | february | 16 | Neutral |
| USA Mngt | expected | 16 | Neutral |
| USA Mngt | entirely | 16 | Neutral |
| USA Mngt | documentation | 16 | Neutral |
| USA Mngt | discharge | 16 | Neutral |
| USA Mngt | continued | 16 | Neutral |
| USA Mngt | consequently | 16 | Neutral |
| USA Mngt | citing | 16 | Neutral |
| USA Mngt | chris | 16 | Neutral |
| USA Mngt | brown | 16 | Neutral |
| USA Mngt | avma | 16 | Neutral |
| USA Mngt | along | 16 | Neutral |
| USA Mngt | allocation | 16 | Neutral |
| USA Mngt | twice | 15 | Neutral |
| USA Mngt | turtle | 15 | Neutral |
| USA Mngt | transfer | 15 | Neutral |
| USA Mngt | sincer | 15 | Neutral |
| USA Mngt | seems | 15 | Neutral |
| USA Mngt | second | 15 | Neutral |
| USA Mngt | scott | 15 | Neutral |
| USA Mngt | rulemake | 15 | Neutral |
| USA Mngt | role | 15 | Neutral |
| USA Mngt | results | 15 | Neutral |
| USA Mngt | removed | 15 | Neutral |
| USA Mngt | remedial | 15 | Neutral |
| USA Mngt | prices | 15 | Neutral |
| USA Mngt | president | 15 | Neutral |
| USA Mngt | pacific | 15 | Neutral |
| USA Mngt | nature | 15 | Neutral |
| USA Mngt | licensed | 15 | Neutral |
| USA Mngt | intent | 15 | Neutral |
| USA Mngt | instance | 15 | Neutral |
| USA Mngt | input | 15 | Neutral |
| USA Mngt | ifthe | 15 | Neutral |
| USA Mngt | houston | 15 | Neutral |
| USA Mngt | hill | 15 | Neutral |
| USA Mngt | hereinafter | 15 | Neutral |
| USA Mngt | gulfs | 15 | Neutral |
| USA Mngt | grow | 15 | Neutral |
| USA Mngt | groups | 15 | Neutral |
| USA Mngt | genetically_modified | 15 | Neutral |
| USA Mngt | fww | 15 | Neutral |
| USA Mngt | feel | 15 | Neutral |
| USA Mngt | fax | 15 | Neutral |
| USA Mngt | farmers | 15 | Neutral |
| USA Mngt | evans | 15 | Neutral |
| USA Mngt | coral | 15 | Neutral |
| USA Mngt | combined | 15 | Neutral |
| USA Mngt | collection | 15 | Neutral |
| USA Mngt | citizens | 15 | Neutral |
| USA Mngt | bob | 15 | Neutral |
| USA Mngt | want | 14 | Neutral |
| USA Mngt | version | 14 | Neutral |
| USA Mngt | urge | 14 | Neutral |
| USA Mngt | university | 14 | Neutral |
| USA Mngt | understand | 14 | Neutral |
| USA Mngt | ten | 14 | Neutral |
| USA Mngt | technical | 14 | Neutral |
| USA Mngt | taylor | 14 | Neutral |
| USA Mngt | stewards | 14 | Neutral |
| USA Mngt | say | 14 | Neutral |
| USA Mngt | rul | 14 | Neutral |
| USA Mngt | rose | 14 | Neutral |
| USA Mngt | reported | 14 | Neutral |
| USA Mngt | relative | 14 | Neutral |
| USA Mngt | red | 14 | Neutral |
| USA Mngt | rebuild | 14 | Neutral |
| USA Mngt | raise | 14 | Neutral |
| USA Mngt | province | 14 | Neutral |
| USA Mngt | producers | 14 | Neutral |
| USA Mngt | presence | 14 | Neutral |
| USA Mngt | practice | 14 | Neutral |
| USA Mngt | physical | 14 | Neutral |
| USA Mngt | panama | 14 | Neutral |
| USA Mngt | overall | 14 | Neutral |
| USA Mngt | oceans | 14 | Neutral |
| USA Mngt | numbers | 14 | Neutral |
| USA Mngt | nothing | 14 | Neutral |
| USA Mngt | mentions | 14 | Neutral |
| USA Mngt | mediterranean | 14 | Neutral |
| USA Mngt | matter | 14 | Neutral |
| USA Mngt | mark | 14 | Neutral |
| USA Mngt | license | 14 | Neutral |
| USA Mngt | legislative | 14 | Neutral |
| USA Mngt | legislation | 14 | Neutral |
| USA Mngt | know | 14 | Neutral |
| USA Mngt | juveniles | 14 | Neutral |
| USA Mngt | issuance | 14 | Neutral |
| USA Mngt | extremely | 14 | Neutral |
| USA Mngt | end | 14 | Neutral |
| USA Mngt | division | 14 | Neutral |
| USA Mngt | despite | 14 | Neutral |
| USA Mngt | deficit | 14 | Neutral |
| USA Mngt | decommission | 14 | Neutral |
| USA Mngt | continue | 14 | Neutral |
| USA Mngt | committee | 14 | Neutral |
| USA Mngt | carry | 14 | Neutral |
| USA Mngt | carried | 14 | Neutral |
| USA Mngt | assumes | 14 | Neutral |
| USA Mngt | association | 14 | Neutral |
| USA Mngt | whale | 13 | Neutral |
| USA Mngt | tuna | 13 | Neutral |
| USA Mngt | tim | 13 | Neutral |
| USA Mngt | third | 13 | Neutral |
| USA Mngt | tampa | 13 | Neutral |
| USA Mngt | subpopulation | 13 | Neutral |
| USA Mngt | steve | 13 | Neutral |
| USA Mngt | space | 13 | Neutral |
| USA Mngt | soybean | 13 | Neutral |
| USA Mngt | snapper | 13 | Neutral |
| USA Mngt | shelf | 13 | Neutral |
| USA Mngt | sense | 13 | Neutral |
| USA Mngt | sanctuaries | 13 | Neutral |
| USA Mngt | release | 13 | Neutral |
| USA Mngt | received | 13 | Neutral |
| USA Mngt | reasons | 13 | Neutral |
| USA Mngt | protect | 13 | Neutral |
| USA Mngt | propose | 13 | Neutral |
| USA Mngt | propose | 13 | Neutral |
| USA Mngt | promot | 13 | Neutral |
| USA Mngt | offer | 13 | Neutral |
| USA Mngt | nitrogen | 13 | Neutral |
| USA Mngt | never | 13 | Neutral |
| USA Mngt | move | 13 | Neutral |
| USA Mngt | magnitude | 13 | Neutral |
| USA Mngt | known | 13 | Neutral |
| USA Mngt | key | 13 | Neutral |
| USA Mngt | infrastructure | 13 | Neutral |
| USA Mngt | ibid | 13 | Neutral |
| USA Mngt | gulfport | 13 | Neutral |
| USA Mngt | fulfill | 13 | Neutral |
| USA Mngt | forth | 13 | Neutral |
| USA Mngt | focus | 13 | Neutral |
| USA Mngt | exist | 13 | Neutral |
| USA Mngt | entities | 13 | Neutral |
| USA Mngt | document | 13 | Neutral |
| USA Mngt | describe | 13 | Neutral |
| USA Mngt | defense | 13 | Neutral |
| USA Mngt | deepwater | 13 | Neutral |
| USA Mngt | davis | 13 | Neutral |
| USA Mngt | countries | 13 | Neutral |
| USA Mngt | claims | 13 | Neutral |
| USA Mngt | cheryl | 13 | Neutral |
| USA Mngt | captive | 13 | Neutral |
| USA Mngt | broad | 13 | Neutral |
| USA Mngt | blue | 13 | Neutral |
| USA Mngt | behalf | 13 | Neutral |
| USA Mngt | aspect | 13 | Neutral |
| USA Mngt | applied | 13 | Neutral |
| USA Mngt | alaska | 13 | Neutral |
| USA Mngt | wastes | 12 | Neutral |
| USA Mngt | virginia | 12 | Neutral |
| USA Mngt | understand | 12 | Neutral |
| USA Mngt | ultimately | 12 | Neutral |
| USA Mngt | twenty | 12 | Neutral |
| USA Mngt | transgenic | 12 | Neutral |
| USA Mngt | special | 12 | Neutral |
| USA Mngt | speci | 12 | Neutral |
| USA Mngt | shore | 12 | Neutral |
| USA Mngt | sedimentation | 12 | Neutral |
| USA Mngt | scenario | 12 | Neutral |
| USA Mngt | road | 12 | Neutral |
| USA Mngt | rights | 12 | Neutral |
| USA Mngt | resistant | 12 | Neutral |
| USA Mngt | residues | 12 | Neutral |
| USA Mngt | researchers | 12 | Neutral |
| USA Mngt | remain | 12 | Neutral |
| USA Mngt | released | 12 | Neutral |
| USA Mngt | reduce | 12 | Neutral |
| USA Mngt | professor | 12 | Neutral |
| USA Mngt | produce | 12 | Neutral |
| USA Mngt | processes | 12 | Neutral |
| USA Mngt | power | 12 | Neutral |
| USA Mngt | perhaps | 12 | Neutral |
| USA Mngt | participants | 12 | Neutral |
| USA Mngt | partially | 12 | Neutral |
| USA Mngt | offish | 12 | Neutral |
| USA Mngt | nations | 12 | Neutral |
| USA Mngt | monitor | 12 | Neutral |
| USA Mngt | miller | 12 | Neutral |
| USA Mngt | mandeville | 12 | Neutral |
| USA Mngt | low | 12 | Neutral |
| USA Mngt | live | 12 | Neutral |
| USA Mngt | lane | 12 | Neutral |
| USA Mngt | justify | 12 | Neutral |
| USA Mngt | journal | 12 | Neutral |
| USA Mngt | indirect | 12 | Neutral |
| USA Mngt | formal | 12 | Neutral |
| USA Mngt | five | 12 | Neutral |
| USA Mngt | fishes | 12 | Neutral |
| USA Mngt | existence | 12 | Neutral |
| USA Mngt | eric | 12 | Neutral |
| USA Mngt | entity | 12 | Neutral |
| USA Mngt | cultural | 12 | Neutral |
| USA Mngt | cost | 12 | Neutral |
| USA Mngt | continental | 12 | Neutral |
| USA Mngt | contain | 12 | Neutral |
| USA Mngt | construction | 12 | Neutral |
| USA Mngt | connected | 12 | Neutral |
| USA Mngt | congressional | 12 | Neutral |
| USA Mngt | club | 12 | Neutral |
| USA Mngt | ciguatera | 12 | Neutral |
| USA Mngt | certification | 12 | Neutral |
| USA Mngt | capital | 12 | Neutral |
| USA Mngt | campbell | 12 | Neutral |
| USA Mngt | california | 12 | Neutral |
| USA Mngt | british_columbia | 12 | Neutral |
| USA Mngt | bond | 12 | Neutral |
| USA Mngt | baseline | 12 | Neutral |
| USA Mngt | anything | 12 | Neutral |
| USA Mngt | allen | 12 | Neutral |
| USA Mngt | written | 11 | Neutral |
| USA Mngt | word | 11 | Neutral |
| USA Mngt | wat | 11 | Neutral |
| USA Mngt | valid | 11 | Neutral |
| USA Mngt | turtles | 11 | Neutral |
| USA Mngt | turn | 11 | Neutral |
| USA Mngt | transportation | 11 | Neutral |
| USA Mngt | toward | 11 | Neutral |
| USA Mngt | though | 11 | Neutral |
| USA Mngt | surveys | 11 | Neutral |
| USA Mngt | summary | 11 | Neutral |
| USA Mngt | sharks | 11 | Neutral |
| USA Mngt | serve | 11 | Neutral |
| USA Mngt | sent | 11 | Neutral |
| USA Mngt | selection | 11 | Neutral |
| USA Mngt | seek | 11 | Neutral |
| USA Mngt | sector | 11 | Neutral |
| USA Mngt | rivers | 11 | Neutral |
| USA Mngt | renewal | 11 | Neutral |
| USA Mngt | relies | 11 | Neutral |
| USA Mngt | regul | 11 | Neutral |
| USA Mngt | rate | 11 | Neutral |
| USA Mngt | prey | 11 | Neutral |
| USA Mngt | precautionary | 11 | Neutral |
| USA Mngt | parts | 11 | Neutral |
| USA Mngt | particularly | 11 | Neutral |
| USA Mngt | originally | 11 | Neutral |
| USA Mngt | obligation | 11 | Neutral |
| USA Mngt | nutrients | 11 | Neutral |
| USA Mngt | norwegian | 11 | Neutral |
| USA Mngt | network | 11 | Neutral |
| USA Mngt | moored | 11 | Neutral |
| USA Mngt | minor | 11 | Neutral |
| USA Mngt | minimal | 11 | Neutral |
| USA Mngt | mean | 11 | Neutral |
| USA Mngt | maria | 11 | Neutral |
| USA Mngt | majority | 11 | Neutral |
| USA Mngt | maintain | 11 | Neutral |
| USA Mngt | louisiana | 11 | Neutral |
| USA Mngt | likelihood | 11 | Neutral |
| USA Mngt | kona | 11 | Neutral |
| USA Mngt | horizon | 11 | Neutral |
| USA Mngt | hopkins | 11 | Neutral |
| USA Mngt | green | 11 | Neutral |
| USA Mngt | flood | 11 | Neutral |
| USA Mngt | fisheri | 11 | Neutral |
| USA Mngt | financ | 11 | Neutral |
| USA Mngt | except | 11 | Neutral |
| USA Mngt | enforcement | 11 | Neutral |
| USA Mngt | east | 11 | Neutral |
| USA Mngt | done | 11 | Neutral |
| USA Mngt | distance | 11 | Neutral |
| USA Mngt | cynthia | 11 | Neutral |
| USA Mngt | cusp | 11 | Neutral |
| USA Mngt | course | 11 | Neutral |
| USA Mngt | corporations | 11 | Neutral |
| USA Mngt | coordination | 11 | Neutral |
| USA Mngt | containment | 11 | Neutral |
| USA Mngt | consumption | 11 | Neutral |
| USA Mngt | code | 11 | Neutral |
| USA Mngt | cobia | 11 | Neutral |
| USA Mngt | coalition | 11 | Neutral |
| USA Mngt | cap | 11 | Neutral |
| USA Mngt | billion | 11 | Neutral |
| USA Mngt | bill | 11 | Neutral |
| USA Mngt | baton | 11 | Neutral |
| USA Mngt | background | 11 | Neutral |
| USA Mngt | away | 11 | Neutral |
| USA Mngt | artificial | 11 | Neutral |
| USA Mngt | aquaculturists | 11 | Neutral |
| USA Mngt | appears | 11 | Neutral |
| USA Mngt | ann | 11 | Neutral |
| USA Mngt | america | 11 | Neutral |
| USA Mngt | agree | 11 | Neutral |
| USA Mngt | across | 11 | Neutral |
| USA Mngt | accept | 11 | Neutral |
| USA Mngt | worldwide | 10 | Neutral |
| USA Mngt | whole | 10 | Neutral |
| USA Mngt | underly | 10 | Neutral |
| USA Mngt | trade | 10 | Neutral |
| USA Mngt | track | 10 | Neutral |
| USA Mngt | threshold | 10 | Neutral |
| USA Mngt | thing | 10 | Neutral |
| USA Mngt | substantially | 10 | Neutral |
| USA Mngt | sierra | 10 | Neutral |
| USA Mngt | shortterm | 10 | Neutral |
| USA Mngt | share | 10 | Neutral |
| USA Mngt | set | 10 | Neutral |
| USA Mngt | serv | 10 | Neutral |
| USA Mngt | seriously | 10 | Neutral |
| USA Mngt | sep | 10 | Neutral |
| USA Mngt | seem | 10 | Neutral |
| USA Mngt | section | 10 | Neutral |
| USA Mngt | san | 10 | Neutral |
| USA Mngt | reportable | 10 | Neutral |
| USA Mngt | report | 10 | Neutral |
| USA Mngt | remove | 10 | Neutral |
| USA Mngt | reef | 10 | Neutral |
| USA Mngt | receive | 10 | Neutral |
| USA Mngt | rear | 10 | Neutral |
| USA Mngt | real | 10 | Neutral |
| USA Mngt | proposes | 10 | Neutral |
| USA Mngt | productivity | 10 | Neutral |
| USA Mngt | process | 10 | Neutral |
| USA Mngt | prevent | 10 | Neutral |
| USA Mngt | preserve | 10 | Neutral |
| USA Mngt | port | 10 | Neutral |
| USA Mngt | placed | 10 | Neutral |
| USA Mngt | phd | 10 | Neutral |
| USA Mngt | pass | 10 | Neutral |
| USA Mngt | parameters | 10 | Neutral |
| USA Mngt | ongo | 10 | Neutral |
| USA Mngt | old | 10 | Neutral |
| USA Mngt | nutrient | 10 | Neutral |
| USA Mngt | naylor | 10 | Neutral |
| USA Mngt | load | 10 | Neutral |
| USA Mngt | lands | 10 | Neutral |
| USA Mngt | inherent | 10 | Neutral |
| USA Mngt | imported | 10 | Neutral |
| USA Mngt | house | 10 | Neutral |
| USA Mngt | hope | 10 | Neutral |
| USA Mngt | heavy | 10 | Neutral |
| USA Mngt | guard | 10 | Neutral |
| USA Mngt | granted | 10 | Neutral |
| USA Mngt | fwc | 10 | Neutral |
| USA Mngt | four | 10 | Neutral |
| USA Mngt | forest | 10 | Neutral |
| USA Mngt | farmer | 10 | Neutral |
| USA Mngt | expressly | 10 | Neutral |
| USA Mngt | expertise | 10 | Neutral |
| USA Mngt | expand | 10 | Neutral |
| USA Mngt | equal | 10 | Neutral |
| USA Mngt | drill | 10 | Neutral |
| USA Mngt | donald | 10 | Neutral |
| USA Mngt | dictionary | 10 | Neutral |
| USA Mngt | deep | 10 | Neutral |
| USA Mngt | deem | 10 | Neutral |
| USA Mngt | conserv | 10 | Neutral |
| USA Mngt | choice | 10 | Neutral |
| USA Mngt | build | 10 | Neutral |
| USA Mngt | build | 10 | Neutral |
| USA Mngt | block | 10 | Neutral |
| USA Mngt | biodiversity | 10 | Neutral |
| USA Mngt | average | 10 | Neutral |
| USA Mngt | attorney | 10 | Neutral |
| USA Mngt | approximately | 10 | Neutral |
| USA Mngt | anderson | 10 | Neutral |
| USA Mngt | analyze | 10 | Neutral |
| USA Mngt | almost | 10 | Neutral |
| USA Mngt | alabama | 10 | Neutral |
| USA Mngt | actual | 10 | Neutral |
| USA Mngt | food | 199 | Positive |
| USA Mngt | significant | 160 | Positive |
| USA Mngt | support | 122 | Positive |
| USA Mngt | approve | 121 | Positive |
| USA Mngt | seafood | 111 | Positive |
| USA Mngt | sustainable | 107 | Positive |
| USA Mngt | available | 99 | Positive |
| USA Mngt | benefit | 91 | Positive |
| USA Mngt | consistent | 88 | Positive |
| USA Mngt | recommend | 69 | Positive |
| USA Mngt | reasonable | 65 | Positive |
| USA Mngt | appropriate | 65 | Positive |
| USA Mngt | protect | 62 | Positive |
| USA Mngt | trust | 60 | Positive |
| USA Mngt | best | 54 | Positive |
| USA Mngt | adequate | 52 | Positive |
| USA Mngt | important | 51 | Positive |
| USA Mngt | interest | 49 | Positive |
| USA Mngt | great | 46 | Positive |
| USA Mngt | protection | 44 | Positive |
| USA Mngt | clear | 44 | Positive |
| USA Mngt | sufficient | 43 | Positive |
| USA Mngt | approval | 43 | Positive |
| USA Mngt | effective | 34 | Positive |
| USA Mngt | thank | 33 | Positive |
| USA Mngt | right | 30 | Positive |
| USA Mngt | encourage | 29 | Positive |
| USA Mngt | clearly | 28 | Positive |
| USA Mngt | work | 27 | Positive |
| USA Mngt | assurance | 27 | Positive |
| USA Mngt | substantive | 22 | Positive |
| USA Mngt | comprehensive | 22 | Positive |
| USA Mngt | better | 22 | Positive |
| USA Mngt | respect | 19 | Positive |
| USA Mngt | enough | 19 | Positive |
| USA Mngt | clean | 19 | Positive |
| USA Mngt | meanful | 17 | Positive |
| USA Mngt | reasonably | 16 | Positive |
| USA Mngt | promise | 16 | Positive |
| USA Mngt | lead | 16 | Positive |
| USA Mngt | saint | 15 | Positive |
| USA Mngt | proper | 15 | Positive |
| USA Mngt | improve | 15 | Positive |
| USA Mngt | enhance | 15 | Positive |
| USA Mngt | fair | 14 | Positive |
| USA Mngt | sincerely | 13 | Positive |
| USA Mngt | selective | 13 | Positive |
| USA Mngt | safe | 13 | Positive |
| USA Mngt | properly | 13 | Positive |
| USA Mngt | healthy | 13 | Positive |
| USA Mngt | capable | 13 | Positive |
| USA Mngt | adaptive | 13 | Positive |
| USA Mngt | survival | 12 | Positive |
| USA Mngt | free | 11 | Positive |
| USA Mngt | variety | 10 | Positive |
| USA Mngt | regard | 10 | Positive |
| USA Mngt | guidance | 10 | Positive |
| USA Mngt | gain | 10 | Positive |
| USA Mngt | facilitate | 10 | Positive |
| USA Policy | impact | 415 | Negative |
| USA Policy | wildlife | 413 | Negative |
| USA Policy | concern | 112 | Negative |
| USA Policy | fishing | 84 | Negative |
| USA Policy | discharge | 70 | Negative |
| USA Policy | pollution | 59 | Negative |
| USA Policy | lice | 53 | Negative |
| USA Policy | risk | 51 | Negative |
| USA Policy | adverse | 47 | Negative |
| USA Policy | chemical | 44 | Negative |
| USA Policy | prevent | 44 | Negative |
| USA Policy | cost | 43 | Negative |
| USA Policy | critical | 42 | Negative |
| USA Policy | harm | 41 | Negative |
| USA Policy | endangered | 37 | Negative |
| USA Policy | limited | 36 | Negative |
| USA Policy | lack | 35 | Negative |
| USA Policy | parasite | 34 | Negative |
| USA Policy | disease | 33 | Negative |
| USA Policy | waste | 32 | Negative |
| USA Policy | escapes | 31 | Negative |
| USA Policy | conflict | 27 | Negative |
| USA Policy | loss | 27 | Negative |
| USA Policy | threat | 22 | Negative |
| USA Policy | risk | 21 | Negative |
| USA Policy | predatory | 20 | Negative |
| USA Policy | unfortunately | 20 | Negative |
| USA Policy | pressure | 19 | Negative |
| USA Policy | negative | 17 | Negative |
| USA Policy | serious | 17 | Negative |
| USA Policy | antibiotics | 16 | Negative |
| USA Policy | degradation | 16 | Negative |
| USA Policy | pesticides | 16 | Negative |
| USA Policy | problem | 16 | Negative |
| USA Policy | threats | 16 | Negative |
| USA Policy | competition | 15 | Negative |
| USA Policy | invasive | 15 | Negative |
| USA Policy | infection | 13 | Negative |
| USA Policy | limit | 13 | Negative |
| USA Policy | oversight | 13 | Negative |
| USA Policy | parasite | 13 | Negative |
| USA Policy | limitation | 12 | Negative |
| USA Policy | prohibit | 12 | Negative |
| USA Policy | uncertainty | 12 | Negative |
| USA Policy | absence | 11 | Negative |
| USA Policy | damage | 11 | Negative |
| USA Policy | entanglement | 11 | Negative |
| USA Policy | virus | 11 | Negative |
| USA Policy | conflict | 10 | Negative |
| USA Policy | effluent | 10 | Negative |
| USA Policy | problem | 10 | Negative |
| USA Policy | nonindigenous | 10 | Negative |
| USA Policy | policy | 886 | Neutral |
| USA Policy | environment | 582 | Neutral |
| USA Policy | ocean | 488 | Neutral |
| USA Policy | develop | 467 | Neutral |
| USA Policy | water | 450 | Neutral |
| USA Policy | salmon | 421 | Neutral |
| USA Policy | federal | 417 | Neutral |
| USA Policy | resource | 342 | Neutral |
| USA Policy | state | 321 | Neutral |
| USA Policy | draft | 317 | Neutral |
| USA Policy | use | 312 | Neutral |
| USA Policy | management | 297 | Neutral |
| USA Policy | industry | 294 | Neutral |
| USA Policy | permit | 289 | Neutral |
| USA Policy | include | 281 | Neutral |
| USA Policy | provide | 252 | Neutral |
| USA Policy | coastal | 223 | Neutral |
| USA Policy | fisheries | 211 | Neutral |
| USA Policy | report | 211 | Neutral |
| USA Policy | production | 209 | Neutral |
| USA Policy | plan | 206 | Neutral |
| USA Policy | require | 200 | Neutral |
| USA Policy | offshore | 187 | Neutral |
| USA Policy | act | 186 | Neutral |
| USA Policy | shellfish | 183 | Neutral |
| USA Policy | project | 179 | Neutral |
| USA Policy | program | 177 | Neutral |
| USA Policy | action | 176 | Neutral |
| USA Policy | research | 175 | Neutral |
| USA Policy | agency | 171 | Neutral |
| USA Policy | ooa | 169 | Neutral |
| USA Policy | effect | 164 | Neutral |
| USA Policy | exist | 162 | Neutral |
| USA Policy | potential | 162 | Neutral |
| USA Policy | regulate | 161 | Neutral |
| USA Policy | public | 159 | Neutral |
| USA Policy | channel | 155 | Neutral |
| USA Policy | increase | 154 | Neutral |
| USA Policy | regulatory | 153 | Neutral |
| USA Policy | commercial | 151 | Neutral |
| USA Policy | california | 150 | Neutral |
| USA Policy | farmed | 150 | Neutral |
| USA Policy | review | 150 | Neutral |
| USA Policy | economic | 149 | Neutral |
| USA Policy | process | 146 | Neutral |
| USA Policy | island | 145 | Neutral |
| USA Policy | operation | 137 | Neutral |
| USA Policy | sea | 135 | Neutral |
| USA Policy | implement | 134 | Neutral |
| USA Policy | active | 133 | Neutral |
| USA Policy | department | 131 | Neutral |
| USA Policy | habitat | 130 | Neutral |
| USA Policy | commerce | 129 | Neutral |
| USA Policy | states | 127 | Neutral |
| USA Policy | facilities | 126 | Neutral |
| USA Policy | consider | 126 | Neutral |
| USA Policy | cinms | 125 | Neutral |
| USA Policy | pacific | 123 | Neutral |
| USA Policy | ecosystem | 119 | Neutral |
| USA Policy | population | 117 | Neutral |
| USA Policy | statement | 117 | Neutral |
| USA Policy | issue | 114 | Neutral |
| USA Policy | areas | 113 | Neutral |
| USA Policy | establish | 111 | Neutral |
| USA Policy | council | 109 | Neutral |
| USA Policy | stocks | 107 | Neutral |
| USA Policy | future | 106 | Neutral |
| USA Policy | addition | 105 | Neutral |
| USA Policy | science | 102 | Neutral |
| USA Policy | commission | 98 | Neutral |
| USA Policy | authority | 97 | Neutral |
| USA Policy | conservation | 97 | Neutral |
| USA Policy | opportunity | 96 | Neutral |
| USA Policy | oil | 94 | Neutral |
| USA Policy | result | 94 | Neutral |
| USA Policy | noaa | 93 | Neutral |
| USA Policy | atlantic | 92 | Neutral |
| USA Policy | domestic | 92 | Neutral |
| USA Policy | effort | 90 | Neutral |
| USA Policy | fishery | 89 | Neutral |
| USA Policy | feed | 88 | Neutral |
| USA Policy | framework | 88 | Neutral |
| USA Policy | april | 86 | Neutral |
| USA Policy | standard | 86 | Neutral |
| USA Policy | year | 86 | Neutral |
| USA Policy | principle | 85 | Neutral |
| USA Policy | priority | 85 | Neutral |
| USA Policy | regional | 85 | Neutral |
| USA Policy | address | 84 | Neutral |
| USA Policy | allow | 84 | Neutral |
| USA Policy | need | 84 | Neutral |
| USA Policy | aquacultur | 83 | Neutral |
| USA Policy | following | 83 | Neutral |
| USA Policy | scientific | 83 | Neutral |
| USA Policy | see | 83 | Neutral |
| USA Policy | local | 82 | Neutral |
| USA Policy | washington | 82 | Neutral |
| USA Policy | alaska | 81 | Neutral |
| USA Policy | escape | 81 | Neutral |
| USA Policy | native | 81 | Neutral |
| USA Policy | united_states | 80 | Neutral |
| USA Policy | agencies | 79 | Neutral |
| USA Policy | ensure | 79 | Neutral |
| USA Policy | specific | 77 | Neutral |
| USA Policy | world | 77 | Neutral |
| USA Policy | area | 75 | Neutral |
| USA Policy | based | 75 | Neutral |
| USA Policy | doc | 75 | Neutral |
| USA Policy | nation | 75 | Neutral |
| USA Policy | congress | 74 | Neutral |
| USA Policy | fin | 74 | Neutral |
| USA Policy | suggest | 74 | Neutral |
| USA Policy | high | 71 | Neutral |
| USA Policy | practices | 71 | Neutral |
| USA Policy | produce | 70 | Neutral |
| USA Policy | hawaii | 69 | Neutral |
| USA Policy | however | 69 | Neutral |
| USA Policy | viewed | 69 | Neutral |
| USA Policy | data | 68 | Neutral |
| USA Policy | health | 68 | Neutral |
| USA Policy | lease | 68 | Neutral |
| USA Policy | legislation | 68 | Neutral |
| USA Policy | take | 67 | Neutral |
| USA Policy | approach | 66 | Neutral |
| USA Policy | cause | 66 | Neutral |
| USA Policy | affect | 65 | Neutral |
| USA Policy | association | 65 | Neutral |
| USA Policy | global | 65 | Neutral |
| USA Policy | naylor | 65 | Neutral |
| USA Policy | net | 65 | Neutral |
| USA Policy | demand | 64 | Neutral |
| USA Policy | goals | 63 | Neutral |
| USA Policy | particular | 63 | Neutral |
| USA Policy | quality | 63 | Neutral |
| USA Policy | disease | 62 | Neutral |
| USA Policy | example | 61 | Neutral |
| USA Policy | genetic | 61 | Neutral |
| USA Policy | associated | 60 | Neutral |
| USA Policy | given | 60 | Neutral |
| USA Policy | growth | 60 | Neutral |
| USA Policy | bunsick | 59 | Neutral |
| USA Policy | ecological | 59 | Neutral |
| USA Policy | eez | 59 | Neutral |
| USA Policy | occur | 59 | Neutral |
| USA Policy | recognize | 59 | Neutral |
| USA Policy | restoration | 59 | Neutral |
| USA Policy | sector | 59 | Neutral |
| USA Policy | coast | 58 | Neutral |
| USA Policy | levels | 58 | Neutral |
| USA Policy | stock | 58 | Neutral |
| USA Policy | puget_sound | 57 | Neutral |
| USA Policy | aquatic | 56 | Neutral |
| USA Policy | community | 56 | Neutral |
| USA Policy | current | 56 | Neutral |
| USA Policy | help | 56 | Neutral |
| USA Policy | likely | 56 | Neutral |
| USA Policy | program | 56 | Neutral |
| USA Policy | zone | 56 | Neutral |
| USA Policy | facility | 55 | Neutral |
| USA Policy | monitoring | 55 | Neutral |
| USA Policy | staff | 55 | Neutral |
| USA Policy | whether | 55 | Neutral |
| USA Policy | region | 54 | Neutral |
| USA Policy | santa | 54 | Neutral |
| USA Policy | human | 53 | Neutral |
| USA Policy | platform | 52 | Neutral |
| USA Policy | stakeholders | 52 | Neutral |
| USA Policy | barbara | 51 | Neutral |
| USA Policy | term | 51 | Neutral |
| USA Policy | advisory | 50 | Neutral |
| USA Policy | job | 50 | Neutral |
| USA Policy | mariculture | 50 | Neutral |
| USA Policy | adopt | 49 | Neutral |
| USA Policy | assessment | 49 | Neutral |
| USA Policy | biological | 49 | Neutral |
| USA Policy | forage | 49 | Neutral |
| USA Policy | role | 49 | Neutral |
| USA Policy | secretary | 49 | Neutral |
| USA Policy | siting | 49 | Neutral |
| USA Policy | animal | 47 | Neutral |
| USA Policy | decisions | 47 | Neutral |
| USA Policy | expansion | 47 | Neutral |
| USA Policy | growing | 47 | Neutral |
| USA Policy | purpose | 47 | Neutral |
| USA Policy | scale | 47 | Neutral |
| USA Policy | forward | 46 | Neutral |
| USA Policy | hatchery | 46 | Neutral |
| USA Policy | pew | 46 | Neutral |
| USA Policy | please | 46 | Neutral |
| USA Policy | specifically | 46 | Neutral |
| USA Policy | study | 46 | Neutral |
| USA Policy | according | 45 | Neutral |
| USA Policy | code | 45 | Neutral |
| USA Policy | currently | 45 | Neutral |
| USA Policy | final | 45 | Neutral |
| USA Policy | initiative | 45 | Neutral |
| USA Policy | responsible | 45 | Neutral |
| USA Policy | similar | 45 | Neutral |
| USA Policy | supply | 45 | Neutral |
| USA Policy | working | 45 | Neutral |
| USA Policy | control | 44 | Neutral |
| USA Policy | make | 44 | Neutral |
| USA Policy | service | 44 | Neutral |
| USA Policy | several | 44 | Neutral |
| USA Policy | without | 44 | Neutral |
| USA Policy | committee | 43 | Neutral |
| USA Policy | industrial | 43 | Neutral |
| USA Policy | meet | 43 | Neutral |
| USA Policy | member | 43 | Neutral |
| USA Policy | private | 43 | Neutral |
| USA Policy | shall | 43 | Neutral |
| USA Policy | spring | 43 | Neutral |
| USA Policy | systems | 43 | Neutral |
| USA Policy | general | 42 | Neutral |
| USA Policy | government | 42 | Neutral |
| USA Policy | juvenile | 42 | Neutral |
| USA Policy | level | 42 | Neutral |
| USA Policy | manner | 42 | Neutral |
| USA Policy | product | 42 | Neutral |
| USA Policy | reduce | 42 | Neutral |
| USA Policy | services | 42 | Neutral |
| USA Policy | since | 42 | Neutral |
| USA Policy | sites | 42 | Neutral |
| USA Policy | believe | 41 | Neutral |
| USA Policy | case | 41 | Neutral |
| USA Policy | eis | 41 | Neutral |
| USA Policy | focus | 41 | Neutral |
| USA Policy | trade | 41 | Neutral |
| USA Policy | usc | 41 | Neutral |
| USA Policy | among | 40 | Neutral |
| USA Policy | analysis | 40 | Neutral |
| USA Policy | director | 40 | Neutral |
| USA Policy | guidelines | 40 | Neutral |
| USA Policy | harvest | 40 | Neutral |
| USA Policy | products | 40 | Neutral |
| USA Policy | silver | 40 | Neutral |
| USA Policy | due | 39 | Neutral |
| USA Policy | investment | 39 | Neutral |
| USA Policy | nepa | 39 | Neutral |
| USA Policy | appendix | 38 | Neutral |
| USA Policy | cage | 38 | Neutral |
| USA Policy | documented | 38 | Neutral |
| USA Policy | goal | 38 | Neutral |
| USA Policy | group | 38 | Neutral |
| USA Policy | order | 38 | Neutral |
| USA Policy | pens | 38 | Neutral |
| USA Policy | stated | 38 | Neutral |
| USA Policy | technology | 38 | Neutral |
| USA Policy | already | 37 | Neutral |
| USA Policy | bay | 37 | Neutral |
| USA Policy | energy | 37 | Neutral |
| USA Policy | import | 37 | Neutral |
| USA Policy | institute | 37 | Neutral |
| USA Policy | social | 37 | Neutral |
| USA Policy | benthic | 36 | Neutral |
| USA Policy | center | 36 | Neutral |
| USA Policy | change | 36 | Neutral |
| USA Policy | full | 36 | Neutral |
| USA Policy | listed | 36 | Neutral |
| USA Policy | manager | 36 | Neutral |
| USA Policy | near | 36 | Neutral |
| USA Policy | nearshore | 36 | Neutral |
| USA Policy | rather | 36 | Neutral |
| USA Policy | regarding | 36 | Neutral |
| USA Policy | feeds | 35 | Neutral |
| USA Policy | greater | 35 | Neutral |
| USA Policy | international | 35 | Neutral |
| USA Policy | maine | 35 | Neutral |
| USA Policy | major | 35 | Neutral |
| USA Policy | nonnative | 35 | Neutral |
| USA Policy | pilot | 35 | Neutral |
| USA Policy | point | 35 | Neutral |
| USA Policy | recent | 35 | Neutral |
| USA Policy | sbc | 35 | Neutral |
| USA Policy | site | 35 | Neutral |
| USA Policy | technologies | 35 | Neutral |
| USA Policy | conditions | 34 | Neutral |
| USA Policy | council | 34 | Neutral |
| USA Policy | dear | 34 | Neutral |
| USA Policy | expanded | 34 | Neutral |
| USA Policy | fishermen | 34 | Neutral |
| USA Policy | interaction | 34 | Neutral |
| USA Policy | introduced | 34 | Neutral |
| USA Policy | market | 34 | Neutral |
| USA Policy | needs | 34 | Neutral |
| USA Policy | nmfs | 34 | Neutral |
| USA Policy | ocs | 34 | Neutral |
| USA Policy | oyster | 34 | Neutral |
| USA Policy | provision | 34 | Neutral |
| USA Policy | represent | 34 | Neutral |
| USA Policy | salmonid | 34 | Neutral |
| USA Policy | susan | 34 | Neutral |
| USA Policy | challenges | 33 | Neutral |
| USA Policy | large | 33 | Neutral |
| USA Policy | law | 33 | Neutral |
| USA Policy | march | 33 | Neutral |
| USA Policy | minimize | 33 | Neutral |
| USA Policy | plastic | 33 | Neutral |
| USA Policy | alternative | 32 | Neutral |
| USA Policy | consumption | 32 | Neutral |
| USA Policy | demonstrate | 32 | Neutral |
| USA Policy | introduction | 32 | Neutral |
| USA Policy | nutrient | 32 | Neutral |
| USA Policy | system | 32 | Neutral |
| USA Policy | value | 32 | Neutral |
| USA Policy | agriculture | 31 | Neutral |
| USA Policy | culture | 31 | Neutral |
| USA Policy | finfish | 31 | Neutral |
| USA Policy | found | 31 | Neutral |
| USA Policy | game | 31 | Neutral |
| USA Policy | identified | 31 | Neutral |
| USA Policy | jurisdiction | 31 | Neutral |
| USA Policy | necessary | 31 | Neutral |
| USA Policy | needed | 31 | Neutral |
| USA Policy | often | 31 | Neutral |
| USA Policy | pelagic | 31 | Neutral |
| USA Policy | producer | 31 | Neutral |
| USA Policy | avoid | 30 | Neutral |
| USA Policy | consumers | 30 | Neutral |
| USA Policy | countries | 30 | Neutral |
| USA Policy | essential | 30 | Neutral |
| USA Policy | fact | 30 | Neutral |
| USA Policy | february | 30 | Neutral |
| USA Policy | known | 30 | Neutral |
| USA Policy | least | 30 | Neutral |
| USA Policy | part | 30 | Neutral |
| USA Policy | proposals | 30 | Neutral |
| USA Policy | simply | 30 | Neutral |
| USA Policy | technical | 30 | Neutral |
| USA Policy | american | 29 | Neutral |
| USA Policy | continue | 29 | Neutral |
| USA Policy | create | 29 | Neutral |
| USA Policy | economy | 29 | Neutral |
| USA Policy | highway | 29 | Neutral |
| USA Policy | importance | 29 | Neutral |
| USA Policy | long | 29 | Neutral |
| USA Policy | possible | 29 | Neutral |
| USA Policy | predator | 29 | Neutral |
| USA Policy | protected | 29 | Neutral |
| USA Policy | recreational | 29 | Neutral |
| USA Policy | release | 29 | Neutral |
| USA Policy | studies | 29 | Neutral |
| USA Policy | university | 29 | Neutral |
| USA Policy | advance | 28 | Neutral |
| USA Policy | bivalve | 28 | Neutral |
| USA Policy | capture | 28 | Neutral |
| USA Policy | either | 28 | Neutral |
| USA Policy | evidence | 28 | Neutral |
| USA Policy | farmers | 28 | Neutral |
| USA Policy | land | 28 | Neutral |
| USA Policy | laws | 28 | Neutral |
| USA Policy | less | 28 | Neutral |
| USA Policy | list | 28 | Neutral |
| USA Policy | living | 28 | Neutral |
| USA Policy | organisms | 28 | Neutral |
| USA Policy | pcsga | 28 | Neutral |
| USA Policy | proposal | 28 | Neutral |
| USA Policy | protecting | 28 | Neutral |
| USA Policy | reduction | 28 | Neutral |
| USA Policy | second | 28 | Neutral |
| USA Policy | therefore | 28 | Neutral |
| USA Policy | thus | 28 | Neutral |
| USA Policy | british_columbia | 27 | Neutral |
| USA Policy | canada | 27 | Neutral |
| USA Policy | conducted | 27 | Neutral |
| USA Policy | executive | 27 | Neutral |
| USA Policy | fishmeal | 27 | Neutral |
| USA Policy | floor | 27 | Neutral |
| USA Policy | geoduck | 27 | Neutral |
| USA Policy | implications | 27 | Neutral |
| USA Policy | letter | 27 | Neutral |
| USA Policy | measures | 27 | Neutral |
| USA Policy | potentially | 27 | Neutral |
| USA Policy | security | 27 | Neutral |
| USA Policy | approved | 26 | Neutral |
| USA Policy | cumulative | 26 | Neutral |
| USA Policy | extent | 26 | Neutral |
| USA Policy | grow | 26 | Neutral |
| USA Policy | guide | 26 | Neutral |
| USA Policy | lands | 26 | Neutral |
| USA Policy | longterm | 26 | Neutral |
| USA Policy | meal | 26 | Neutral |
| USA Policy | nature | 26 | Neutral |
| USA Policy | place | 26 | Neutral |
| USA Policy | raising | 26 | Neutral |
| USA Policy | released | 26 | Neutral |
| USA Policy | salmo | 26 | Neutral |
| USA Policy | sources | 26 | Neutral |
| USA Policy | strongly | 26 | Neutral |
| USA Policy | understanding | 26 | Neutral |
| USA Policy | board | 25 | Neutral |
| USA Policy | direct | 25 | Neutral |
| USA Policy | eastwest | 25 | Neutral |
| USA Policy | expand | 25 | Neutral |
| USA Policy | fax | 25 | Neutral |
| USA Policy | goldburg | 25 | Neutral |
| USA Policy | intertidal | 25 | Neutral |
| USA Policy | life | 25 | Neutral |
| USA Policy | little | 25 | Neutral |
| USA Policy | managed | 25 | Neutral |
| USA Policy | move | 25 | Neutral |
| USA Policy | precautionary | 25 | Neutral |
| USA Policy | prey | 25 | Neutral |
| USA Policy | promote | 25 | Neutral |
| USA Policy | salar | 25 | Neutral |
| USA Policy | seem | 25 | Neutral |
| USA Policy | spread | 25 | Neutral |
| USA Policy | west | 25 | Neutral |
| USA Policy | animals | 24 | Neutral |
| USA Policy | appears | 24 | Neutral |
| USA Policy | believes | 24 | Neutral |
| USA Policy | bullet | 24 | Neutral |
| USA Policy | cultured | 24 | Neutral |
| USA Policy | exclusive | 24 | Neutral |
| USA Policy | fully | 24 | Neutral |
| USA Policy | mafac | 24 | Neutral |
| USA Policy | regions | 24 | Neutral |
| USA Policy | small | 24 | Neutral |
| USA Policy | substantial | 24 | Neutral |
| USA Policy | achieve | 23 | Neutral |
| USA Policy | added | 23 | Neutral |
| USA Policy | boundaries | 23 | Neutral |
| USA Policy | changes | 23 | Neutral |
| USA Policy | community | 23 | Neutral |
| USA Policy | continued | 23 | Neutral |
| USA Policy | described | 23 | Neutral |
| USA Policy | epa | 23 | Neutral |
| USA Policy | experience | 23 | Neutral |
| USA Policy | factors | 23 | Neutral |
| USA Policy | feeding | 23 | Neutral |
| USA Policy | gas | 23 | Neutral |
| USA Policy | higher | 23 | Neutral |
| USA Policy | ofthe | 23 | Neutral |
| USA Policy | past | 23 | Neutral |
| USA Policy | primary | 23 | Neutral |
| USA Policy | reports | 23 | Neutral |
| USA Policy | sentence | 23 | Neutral |
| USA Policy | source | 23 | Neutral |
| USA Policy | various | 23 | Neutral |
| USA Policy | certain | 22 | Neutral |
| USA Policy | corps | 22 | Neutral |
| USA Policy | criteria | 22 | Neutral |
| USA Policy | december | 22 | Neutral |
| USA Policy | different | 22 | Neutral |
| USA Policy | discussion | 22 | Neutral |
| USA Policy | figure | 22 | Neutral |
| USA Policy | growers | 22 | Neutral |
| USA Policy | knowledge | 22 | Neutral |
| USA Policy | making | 22 | Neutral |
| USA Policy | office | 22 | Neutral |
| USA Policy | operating | 22 | Neutral |
| USA Policy | president | 22 | Neutral |
| USA Policy | programmatic | 22 | Neutral |
| USA Policy | reflect | 22 | Neutral |
| USA Policy | set | 22 | Neutral |
| USA Policy | socioeconomic | 22 | Neutral |
| USA Policy | stewardship | 22 | Neutral |
| USA Policy | supplies | 22 | Neutral |
| USA Policy | united | 22 | Neutral |
| USA Policy | usda | 22 | Neutral |
| USA Policy | alternate | 21 | Neutral |
| USA Policy | another | 21 | Neutral |
| USA Policy | business | 21 | Neutral |
| USA Policy | conduct | 21 | Neutral |
| USA Policy | decision | 21 | Neutral |
| USA Policy | definition | 21 | Neutral |
| USA Policy | directly | 21 | Neutral |
| USA Policy | esa | 21 | Neutral |
| USA Policy | especially | 21 | Neutral |
| USA Policy | generally | 21 | Neutral |
| USA Policy | hawaiian | 21 | Neutral |
| USA Policy | identify | 21 | Neutral |
| USA Policy | journal | 21 | Neutral |
| USA Policy | largest | 21 | Neutral |
| USA Policy | mammals | 21 | Neutral |
| USA Policy | managing | 21 | Neutral |
| USA Policy | mms | 21 | Neutral |
| USA Policy | note | 21 | Neutral |
| USA Policy | openocean | 21 | Neutral |
| USA Policy | operators | 21 | Neutral |
| USA Policy | present | 21 | Neutral |
| USA Policy | relevant | 21 | Neutral |
| USA Policy | though | 21 | Neutral |
| USA Policy | upon | 21 | Neutral |
| USA Policy | annual | 20 | Neutral |
| USA Policy | application | 20 | Neutral |
| USA Policy | approximately | 20 | Neutral |
| USA Policy | birds | 20 | Neutral |
| USA Policy | chair | 20 | Neutral |
| USA Policy | determined | 20 | Neutral |
| USA Policy | division | 20 | Neutral |
| USA Policy | enable | 20 | Neutral |
| USA Policy | infectious | 20 | Neutral |
| USA Policy | lubchenco | 20 | Neutral |
| USA Policy | maintain | 20 | Neutral |
| USA Policy | norway | 20 | Neutral |
| USA Policy | oysters | 20 | Neutral |
| USA Policy | raised | 20 | Neutral |
| USA Policy | researchers | 20 | Neutral |
| USA Policy | sand | 20 | Neutral |
| USA Policy | scientists | 20 | Neutral |
| USA Policy | short | 20 | Neutral |
| USA Policy | spatial | 20 | Neutral |
| USA Policy | spawning | 20 | Neutral |
| USA Policy | speci | 20 | Neutral |
| USA Policy | technological | 20 | Neutral |
| USA Policy | total | 20 | Neutral |
| USA Policy | yet | 20 | Neutral |
| USA Policy | urge | 20 | Neutral |
| USA Policy | appear | 19 | Neutral |
| USA Policy | array | 19 | Neutral |
| USA Policy | basis | 19 | Neutral |
| USA Policy | become | 19 | Neutral |
| USA Policy | chinook | 19 | Neutral |
| USA Policy | consistency | 19 | Neutral |
| USA Policy | defined | 19 | Neutral |
| USA Policy | despite | 19 | Neutral |
| USA Policy | freshwater | 19 | Neutral |
| USA Policy | highly | 19 | Neutral |
| USA Policy | immediate | 19 | Neutral |
| USA Policy | january | 19 | Neutral |
| USA Policy | objectives | 19 | Neutral |
| USA Policy | offshor | 19 | Neutral |
| USA Policy | oper | 19 | Neutral |
| USA Policy | play | 19 | Neutral |
| USA Policy | question | 19 | Neutral |
| USA Policy | removal | 19 | Neutral |
| USA Policy | safeguard | 19 | Neutral |
| USA Policy | safety | 19 | Neutral |
| USA Policy | shore | 19 | Neutral |
| USA Policy | sound | 19 | Neutral |
| USA Policy | submit | 19 | Neutral |
| USA Policy | throughout | 19 | Neutral |
| USA Policy | tons | 19 | Neutral |
| USA Policy | via | 19 | Neutral |
| USA Policy | volpe | 19 | Neutral |
| USA Policy | wastes | 19 | Neutral |
| USA Policy | wat | 19 | Neutral |
| USA Policy | authorities | 18 | Neutral |
| USA Policy | billion | 18 | Neutral |
| USA Policy | burke | 18 | Neutral |
| USA Policy | consultation | 18 | Neutral |
| USA Policy | discuss | 18 | Neutral |
| USA Policy | documents | 18 | Neutral |
| USA Policy | drugs | 18 | Neutral |
| USA Policy | emerging | 18 | Neutral |
| USA Policy | export | 18 | Neutral |
| USA Policy | fishes | 18 | Neutral |
| USA Policy | grant | 18 | Neutral |
| USA Policy | held | 18 | Neutral |
| USA Policy | inc | 18 | Neutral |
| USA Policy | individual | 18 | Neutral |
| USA Policy | look | 18 | Neutral |
| USA Policy | maf | 18 | Neutral |
| USA Policy | mandate | 18 | Neutral |
| USA Policy | methods | 18 | Neutral |
| USA Policy | nfi | 18 | Neutral |
| USA Policy | north | 18 | Neutral |
| USA Policy | organic | 18 | Neutral |
| USA Policy | outreach | 18 | Neutral |
| USA Policy | pen | 18 | Neutral |
| USA Policy | prepared | 18 | Neutral |
| USA Policy | put | 18 | Neutral |
| USA Policy | questions | 18 | Neutral |
| USA Policy | sense | 18 | Neutral |
| USA Policy | sincer | 18 | Neutral |
| USA Policy | sustain | 18 | Neutral |
| USA Policy | tuna | 18 | Neutral |
| USA Policy | whole | 18 | Neutral |
| USA Policy | authorized | 17 | Neutral |
| USA Policy | beds | 17 | Neutral |
| USA Policy | climate | 17 | Neutral |
| USA Policy | compliance | 17 | Neutral |
| USA Policy | components | 17 | Neutral |
| USA Policy | contact | 17 | Neutral |
| USA Policy | debris | 17 | Neutral |
| USA Policy | determine | 17 | Neutral |
| USA Policy | ensuring | 17 | Neutral |
| USA Policy | expected | 17 | Neutral |
| USA Policy | far | 17 | Neutral |
| USA Policy | form | 17 | Neutral |
| USA Policy | imported | 17 | Neutral |
| USA Policy | input | 17 | Neutral |
| USA Policy | krkosek | 17 | Neutral |
| USA Policy | legal | 17 | Neutral |
| USA Policy | low | 17 | Neutral |
| USA Policy | manager | 17 | Neutral |
| USA Policy | meeting | 17 | Neutral |
| USA Policy | multiple | 17 | Neutral |
| USA Policy | noa | 17 | Neutral |
| USA Policy | others | 17 | Neutral |
| USA Policy | perhaps | 17 | Neutral |
| USA Policy | prior | 17 | Neutral |
| USA Policy | stewards | 17 | Neutral |
| USA Policy | stocking | 17 | Neutral |
| USA Policy | structures | 17 | Neutral |
| USA Policy | surrounding | 17 | Neutral |
| USA Policy | worldwide | 17 | Neutral |
| USA Policy | america | 16 | Neutral |
| USA Policy | assess | 16 | Neutral |
| USA Policy | base | 16 | Neutral |
| USA Policy | beyond | 16 | Neutral |
| USA Policy | bill | 16 | Neutral |
| USA Policy | bluefin | 16 | Neutral |
| USA Policy | citizen | 16 | Neutral |
| USA Policy | commun | 16 | Neutral |
| USA Policy | component | 16 | Neutral |
| USA Policy | course | 16 | Neutral |
| USA Policy | degree | 16 | Neutral |
| USA Policy | designated | 16 | Neutral |
| USA Policy | early | 16 | Neutral |
| USA Policy | etc | 16 | Neutral |
| USA Policy | extensive | 16 | Neutral |
| USA Policy | findings | 16 | Neutral |
| USA Policy | follow | 16 | Neutral |
| USA Policy | genetically_modified | 16 | Neutral |
| USA Policy | hswri | 16 | Neutral |
| USA Policy | infrastructure | 16 | Neutral |
| USA Policy | intended | 16 | Neutral |
| USA Policy | interim | 16 | Neutral |
| USA Policy | issued | 16 | Neutral |
| USA Policy | june | 16 | Neutral |
| USA Policy | key | 16 | Neutral |
| USA Policy | later | 16 | Neutral |
| USA Policy | leadership | 16 | Neutral |
| USA Policy | model | 16 | Neutral |
| USA Policy | overall | 16 | Neutral |
| USA Policy | pacilic | 16 | Neutral |
| USA Policy | pathogens | 16 | Neutral |
| USA Policy | plant | 16 | Neutral |
| USA Policy | pollutants | 16 | Neutral |
| USA Policy | presentation | 16 | Neutral |
| USA Policy | previous | 16 | Neutral |
| USA Policy | proceed | 16 | Neutral |
| USA Policy | published | 16 | Neutral |
| USA Policy | range | 16 | Neutral |
| USA Policy | remain | 16 | Neutral |
| USA Policy | reporting | 16 | Neutral |
| USA Policy | restore | 16 | Neutral |
| USA Policy | shelf | 16 | Neutral |
| USA Policy | southern | 16 | Neutral |
| USA Policy | special | 16 | Neutral |
| USA Policy | status | 16 | Neutral |
| USA Policy | trout | 16 | Neutral |
| USA Policy | using | 16 | Neutral |
| USA Policy | vision | 16 | Neutral |
| USA Policy | vital | 16 | Neutral |
| USA Policy | web | 16 | Neutral |
| USA Policy | whale | 16 | Neutral |
| USA Policy | able | 15 | Neutral |
| USA Policy | acknowledge | 15 | Neutral |
| USA Policy | actually | 15 | Neutral |
| USA Policy | adjacent | 15 | Neutral |
| USA Policy | although | 15 | Neutral |
| USA Policy | applied | 15 | Neutral |
| USA Policy | aquarium | 15 | Neutral |
| USA Policy | army | 15 | Neutral |
| USA Policy | avenue | 15 | Neutral |
| USA Policy | background | 15 | Neutral |
| USA Policy | broad | 15 | Neutral |
| USA Policy | chile | 15 | Neutral |
| USA Policy | come | 15 | Neutral |
| USA Policy | company | 15 | Neutral |
| USA Policy | consequence | 15 | Neutral |
| USA Policy | consumer | 15 | Neutral |
| USA Policy | continues | 15 | Neutral |
| USA Policy | coordinate | 15 | Neutral |
| USA Policy | define | 15 | Neutral |
| USA Policy | demonstration | 15 | Neutral |
| USA Policy | density | 15 | Neutral |
| USA Policy | diego | 15 | Neutral |
| USA Policy | diet | 15 | Neutral |
| USA Policy | ecology | 15 | Neutral |
| USA Policy | emphasis | 15 | Neutral |
| USA Policy | focused | 15 | Neutral |
| USA Policy | foreign | 15 | Neutral |
| USA Policy | four | 15 | Neutral |
| USA Policy | funding | 15 | Neutral |
| USA Policy | furthermore | 15 | Neutral |
| USA Policy | ingredients | 15 | Neutral |
| USA Policy | magnusonstevens | 15 | Neutral |
| USA Policy | manage | 15 | Neutral |
| USA Policy | mean | 15 | Neutral |
| USA Policy | means | 15 | Neutral |
| USA Policy | mentioned | 15 | Neutral |
| USA Policy | michael | 15 | Neutral |
| USA Policy | northwest | 15 | Neutral |
| USA Policy | numerous | 15 | Neutral |
| USA Policy | oregon | 15 | Neutral |
| USA Policy | partner | 15 | Neutral |
| USA Policy | pdf | 15 | Neutral |
| USA Policy | promotional | 15 | Neutral |
| USA Policy | rapidly | 15 | Neutral |
| USA Policy | river | 15 | Neutral |
| USA Policy | room | 15 | Neutral |
| USA Policy | rulemaking | 15 | Neutral |
| USA Policy | scotland | 15 | Neutral |
| USA Policy | sections | 15 | Neutral |
| USA Policy | size | 15 | Neutral |
| USA Policy | soybean | 15 | Neutral |
| USA Policy | structure | 15 | Neutral |
| USA Policy | submerged | 15 | Neutral |
| USA Policy | toward | 15 | Neutral |
| USA Policy | turn | 15 | Neutral |
| USA Policy | unit | 15 | Neutral |
| USA Policy | urgency | 15 | Neutral |
| USA Policy | americans | 14 | Neutral |
| USA Policy | andor | 14 | Neutral |
| USA Policy | apply | 14 | Neutral |
| USA Policy | associ | 14 | Neutral |
| USA Policy | capacity | 14 | Neutral |
| USA Policy | caught | 14 | Neutral |
| USA Policy | clarification | 14 | Neutral |
| USA Policy | collaboration | 14 | Neutral |
| USA Policy | communication | 14 | Neutral |
| USA Policy | companies | 14 | Neutral |
| USA Policy | context | 14 | Neutral |
| USA Policy | coordination | 14 | Neutral |
| USA Policy | decommissioning | 14 | Neutral |
| USA Policy | diets | 14 | Neutral |
| USA Policy | diversity | 14 | Neutral |
| USA Policy | drug | 14 | Neutral |
| USA Policy | eliminate | 14 | Neutral |
| USA Policy | emission | 14 | Neutral |
| USA Policy | engineer | 14 | Neutral |
| USA Policy | estuaries | 14 | Neutral |
| USA Policy | experts | 14 | Neutral |
| USA Policy | extension | 14 | Neutral |
| USA Policy | finalized | 14 | Neutral |
| USA Policy | grown | 14 | Neutral |
| USA Policy | haaa | 14 | Neutral |
| USA Policy | ices | 14 | Neutral |
| USA Policy | indicate | 14 | Neutral |
| USA Policy | interior | 14 | Neutral |
| USA Policy | isa | 14 | Neutral |
| USA Policy | main | 14 | Neutral |
| USA Policy | mission | 14 | Neutral |
| USA Policy | mitigation | 14 | Neutral |
| USA Policy | netpen | 14 | Neutral |
| USA Policy | offer | 14 | Neutral |
| USA Policy | outer | 14 | Neutral |
| USA Policy | penttila | 14 | Neutral |
| USA Policy | percent | 14 | Neutral |
| USA Policy | press | 14 | Neutral |
| USA Policy | relatively | 14 | Neutral |
| USA Policy | request | 14 | Neutral |
| USA Policy | responsibility | 14 | Neutral |
| USA Policy | sardine | 14 | Neutral |
| USA Policy | seq | 14 | Neutral |
| USA Policy | sited | 14 | Neutral |
| USA Policy | steps | 14 | Neutral |
| USA Policy | submit | 14 | Neutral |
| USA Policy | suite | 14 | Neutral |
| USA Policy | terrestrial | 14 | Neutral |
| USA Policy | threatened | 14 | Neutral |
| USA Policy | transmission | 14 | Neutral |
| USA Policy | view | 14 | Neutral |
| USA Policy | whale | 14 | Neutral |
| USA Policy | attempt | 13 | Neutral |
| USA Policy | behalf | 13 | Neutral |
| USA Policy | broader | 13 | Neutral |
| USA Policy | carbaryl | 13 | Neutral |
| USA Policy | coalition | 13 | Neutral |
| USA Policy | conducting | 13 | Neutral |
| USA Policy | cultivation | 13 | Neutral |
| USA Policy | deficit | 13 | Neutral |
| USA Policy | distribution | 13 | Neutral |
| USA Policy | domest | 13 | Neutral |
| USA Policy | east | 13 | Neutral |
| USA Policy | enforce | 13 | Neutral |
| USA Policy | evaluate | 13 | Neutral |
| USA Policy | expertise | 13 | Neutral |
| USA Policy | fleming | 13 | Neutral |
| USA Policy | foundation | 13 | Neutral |
| USA Policy | gaps | 13 | Neutral |
| USA Policy | governor | 13 | Neutral |
| USA Policy | hand | 13 | Neutral |
| USA Policy | influence | 13 | Neutral |
| USA Policy | instead | 13 | Neutral |
| USA Policy | issuance | 13 | Neutral |
| USA Policy | know | 13 | Neutral |
| USA Policy | located | 13 | Neutral |
| USA Policy | msa | 13 | Neutral |
| USA Policy | nets | 13 | Neutral |
| USA Policy | noted | 13 | Neutral |
| USA Policy | notes | 13 | Neutral |
| USA Policy | november | 13 | Neutral |
| USA Policy | npdes | 13 | Neutral |
| USA Policy | numbers | 13 | Neutral |
| USA Policy | operate | 13 | Neutral |
| USA Policy | options | 13 | Neutral |
| USA Policy | outside | 13 | Neutral |
| USA Policy | paper | 13 | Neutral |
| USA Policy | partnership | 13 | Neutral |
| USA Policy | people | 13 | Neutral |
| USA Policy | physical | 13 | Neutral |
| USA Policy | prepare | 13 | Neutral |
| USA Policy | protein | 13 | Neutral |
| USA Policy | protocols | 13 | Neutral |
| USA Policy | regular | 13 | Neutral |
| USA Policy | responsibilities | 13 | Neutral |
| USA Policy | separate | 13 | Neutral |
| USA Policy | september | 13 | Neutral |
| USA Policy | serve | 13 | Neutral |
| USA Policy | transfer | 13 | Neutral |
| USA Policy | version | 13 | Neutral |
| USA Policy | viable | 13 | Neutral |
| USA Policy | yes | 13 | Neutral |
| USA Policy | zooplankton | 13 | Neutral |
| USA Policy | asbs | 12 | Neutral |
| USA Policy | aspect | 12 | Neutral |
| USA Policy | assist | 12 | Neutral |
| USA Policy | august | 12 | Neutral |
| USA Policy | aware | 12 | Neutral |
| USA Policy | balance | 12 | Neutral |
| USA Policy | biology | 12 | Neutral |
| USA Policy | blue | 12 | Neutral |
| USA Policy | body | 12 | Neutral |
| USA Policy | bush | 12 | Neutral |
| USA Policy | carnivorous | 12 | Neutral |
| USA Policy | central | 12 | Neutral |
| USA Policy | club | 12 | Neutral |
| USA Policy | com | 12 | Neutral |
| USA Policy | conservancy | 12 | Neutral |
| USA Policy | densities | 12 | Neutral |
| USA Policy | designed | 12 | Neutral |
| USA Policy | eelgrass | 12 | Neutral |
| USA Policy | email | 12 | Neutral |
| USA Policy | every | 12 | Neutral |
| USA Policy | experimental | 12 | Neutral |
| USA Policy | herring | 12 | Neutral |
| USA Policy | highest | 12 | Neutral |
| USA Policy | importantly | 12 | Neutral |
| USA Policy | initial | 12 | Neutral |
| USA Policy | intensive | 12 | Neutral |
| USA Policy | interbreeding | 12 | Neutral |
| USA Policy | invest | 12 | Neutral |
| USA Policy | john | 12 | Neutral |
| USA Policy | lance | 12 | Neutral |
| USA Policy | larger | 12 | Neutral |
| USA Policy | largescale | 12 | Neutral |
| USA Policy | location | 12 | Neutral |
| USA Policy | maintenance | 12 | Neutral |
| USA Policy | matter | 12 | Neutral |
| USA Policy | metric | 12 | Neutral |
| USA Policy | misinformation | 12 | Neutral |
| USA Policy | morton | 12 | Neutral |
| USA Policy | mussel | 12 | Neutral |
| USA Policy | organization | 12 | Neutral |
| USA Policy | otherwise | 12 | Neutral |
| USA Policy | oversea | 12 | Neutral |
| USA Policy | personal | 12 | Neutral |
| USA Policy | position | 12 | Neutral |
| USA Policy | preliminary | 12 | Neutral |
| USA Policy | prices | 12 | Neutral |
| USA Policy | reducing | 12 | Neutral |
| USA Policy | regulators | 12 | Neutral |
| USA Policy | relative | 12 | Neutral |
| USA Policy | rely | 12 | Neutral |
| USA Policy | remains | 12 | Neutral |
| USA Policy | remove | 12 | Neutral |
| USA Policy | representatives | 12 | Neutral |
| USA Policy | rubino | 12 | Neutral |
| USA Policy | sciencebase | 12 | Neutral |
| USA Policy | sediment | 12 | Neutral |
| USA Policy | series | 12 | Neutral |
| USA Policy | share | 12 | Neutral |
| USA Policy | shown | 12 | Neutral |
| USA Policy | shrimp | 12 | Neutral |
| USA Policy | sierra | 12 | Neutral |
| USA Policy | six | 12 | Neutral |
| USA Policy | southeast | 12 | Neutral |
| USA Policy | space | 12 | Neutral |
| USA Policy | strategic | 12 | Neutral |
| USA Policy | team | 12 | Neutral |
| USA Policy | unless | 12 | Neutral |
| USA Policy | venoco | 12 | Neutral |
| USA Policy | ways | 12 | Neutral |
| USA Policy | week | 12 | Neutral |
| USA Policy | wwf | 12 | Neutral |
| USA Policy | amended | 11 | Neutral |
| USA Policy | america | 11 | Neutral |
| USA Policy | applicable | 11 | Neutral |
| USA Policy | approaches | 11 | Neutral |
| USA Policy | apr | 11 | Neutral |
| USA Policy | assessments | 11 | Neutral |
| USA Policy | begin | 11 | Neutral |
| USA Policy | challenge | 11 | Neutral |
| USA Policy | chapter | 11 | Neutral |
| USA Policy | charting | 11 | Neutral |
| USA Policy | combined | 11 | Neutral |
| USA Policy | complete | 11 | Neutral |
| USA Policy | completed | 11 | Neutral |
| USA Policy | constitution | 11 | Neutral |
| USA Policy | consum | 11 | Neutral |
| USA Policy | consumed | 11 | Neutral |
| USA Policy | controlled | 11 | Neutral |
| USA Policy | conversion | 11 | Neutral |
| USA Policy | darden | 11 | Neutral |
| USA Policy | depend | 11 | Neutral |
| USA Policy | detail | 11 | Neutral |
| USA Policy | enforcement | 11 | Neutral |
| USA Policy | engage | 11 | Neutral |
| USA Policy | engineered | 11 | Neutral |
| USA Policy | extend | 11 | Neutral |
| USA Policy | fda | 11 | Neutral |
| USA Policy | feces | 11 | Neutral |
| USA Policy | fed | 11 | Neutral |
| USA Policy | fgc | 11 | Neutral |
| USA Policy | five | 11 | Neutral |
| USA Policy | forms | 11 | Neutral |
| USA Policy | greatly | 11 | Neutral |
| USA Policy | independent | 11 | Neutral |
| USA Policy | jane | 11 | Neutral |
| USA Policy | joint | 11 | Neutral |
| USA Policy | july | 11 | Neutral |
| USA Policy | largely | 11 | Neutral |
| USA Policy | larvae | 11 | Neutral |
| USA Policy | lessee | 11 | Neutral |
| USA Policy | line | 11 | Neutral |
| USA Policy | locally | 11 | Neutral |
| USA Policy | marin | 11 | Neutral |
| USA Policy | monitor | 11 | Neutral |
| USA Policy | organizations | 11 | Neutral |
| USA Policy | overview | 11 | Neutral |
| USA Policy | partnerships | 11 | Neutral |
| USA Policy | path | 11 | Neutral |
| USA Policy | peir | 11 | Neutral |
| USA Policy | phone | 11 | Neutral |
| USA Policy | presented | 11 | Neutral |
| USA Policy | proceedings | 11 | Neutral |
| USA Policy | prohibition | 11 | Neutral |
| USA Policy | protections | 11 | Neutral |
| USA Policy | rates | 11 | Neutral |
| USA Policy | rearing | 11 | Neutral |
| USA Policy | recently | 11 | Neutral |
| USA Policy | renewable | 11 | Neutral |
| USA Policy | response | 11 | Neutral |
| USA Policy | revised | 11 | Neutral |
| USA Policy | rhe | 11 | Neutral |
| USA Policy | sci | 11 | Neutral |
| USA Policy | scottish | 11 | Neutral |
| USA Policy | sediment | 11 | Neutral |
| USA Policy | show | 11 | Neutral |
| USA Policy | society | 11 | Neutral |
| USA Policy | south | 11 | Neutral |
| USA Policy | spawn | 11 | Neutral |
| USA Policy | subsequent | 11 | Neutral |
| USA Policy | taylor | 11 | Neutral |
| USA Policy | ten | 11 | Neutral |
| USA Policy | tigers | 11 | Neutral |
| USA Policy | types | 11 | Neutral |
| USA Policy | usdoc | 11 | Neutral |
| USA Policy | wdfw | 11 | Neutral |
| USA Policy | workshop | 11 | Neutral |
| USA Policy | account | 10 | Neutral |
| USA Policy | acoustic | 10 | Neutral |
| USA Policy | ahds | 10 | Neutral |
| USA Policy | altern | 10 | Neutral |
| USA Policy | amendment | 10 | Neutral |
| USA Policy | amount | 10 | Neutral |
| USA Policy | antibiotic | 10 | Neutral |
| USA Policy | applications | 10 | Neutral |
| USA Policy | attention | 10 | Neutral |
| USA Policy | attn | 10 | Neutral |
| USA Policy | author | 10 | Neutral |
| USA Policy | basic | 10 | Neutral |
| USA Policy | bass | 10 | Neutral |
| USA Policy | bring | 10 | Neutral |
| USA Policy | carbon | 10 | Neutral |
| USA Policy | catch | 10 | Neutral |
| USA Policy | clams | 10 | Neutral |
| USA Policy | clarify | 10 | Neutral |
| USA Policy | cmsp | 10 | Neutral |
| USA Policy | cod | 10 | Neutral |
| USA Policy | composition | 10 | Neutral |
| USA Policy | conclusion | 10 | Neutral |
| USA Policy | continental | 10 | Neutral |
| USA Policy | coordinate | 10 | Neutral |
| USA Policy | county | 10 | Neutral |
| USA Policy | defense | 10 | Neutral |
| USA Policy | derived | 10 | Neutral |
| USA Policy | dietary | 10 | Neutral |
| USA Policy | direction | 10 | Neutral |
| USA Policy | diverse | 10 | Neutral |
| USA Policy | dnr | 10 | Neutral |
| USA Policy | dollars | 10 | Neutral |
| USA Policy | earlier | 10 | Neutral |
| USA Policy | efficiency | 10 | Neutral |
| USA Policy | england | 10 | Neutral |
| USA Policy | estuary | 10 | Neutral |
| USA Policy | feasibility | 10 | Neutral |
| USA Policy | financial | 10 | Neutral |
| USA Policy | force | 10 | Neutral |
| USA Policy | foster | 10 | Neutral |
| USA Policy | functions | 10 | Neutral |
| USA Policy | fundamental | 10 | Neutral |
| USA Policy | funds | 10 | Neutral |
| USA Policy | hearing | 10 | Neutral |
| USA Policy | hindar | 10 | Neutral |
| USA Policy | hope | 10 | Neutral |
| USA Policy | humpback | 10 | Neutral |
| USA Policy | hydrocarbon | 10 | Neutral |
| USA Policy | indigenous | 10 | Neutral |
| USA Policy | inlet | 10 | Neutral |
| USA Policy | insects | 10 | Neutral |
| USA Policy | items | 10 | Neutral |
| USA Policy | johnson | 10 | Neutral |
| USA Policy | juveniles | 10 | Neutral |
| USA Policy | kona | 10 | Neutral |
| USA Policy | locke | 10 | Neutral |
| USA Policy | lower | 10 | Neutral |
| USA Policy | maintaining | 10 | Neutral |
| USA Policy | makes | 10 | Neutral |
| USA Policy | markets | 10 | Neutral |
| USA Policy | material | 10 | Neutral |
| USA Policy | mechanism | 10 | Neutral |
| USA Policy | miles | 10 | Neutral |
| USA Policy | mitigate | 10 | Neutral |
| USA Policy | moore | 10 | Neutral |
| USA Policy | nearly | 10 | Neutral |
| USA Policy | never | 10 | Neutral |
| USA Policy | olympia | 10 | Neutral |
| USA Policy | overarching | 10 | Neutral |
| USA Policy | real | 10 | Neutral |
| USA Policy | reason | 10 | Neutral |
| USA Policy | rec | 10 | Neutral |
| USA Policy | record | 10 | Neutral |
| USA Policy | reliance | 10 | Neutral |
| USA Policy | reported | 10 | Neutral |
| USA Policy | rivers | 10 | Neutral |
| USA Policy | shows | 10 | Neutral |
| USA Policy | third | 10 | Neutral |
| USA Policy | title | 10 | Neutral |
| USA Policy | together | 10 | Neutral |
| USA Policy | tools | 10 | Neutral |
| USA Policy | turtles | 10 | Neutral |
| USA Policy | unique | 10 | Neutral |
| USA Policy | usa | 10 | Neutral |
| USA Policy | usace | 10 | Neutral |
| USA Policy | vessel | 10 | Neutral |
| USA Policy | weight | 10 | Neutral |
| USA Policy | seafood | 278 | Positive |
| USA Policy | sanctuary | 241 | Positive |
| USA Policy | sustainable | 215 | Positive |
| USA Policy | support | 206 | Positive |
| USA Policy | significant | 154 | Positive |
| USA Policy | food | 142 | Positive |
| USA Policy | recommend | 125 | Positive |
| USA Policy | available | 110 | Positive |
| USA Policy | important | 97 | Positive |
| USA Policy | work | 85 | Positive |
| USA Policy | natural | 83 | Positive |
| USA Policy | encourage | 80 | Positive |
| USA Policy | protection | 76 | Positive |
| USA Policy | benefit | 68 | Positive |
| USA Policy | best | 63 | Positive |
| USA Policy | clear | 63 | Positive |
| USA Policy | protect | 61 | Positive |
| USA Policy | comprehensive | 60 | Positive |
| USA Policy | interest | 57 | Positive |
| USA Policy | lead | 57 | Positive |
| USA Policy | appropriate | 54 | Positive |
| USA Policy | healthy | 53 | Positive |
| USA Policy | grace | 46 | Positive |
| USA Policy | respect | 45 | Positive |
| USA Policy | strong | 41 | Positive |
| USA Policy | thank | 39 | Positive |
| USA Policy | clearly | 37 | Positive |
| USA Policy | consistent | 37 | Positive |
| USA Policy | adequate | 36 | Positive |
| USA Policy | guidance | 36 | Positive |
| USA Policy | success | 28 | Positive |
| USA Policy | better | 27 | Positive |
| USA Policy | sufficient | 26 | Positive |
| USA Policy | innovation | 22 | Positive |
| USA Policy | safe | 22 | Positive |
| USA Policy | recovery | 21 | Positive |
| USA Policy | enhancement | 19 | Positive |
| USA Policy | productive | 19 | Positive |
| USA Policy | approval | 18 | Positive |
| USA Policy | competitive | 18 | Positive |
| USA Policy | enhance | 18 | Positive |
| USA Policy | facilitate | 18 | Positive |
| USA Policy | progress | 18 | Positive |
| USA Policy | sensitive | 18 | Positive |
| USA Policy | clean | 17 | Positive |
| USA Policy | efficient | 17 | Positive |
| USA Policy | resilient | 17 | Positive |
| USA Policy | right | 17 | Positive |
| USA Policy | great | 16 | Positive |
| USA Policy | integrated | 16 | Positive |
| USA Policy | reasonable | 16 | Positive |
| USA Policy | trust | 16 | Positive |
| USA Policy | commitment | 15 | Positive |
| USA Policy | enough | 15 | Positive |
| USA Policy | good | 15 | Positive |
| USA Policy | survival | 15 | Positive |
| USA Policy | abundance | 14 | Positive |
| USA Policy | adaptive | 14 | Positive |
| USA Policy | compatible | 14 | Positive |
| USA Policy | meaningful | 14 | Positive |
| USA Policy | regard | 14 | Positive |
| USA Policy | transparent | 14 | Positive |
| USA Policy | improve | 13 | Positive |
| USA Policy | sincerely | 13 | Positive |
| USA Policy | variety | 13 | Positive |
| USA Policy | appreciate | 12 | Positive |
| USA Policy | free | 12 | Positive |
| USA Policy | timely | 12 | Positive |
| USA Policy | useful | 12 | Positive |
| USA Policy | beneficial | 11 | Positive |
| USA Policy | recover | 11 | Positive |
| USA Policy | accomplish | 10 | Positive |
| USA Policy | appreciate | 10 | Positive |
| USA Policy | commend | 10 | Positive |
| USA Policy | leading | 10 | Positive |
